# Continuous flow unlocks modular ketones assembly enabled by dynamic orbital selection

**DOI:** 10.1039/d5sc09414c

**Published:** 2026-01-29

**Authors:** Jiayin Wang, Shuangshuang Zhou, Xinyao Hu, Siyu Pan, Xiaohui Zhuang, Jie Li, Rongbo Tang, Yuanyuan Xie, Bin Sun, Can Jin

**Affiliations:** a College of Pharmaceutical Sciences, Collaborative Innovation Center of Yangtze River Delta Region Green Pharmaceuticals, Zhejiang University of Technology Hangzhou Zhejiang 310032 P. R. China jincan@zjut.edu.cn sunbin@zjut.edu.cn; b State Key Laboratory of Green Chemical Synthesis and Conversion, Zhejiang Key Laboratory of Green Manufacturing Technology for Chemical Drugs, Key Laboratory for Green Pharmaceutical Technology and Equipment (Zhejiang University of Technology) of Ministry of Education Deqing Zhejiang 313200 P. R. China

## Abstract

Cross-coupling based on diverse structural motifs offers a powerful strategy for ketone synthesis. However, existing methodologies are often hampered by a narrow substrate scope, non-neutral redox conditions, and poor scalability, particularly for sterically hindered ketones. Herein, we have developed a continuous-flow metallaphotoredox catalytic strategy driven by a dynamic orbital selection mechanism, leveraging the differential bond dissociation energies between various radicals and the metal. This approach enables efficient cross-coupling of aldehydes and carboxylic acids without the need for oxidative addition steps, thereby circumventing the reliance on stoichiometric redox reagents. It demonstrates broad substrate compatibility and excellent functional group tolerance, allowing access to a wide range of ketones, including highly sterically congested frameworks. Notably, the implementation of continuous-flow technology significantly enhances process efficiency and scalability. A successful 100 gram-scale conversion conducted in a flow microreactor within 24 h further underscores the potential of continuous-flow photocatalysis as a sustainable platform for organic synthesis.

## Abstract 2

A continuous-flow metallaphotoredox platform enables access to diverse aliphatic ketones *via* a dynamic orbital selection mechanism, with its practical applicability demonstrated by a successful 100 gram-scale synthesis.

## Graphical abstract

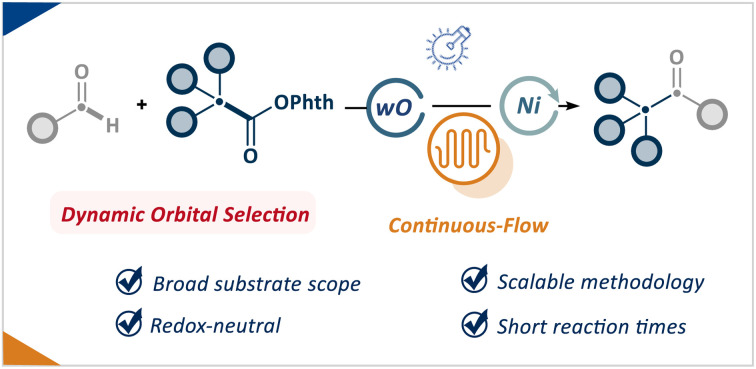

## Introduction

**Scheme 1:**
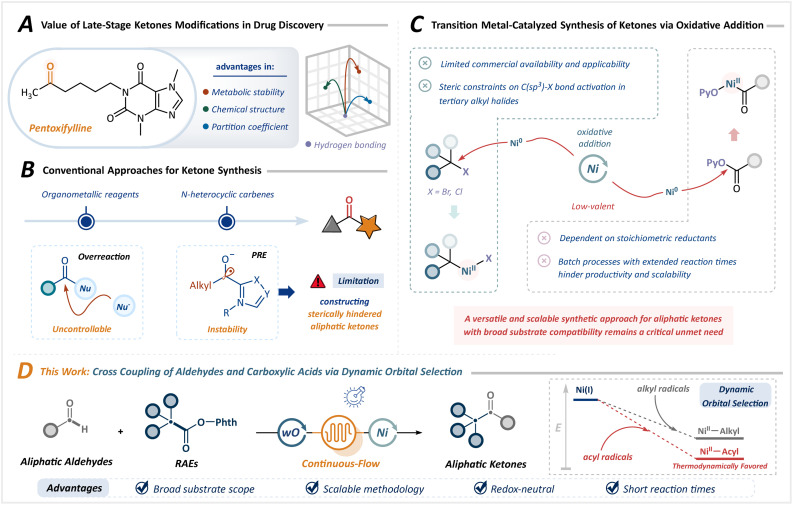
Project design of cross-coupling synthesis strategies for ketones *via* dynamic orbital selection. (A) Value of late-stage ketones modifications in drug discovery. (B) Conventional approaches for ketone synthesis. (C) Transition metal-catalyzed synthesis of ketones *via* oxidative addition. (D) Cross-coupling of aldehydes and carboxylic acids *via* dynamic orbital selection (this work).

The rapid pace of drug discovery has been profoundly influenced by innovations in synthetic chemistry, particularly in structure–activity relationship (SAR) studies, in which chemists design efficient and modular assembly strategies for diverse lead compounds.^1–5^ Ketones serve as essential pharmacophores, significantly enhancing the therapeutic potential of drug candidates by fine-tuning the structural configuration, lipophilicity, and metabolic stability (Scheme 1A).^6–8^ Consequently, the construction of complex ketones from versatile structural motifs has emerged as a particularly attractive objective. While highly reactive organometallic reagents are widely employed, their propensity toward overreaction and limited functional group compatibility often leads to uncontrolled reactivity and constrained utility.^9,10^

Among established ketone synthesis strategies, the *N*-heterocyclic carbene (NHC)-catalyzed Stetter reaction, which proceeds through a polarity reversal process *via* the reductive Breslow intermediate, has been extensively explored.^11–14^ However, its applicability has largely been restricted to aromatic aldehydes. Although Ohmiya *et al.* developed a macrocyclic backbone NHC catalyst capable of activating aliphatic aldehydes, the pronounced steric sensitivity of the Breslow intermediate still impedes engagement with tertiary aldehydes, thereby limiting the practical applicability in the synthesis of sterically demanding aliphatic ketones (Scheme 1B).^14^

Transition metal catalysis offers an alternative avenue, typically involving the coupling of alkyl halides with aldehydes.^15–21^ However, the precise installation of halogen groups remains a considerable challenge, especially in complex drug molecules. Furthermore, the activation of tertiary alkyl halides by low-valent metals (*e.g.*, Ni^0^) is hampered by steric effects, narrowing the substrate scope. Although carboxylic acid derivative precursors allow access to a wide range of structural motifs, their utility is largely limited to sterically unhindered ketones and involves peroxides.^22–25^ Recent advances in reductive cross-coupling have broadened the substrate applicability toward sterically hindered ketones.^26,27^ Nevertheless, the inherent reactivity of low-valent metals mandates stoichiometric reductants and exogenous additives, frequently eroding selectivity (Scheme 1C). Additionally, the heterogeneity imposed by insoluble metals and inorganic salts renders these strategies incompatible with continuous-flow technologies, presenting a bottleneck to scalability.

Despite these advances, a versatile and scalable synthetic approach for aliphatic ketones with broad substrate compatibility remains a critical unmet need. Diverging from conventional cross-coupling paradigms reliant on oxidative addition, radical–radical cross-coupling, particularly the dynamic orbital selection strategy proposed by Macmillan *et al.*, exploits the bond dissociation energies between different radicals and metals to enable highly selective coupling between two transient radicals.^28^ This approach provides access to sterically hindered structures, including quaternary carbon centers, circumventing the intrinsic sensitivity of two-electron oxidative addition in transition metal catalysis. Furthermore, conventional batch reactors are inherently limited by the Lambert–Beer law, rendering scale-up through dimensional amplification profoundly challenging. Photochemical continuous-flow microreactors, which combine the intrinsic advantages of photochemistry with advanced engineering principles, offer a promising alternative.^29–38^ These systems feature short residence times, superior mass transport efficiency, and high surface-to-volume ratios, optimizing photon absorption and significantly improving reaction efficiency while minimizing side reactions. This innovation unlocks transformative potential for metallaphotoredox catalysis in aliphatic ketone synthesis.

Herein, we developed a scalable and practical metallaphotoredox catalytic strategy using continuous-flow photochemistry (Scheme 1D). Employing commercially accessible aldehydes and carboxylic acid-derived redox-active esters (RAEs) as feedstocks, this approach leverages dynamic orbital selection to enable a single metal center to sequentially and selectively discriminate between acyl and alkyl radicals. The key to this strategy lies in decoupling the traditional activation pathways of metal catalysts and electrophilic substrates, enabling independent regulation of radical generation and coupling steps *via* dual catalytic cycles. This allows for precise control over C(sp^3^)–C(sp^2^) bond formation through the dynamic equilibrium between radicals and metal-bound states, without the need for stoichiometric redox reagents. Building on this, it effectively integrates the inherent advantages of flow chemistry and photochemistry, successfully overcoming persistent challenges in substrate scope, selectivity, and scalability. This approach offers an efficient, modular, and broadly applicable route to diverse ketones, including highly sterically congested variants.

## Results and discussion

To initiate this investigation, a photocatalytic radical cross-coupling between RAE and acetaldehyde was explored as a model reaction under continuous-flow conditions. Based on our extensive experience in the HAT domain and considering the high charge density of aldehyde C(sp^2^)–H bonds, we selected cost-effective TBADT as the photocatalyst.^39,40^ Following comprehensive optimization (see SI for details), the optimal conditions were established, as detailed in Table 1. A solution of the RAE, 5 mol% TBADT, 10 mol% NiBr_2_ (DME) and dtbbpy, 0.5 equiv. of DIPEA, and 3.0 equiv. of acetaldehyde in MeCN was degassed and irradiated at 370 nm in a flow reactor under a nitrogen atmosphere. This mixture was pumped through the flow reactor with a residence time (*t*_R_) of 10 min, affording the desired product 21 in 82% yield (entry 1). Control experiments revealed that the presence of the photocatalyst, nickel catalyst, ligand, light, and base was essential for the successful completion of this transformation (entries 2–5). The presence of H_2_O and air significantly inhibited the reaction progress (entries 6 and 7). Alternative ligands also facilitated acylation, albeit with lower yields (entry 8). Compared to the 9 h needed in a batch reactor, this process offers a substantial increase in transformation efficiency, representing superior performance in both reaction rate and productivity (entry 9).

Control reactions of optimized conditions^a^

**Table:** 

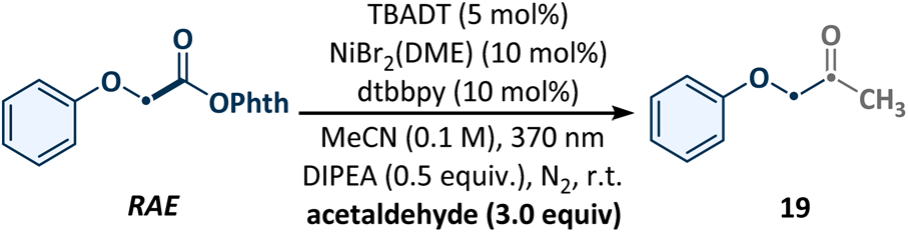		
Entry	Deviation from optimal conditions	Yield (%)^b^
1	None	82
2	No photocatalyst	0
3	No nickel catalyst	0
4	No light	0
5	No DIPEA	Trace
6	H_2_O (1.0 equiv.) as an additive	Trace
7	Air instead of N_2_	17
8	L1–L7 instead of dtbbpy	As shown ligand
9	9 h, in a batch reactor	75

a Reactions performed with NHPI ester (1 mmol, 1.0 equiv.), TBADT (5 mol%), NiBr_2_ (DME) (10 mol%), dtbbpy (10 mol%), DIPEA (0.5 equiv.), acetaldehyde (3.0 equiv.), and MeCN (0.1 M), at room temperature, 2 × 40 W 370 nm LEDs, 1.2 mL min^−1^, *t*_R_ = 10 min.

b Isolated yields.

c Reactions performed with NHPI ester (1 mmol, 1.0 equiv.), TBADT (5 mol%), NiBr_2_ (DME) (10 mol%), ligand (10 mol%), PMDETA (1.5 equiv.), acetaldehyde (3.0 equiv.), MeCN (0.1 M), at room temperature, *t*_R_ = 10 min. See SI for more details. TBADT: tetrabutylammonium decatungstate. Dtbbpy: 4,4′-di-*tert*-butyl-2,2′-dipyidyl. DIPEA: *N*,*N*-diisopropylethylamine. PMDETA: pentamethyldiethylenetriamine.

**Table:** 

Ligand^c^
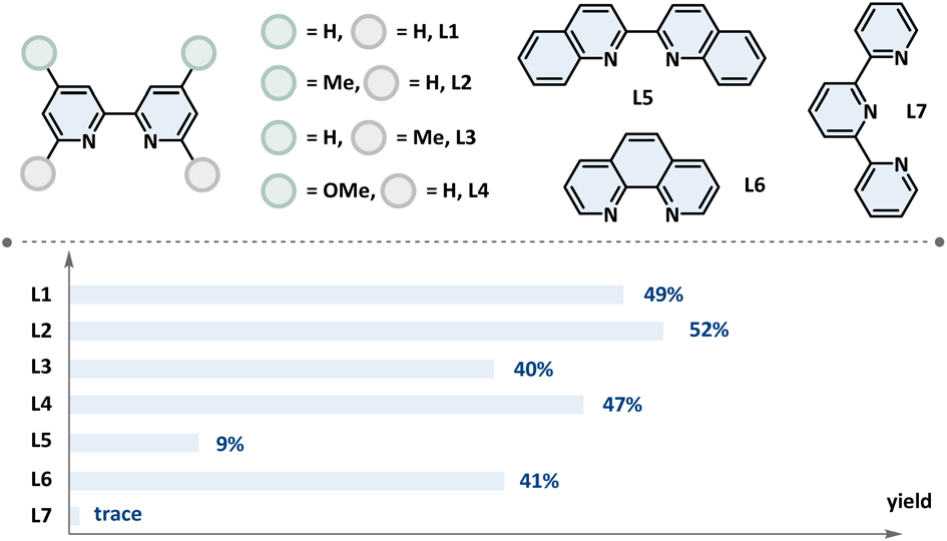

**Scheme 2:**
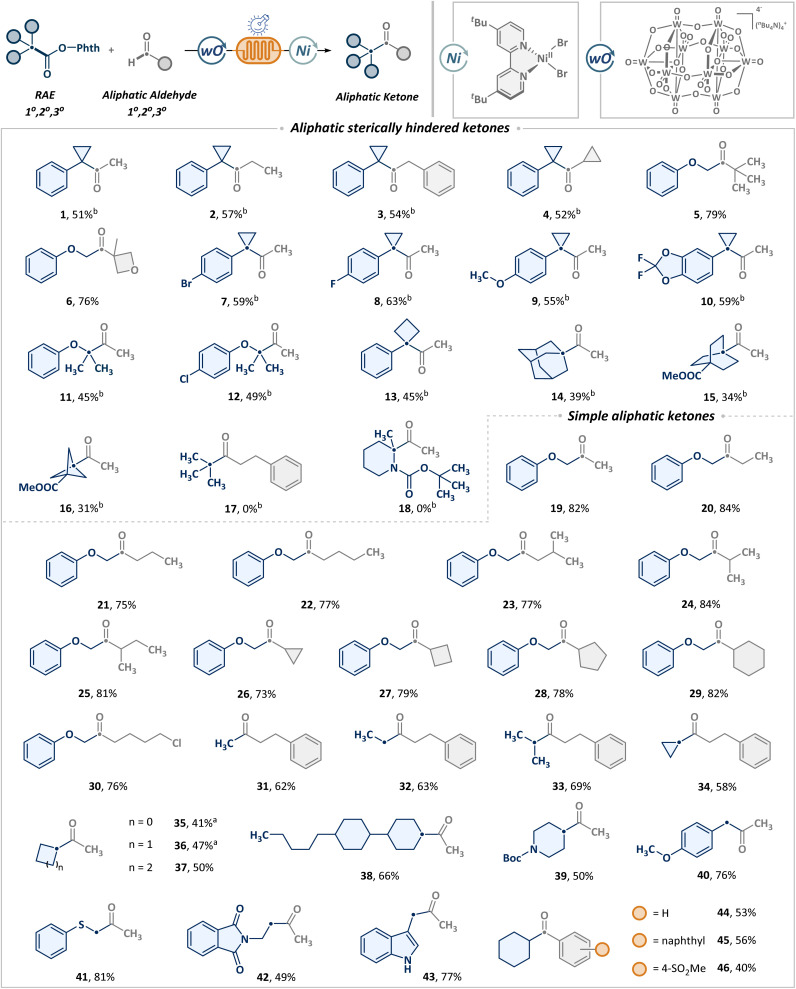
Substrate scope of metallaphotoredox-enabled construction of ketones. Reactions performed with NHPI ester (1 mmol, 1.0 equiv.), TBADT (5 mol%), NiBr_2_ (DME) (10 mol%), dtbbpy (10 mol%), DIPEA (0.5 equiv.), acetaldehyde (3.0 equiv.), MeCN (0.1 M), at room temperature, 40 W 370 nm LEDs, 1.2 mL min^−1^, *t*_R_ = 10 min, isolated yields. ^*a*^Yields were determined by ^1^H NMR with 1,3,5-trimethoxybenzene as an internal standard. ^*b*^0.4 mL min^−1^, *t*_R_ = 30 min. See SI for more details.

Building on the optimized conditions outlined in Table 1, we evaluated the synthetic versatility of the continuous-flow platform for the synthesis of diverse ketones from aldehydes and RAEs on a synthetically relevant scale (1 mmol), as shown in Scheme 2. Given the pervasive incorporation of sterically hindered ketones in molecular scaffolds and the inherent synthetic difficulties, we initially targeted the synthesis of sterically hindered ketones (1–18). Pleasingly, a broad range of aliphatic aldehydes, including primary (1–3) and secondary (4), were compatible with the tertiary benzyl cyclopropyl radical, leading to the formation of sterically hindered alkyl ketones in moderate to good yields. Tertiary aldehydes (5, 6) can also be employed in similar transformations to construct sterically hindered scaffolds. Besides, this process was found to exhibit broad applicability across a range of tertiary benzyl cyclopropyl radicals, irrespective of the electronic nature of arenes, whether bearing electron-withdrawing (7, 8) or electron-donating groups (9, 10). Additionally, heteroatom α-carbon radicals (11, 12) and benzylic radicals incorporating four-membered ring strain (13) were also tolerated well. Notably, substrates featuring Br (7) and Cl (12) offer a refined handle for further modification. The scope also included steric bridgehead radicals, such as those derived from adamantane (14), bicyclo[2.2.2]octane (15) and spirocyclohexane (16), albeit with a slight reduction in yield. Regrettably, the unbiased *t*-butyl radical was unable to deliver the anticipated product (17). Nonetheless, acyl electrophiles derived from *t*-butyl aldehyde (5) demonstrated exceptional functional group compatibility, thereby allowing for the successful formation of *t*-butyl-substituted ketones *via* replacement of scaffold precursor modules. Furthermore, the target product cannot be obtained from the heteroatom-substituted tertiary carbon (18) either.

Beyond establishing the utility of synthesizing sterically hindered ketones, further generality was evaluated regarding the formation of non-sterically hindered ketones. Satisfyingly, a series of straight-chain (19–22) and branched (23) aliphatic primary aldehydes underwent coupling with α-position primary radicals featuring oxygen. The α-branched alkanals also exhibited favorable reactivity, with no significant impact observed from cyclic scaffolds of varying strain (24–29). The scope was successfully extended to long-chain primary aldehydes bearing distal halogens. Notably, alkyl chlorides (30) showcased excellent compatibility with nucleophilic phthalimide groups, providing a robust platform for subsequent derivatization. In addition to aldehydes, we further explored the potential of diverse alkanoic acids to access structurally varied asymmetric ketones (31–44). Simple acetic acid (31) served as an effective methyl source to furnish terminal methyl ketones. A range of primary (32) and secondary (33–38) carboxylic acids, including those with different ring strain frameworks (34–38), can also undergo smooth cross-coupling with aldehydes. Furthermore, the presence of heteroatoms such as nitrogen (39), oxygen (40), and sulfur (41) has no adverse effect on the transformation, showing excellent functional group tolerance. Among the heterocyclic scaffolds, indole-derived acids (43) exhibited superior reactivity compared to phthalimide (42). Additionally, the method was also compatible with aromatic aldehydes (44–46), further attesting to its broad applicability.

**Scheme 3:**
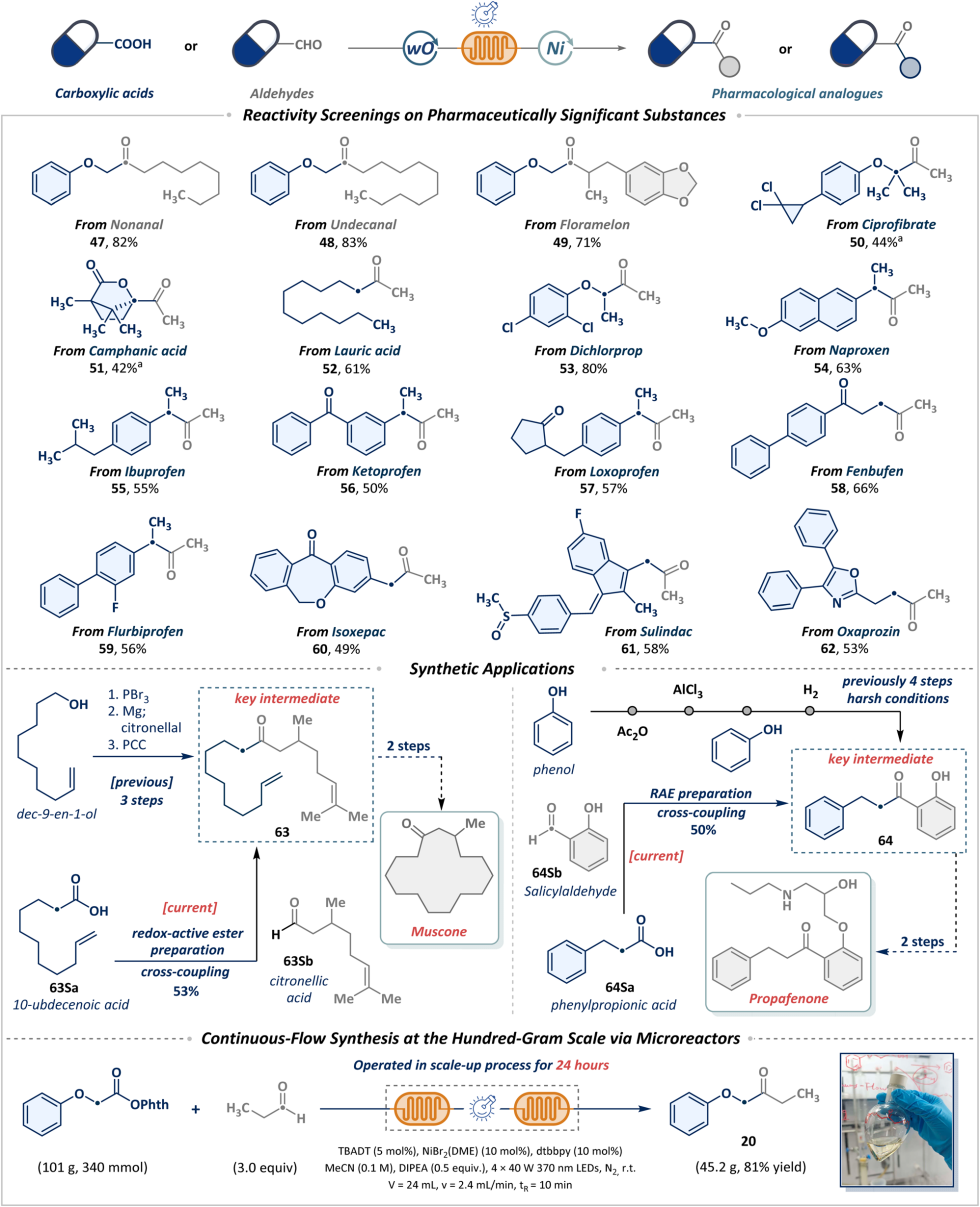
Application of the construction of ketones to drugs and active intermediates and continuous-flow synthesis at the hundred-gram scale. Reactions performed with NHPI ester (1 mmol, 1.0 equiv.), TBADT (5 mol%), NiBr_2_ (DME) (10 mol%), dtbbpy (10 mol%), DIPEA (0.5 equiv.), acetaldehyde (3.0 equiv.), MeCN (0.1 M), at room temperature, 2 × 40 W 370 nm LEDs, 1.2 mL min^−1^, *t*_R_ = 10 min, isolated yields. ^*a*^0.4 mL min^−1^, *t*_R_ = 30 min. See SI for more details.

Building on foundational studies of substrate compatibility and functional group tolerance, we seek to leverage the cross-coupling strategy for the late-stage functionalization of pharmaceuticals, natural products, and bioactive molecules. As depicted in Scheme 3, natural aldehydes such as nonanal (47), undecanal (48), and floramelon (49) were efficiently converted into ketones. Motivated by the prevalence of carboxylic acids in bioactive molecules and their commercial availability, alongside the pivotal role of the acetyl group in modulating pharmaceutical efficacy, we systematically explored cross-coupling between a range of complex carboxylic acid derivatives (50–62) and acetaldehyde. Notable examples include the hypolipidemic agent ciprofibrate (50) and the natural product camphanic acid (51), which can efficiently yield sterically constrained terminal methyl ketones. Furthermore, acetylated derivatives of lauric acid (52), dichlorprop (53), naproxen (54), ibuprofen (55), ketoprofen (56), loxoprofen (57), fenbufen (58), flubiprofen (59), and isoxepac (60) were successfully integrated into this strategy. Notably, nonsteroidal anti-inflammatory drugs (NSAIDs), such as sulindac (61) and oxaprozin (62), also demonstrated compatibility with this methodology. These results highlight the capability of this protocol to rapidly attain pharmacologically relevant structural diversity from readily accessible or abundant starting materials. The efficacy of this strategy is further underscored by its successful implementation in the streamlined synthesis of complex molecular intermediates. Specifically, ketone 63, a critical intermediate en route to muscone, was efficiently synthesized from simple building blocks. Equally compelling, as the key intermediate in the synthesis of propafenone, ketone 64 could also be obtained in 50% yield from inexpensive and abundant starting materials. In a practical context, a gram-scale transformation was efficiently achieved through continuous production using flow microreactors. The metallaphotoredox cross-coupling of RAEs with propionaldehyde was conducted continuously in a tandem microreactor system, affording ketone product 20 in 45.2 g (81% yield). This demonstrates the robustness and excellent scalability of the photochemical system for long-term operation.

**Scheme 4:**
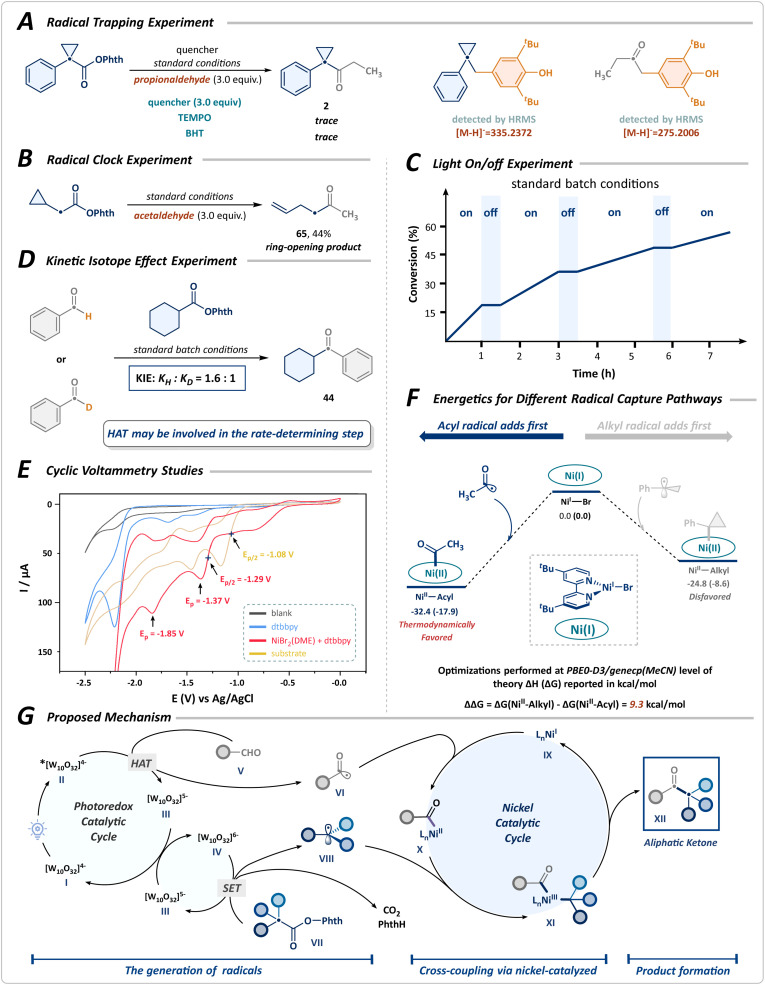
Mechanistic studies. (A) Radical trapping experiment. (B) Radical-clock experiment. (C) Light on/off experiment. (D) Kinetic isotope effect experiment. (E) Cyclic voltammetry studies. (F) Energetics for different radical capture pathways. (G) Proposed mechanism.

Subsequently, preliminary mechanistic experiments, shown in Scheme 4 and the SI, were performed to investigate the mechanism of cross-coupling. The transformation was notably suppressed upon the addition of the radical scavengers 2,2,6,6-tetramethylpiperidin-1-oxy (TEMPO) and butylated hydroxytoluene (BHT), with the corresponding alkyl and acyl radical adducts detected by HRMS (Scheme 4A). The radical-clock experiment, using the NHPI ester derived from cyclopropyl acetic acid, afforded the desired ring-opening product 65 with a yield of 44% (Scheme 4B). These results suggest a radical-mediated mechanistic pathway. Besides, the light on/off experiment confirmed that continuous illumination was crucial for the progression of the transformation, thereby ruling out the possibility of a radical chain mechanism (Scheme 4C). Furthermore, we conducted a parallel kinetic isotope effect (KIE) study employing benzaldehyde as the substrate, observing negligible differences in reaction rate between benzaldehyde and its deuterated forms. This finding indicates that the HAT process mediated by photoexcited decatungstate may be the rate-determining step in the cross-coupling reaction (Scheme 4D). The cyclic voltammogram studies confirm that the reduction of nickel species and RAEs by the reduced state of decatungstate (*E*_1/2_^red^ [W_10_O_32_]^5−^/[W_10_O_32_]^6−^ = −1.35 V *vs.* Ag/AgCl) is thermodynamically favorable (Scheme 4E).^41^

In the catalytic cycle, both the acyl and alkyl radicals generated can coordinate to the nickel, forming either Ni^II^-acyl or Ni^II^-alkyl species. To further elucidate the reaction mechanism, density functional theory (DFT) calculations were performed to verify the initiation mechanism of Ni-catalyzed acyl and alkyl radical sequential-coupling (Scheme 4F). These results suggested that the radical capture processes for both species proceed without any barriers. Thermodynamic analysis further reveals that the capture of the acyl radical to Ni^I^ species is significantly favored (ΔΔ*G* = 9.3 kcal mol^−1^) and is an irreversible process. In contrast, the capture of the alkyl radical is thermodynamically less favorable.

Based on the above results, we proposed a plausible mechanism involving the generation of both alkyl and acyl radicals, followed by a nickel-catalyzed sequential radical cross-coupling, as illustrated in Scheme 4G. Upon photoexcitation of the decatungstate anion I, rapid intersystem crossing facilitates the generation of the triplet excited state II under near-ultraviolet irradiation. The highly electrophilic excited species II undergoes a HAT process, driven by polarity matching with the hydridic C(sp^2^)–H bond of aldehydes V, leading to the formation of an acyl radical VI and the reduced decatungstate III. Subsequently, the acyl radical VI is captured by the nickel catalyst IX, forming a complex X, while the reduced decatungstate III undergoes disproportionation to reconstitute the ground-state decatungstate I along with the doubly reduced decatungstate IV. The species IV then mediates a single-electron reduction of the RAE VII, initiating its decarboxylation to yield an alkyl radical VIII, thus perpetuating the catalytic cycle of the decatungstate. The alkyl radical VIII is selectively sequestered by the nickel complex X, which undergoes reductive elimination to produce a ketone XII and regenerate Ni^I^IX, thereby enabling the initiation of a new catalytic cycle.

## Conclusions

In conclusion, we present a continuous-flow metallaphotoredox strategy based on dynamic orbital selection, enabling direct cross-coupling of carboxylic acids and aldehydes without stoichiometric redox reagents. This approach exhibits remarkable functional group tolerance and broad substrate scope, efficiently accessing sterically hindered aliphatic ketones that are difficult to access *via* traditional methods. The robustness and scalability of the process were validated through continuous in-flow microreactors. Furthermore, the synthetic utility of this strategy was demonstrated by its successful application in preparing key ketone intermediates and in the late-stage functionalization of complex molecules. This work establishes an efficient and scalable paradigm for constructing challenging C(sp^3^)–C(sp^2^) bonds, showcasing the promising potential of continuous-flow photocatalysis in advancing synthetic chemistry.

## Experimental

### General procedure for ketone synthesis in continuous flow

An oven-dried vial equipped with a stirring bar was charged sequentially with TBADT (0.05 mmol, 5 mol%), NiBr_2_ DME (0.1 mmol, 10 mol%), dtbbpy (0.1 mmol, 10 mol%), a Teflon-coated magnetic stir bar, and MeCN (10.0 mL). The reaction mixture was stirred at ambient temperature for 30 min. Subsequently, RAEs (1 mmol, 1.0 equiv.), DIPEA (0.5 equiv.) and the corresponding aldehydes (3.0 equiv.) were added sequentially. Subsequently, the vial was sealed with a rubber septum, and the solution was backfilled with nitrogen three times. The solution was loaded onto a filling loop (PFA capillary tubing: 1.6 mm OD, 1.0 mm ID) that had been sparged with N_2_. By using a peristaltic pump, the solution was delivered to the flow reactor (UFlow) with a PFA coil (1.6 mm OD, 1 mm ID, 12.0 mL volume). The solution was pumped with a total flow rate of 1.2 mL min^−1^ flow rate (10 min of residence time). The crude reaction mixture was extracted with DCM (3 × 20 mL), and the combined organic extracts were dried (anhydrous Na_2_SO_4_). The solvent was removed *via* rotary evaporation, and the residue was purified *via* silica gel chromatography to give the corresponding products.

## Author contributions

J. Y. W., B. S. and C. J. discovered and developed the reaction. J. Y. W., S. S. Z., B. S., and C. J. conceived and designed the investigations. J. Y. W., S. S. Z., X. Y. H., S. Y. P., X. H. Z., J. L., R. B. T. and Y. Y. X. performed the experiments. J. Y. W., B. S. and C. J. wrote the manuscript.

## Conflicts of interest

There are no conflicts to declare.

## Supplementary Material

SC-OLF-D5SC09414C-s001

## Data Availability

The data supporting this article have been included as part of the supplementary information (SI). Supplementary information: Table 1 and Schemes 2–4, NMR spectra and further experimental details. See DOI: https://doi.org/10.1039/d5sc09414c.

## References

[cit1] Castellino N. J., Montgomery A. P., Danon J. J., Kassiou M. (2023). Chem. Rev..

[cit2] Cernak T., Dykstra K. D., Tyagarajan S., Vachal P., Krska S. W. (2016). Chem. Soc. Rev..

[cit3] Walters W. P., Green J., Weiss J. R., Murcko M. A. (2011). J. Med. Chem..

[cit4] Wang Y., Haight I., Gupta R., Vasudevan A. (2021). J. Med. Chem..

[cit5] Blakemore D. C. (2018). et al.. Nat. Chem..

[cit6] Jabeen I., Pleban K., Rinner U., Chiba P., Ecker G. F. (2012). J. Med. Chem..

[cit7] Kuhn B., Mohr P., Stahl M. (2010). J. Med. Chem..

[cit8] McDonagh A. F., Lightner D. A. (2007). J. Med. Chem..

[cit9] Brunet J.-J., Chauvin R. (1995). Chem. Soc. Rev..

[cit10] Lawrence N. J. (1998). J. Chem. Soc., Perkin Trans..

[cit11] Liu X., Xu S., Chen H., Yang Y. (2024). ACS Catal..

[cit12] Tan C.-Y., Kim M., Hong S. (2023). Angew. Chem., Int. Ed..

[cit13] Ishii T., Kakeno Y., Nagao K., Ohmiya H. (2019). J. Am. Chem. Soc..

[cit14] Kakeno Y., Kusakabe M., Nagao K., Ohmiya H. (2020). ACS Catal..

[cit15] Cheng W.-M., Shang R. (2020). ACS Catal..

[cit16] Chan A. Y. (2022). et al.. Chem. Rev..

[cit17] Ruzi R., Liu K., Zhu C., Xie J. (2020). Nat. Commun..

[cit18] Ai Y., Ye N., Wang Q., Yahata K., Kishi Y. (2017). Angew. Chem., Int. Ed..

[cit19] Hu Z. (2025). et al.. Org. Lett..

[cit20] Zhang X., MacMillan D. W. C. (2017). J. Am. Chem. Soc..

[cit21] Fan P., Zhang C., Zhang L., Wang C. (2020). Org. Lett..

[cit22] Li T., Xu Z., Huang Y., Zu W., Huo H. (2025). J. Am. Chem. Soc..

[cit23] Yan X.-B. (2025). et al.. J. Am. Chem. Soc..

[cit24] Ling B. (2024). et al.. Angew. Chem., Int. Ed..

[cit25] Yang S. (2025). et al.. Org. Chem. Front..

[cit26] Wang J., Cary B. P., Beyer P. D., Gellman S. H., Weix D. J. (2019). Angew. Chem., Int. Ed..

[cit27] Xi X. (2022). et al.. Angew. Chem., Int. Ed..

[cit28] Großkopf J., Gopatta C., Martin R. T., Haseloer A., MacMillan D. W. C. (2025). Nature.

[cit29] Zondag S. D. A. (2024). et al.. Nat. Chem. Eng..

[cit30] Crawford R., Baumann M. (2025). Adv. Synth. Catal..

[cit31] Laporte A. A. H., Masson T. M., Zondag S. D. A., Noël T. (2024). Angew. Chem., Int. Ed..

[cit32] Pulcinella A. (2025). et al.. Angew. Chem., Int. Ed..

[cit33] Raymenants F., Masson T. M., Sanjosé-Orduna J., Noël T. (2023). Angew. Chem., Int. Ed..

[cit34] Yuan X. (2023). et al.. Chin. J. Catal..

[cit35] Pasca F. (2025). et al.. JACS Au.

[cit36] Bonciolini S., Pulcinella A., Noël T. (2025). J. Am. Chem. Soc..

[cit37] Pulcinella A. (2025). et al.. Nat. Commun..

[cit38] Slattery A. (2024). et al.. Science.

[cit39] Sun B. (2024). et al.. ACS Catal..

[cit40] Sun B. (2025). et al.. Angew. Chem., Int. Ed..

[cit41] Hu X., Cheng-Sánchez I., Kong W., Molander G. A., Nevado C. (2024). Nat. Catal..

