# Supplementary material for: Continuous flow unlocks modular ketones assembly enabled by dynamic orbital selection
Source: Chem Sci. 2026 Jan 29;17(14):7010–7. doi: 10.1039/d5sc09414c (PMC12897494; doi:10.1039/d5sc09414c)

Supplementary Information (SI) for Chemical Science.
This journal is © The Royal Society of Chemistry 2026

Supplementary Information

Continuous Flow Unlocks Modular Ketones Assembly Enabled by Dynamic Orbital Selection

Jiayin Wang,[a] Shuangshuang Zhou,[a] Xinyao Hu,[a] Siyu Pan,[a] Xiaohui Zhuang,[a] Jie Li,[a] Rongbo Tang,[a] Yuanyuan Xie,[a, b] Bin Sun,*[a, b] and Can Jin*[a, b]

[a]College of Pharmaceutical Sciences, Collaborative Innovation Center of Yangtze River Delta Region Green Pharmaceuticals, Zhejiang University of Technology, Hangzhou, Zhejiang 310032, P. R. China;

[b]State Key Laboratory of Green Chemical Synthesis and Conversion, Zhejiang Key Laboratory of Green Manufacturing Technology for Chemical Drugs, Key Laboratory for Green Pharmaceutical Technology and Equipment (Zhejiang University of Technology) of Ministry of Education, Deqing, Zhejiang 313200, P. R. China;

*Corresponding author. Email: jincan@zjut.edu.cn (C. Jin); sunbin@zjut.edu.cn (B. Sun)

1. **General Information** S1
2. **Preparation of Catalysts and Substrates** S2
3. **Reaction Setup** S3
4. **Reaction Optimization and Control Experiments** S4
5. **General Procedure for the Dehydrogenation of Ketones** S7
6. **Mechanistic Investigation** S8
7. **Analytic Data of Products** S19
8. **Supplementary References** S45
9. **NMR Spectra** S46

1. General Information

Chemicals and solvents were purchased from commercial suppliers and used as received. Tetrabutylammonium decatungstate[1] was synthesized according to literature. Uncommercial available substrates were synthesized according to literature. ^{1}H NMR, ^{13}C NMR, ^{19}F NMR spectra were recorded on a Bruker AV 600 or AV 400 NMR spectrometer. Chemical shifts were reported in parts per million (ppm) and calibrated using residual undeuterated solvent as an internal reference ($CDCl_3$: 7.26 ppm ^{1}H NMR, 77.0 ppm ^{13}C NMR). Multiplicity was indicated as follows: s (singlet), d (doublet), t (triplet), q (quartet), m (multiplet), dd (doublet of doublet), dt (doublet of triplet), td (triplet of doublet), ddd (doublet of doublet of doublet), dddd (doublet of doublet of doublet of doublet). All high-resolution mass spectra (HRMS) were obtained on Agilent 6545 LC/Q-TOF spectrometer. UV-vis absorption spectra were taken at ambient temperature using MAPADA P6 spectrofluorometer. Column chromatography was carried out with 200-300 mesh silica gel. Analytical thin layer chromatography (TLC) was performed with glass TLC plates (silica gel GF-254). Visualization was achieved by short wave (254 nm) ultraviolet light or by staining with I_2 or Phosphomolybdic Acid (PMA), followed by heating.

Photochemical reactions were performed with 40 W 370 nm LED purchased from Anhui Kemi Instrument Co., LTD. (http://www.ahkemi.com/).

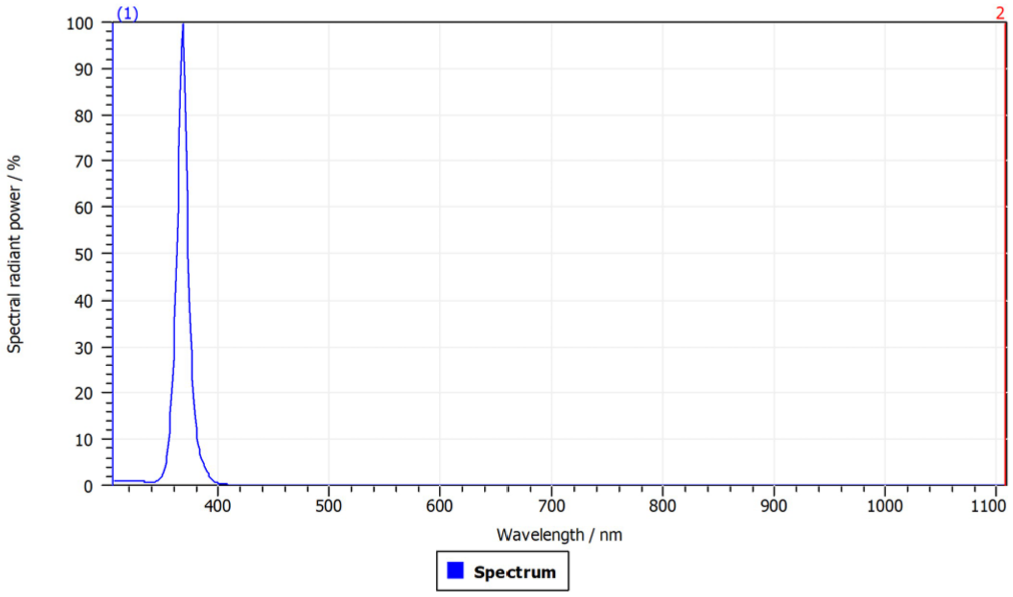

Supplementary Fig. 1 Emission spectrum of 40 W 370 nm LED strips. Maximum emission at around 370 nm.

2. Preparation of Catalysts and Substrates

2.1 Preparation of tetrabutylammonium decatungstate (TBADT):

To a 2 L beaker wrapped in aluminum foil for insulation and equipped with a stir bar were added tetrabutylammonium bromide (4.80 g, 14.9 mmol, 0.49 equiv.) and deionized water (1600 mL). In a separate 4 L beaker wrapped in aluminum foil for insulation and equipped with a stir bar were added $Na_2WO_4•2H_2O$ (10 g, 30.3 mmol, 1.0 equiv.) and deionized water (1600 mL). Both solutions were rapidly stirred and heated to 90 °C. When both solutions reached 90 °C, concentrated HCl was added to each solution until pH stabilized at 2. At this point, the acidified solutions were combined in the 4 L beaker, and the resultant suspension was stirred at 90 °C for an additional 30 minutes. The reaction mixture was cooled to room temperature, then filtered through a pad of silica gel. The solids were washed with water and left to dry under vacuum. When the silica-supported solids were dry, the receiving flask was exchanged, and the pad was washed with 3 × 200 mL acetonitrile. The filtrate was collected and solvent was removed. The crude residue was thoroughly dried under vacuum, dissolved in minimal hot acetonitrile, then placed in the freezer at 0 °C for 12 hours. The solids were collected on a filter, washed with minimal cold acetonitrile, then dried under vacuum. The filtrate was reconcentrated, dissolved in minimal hot acetonitrile, and crystallized again to afford a second crop of TBADT. Isolated as white solid. UV-Vis and cyclic voltammetry characterization is consistent with literature data.[1]

2.2 Preparation of NHPI Redox-Active Esters[2]:

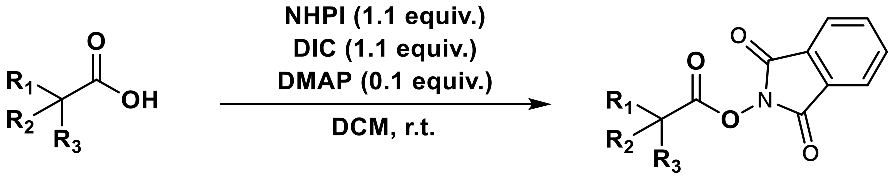

A round-bottom flask tube was charged with carboxylic acid (1.0 equiv), *N*-hydroxyphthalimide (1.1 equiv.), and DMAP (0.1 equiv). DCM was added (0.1 M), and the mixture was stirred vigorously. DIC (1.1 equiv.) was then added dropwise, and the reaction mixture was allowed to stir until the acid was consumed (determined by TLC). Upon completion, the mixture was concentrated and was directly purified via silica gel column chromatography to afford the activated ester. If necessary, the NHPI redox-active ester could be further recrystallized from DCM/MeOH.

3. Reaction Setup

3.1 Flow reactor

For all flow experiments a homemade, 3D-printed reactor was adopted (Fig. 3). The reactor consists of a lid to host the lamp and a support around which a PFA tubing (1.6 mm OD, 1 mm ID) is coiled; a fan is mounted on the bottom of the reactor for cooling. The coil is inserted in a cylindrical plastic body with holes to allow the air flow to escape the reactor and keep the temperature stable around 35 °C. Additional details on the reactor is consistent with literature data.[3]

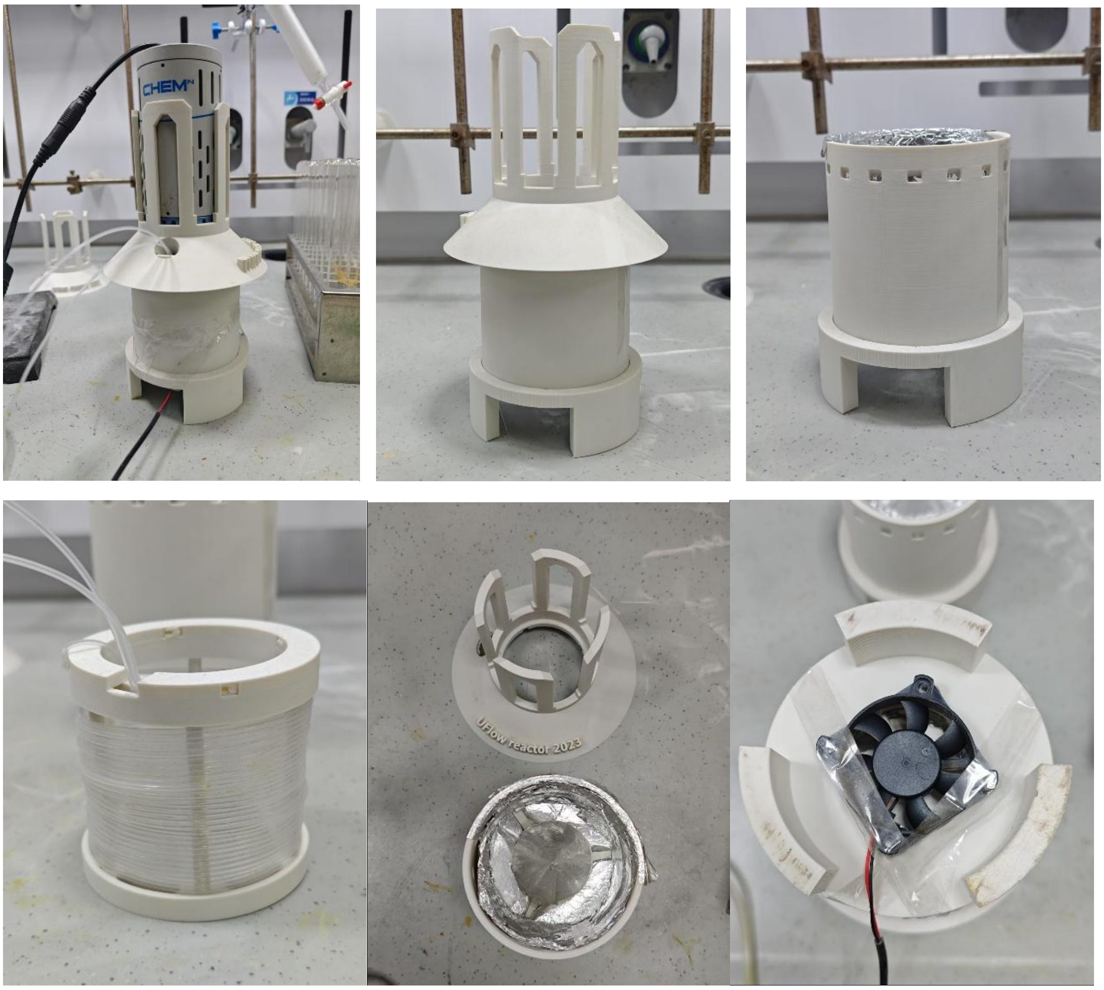

Supplementary Fig. 2 Overview of the 3D-printed flow reactor.

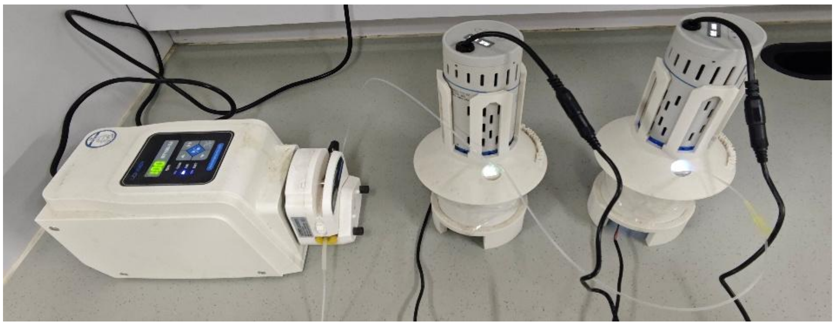

Supplementary Fig. 3 Reaction vessel and reaction set-up (1 mmol scale).

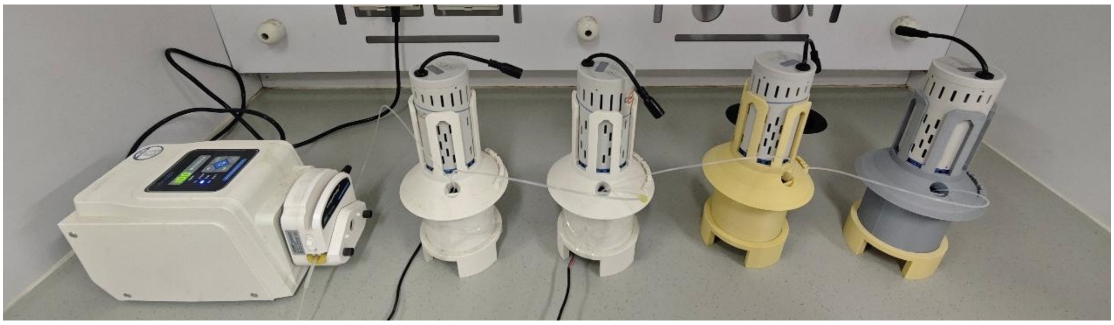

Supplementary Fig. 4 Reaction vessel and reaction set-up (gram scale).

4. Reaction Optimization and Control Experiments

4.1 Supplementary Table 1 Evaluation of nickel catalyst and ligand.

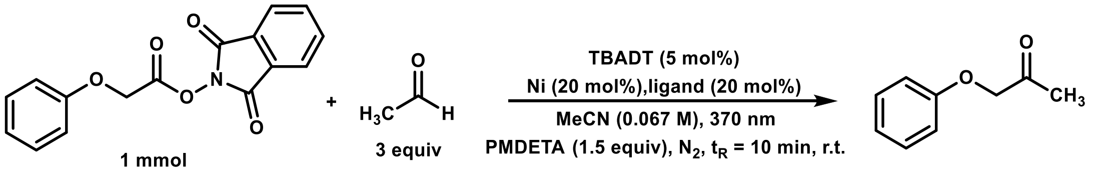

entry	Ni catalyst	ligand	yield (%)[a]
**1**	**$NiBr_2$(DME)**	**4,4'-Di-tert-butyl-2,2'-dipyidyl (dtbbpy)**	**55**
2	$NiCl_2$(DME)	dtbbpy	39
3	$NiBr_2 \cdot 3H_2O$	dtbbpy	46
4	$Ni(acac)_2$	dtbbpy	trace
5	$NiBr_2$(DME)	2,2'-bipyridine	49
6	$NiBr_2$(DME)	1,10-phenanthroline	41
7	$NiBr_2$(DME)	4,4'-dimethoxy-2,2'-bipyridyl	47

8	$NiBr_2$(DME)	2,2':6',2''-terpyridine	trace
9	$NiBr_2$(DME)	1.1'-binaphthyl-2.2'-diphemyl phosphine	n.d.
10	$NiBr_2$(DME)	6,6'-dimethyl-2,2'-dipyridine	40
11	$NiBr_2$(DME)	2,2'-biquinoline	9
12	$NiBr_2$(DME)	4,4'-dimethyl-2,2'-bipyridyl	52

[a]Isolated yields.

4.2 Supplementary Table 2 Evaluation of the solvent and light source.

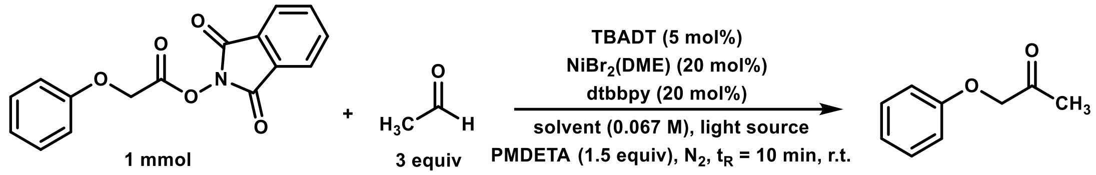

entry	solvent	light source	yield (%)[a]
1	Acetone	370 nm	45
**2**	**MeCN**	**370 nm**	**55**
3	EA	370 nm	6
4	DCE	370 nm	trace
5	DMSO	370 nm	trace
6	MeCN	390 nm	50
7	MeCN	405 nm	31
8	MeCN	435 nm	trace
9	MeCN	455 nm	n.d.

[a]Isolated yields.

4.3 Supplementary Table 3 Evaluation of the base sources and amounts of base.

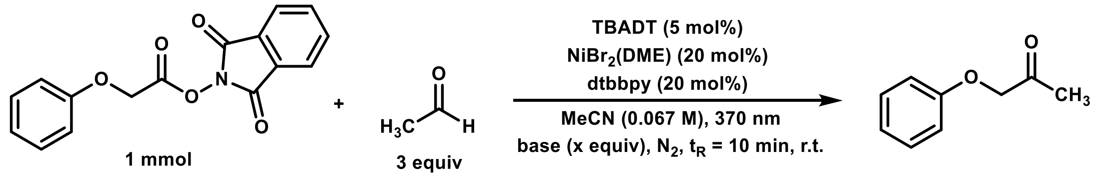

entry	base sources	amounts of base	yield (%)[a]

1	DBU	1.5 equiv	trace
2	DIPEA	1.5 equiv	82%
3	PMDETA	1.5 equiv	55%
4	pyridine	1.5 equiv	13%
5	$NaHCO_3$	1.5 equiv	45%
6	TMG	1.5 equiv	31%
7	Et_3N	1.5 equiv	33%
8	DIPEA	0.1 equiv	39%
**9**	**DIPEA**	**0.5 equiv**	**82%**
10	DIPEA	1.0 equiv	81%

[a]Isolated yields.

4.4 Supplementary Table 4 Evaluation of the amounts of photocatalyst, nickel, ligand and solvent.

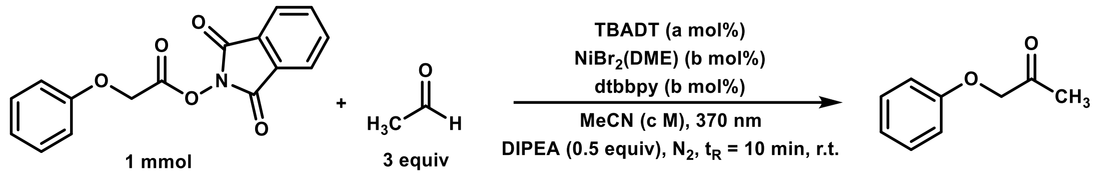

entry	TBADT (a mol%)	Ni and ligand (b mol%)	MeCN (c M)	yield (%)[a]
1	1	20	0.067	31
2	3	20	0.067	67
3	5	20	0.067	82
4	7	20	0.067	82
5	5	5	0.067	59
6	5	10	0.067	82
7	5	15	0.067	83
8	5	10	0.2	74
**9**	**5**	**10**	**0.1**	**82**
10	5	10	0.05	78

[a]Isolated yields.

4.5 Supplementary Table 5 Control experiments on reaction parameters.

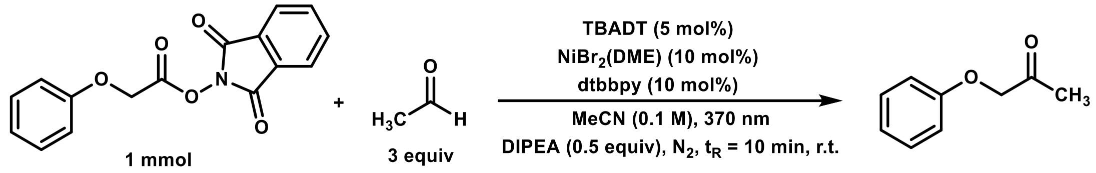

entry	deviation	yield (%)[a]
1	none	82
2	no photocatalyst	0
3	no nickel	0
4	no light	0
5	no DIPEA	trace
6	H_2O (1.0 equiv) as an additive	trace

[a]Isolated yields.

4.5 Supplementary Table 5 Evaluation of the residence time.

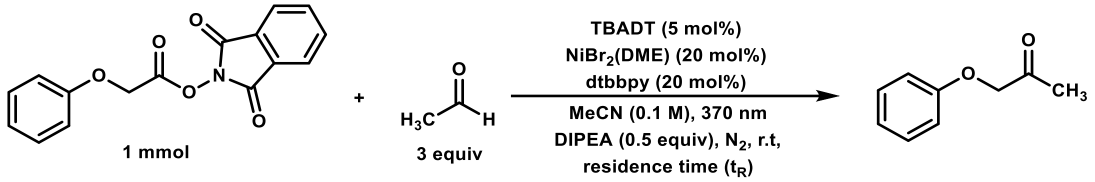

entry	residence time (t_R) (min)	yield (%)[a]
1	7.5	74
**2**	**10**	**82**
3	15	77
4	30	74

[a]Isolated yields.

5. General Procedure for the Construction of Ketones

5.1 General procedure: ketones synthesis in continuous flow

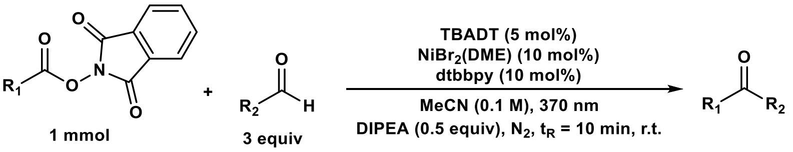

To an oven-dried vial equipped with a stirring bar were charged sequentially with TBADT (0.05 mmol, 5 mol%), $NiBr_2$·DME (0.1 mmol, 10 mol%), dtbbpy (0.1 mmol, 10 mol%), a Teflon-coated magnetic stir bar, and MeCN (10.0 mL). The reaction mixture was stirred at ambient temperature for 30 minutes. Subsequently, RAEs (1 mmol, 1.0 equiv.), DIPEA (0.5 equiv.) and corresponding aldehydes (3.0 equiv.) were added sequentially. Subsequently, the vial was sealed with a rubber septum and the solution was backfilled with nitrogen three times. The solution was loaded to a filling loop (PFA capillary tubing: 1.6 mm OD, 1.0 mm ID), that had been sparged with N_2. By using a peristaltic pump, the solution was delivered to the flow reactor (UFlow) with a PFA coil (1.6 mm OD, 1 mm ID, 12.0 mL volume). The solution was pumped with a total flow rate of 1.2 mL/min flow rate (10 minutes of residence time). The crude reaction mixture was extracted with DCM (3 x 20 mL) and combined organic extracts were dried (anhydrous Na_2SO_4). The solvent was removed via rotary evaporation and the residue was purified via silica gel chromatography to give the corresponding products.

5.2 General procedure for 100-gram large scale synthesis:

TBADT (17 mmol, 5 mol%), $NiBr_2$·DME (34 mmol, 10 mol%), dtbbpy (34 mmol, 10 mol%) were dissolved in dry MeCN (3400 mL). The reaction mixture was stirred at ambient temperature for 1 hours. Subsequently, RAEs (340 mmol, 1.0 equiv.), DIPEA (0.5 equiv.) and corresponding aldehydes (3.0 equiv.) were added sequentially. The solution was backfilled with nitrogen three times and was loaded to a filling loop (PFA capillary tubing: 1.6 mm OD, 1.0 mm ID), that had been sparged with N_2. By using a peristaltic pump, the solution was delivered to the flow reactor (UFlow) with a PFA coil (1.6 mm OD, 1 mm ID, 24.0 mL volume). The solution was pumped with a total flow rate of 2.4 mL/min flow rate (10 minutes of residence time). The crude reaction mixture was concentrated under reduced pressure and divided into 6 parts and was purified via silica gel chromatography to give the yellow oil (45.2 g, 81%).

6. Mechanistic Investigation

6.1 Radical quenching experiment

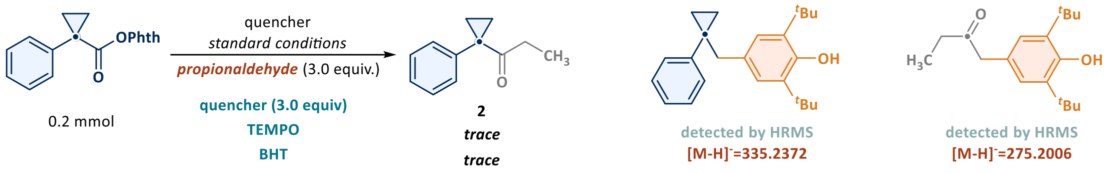

To an oven-dried vial equipped with a stirring bar were charged sequentially with TBADT (0.05 mmol, 5 mol%), $NiBr_2$·DME (0.1 mmol, 10 mol%), dtbbpy (0.1 mmol, 10 mol%), a Teflon-coated magnetic stir bar, and MeCN (10.0 mL). The reaction mixture was stirred at ambient temperature for 30 minutes. Subsequently, RAEs (1 mmol, 1.0 equiv.), DIPEA (0.5 equiv.) and corresponding aldehydes (3.0 equiv.) and a radical quencher (TEMPO or BHT, 3 mmol) were added sequentially. Subsequently, the vial was sealed with a rubber septum and the solution was backfilled with nitrogen three times. The solution was loaded to a filling loop (PFA capillary tubing: 1.6 mm OD, 1.0 mm ID), that had been sparged with N_2. By using a peristaltic pump, the solution was delivered to the flow reactor (UFlow) with a PFA coil (1.6 mm OD, 1 mm ID, 12.0 mL volume). The solution was pumped with a total flow rate of 1.2 mL/min flow rate (10 minutes of residence time). The crude reaction mixture was extracted with DCM (3 x 20 mL) and combined organic extracts were dried (anhydrous Na_2SO_4). The volatiles were removed under reduced pressure to obtain the crude product, and the residue was detected via HRMS. Evidently, the radical adducts of BHT were detected by HRMS (Fig. 4).

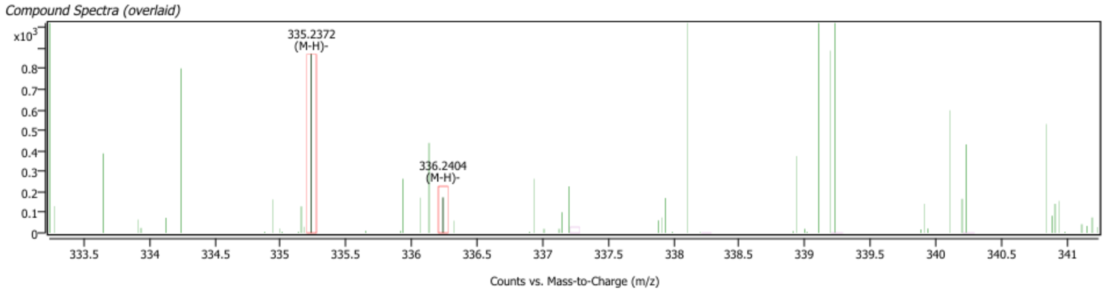

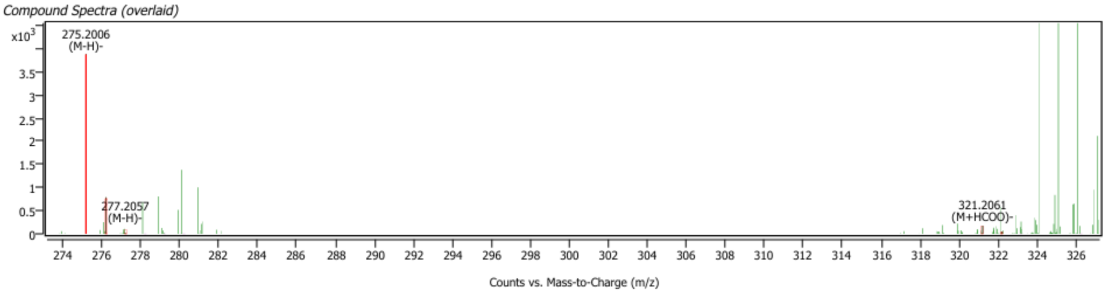

Supplementary Fig. 5 HRMS spectra of the adducts of BHT.

6.2 Radical clock experiment

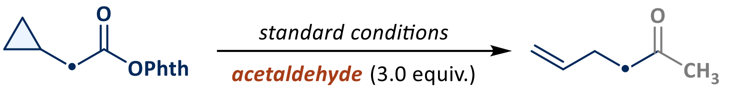

65, 44%
ring-opening product

To an oven-dried vial equipped with a stirring bar were charged sequentially with TBADT (0.05 mmol, 5 mol%), $NiBr_2$·DME (0.1 mmol, 10 mol%), dtbbpy (0.1 mmol, 10 mol%), a Teflon-coated magnetic stir bar, and MeCN (10.0 mL). The reaction mixture was stirred at ambient temperature for 30 minutes. Subsequently, RAEs (1 mmol, 1.0 equiv.), DIPEA (0.5 equiv.) and corresponding aldehyde (3.0 equiv.) were added sequentially. Subsequently, the vial was sealed with a rubber septum and the solution was backfilled with nitrogen three times. The solution was loaded to a filling loop (PFA capillary tubing: 1.6 mm OD, 1.0 mm ID), that had been sparged with N_2. By using a peristaltic pump, the solution was delivered to the flow reactor (UFlow) with a PFA coil (1.6 mm OD, 1 mm ID, 12.0 mL volume). The solution was pumped with a total flow rate of 1.2 mL/min flow rate (10 minutes of residence time). The crude reaction mixture was extracted with DCM (3 x 20 mL) and combined organic extracts were dried (anhydrous Na_2SO_4). The collected solution was evaporated under reduced pressure and the crude reaction mixture was purified via flash column chromatography on silica gel to give the corresponding product **65**. Spectral data are provided below.

6.3 Light on/off experiment

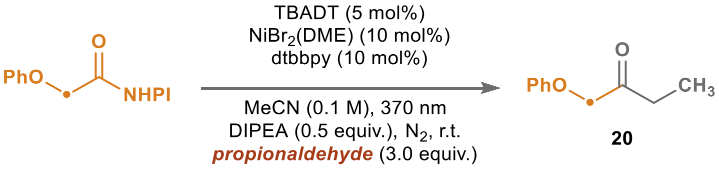

20

A 50 mL oven-dried Schlenk tube was charged sequentially with TBADT (0.05 mmol, 5 mol%), $NiBr_2$·DME (0.1 mmol, 10 mol%), dtbbpy (0.1 mmol, 10 mol%), a Teflon-coated magnetic stir bar, and MeCN (10.0 mL). The reaction mixture was stirred at ambient temperature for 30 minutes. Subsequently, RAEs (1 mmol, 1.0 equiv.), DIPEA (0.5 equiv.) and corresponding aldehydes (3.0 equiv.) were added sequentially. The resulting mixture was backfilled with nitrogen three times. After that, the reaction was placed under 40 W 370 nm LED, stirred and irradiated under nitrogen atmosphere. As the time period indicated in Fig. 5. At the end of each period, a small portion (200 μL) of the reacting solution was taken by a syringe, extracted with DCM, and the volatiles were removed under reduced pressure to obtain the crude sample, which was taken for ^{1}H NMR analysis.

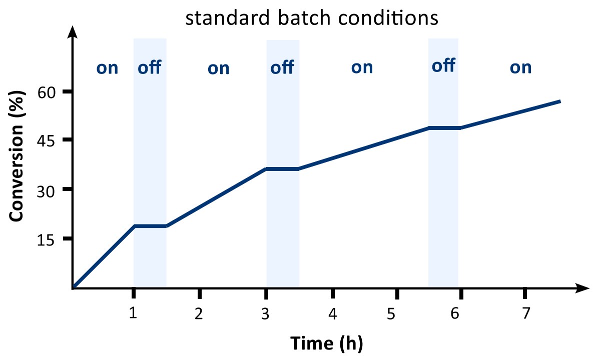

Supplementary Fig. 6 Light on/off experiments over time.

6.4 Kinetic isotope effect experiments

Preparation of benzaldehyde-alpha-d_1[4]:

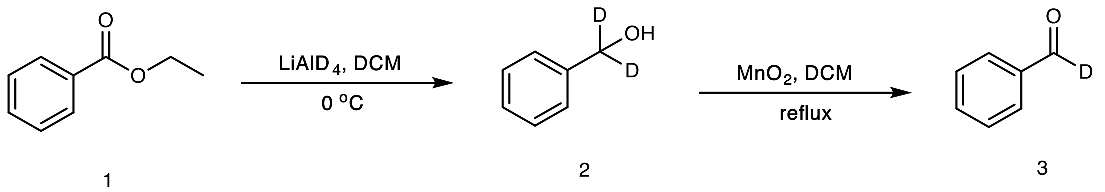

To a solution of the above ester **1** (1.00 g) in DCM (20 ml) were added $LiAlD_4$ (0.336 g, 8 mmol, 1.2 eq.) at 0 °C. After stirring for 30 min at same temperature, the reaction was quenched with saturated aqueous ammonium chloride. The aqueous phase was extracted twice with DCM (2×20 mL). The combined organic phase was washed with brine (30 mL), dried over Na_2SO_4, filtered and concentrated in vacuo, which was used for the next step without further purification. To a solution of the above mixture including **2** in DCM (25 ml), MnO_2 (13 g) was added, heat up to reflux temperature. After stirring for 2.5 h at the same temperature, the mixture was filtered and concentrated in vacuo. The residue was purified by flash column chromatography to afford **3** as a colorless oil.

General procedure for the kinetic isotope effect experiment:

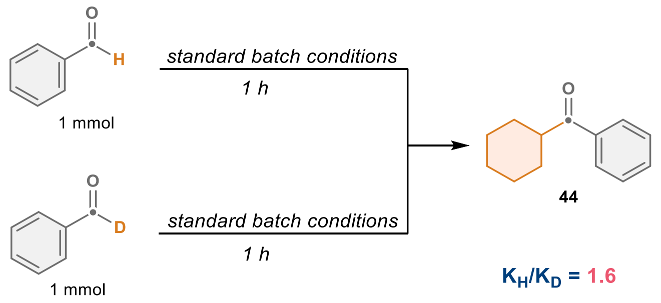

A 50 mL oven-dried Schlenk tube was charged sequentially with TBADT (0.05 mmol, 5 mol%), $NiBr_2 \cdot DME$ (0.1 mmol, 10 mol%), dtbbpy (0.1 mmol, 10 mol%), a Teflon-coated magnetic stir bar, and

MeCN (10.0 mL). The reaction mixture was stirred at ambient temperature for 30 minutes. Subsequently, RAEs (1 mmol, 1.0 equiv.), DIPEA (0.5 equiv.) and benzaldehyde or benzaldehyde-alpha-d_1 (3.0 equiv.) were added sequentially. The resulting mixture was backfilled with nitrogen three times. After that, the reaction was placed under 40 W 370 nm LEDs, stirred and irradiated under nitrogen atmosphere. After 1 hours, the two reaction mixtures were separately isolated by silica gel column chromatography in 18% and 11% yields, respectively.

The value of K_H/K_D (1.6) from two parallel reactions indicated that C-H bonds cleavage might be the kinetically rate-determining step in this reaction.

6.5 Cyclic Voltammetry Studies

Cyclic voltammograms were recorded in an electrolyte of n-Bu_4NPF_6 (0.1 M) in MeCN at room temperature using a glassy carbon disk working electrode (diameter, 3 mm), a Pt wire auxiliary electrode and an Ag/AgCl reference electrode. The solution was sparged with nitrogen for 10 minutes before data collection.

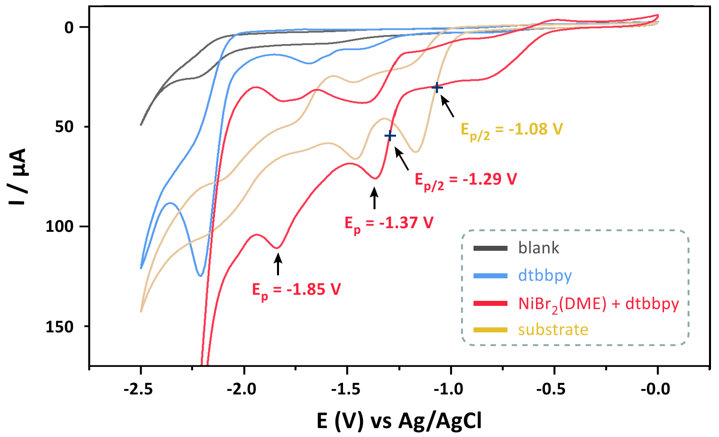

Supplementary Fig. 7 Cyclic voltammograms of $NiBr_2$(DME), dtbbpy and **19S RAE** in MeCN. The scan rate was 100 mV/s: MeCN (10 mL) containing $^{n}Bu_4NPF_6$ (0.1 M) (black curve); MeCN (10 mL) containing $^{n}Bu_4NPF_6$ (0.1 M), with dtbbpy (3 mM) (blue curve); MeCN (10 mL) containing $^{n}Bu_4NPF_6$ (0.1 M), with $NiBr_2$(DME) (3 mM) and dtbbpy (3 mM) (red curve); MeCN (10 mL) containing n-$^{n}Bu_4NPF_6$ (0.1 M), with **19S RAE** (3 mM) (yellow curve).

6.6 Density functional theory calculations

Method

All the quantum chemical calculation was conducted with Gaussian 16 version C01 package (for structural optimization and vibrational analysis).[5] The PBE0 hybrid function[6] all was applied for all calculations in combination with the D3BJ[7] dispersion correction and SMD implicit solvent model (with MeCN as solvent). For geometry optimization, the 6-311g(d,p) basis for C, N, O, F, Hand SDD basis for Ni set was used. The frequencies were computed analytically at the same level of theory as the geometry optimizations to identify the nature of all stationary points being either minimum (no imaginary frequency) or transition state (only one imaginary frequency) and also to obtain the Gibbs free energy correction at 298.15 K. The final Gibbs free energies were obtained by directly sum up of the single point energies and the free energy correction.

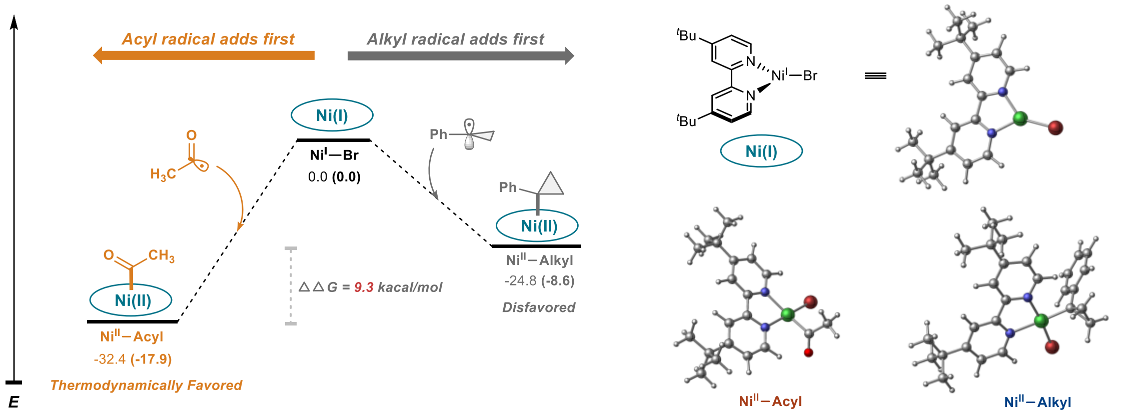

Supplementary Fig. 8 The 3D structures of involved substrates, catalysts, intermediates and transition states.

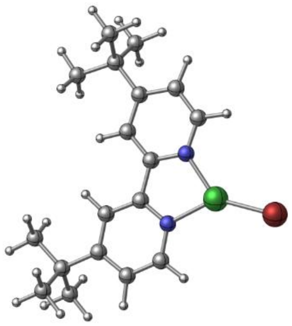

Ni^{I}-Br species

C	-0.42289100	0.73912900	-0.00000900
C	0.91231100	2.62647300	-0.00001500
C	-0.19077800	3.46105100	0.00000700
C	-1.48627700	2.91891200	0.00000500

C	-1.57529800	1.52759000	-0.00001100
C	-0.42317200	-0.73895700	-0.00003100
C	0.91116900	-2.62687500	-0.00007700
C	-0.19232000	-3.46092500	-0.00004200
C	-1.48760700	-2.91825000	-0.00001200
C	-1.57593800	-1.52689600	-0.00000100
H	1.92326300	3.01630300	-0.00001700
H	-0.03224900	4.53150600	0.00001900
H	-2.54040800	1.04441200	-0.00004200
H	1.92192500	-3.01720800	-0.00011800
H	-0.03420600	-4.53145200	-0.00002600
H	-2.54072500	-1.04306400	0.00003200
N	0.81175200	1.28849500	-0.00002300
N	0.81125400	-1.28884100	-0.00007100
C	-2.71216900	3.83884200	0.00000600
C	-2.67404100	4.72960900	-1.26307800
C	-2.67406800	4.72960500	1.26309500
H	-2.69321600	4.12046500	-2.17054500
H	-3.54233100	5.39427000	-1.27855400
H	-1.77736900	5.35207700	-1.29464100
H	-2.69328100	4.12045600	2.17055700
H	-1.77739800	5.35207300	1.29469500
H	-3.54235700	5.39426900	1.27853900
C	-2.71385900	-3.83775700	0.00001900
C	-2.67599000	-4.72851500	1.26309700
C	-2.67610100	-4.72847700	-1.26308500
H	-2.69502200	-4.11934500	2.17055200
H	-3.54447700	-5.39292000	1.27856200
H	-1.77948800	-5.35121200	1.29468400
H	-2.69526400	-4.11928300	-2.17052100
H	-1.77957800	-5.35113600	-1.29479700
H	-3.54456300	-5.39291600	-1.27846600
Ni	2.32654500	-0.00051800	-0.00001700
Br	4.61408900	-0.00095100	0.00003400
C	-4.03516300	-3.04951600	0.00008800
H	-4.13137600	-2.41855200	-0.88786500
H	-4.13126300	-2.41851300	0.88802700
C	-4.03385500	3.05118800	-0.00000200
H	-4.13031700	2.42019800	0.88790600
H	-4.13038300	2.42031600	-0.88798600
H	-4.87635700	-3.74663900	0.00015800
H	-4.87468800	3.74874600	0.00007000

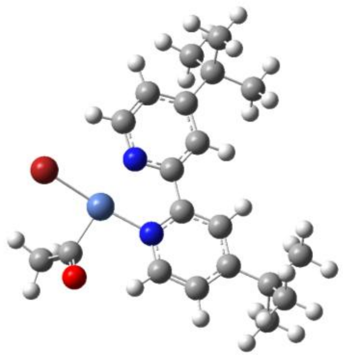

Ni^{II}-acyl species

C	0.39442900	0.91286100	0.01900400
C	-1.55597400	2.12099100	0.24540100
C	-0.87160500	3.32138200	0.14239100
C	0.51688600	3.33078700	-0.04781700
C	1.14178900	2.08511900	-0.10841000
C	0.97396900	-0.44520100	0.00108200
C	0.53413500	-2.71177200	0.07579700
C	1.88430100	-3.01275600	0.11091500
C	2.84286000	-1.99262200	0.07965600
C	2.34650400	-0.69105100	0.03481300
H	-2.63012500	2.07283400	0.37974000
H	-1.43400200	4.24380800	0.20602300
H	2.20740100	2.01716800	-0.26385300
H	-0.21922600	-3.48485900	0.12530400
H	2.17393000	-4.05376700	0.16803200
H	3.02285900	0.14939300	0.04618000
N	-0.94078700	0.93403400	0.18458800
N	0.06745100	-1.45299300	0.00005200
C	1.27538700	4.65592500	-0.18106200
C	1.06352400	5.48729600	1.10505100
C	0.72139400	5.43235100	-1.39775200
H	1.44106700	4.95385300	1.98132900
H	1.59779500	6.43821400	1.02697900
H	0.00847300	5.71059600	1.27585300
H	0.85286300	4.85911100	-2.31919700
H	-0.34210500	5.65412500	-1.28828200
H	1.25153400	6.38248600	-1.50730800
C	4.33823800	-2.32219700	0.11370900
C	4.68500300	-3.20190500	-1.10948600
C	4.65401900	-3.09544200	1.41472200
H	4.46374900	-2.67768100	-2.04288800
H	5.74993300	-3.44994800	-1.10075900
H	4.12422800	-4.13885300	-1.10821300
H	4.40743500	-2.49583500	2.29476800

H	4.09482100	-4.03121200	1.47758000
H	5.71918500	-3.33949900	1.45441700
Ni	-1.81793500	-0.91946300	0.02769500
Br	-4.01058800	-0.22607000	0.45835800
C	5.21572700	-1.05914900	0.07089400
H	5.03713200	-0.41094900	0.93331100
H	5.04863300	-0.47973700	-0.84142100
C	2.78638800	4.44761200	-0.38294800
H	3.00060300	3.88673000	-1.29693900
H	3.24047000	3.92321700	0.46249000
H	6.26965500	-1.34602400	0.08949400
H	3.28011700	5.41822000	-0.47046400
C	-2.48753500	-2.59732500	-0.47262900
O	-2.34405300	-3.60561900	0.18113100
C	-3.16393400	-2.63168600	-1.84003400
H	-3.25774700	-3.66458800	-2.18524700
H	-4.14768200	-2.16916100	-1.74070600
H	-2.59549600	-2.03189900	-2.55551800

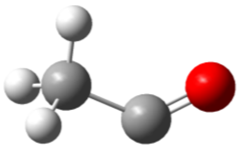

Acyl radical species

C	0.24903000	-0.43523700	0.00000300
O	1.25861100	0.17570500	0.00000600
C	-1.16863000	0.09831000	0.00003100
H	-1.17631500	1.19306700	0.00020200
H	-1.68727000	-0.28835000	-0.88023100
H	-1.68769900	-0.28879300	0.87977700

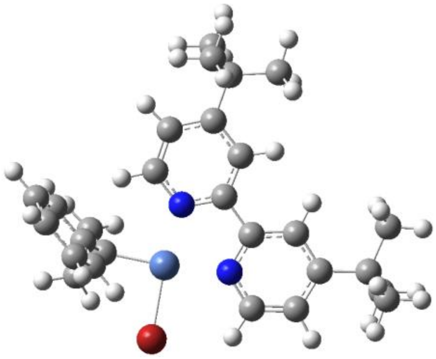

Ni^{I}-Alkyl speices

C	1.73803100	-0.17680300	-0.05409200
C	1.74018300	-2.47961500	0.00378700
C	3.11467700	-2.51431700	0.17160200
C	3.85258800	-1.32545800	0.23111800
C	3.12383600	-0.14294900	0.11172900
C	0.89846600	1.02699900	-0.19473200
C	-1.22203200	1.84065600	-0.57814000
C	-0.78686000	3.14907000	-0.46773700
C	0.55891200	3.43055700	-0.20678200
C	1.39609200	2.32479300	-0.07352300
H	1.13832600	-3.37869400	-0.04034000
H	3.59804400	-3.47899800	0.25442700
H	3.62739500	0.81057000	0.13932400
H	-2.25531500	1.61579300	-0.77610000
H	-1.51593000	3.94031900	-0.58225900
H	2.44515700	2.46072700	0.13691900
N	1.05255400	-1.33469800	-0.10695600
N	-0.40936900	0.78059500	-0.44554800
C	5.37343100	-1.35888100	0.41492200
C	6.00491100	-2.13702200	-0.76226800
C	5.70200200	-2.07450300	1.74540400
H	5.77846700	-1.65335600	-1.71608700
H	7.09166700	-2.17239000	-0.64613200
H	5.64086600	-3.16525600	-0.81150700
H	5.25792700	-1.54599100	2.59298600
H	5.33061700	-3.10118700	1.75716900
H	6.78496500	-2.10899700	1.89296100
C	1.04590300	4.87651700	-0.07534600
C	0.29114400	5.55488300	1.09109900
C	0.74829600	5.62856100	-1.39290000
H	0.47998200	5.03423200	2.03344000
H	0.62491200	6.59058300	1.19920000
H	-0.78789100	5.56710900	0.92433500
H	1.26593600	5.16135600	-2.23480000
H	-0.31962500	5.64328400	-1.62016100
H	1.08753000	6.66518200	-1.31555300
Ni	-0.95688400	-1.11757000	-0.44250200
Br	-1.42969900	-3.39317500	-0.09817400
C	2.55635900	4.96112400	0.20459700
H	3.14426600	4.51469800	-0.60229200
H	2.82338600	4.46925200	1.14403700
C	5.98920300	0.05054000	0.45433200
H	5.59694900	0.64176500	1.28650200
H	5.81204600	0.59730900	-0.47603200

H	2.85431200	6.00891600	0.28693000
H	7.07063600	-0.02722600	0.58821600
C	-4.37586400	-0.40933200	2.50900600
C	-3.59235500	-0.84875600	1.44768400
C	-3.65866900	-0.23312600	0.18478000
C	-4.55065000	0.84286500	0.04299100
C	-5.33692900	1.28550700	1.10666700
C	-5.25293900	0.66384200	2.34848500
H	-4.30142200	-0.90969500	3.46893100
H	-2.91799000	-1.68510000	1.57947000
H	-4.64499900	1.34346700	-0.91426300
H	-6.01727000	2.11825400	0.95952700
H	-5.86189900	1.00667500	3.17775600
C	-2.79846400	-0.73568200	-0.93500300
C	-2.96730700	-0.19093200	-2.34769900
C	-3.46812300	-1.56884100	-2.00602200
H	-3.69649900	0.58411900	-2.56609400
H	-2.07010500	-0.12071600	-2.95477300
H	-4.54189200	-1.70546700	-1.92195800
H	-2.93569500	-2.42693700	-2.39384300

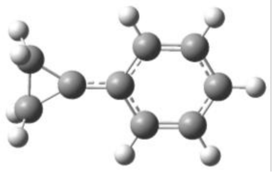

Alkyl radical species

C	1.97312600	1.21069600	-0.00001400
C	0.59001400	1.21990100	0.00005100
C	-0.14598300	0.00009300	0.00007000
C	0.58986100	-1.21980800	0.00003000
C	1.97297100	-1.21077200	-0.00002700
C	2.67749600	-0.00008100	-0.00004900
H	2.51662100	2.14936600	-0.00004100
H	0.04419500	2.15666100	0.00008100
H	0.04392000	-2.15649700	0.00004200
H	2.51635300	-2.14950800	-0.00005300
H	3.76120700	-0.00015500	-0.00009500
C	-1.53802400	0.00014500	0.00016400
C	-2.77093700	-0.77366300	-0.00005500
C	-2.77116900	0.77357200	-0.00009200
H	-3.08637500	-1.27997900	-0.91133000
H	-3.08656400	-1.27976700	0.91129700

H	-3.08654200	1.27966900	-0.91151300
H	-3.08694900	1.27972200	0.91114400

7. Analytic Data of Products

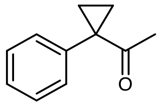

1-(1-phenylcyclopropyl)ethan-1-one (1). Prepared as described above. Purified by silica column chromatography using petroleum ether/EtOAc to give 51% yield (81.7 mg) of the product as a colorless oil. ^{1}H NMR and ^{13}C NMR data match previously reported data. (Known compound: *Adv. Synth. Catal.*, **2018**, *360*, 4459).

R$_f$ value: 0.53 (PE:EA = 10:1).

^{1}H NMR (400 MHz, Chloroform-*d*) δ 7.41 – 7.24 (m, 5H), 2.01 (s, 3H), 1.60 (q, *J* = 3.6 Hz, 2H), 1.18 (q, *J* = 3.6 Hz, 2H).

^{13}C NMR (101 MHz, Chloroform-*d*) δ 208.9, 141.2, 130.8, 128.7, 127.5, 37.6, 29.5, 18.7.

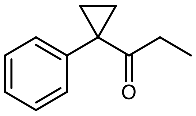

1-(1-phenylcyclopropyl)propan-1-one (2). Prepared as described above. Purified by silica column chromatography using petroleum ether/EtOAc to give 57% yield (99.2 mg) of the product as a colorless oil.

R$_f$ value: 0.5 (PE:EA = 10:1).

^{1}H NMR (400 MHz, Chloroform-*d*) δ 7.39 – 7.27 (m, 5H), 2.30 (q, *J* = 7.2 Hz, 5H), 1.59 (q, *J* = 3.5 Hz, 2H), 1.14 (q, *J* = 3.5 Hz, 2H), 0.92 (t, *J* = 7.2 Hz, 3H).

^{13}C NMR (101 MHz, Chloroform-*d*) δ 211.4, 141.1, 131.0, 128.6, 127.4, 37.1, 35.2, 18.7, 8.0.

HRMS: Calculated for $C_{12}H_{14}O$, $[M+H]^+$ 175.1118; found 175.1119.

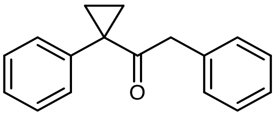

2-phenyl-1-(1-phenylcyclopropyl)ethan-1-one (3). Prepared as described above. Purified by silica column chromatography using petroleum ether/EtOAc to give 54% yield (127.5 mg) of the product as a colorless oil. ^{1}H NMR and ^{13}C NMR data match previously reported data. (Known compound: *Org. Lett.*, **2006**, *8*, 1709-1712).

Rf value: 0.6 (PE:EA = 10:1).

^{1}H NMR (400 MHz, Chloroform-*d*) δ 7.38 – 7.30 (m, 5H), 7.28 – 7.17 (m, 3H), 6.97 – 6.87 (m, 2H), 3.64 (s, 2H), 1.66 (q, *J* = 3.6 Hz, 2H), 1.20 (q, *J* = 3.6 Hz, 2H).

^{13}C NMR (101 MHz, Chloroform-*d*) δ 208.1, 140.6, 134.4, 131.2, 129.5, 128.7, 128.3, 127.6, 126.6, 48.1, 37.4, 19.2.

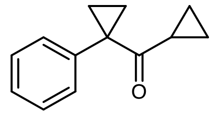

cyclopropyl(1-phenylcyclopropyl)methanone (4). Prepared as described above. Purified by silica column chromatography using petroleum ether/EtOAc to give 52% yield (96.7 mg) of the product as a colorless oil.

Rf value: 0.5 (PE:EA = 10:1).

^{1}H NMR (400 MHz, Chloroform-*d*) δ 7.46 – 7.40 (m, 2H), 7.40 – 7.33 (m, 2H), 7.33 – 7.27 (m, 1H), 1.85 – 1.76 (m, 1H), 1.61 (q, *J* = 3.6 Hz, 2H), 1.21 (q, *J* = 3.6 Hz, 2H), 1.01 – 0.96 (m, 2H), 0.74 – 0.68 (m, 2H).

^{13}C NMR (101 MHz, Chloroform-*d*) δ 210.4, 141.1, 130.9, 128.6, 127.3, 37.5, 18.6, 18.6, 11.8.

HRMS: Calculated for $C_{13}H_{14}O$, $[M+H]^+$ 187.1114; found 187.1115.

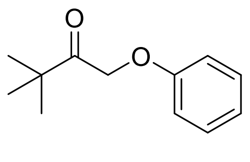

3,3-dimethyl-1-phenoxybutan-2-one (5). Prepared as described above. Purified by silica column chromatography using petroleum ether/EtOAc to give 79% yield (151.8 mg) of the product as a yellow oil. ^{1}H NMR and ^{13}C NMR data match previously reported data. (Known compound: *Org. Lett.*, **2020**, *22*, 6526-6531).

Rf value: 0.7 (PE:EA = 10:1).

^{1}H NMR (400 MHz, Chloroform-*d*) δ 7.34 – 7.26 (m, 2H), 7.00 (td, *J* = 7.4, 1.1 Hz, 1H), 6.91 (dt, *J* = 7.9, 1.0 Hz, 2H), 4.89 (s, 2H), 1.28 (s, 9H).

^{13}C NMR (101 MHz, Chloroform-*d*) δ 209.6, 158.1, 129.5, 121.5, 114.7, 68.9, 43.2, 26.4.

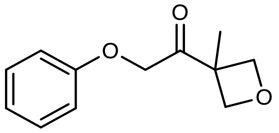

1-(3-methyloxetan-3-yl)-2-phenoxyethan-1-one (6). Prepared as described above. Purified by silica column chromatography using petroleum ether/EtOAc to give 76% yield (156.6 mg) of the product as a yellow oil.

R_f value: 0.43 (PE:EA = 10:1).

^{1}H NMR (400 MHz, Chloroform-*d*) δ 7.37 – 7.30 (m, 2H), 7.08 – 7.01 (m, 1H), 6.92 – 6.85 (m, 2H), 5.04 (d, *J* = 6.3 Hz, 2H), 4.72 (s, 2H), 4.48 (d, *J* = 6.3 Hz, 2H), 1.74 (s, 3H).

^{13}C NMR (101 MHz, Chloroform-*d*) δ 207.3, 157.4, 129.8, 122.1, 114.4, 78.8, 71.1, 50. 0, 21.6.

HRMS: Calculated for $C_{12}H_{14}O_3$, $[M+H]^+$ 207.1016; found 207.1009.

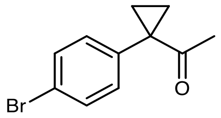

1-(1-(4-bromophenyl)cyclopropyl)ethan-1-one (7). Prepared as described above. Purified by silica column chromatography using petroleum ether/EtOAc to give 59% yield (140.3 mg) of the product as a yellow oil. ^{1}H NMR and ^{13}C NMR data match previously reported data. (Known compound: *J. Org. Chem.*, **2018**, *83*, 4905-4921).

R_f value: 0.5 (PE:EA = 10:1).

^{1}H NMR (400 MHz, Chloroform-*d*) δ 7.49 – 7.44 (m, 2H), 7.26 – 7.21 (m, 2H), 1.99 (s, 3H), 1.60 (q, *J* = 3.7 Hz, 2H), 1.14 (q, *J* = 3.7 Hz, 2H).

^{13}C NMR (101 MHz, Chloroform-*d*) δ 208.0, 140.2, 132.5, 131.8, 121.5, 37.1, 29.3, 18.7.

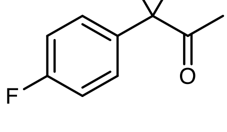

1-(1-(4-fluorophenyl)cyclopropyl)ethan-1-one (8). Prepared as described above. Purified by silica column chromatography using petroleum ether/EtOAc to give 63% yield (112.2 mg) of the product as a yellow oil. ^{1}H NMR and ^{13}C NMR data match previously reported data. (Known compound: *Eur. J. Org. Chem.*, **2020**, 1778-1781).

R$_f$ value: 0.53 (PE:EA = 10:1).

^{1}H NMR (400 MHz, Chloroform-*d*) δ 7.36 – 7.29 (m, 2H), 7.07 – 6.99 (m, 2H), 1.99 (s, 3H), 1.60 (q, *J* = 3.6 Hz, 2H), 1.14 (q, *J* = 3.7 Hz, 2H).

^{13}C NMR (101 MHz, Chloroform-*d*) δ 208.6, 162.0 (d, *J* = 246.4 Hz), 137.0 (d, *J* = 3.3 Hz), 132.4 (d, *J* = 8.1 Hz), 115.5 (d, *J* = 21.3 Hz), 36.9, 29.3, 18.9.

^{19}F NMR (376 MHz, Chloroform-*d*) δ -114.63.

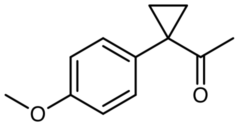

1-(1-(4-methoxyphenyl)cyclopropyl)ethan-1-one (9). Prepared as described above. Purified by silica column chromatography using petroleum ether/EtOAc to give 55% yield (104.5mg) of the product as a colorless oil.

R$_f$ value: 0.45 (PE:EA = 10:1).

^{1}H NMR (400 MHz, Chloroform-*d*) δ 7.35 – 7.24 (m, 2H), 6.95 – 6.84 (m, 2H), 3.83 (s, 3H), 2.02 (s, 3H), 1.59 (q, *J* = 3.5 Hz, 2H), 1.15 (q, *J* = 3.5 Hz, 2H).

^{13}C NMR (101 MHz, Chloroform-*d*) δ 209.6, 158.9, 133.3, 131.8, 114.0, 55.3, 36.8, 29.5, 19.0.

HRMS: Calculated for $C_{12}H_{14}O_2$, $[M+H]^+$ 191.1067; found 191.1066.

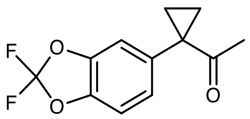

1-(1-(2,2-difluorobenzo[*d*][1,3]dioxol-5-yl)cyclopropyl)ethan-1-one (10). Prepared as described above. Purified by silica column chromatography using petroleum ether/EtOAc to give 59% yield (141.6 mg) of the product as a yellow oil. ^{1}H NMR and ^{13}C NMR data match previously reported data. (Known compound: *J. Org. Chem.*, **2018**, 83, 4905-4921).

R$_f$ value: 0.5 (PE:EA = 10:1).

^{1}H NMR (400 MHz, Chloroform-*d*) δ 7.14 – 7.08 (m, 2H), 7.07 – 7.02 (m, 1H), 2.03 (s, 3H), 1.64 (q, J = 3.8 Hz, 2H), 1.18 (q, J = 3.8 Hz, 2H).

^{13}C NMR (101 MHz, Chloroform-*d*) δ 207.8, 143.8, 143.0, 137.3, 131.7 (t, J = 255.5 Hz), 126.0, 112.0, 109.4, 37.5, 28.9, 18.9.

^{19}F NMR (376 MHz, Chloroform-*d*) δ -49.89.

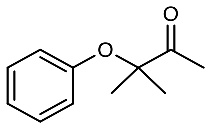

3-methyl-3-phenoxybutan-2-one (11). Prepared as described above. Purified by silica column chromatography using petroleum ether/EtOAc to give 45% yield (80.1 mg) of the product as a colorless oil. ^{1}H NMR and ^{13}C NMR data match previously reported data. (Known compound: *Chem. Lett.*, **2006**, *35*, 612-613).

R_f value: 0.39 (PE:EA = 10:1).

^{1}H NMR (400 MHz, Chloroform-*d*) δ 7.31 – 7.22 (m, 2H), 7.05 – 6.97 (m, 1H), 6.83 – 6.77 (m, 2H), 2.30 (s, 3H), 1.51 (s, 6H).

^{13}C NMR (101 MHz, Chloroform-*d*) δ 212.3, 155.3, 129.4, 122.0, 84.0, 24.4, 23.9.

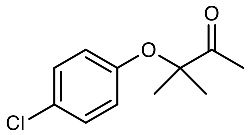

3-(4-chlorophenoxy)-3-methylbutan-2-one (12). Prepared as described above. Purified by silica column chromatography using petroleum ether/EtOAc to give 49% yield (103.9 mg) of the product as a yellow oil. ^{1}H NMR and ^{13}C NMR data match previously reported data. (Known compound: *Nature*, **2024**, *631*, 556-562).

R_f value: 0.43 (PE:EA = 10:1).

^{1}H NMR (400 MHz, Chloroform-*d*) δ 7.27 – 7.17 (m, 2H), 6.79 – 6.67 (m, 2H), 2.27 (s, 3H), 1.48 (s, 6H).

^{13}C NMR (101 MHz, Chloroform-*d*) δ 211.7, 153.9, 129.4, 127.1, 119.8, 84.4, 24.3, 23.8.

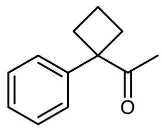

1-(1-phenylcyclobutyl)ethan-1-one (13). Prepared as described above. Purified by silica column chromatography using petroleum ether/EtOAc to give 45% yield (78.3 mg) of the product as a colorless oil. ^{1}H NMR and ^{13}C NMR data match previously reported data. (Known compound: *Synth. Commun.*, **2010**, 3237-3245).

R$_f$ value: 0.5 (PE:EA = 10:1).

^{1}H NMR (400 MHz, Chloroform-*d*) δ 7.42 – 7.34 (m, 2H), 7.30 – 7.23 (m, 3H), 2.82 – 2.72 (m, 2H), 2.50 – 2.39 (m, 2H), 1.95 (s, 3H), 1.94 – 1.82 (m, 2H).

^{13}C NMR (101 MHz, Chloroform-*d*) δ 208.6, 143.2, 128.7, 126.7, 126.2, 59.3, 30.5, 24.4, 15.9.

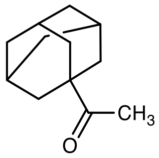

1-(adamantan-1-yl)ethan-1-one (14). Prepared as described above. Purified by silica column chromatography using petroleum ether/EtOAc to give 39% yield (69.4 mg) of the product as a white solid. ^{1}H NMR and ^{13}C NMR data match previously reported data. (Known compound: *J. Org. Chem.*, **2023**, *88*, 9130-9135).

Mp: 54.4-56.9 °C.

R$_f$ value: 0.75 (PE:EA = 10:1).

^{1}H NMR (400 MHz, Chloroform-*d*) δ 2.08 (s, 3H), 2.06 – 2.00 (m, 3H), 1.82 – 1.78 (m, 6H), 1.77 – 1.63 (m, 6H).

^{13}C NMR (101 MHz, Chloroform-*d*) δ 214.1, 46.5, 38.3, 36.6, 28.0, 24.3.

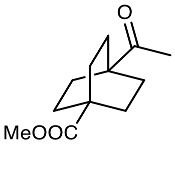

methyl 4-acetylbicyclo[2.2.2]octane-1-carboxylate (15). Prepared as described above. Purified by silica column chromatography using petroleum ether/EtOAc to give 34% yield (71.4 mg) of the product as a colorless oil. ^{1}H NMR and ^{13}C NMR data match previously reported data. (Known compound:

WO2020168143).

Rf value: 0.18 (PE:EA = 10:1).

^{1}H NMR (400 MHz, Chloroform-*d*) δ 3.64 (s, 3H), 2.07 (s, 3H), 1.86 – 1.66 (m, 12H).

^{13}C NMR (101 MHz, Chloroform-*d*) δ 213.2, 177.8, 51.7, 44.7, 38.9, 27.8, 27.1, 25.3.

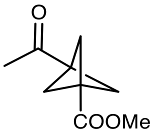

methyl 3-acetylbicyclo[1.1.1]pentane-1-carboxylate (16). Prepared as described above. Purified by silica column chromatography using petroleum ether/EtOAc to give 31% yield (52.1 mg) of the product as a colorless oil. ^{1}H NMR and ^{13}C NMR data match previously reported data. (Known compound: *Green Chem.*, **2025**, *27*, 256-263).

Rf value: 0.2 (PE:EA = 10:1).

^{1}H NMR (400 MHz, Chloroform-*d*) δ 3.67 (s, 3H), 2.26 (s, 6H), 2.12 (s, 3H).

^{13}C NMR (101 MHz, Chloroform-*d*) δ 205.4, 169.9, 52.4, 51.9, 44.0, 37.0, 26.2.

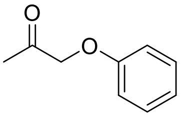

1-phenoxypropan-2-one (19). Prepared as described above. Purified by silica column chromatography using petroleum ether/EtOAc to give 82% yield (123.1 mg) of the product as a yellow oil. ^{1}H NMR and ^{13}C NMR data match previously reported data. (Known compound: *ACS Catal.*, **2011**, *1*, 116-119).

Rf value: 0.61 (PE:EA = 10:1).

^{1}H NMR (400 MHz, Chloroform-*d*) δ 7.37 – 7.30 (m, 2H), 7.07 – 6.99 (m, 1H), 6.94 – 6.88 (m, 2H), 4.56 (s, 2H), 2.30 (s, 3H).

^{13}C NMR (101 MHz, Chloroform-*d*) δ 205.9, 157.8, 129.7, 121.8, 114.5, 73.0, 26.6.

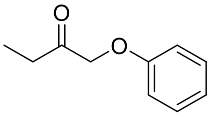

1-phenoxybutan-2-one (20). Prepared as described above. Purified by silica column chromatography using petroleum ether/EtOAc to give 84% yield (137.8 mg) of the product as a yellow oil. ^{1}H NMR and

^{13}C NMR data match previously reported data. (Known compound: *Tetrahedron*, **2010**, *66*, 5675-5686).

R$_f$ value: 0.7 (PE:EA = 10:1).

^{1}H NMR (400 MHz, Chloroform-*d*) δ 7.34 – 7.27 (m, 2H), 7.00 (tt, *J* = 7.3, 1.0 Hz, 1H), 6.91 – 6.85 (m, 2H), 4.55 (s, 2H), 2.63 (q, *J* = 7.3 Hz, 2H), 1.11 (t, *J* = 7.3 Hz, 3H).

^{13}C NMR (101 MHz, Chloroform-*d*) δ 208.5, 157.8, 129.7, 121.7, 114.5, 72.6, 32.4, 7.1.

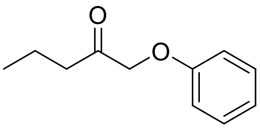

1-phenoxypentan-2-one (21). Prepared as described above. Purified by silica column chromatography using petroleum ether/EtOAc to give 75% yield (133.6 mg) of the product as a yellow oil. ^{1}H NMR and ^{13}C NMR data match previously reported data. (Known compound: *Angew. Chem. Int. Ed.*, **2020**, *59*, 14265).

R$_f$ value: 0.73 (PE:EA = 10:1).

^{1}H NMR (400 MHz, Chloroform-*d*) δ 7.36 – 7.30 (m, 2H), 7.09 – 6.95 (m, 1H), 6.94 – 6.79 (m, 2H), 4.56 (s, 2H), 2.60 (t, *J* = 7.3 Hz, 2H), 1.75 – 1.62 (m, 2H), 0.97 (t, *J* = 7.4 Hz, 3H).

^{13}C NMR (101 MHz, Chloroform-*d*) δ 207.9, 157.8, 129.7, 121.7, 114.5, 72.8, 40.9, 16.6, 13.7.

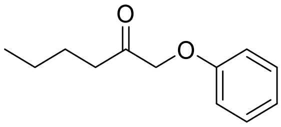

1-phenoxyhexan-2-one (22). Prepared as described above. Purified by silica column chromatography using petroleum ether/EtOAc to give 77% yield (147.8 mg) of the product as a yellow oil. ^{1}H NMR and ^{13}C NMR data match previously reported data. (Known compound: WO2005044008).

R$_f$ value: 0.73 (PE:EA = 10:1).

^{1}H NMR (400 MHz, Chloroform-*d*) δ 7.34 – 7.27 (m, 2H), 7.04 – 6.96 (m, 1H), 6.92 – 6.85 (m, 2H), 4.54 (s, 2H), 2.59 (t, *J* = 7.4 Hz, 2H), 1.66 – 1.56 (m, 2H), 1.40 – 1.29 (m, 2H), 0.91 (t, *J* = 7.4 Hz, 3H).

^{13}C NMR (101 MHz, Chloroform-*d*) δ 208.1, 157.8, 129.7, 121.7, 114.5, 72.8, 38.8, 25.3, 22.3, 13.9.

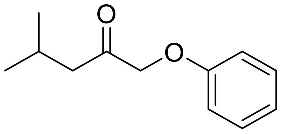

4-methyl-1-phenoxypentan-2-one (23). Prepared as described above. Purified by silica column chromatography using petroleum ether/EtOAc to give 77% yield (147.9 mg) of the product as a yellow oil. ^{1}H NMR and ^{13}C NMR data match previously reported data. (Known compound: *Tetrahedron*, **2001**, *57*, 5543-5549).

R$_f$ value: 0.75 (PE:EA = 10:1).

^{1}H NMR (400 MHz, Chloroform-*d*) δ 7.33 – 7.27 (m, 2H), 7.02 – 6.97 (m, 1H), 6.91 – 6.86 (m, 2H), 4.52 (s, 2H), 2.47 (d, *J* = 6.9 Hz, 2H), 2.29 – 2.16 (m, 1H), 0.95 (d, *J* = 6.7 Hz, 6H).

^{13}C NMR (101 MHz, Chloroform-*d*) δ 207.4, 157.8, 129.7, 121.7, 114.6, 73.1, 47.8, 24.1, 22.6.

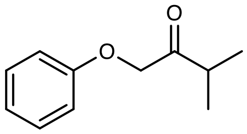

3-methyl-1-phenoxybutan-2-one (24). Prepared as described above. Purified by silica column chromatography using petroleum ether/EtOAc to give 84% yield (149.5 mg) of the product as a yellow oil. ^{1}H NMR and ^{13}C NMR data match previously reported data. (Known compound: *Tetrahedron*, **2010**, *66*, 5675-5686).

R$_f$ value: 0.75 (PE:EA = 10:1).

^{1}H NMR (400 MHz, Chloroform-*d*) δ 7.40 – 7.18 (m, 2H), 7.05 – 6.99 (m, 1H), 6.94 – 6.88 (m, 2H), 4.66 (s, 2H), 3.16 – 2.87 (m, 1H), 1.19 (d, *J* = 6.9 Hz, 6H).

^{13}C NMR (101 MHz, Chloroform-*d*) δ 210.9, 157.9, 129.7, 121.7, 114.6, 71.4, 37.1, 17.9.

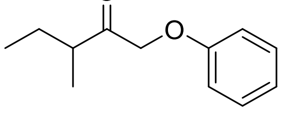

3-methyl-1-phenoxypentan-2-one (25). Prepared as described above. Purified by silica column chromatography using petroleum ether/EtOAc to give 81% yield (155.5 mg) of the product as a yellow oil.

R$_f$ value: 0.73 (PE:EA = 10:1).

¹H NMR (400 MHz, Chloroform-*d*) δ 7.33 – 7.27 (m, 2H), 7.04 – 6.96 (m, 1H), 6.93 – 6.85 (m, 2H), 4.62 (s, 2H), 2.86 – 2.74 (m, 1H), 1.85 – 1.69 (m, 1H), 1.52 – 1.38 (m, 1H), 1.13 (d, *J* = 7.0 Hz, 3H), 0.91 (t, *J* = 7.5 Hz, 3H).

¹³C NMR (101 MHz, Chloroform-*d*) δ 210.8, 157.9, 129.7, 121.7, 114.6, 72.0, 43.8, 25.6, 15.6, 11.6.

HRMS: Calculated for $C_{12}H_{16}O_2$, $[M+Na]^+$ 215.1051; found 215.1057.

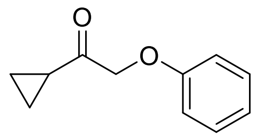

1-cyclopropyl-2-phenoxyethan-1-one (26). Prepared as described above. Purified by silica column chromatography using petroleum ether/EtOAc to give 73% yield (128.5 mg) of the product as a yellow oil. ¹H NMR and ¹³C NMR data match previously reported data. (Known compound: *Angew. Chem. Int. Ed.*, **2020**, *59*, 14265).

R_f value: 0.73 (PE:EA = 10:1).

¹H NMR (400 MHz, Chloroform-*d*) δ 7.34 – 7.27 (m, 2H), 7.03 – 6.97 (m, 1H), 6.95 – 6.87 (m, 2H), 4.68 (s, 2H), 2.31 (tt, *J* = 7.8, 4.5 Hz, 1H), 1.18 – 1.12 (m, 2H), 1.01 – 0.95 (m, 2H).

¹³C NMR (101 MHz, Chloroform-*d*) δ 207.7, 158.0, 129.7, 121.6, 114.6, 73.1, 17.1, 12.0.

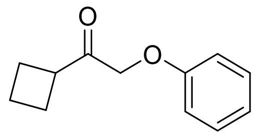

1-cyclobutyl-2-phenoxyethan-1-one (27). Prepared as described above. Purified by silica column chromatography using petroleum ether/EtOAc to give 79% yield (150.1 mg) of the product as a yellow oil. ¹H NMR and ¹³C NMR data match previously reported data. (Known compound: *Angew. Chem. Int. Ed.*, **2020**, *59*, 14265).

R_f value: 0.7 (PE:EA = 10:1).

¹H NMR (400 MHz, Chloroform-*d*) δ 7.39 – 7.25 (m, 2H), 7.04 – 6.97 (m, 1H), 6.93 – 6.86 (m, 2H), 4.59 (s, 2H), 3.66 – 3.51 (m, 1H), 2.40 – 2.28 (m, 2H), 2.26 – 2.14 (m, 2H), 2.11 – 1.97 (m, 1H), 1.96 – 1.84 (m, 1H).

¹³C NMR (101 MHz, Chloroform-*d*) δ 208.4, 157.9, 129.7, 121.6, 114.5, 71.4, 42.3, 24.2, 18.2.

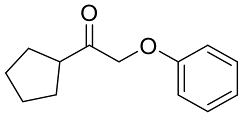

1-cyclopentyl-2-phenoxyethan-1-one (28). Prepared as described above. Purified by silica column chromatography using petroleum ether/EtOAc to give 78% yield (159.2 mg) of the product as a yellow oil.

R_f value: 0.75 (PE:EA = 10:1).

^{1}H NMR (400 MHz, Chloroform-*d*) δ 7.35 – 7.26 (m, 2H), 7.04 – 6.98 (m, 1H), 6.93 – 6.81 (m, 2H), 4.65 (s, 2H), 3.28 – 3.10 (m, 1H), 1.95 – 1.77 (m, 4H), 1.76 – 1.59 (m, 4H).

^{13}C NMR (101 MHz, Chloroform-*d*) δ 209.7, 158.0, 129.7, 121.6, 114.6, 72.1, 47.5, 28.8, 26.1.

HRMS: Calculated for $C_{13}H_{16}O_2$, $[M+H]^+$ 205.1223; found 205.1217.

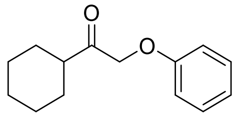

1-cyclohexyl-2-phenoxyethan-1-one (29). Prepared as described above. Purified by silica column chromatography using petroleum ether/EtOAc to give 82% yield (178.8 mg) of the product as a yellow oil. ^{1}H NMR and ^{13}C NMR data match previously reported data. (Known compound: *J. Am. Chem. Soc.*, **2024**, *146*, 22829-22839).

R_f value: 0.73 (PE:EA = 10:1).

^{1}H NMR (400 MHz, Chloroform-*d*) δ 7.34 – 7.25 (m, 2H), 6.99 (t, *J* = 7.4 Hz, 1H), 6.88 (d, *J* = 8.1 Hz, 2H), 4.63 (s, 2H), 2.69 (tt, *J* = 11.3, 3.4 Hz, 1H), 1.91 – 1.84 (m, 2H), 1.83 – 1.77 (m, 2H), 1.72 – 1.65 (m, 1H), 1.48 – 1.37 (m, 2H), 1.36 – 1.28 (m, 2H), 1.28 – 1.24 (m, 1H).

^{13}C NMR (101 MHz, Chloroform-*d*) δ 209.9, 158.0, 129.6, 121.6, 114.6, 71.5, 47.0, 28.2, 25.8, 25.6.

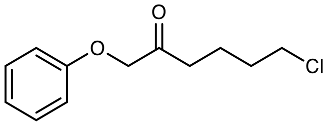

6-chloro-1-phenoxyhexan-2-one (30). Prepared as described above. Purified by silica column chromatography using petroleum ether/EtOAc to give 76% yield (171.8 mg) of the product as a yellow oil. ^{1}H NMR and ^{13}C NMR data match previously reported data. (Known compound: *Tetrahedron*, **2001**,

57, 5543-5549).

Rf value: 0.7 (PE:EA = 10:1).

^{1}H NMR (400 MHz, Chloroform-*d*) δ 7.36 – 7.30 (m, 2H), 7.05 – 6.99 (m, 1H), 6.94 – 6.88 (m, 2H), 4.56 (s, 2H), 3.61 – 3.50 (m, 2H), 2.72 – 2.64 (m, 2H), 1.92 – 1.75 (m, 4H).

^{13}C NMR (101 MHz, Chloroform-*d*) δ 207.5, 157.7, 129.8, 121.8, 114.5, 72.8, 44.6, 38.1, 31.8, 20.4.

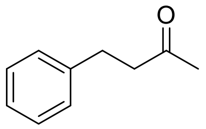

4-phenylbutan-2-one (31). Prepared as described above. Purified by silica column chromatography using petroleum ether/EtOAc to give 62% yield (91.7 mg) of the product as a colorless oil. ^{1}H NMR and ^{13}C NMR data match previously reported data. (Known compound: *Chem. Eur. J.,* **2016***, 22*, 4738-4742).

Rf value: 0.48 (PE:EA = 10:1).

^{1}H NMR (400 MHz, Chloroform-*d*) δ 7.29 (m, 2H), 7.24 – 7.16 (m, 3H), 2.90 (t, *J* = 7.6 Hz, 2H), 2.76 (t, *J* = 7.9 Hz, 2H), 2.14 (s, 3H).

^{13}C NMR (101 MHz, Chloroform-*d*) δ 208.0, 141.0, 128.5, 128.3, 126.1, 45.2, 30.1, 29.8.

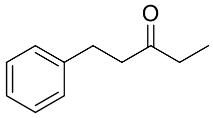

1-phenylpentan-3-one (32). Prepared as described above. Purified by silica column chromatography using petroleum ether/EtOAc to give 69% yield (111.8 mg) of the product as a colorless oil. ^{1}H NMR and ^{13}C NMR data match previously reported data. (Known compound: *Adv. Synth. Catal.*, **2012**, *354*, 341-346).

Rf value: 0.5 (PE: EA = 10:1).

^{1}H NMR (400 MHz, Chloroform-*d*) δ 7.36 – 7.26 (m, 2H), 7.25 – 7.18 (m, 3H), 2.93 (t, *J* = 7.6 Hz, 2H), 2.76 (dd, *J* = 8.3, 6.9 Hz, 2H), 2.44 (q, *J* = 7.3 Hz, 2H), 1.07 (t, *J* = 7.3 Hz, 3H).

^{13}C NMR (101 MHz, Chloroform-*d*) δ 210.7, 141.2, 128.5, 128.3, 126.1, 43.9, 36.2, 29.9, 7.8.

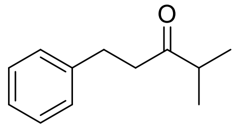

4-methyl-1-phenylpentan-3-one (33). Prepared as described above. Purified by silica column chromatography using petroleum ether/EtOAc to give 69% yield (121.4 mg) of the product as a colorless oil. ^{1}H NMR and ^{13}C NMR data match previously reported data. (Known compound: *Chem. Eur. J.*, **2016**, *22*, 15659–15663).

R_f value: 0.5 (PE:EA = 10:1).

^{1}H NMR (400 MHz, Chloroform-*d*) δ 7.34 – 7.27 (m, 2H), 7.25 – 7.18 (m, 3H), 2.96 – 2.89 (m, 2H), 2.84 – 2.76 (m, 2H), 2.64 – 2.55 (m, 1H), 1.10 (d, *J* = 7.0 Hz, 6H).

^{13}C NMR (101 MHz, Chloroform-*d*) δ 213.8, 141.4, 128.5, 128.4, 126.1, 42.0, 41.0, 29.9, 18.2.

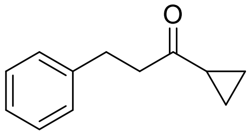

1-cyclopropyl-3-phenylpropan-1-one (34). Prepared as described above. Purified by silica column chromatography using petroleum ether/EtOAc to give 58% yield (85.8 mg) of the product as a colorless oil. ^{1}H NMR and ^{13}C NMR data match previously reported data. (Known compound: *Org. Lett.*, **2023**, *25*, 1305−1309).

R_f value: 0.45 (PE:EA = 10:1).

^{1}H NMR (400 MHz, Chloroform-*d*) δ 7.33 – 7.26 (m, 2H), 7.24 – 7.17 (m, 3H), 2.97 – 2.85 (m, 4H), 1.92 (tt, *J* = 7.8, 4.6 Hz, 1H), 1.02 (dt, *J* = 4.7, 3.3 Hz, 2H), 0.87 (dt, *J* = 8.0, 3.4 Hz, 2H).

^{13}C NMR (101 MHz, Chloroform-*d*) δ 210.0, 141.3, 128.5, 128.4, 126.1, 45.0, 30.0, 20.6, 10.8.

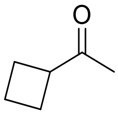

1-cyclobutylethan-1-one (35). Prepared as described above. Purified by silica column chromatography using petroleum ether/EtOAc to give 41% yield (40.2 mg) of the product as a colorless oil. ^{1}H NMR and ^{13}C NMR data match previously reported data. (Known compound: *Org. Biomol. Chem.*, **2016**, *14*, 2057-2089).

R$_f$ value: 0.55 (PE:EA = 10:1).

^{1}H NMR (400 MHz, Chloroform-*d*) δ 3.29 – 3.17 (m, 1H), 2.25 – 2.07 (m, 4H), 2.05 (s, 3H), 2.01 – 1.87 (m, 1H), 1.85 – 1.73 (m, 1H).

^{13}C NMR (101 MHz, Chloroform-*d*) δ 210.1, 46.2, 27.0, 24.3, 17.6.

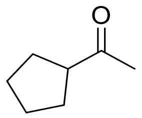

1-cyclopentylethan-1-one (36). Prepared as described above. Purified by silica column chromatography using petroleum ether/EtOAc to give 47% yield (52.6 mg) of the product as a colorless oil. ^{1}H NMR and ^{13}C NMR data match previously reported data. (Known compound: *Chem. Commun.*, **2019,** *55*, 14143-14146).

R$_f$ value: 0.6 (PE:EA = 10:1).

^{1}H NMR (400 MHz, Chloroform-*d*) δ 2.89 – 2.79 (m, 1H), 2.13 (s, 3H), 1.88 – 1.47 (m, 8H).

^{13}C NMR (101 MHz, Chloroform-*d*) δ 211.3, 52.2, 28.8, 28.7, 26.0.

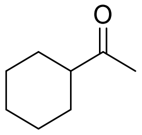

1-cyclohexylethan-1-one (37). Prepared as described above. Purified by silica column chromatography using petroleum ether/EtOAc to give 50% yield (63.1 mg) of the product as a colorless oil. ^{1}H NMR and ^{13}C NMR data match previously reported data. (Known compound: *ChemCatChem.*, **2016**, *8*, 262).

R$_f$ value: 0.64 (PE:EA = 10:1).

^{1}H NMR (400 MHz, Chloroform-*d*) δ 2.35 – 2.25 (m, 1H), 2.11 (s, 3H), 1.89 – 1.60 (m, 5H), 1.38 – 1.11 (m, 5H).

^{13}C NMR (101 MHz, Chloroform-*d*) δ 28.4, 27.9, 25.9, 25.6.

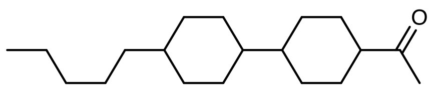

1-(4'-pentyl-[1,1'-bi(cyclohexan)]-4-yl)ethan-1-one (38). Prepared as described above. Purified by silica column chromatography using petroleum ether/EtOAc to give 66% yield (183.5 mg) of the product

as a colorless oil.

R_f value: 0.75 (PE:EA = 10:1)

^{1}H NMR (400 MHz, Chloroform-*d*) δ 2.24 (tt, *J* = 12.2, 3.5 Hz, 1H), 2.11 (s, 3H), 1.97 – 1.87 (m, 2H), 1.85 – 1.64 (m, 6H), 1.35 – 0.78 (m, 22H).

^{13}C NMR (101 MHz, Chloroform-*d*) δ 212.5, 51.9, 43.3, 42.7, 37.9, 37.4, 33.6, 32.2, 30.0, 29.3, 28.7, 27.9, 26.7, 22.7, 14.1.

HRMS: Calculated for $C_{19}H_{34}O$, $[M+H]^+$ 279.2683; found 279.2681.

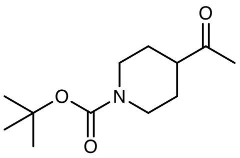

***tert*-butyl 4-acetylpiperidine-1-carboxylate (39).** Prepared as described above. Purified by silica column chromatography using petroleum ether/EtOAc to give 50% yield (113.5 mg) of the product as a white solid. ^{1}H NMR and ^{13}C NMR data match previously reported data. (Known compound: *J. Med. Chem.,* **2023**, *66*, 4025-4044).

R_f value: 0.29 (PE:EA = 4:1).

^{1}H NMR (400 MHz, Chloroform-*d*) δ 4.06 (d, *J* = 13.2 Hz, 2H), 2.75 (t, *J* = 13.3 Hz, 2H), 2.43 (tt, *J* = 11.4, 3.7 Hz, 1H), 2.13 (s, 3H), 1.80 (d, *J* = 13.2 Hz, 2H), 1.55 – 1.44 (m, 2H), 1.42 (s, 9H).

^{13}C NMR (101 MHz, Chloroform-*d*) δ 210.1, 154.7, 79.6, 49.2, 28.4, 27.8, 27.4.

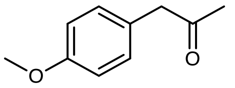

1-(4-methoxyphenyl)propan-2-one (40). Prepared as described above. Purified by silica column chromatography using petroleum ether/EtOAc to give 76% yield (124.6 mg) of the product as a yellow oil. ^{1}H NMR and ^{13}C NMR data match previously reported data. (Known compound: *Chem. Commun.*, **2018,** *54*, 11340-11343).

R_f value: 0.3 (PE:EA = 10:1).

^{1}H NMR (400 MHz, Chloroform-*d*) δ 7.16 – 7.07 (m, 2H), 6.92 – 6.82 (m, 2H), 3.79 (s, 3H), 3.62 (s, 2H), 2.13 (s, 3H).

^{13}C NMR (101 MHz, Chloroform-*d*) δ 206.9, 158.7, 130.4, 126.3, 114.2, 55.3, 50.1, 29.1.

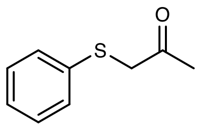

1-(phenylthio)propan-2-one (41). Prepared as described above. Purified by silica column chromatography using petroleum ether/EtOAc to give 81% yield (134.5 mg) of the product as a yellow oil. ^{1}H NMR and ^{13}C NMR data match previously reported data. (Known compound: *Org. Lett.*, **2012**, *14,* 2346–2349).

R$_f$ value: 0.27 (PE:EA = 10:1).

^{1}H NMR (400 MHz, Chloroform-*d*) δ 7.37 – 7.25 (m, 4H), 7.24 – 7.18 (m, 1H), 3.67 (s, 2H), 2.27 (s, 3H).

^{13}C NMR (101 MHz, Chloroform-*d*) δ 203.6, 134.7, 129.5, 129.2, 126.9, 44.7, 28.0.

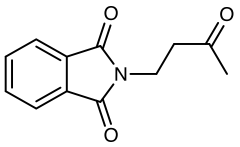

2-(3-oxobutyl)isoindoline-1,3-dione (42). Prepared as described above. Purified by silica column chromatography using petroleum ether/EtOAc to give 49% yield (106.3 mg) of the product as a white solid. ^{1}H NMR and ^{13}C NMR data match previously reported data. (Known compound: *Org. Lett.*, 2020, *22*, 239-243).

R$_f$ value: 0.36 (PE:EA = 4:1).

^{1}H NMR (400 MHz, Chloroform-*d*) δ 7.88 – 7.77 (m, 2H), 7.76 – 7.63 (m, 2H), 3.94 (td, *J* = 7.4, 0.9 Hz, 2H), 2.86 (t, *J* = 7.4 Hz, 2H), 2.17 (s, 3H).

^{13}C NMR (101 MHz, Chloroform-*d*) δ 205.8, 168.1, 134.0, 132.1, 123.3, 41.6, 33.0, 29.9.

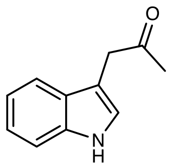

1-(1*H*-indol-3-yl)propan-2-one (43). Prepared as described above. Purified by silica column chromatography using petroleum ether/EtOAc to give 77% yield (133.2 mg) of the product as a yellow

solid. ^{1}H NMR and ^{13}C NMR data match previously reported data. (Known compound: *Org. Biomol. Chem.*, **2022**, *20*, 3292-3302).

Mp: 114.3-116.2 °C

R_f value: 0.27 (PE:EA = 10:1).

^{1}H NMR (400 MHz, Chloroform-*d*) δ 8.31 (s, 1H), 7.62 – 7.51 (m, 1H), 7.38 – 7.33 (m, 1H), 7.25 – 7.11 (m, 2H), 7.10 – 7.03 (m, 1H), 3.83 (s, 2H), 2.19 (s, 3H).

^{13}C NMR (101 MHz, Chloroform-*d*) δ 207.9, 136.2, 127.3, 123.3, 122.3, 119.8, 118.7, 111.4, 108.6, 40.9, 29.0.

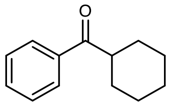

cyclohexyl(phenyl)methanone (44). Prepared as described above. Purified by silica column chromatography using petroleum ether/EtOAc to give 53% yield (99.6 mg) of the product as a yellow oil. ^{1}H NMR and ^{13}C NMR data match previously reported data. (Known compound: *Chem. Commun.*, **2020**, *56*, 12443-12446).

R_f value: 0.62 (PE:EA = 10:1).

^{1}H NMR (400 MHz, Chloroform-*d*) δ 8.05 – 7.91 (m, 2H), 7.56 (t, *J* = 7.3 Hz, 1H), 7.48 (t, *J* = 7.6 Hz, 2H), 3.29 (tt, *J* = 11.5, 3.3 Hz, 1H), 2.00 – 1.82 (m, 4H), 1.80 – 1.68 (m, 1H), 1.62 – 1.19 (m, 5H).

^{13}C NMR (101 MHz, Chloroform-*d*) δ 203.9, 136.4, 132.7, 128.6, 128.3, 45.6, 29.4, 26.0, 25.9.

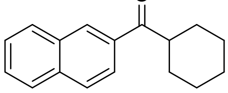

cyclohexyl(naphthalen-2-yl)methanone (45). Prepared as described above. Purified by silica column chromatography using petroleum ether/EtOAc to give 56% yield (131.6 mg) of the product as a yellow oil. ^{1}H NMR and ^{13}C NMR data match previously reported data.

R_f value: 0.6 (100% PE).

^{1}H NMR (400 MHz, Chloroform-*d*) δ 8.46 (s, 1H), 8.06 – 7.95 (m, 2H), 7.88 (t, *J* = 8.6 Hz, 2H), 7.64 – 7.49 (m, 2H), 3.44 (tt, *J* = 11.4, 3.4 Hz, 1H), 2.01 – 1.84 (m, 4H), 1.82 – 1.73 (m, 1H), 1.63 – 1.40 (m, 4H), 1.37 – 1.25 (m, 1H).

^{13}C NMR (101 MHz, Chloroform-*d*) δ 203.9, 135.5, 133.7, 132.7, 129.6 (d, *J* = 1.8 Hz), 128.5, 128.3,

127.8, 126.7, 124.4, 45.7, 29.6, 26.0, 26.0.

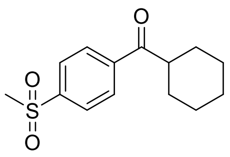

cyclohexyl(4-(methylsulfonyl)phenyl)methanone (46). Prepared as described above. Purified by silica column chromatography using petroleum ether/EtOAc to give 40% yield (106.4 mg) of the product as a white solid. ^{1}H NMR and ^{13}C NMR data match previously reported data.

R_f value: 0.3 (PE:EA = 3:1).

^{1}H NMR (400 MHz, Chloroform-*d*) δ 8.15 – 7.89 (m, 4H), 3.23 (tt, *J* = 11.3, 3.2 Hz, 1H), 3.07 (s, 3H), 1.92 – 1.79 (m, 4H), 1.78 – 1.68 (m, 1H), 1.54 – 1.17 (m, 5H).

^{13}C NMR (101 MHz, Chloroform-*d*) δ 202.6, 143.8, 140.5, 129.1, 127.8, 46.1, 44.3, 29.2, 25.8, 25.7.

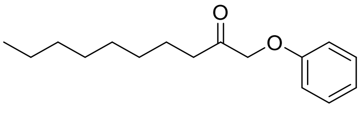

1-phenoxydecan-2-one (47). Prepared as described above. Purified by silica column chromatography using petroleum ether/EtOAc to give 82% yield (203.3 mg) of the product as a yellow solid.

R_f value: 0.8 (PE:EA = 10:1).

^{1}H NMR (400 MHz, Chloroform-*d*) δ 7.42 – 7.29 (m, 2H), 7.02 (td, *J* = 7.3, 1.1 Hz, 1H), 6.91 (dd, *J* = 7.6, 1.6 Hz, 2H), 4.57 (s, 2H), 2.61 (t, *J* = 7.4 Hz, 2H), 1.70 – 1.59 (m, 2H), 1.34 – 1.25 (m, 10H), 0.92 – 0.88 (m, 3H).

^{13}C NMR (101 MHz, Chloroform-*d*) δ 203.0, 158.6, 129.5, 121.1, 114.7, 66.4, 42.8, 31.8, 29.4, 29.3, 29.2, 23.9, 22.66, 14.1.

HRMS: Calculated for $C_{16}H_{24}O_2$, $[M+H]^+$ 249.1849; found 249.1840.

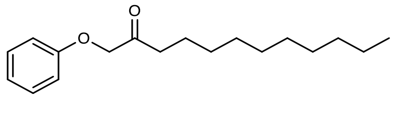

1-phenoxydodecan-2-one (48). Prepared as described above. Purified by silica column chromatography using petroleum ether/EtOAc to give 83% yield (229.1 mg) of the product as a yellow oil.

R_f value: 0.8 (PE:EA = 10:1).

^{1}H NMR (400 MHz, Chloroform-*d*) δ 7.37 – 7.29 (m, 2H), 7.05 – 6.99 (m, 1H), 6.94 – 6.88 (m, 2H), 4.57 (s, 2H), 2.61 (t, *J* = 7.4 Hz, 2H), 1.29 (m, 16H), 0.91 (t, *J* = 6.8 Hz, 3H).

^{13}C NMR (101 MHz, Chloroform-*d*) δ 208.1, 157.8, 129.7, 121.7, 114.5, 72.8, 39.1, 31.9, 29.6, 29.5, 29.4, 29.3, 29.2, 23.2, 22.7, 14.1.

HRMS: Calculated for $C_{18}H_{28}O_2$, $[M+H]^+$ 277.2162; found 277.2160.

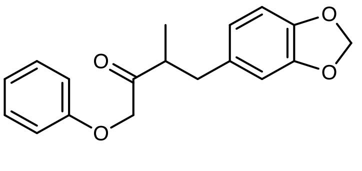

4-(benzo[d][1,3]dioxol-5-yl)-3-methyl-1-phenoxybutan-2-one (49). Prepared as described above. Purified by silica column chromatography using petroleum ether/EtOAc to give 71% yield (211.6 mg) of the product as a yellow oil.

R_f value: 0.44 (PE:EA = 10:1).

^{1}H NMR (400 MHz, Chloroform-*d*) δ 7.33 – 7.24 (m, 2H), 7.04 – 6.97 (m, 1H), 6.83 – 6.78 (m, 2H), 6.74 (d, *J* = 8.0 Hz, 1H), 6.70 – 6.62 (m, 2H), 5.94 – 5.91 (m, 2H), 4.60 – 4.40 (m, 2H), 3.22 – 3.11 (m, 1H), 3.02 – 2.94 (m, 1H), 2.60 – 2.52 (m, 1H), 1.17 (d, *J* = 6.9 Hz, 3H).

^{13}C NMR (101 MHz, Chloroform-*d*) δ 210.4, 157.8, 147.7, 146.1, 133.1, 129.6, 122.0, 114.5, 109.4, 100.9, 72.3, 44.3, 38.6, 16.3.

HRMS: Calculated for $C_{18}H_{18}O_4$, $[M+H]^+$ 299.1278; found 299.1280.

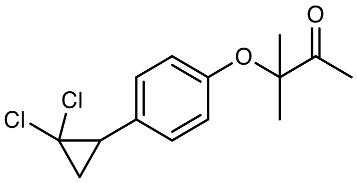

3-(4-(2,2-dichlorocyclopropyl)phenoxy)-3-methylbutan-2-one (50). Prepared as described above. Purified by silica column chromatography using petroleum ether/EtOAc to give 44% yield (125.8 mg) of the product as a yellow oil. ^{1}H NMR and ^{13}C NMR data match previously reported data. (Known compound: *Org. Chem. Front.,* **2023***, 10,* 3669-3675).

R_f value: 0.44 (PE:EA = 10:1).

^{1}H NMR (400 MHz, Chloroform-*d*) δ 7.16 – 7.02 (m, 2H), 6.78 – 6.68 (m, 2H), 2.82 (dd, *J* = 10.7, 8.3 Hz, 1H), 2.26 (s, 3H), 1.94 (dd, *J* = 10.7, 7.4 Hz, 1H), 1.77 (dd, *J* = 8.4, 7.4 Hz, 1H), 1.48 (s, 6H).

13C NMR (101 MHz, Chloroform-*d*) δ 212.1, 154.8, 129.9, 128.2, 118.2, 84.2, 60.8, 34.8, 25.9, 24.4, 23.9 (d, J = 1.3 Hz).

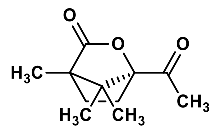

(1S)-1-acetyl-4,7,7-trimethyl-2-oxabicyclo[2.2.1]heptan-3-one (51). Prepared as described above. Purified by silica column chromatography using petroleum ether/EtOAc to give 42% yield (82.3 mg) of the product as a white solid.

R_f value: 0.17 (PE:EA = 10:1).

1H NMR (400 MHz, Chloroform-*d*) δ 2.40 – 2.31 (m, 1H), 2.30 (s, 3H), 1.94 – 1.81 (m, 2H), 1.69 – 1.59 (m, 1H), 1.08 (s, 3H), 1.00 (s, 3H), 0.92 (s, 3H).

13C NMR (101 MHz, Chloroform-*d*) δ 206.0, 178.7, 96.3, 55.4, 54.7, 30.5, 28.9, 28.6, 16.7, 16.4, 9.5.

HRMS: Calculated for $C_{11}H_{16}O_3$, $[M+H]^+$ 197.1172; found 197.1170.

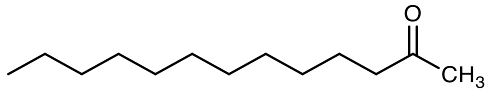

tridecan-2-one (52). Prepared as described above. Purified by silica column chromatography using petroleum ether/EtOAc to give 61% yield (120.9 mg) of the product as a white solid 1H NMR and 13C NMR data match previously reported data. (Known compound: *Adv. Synth. Catal.*, **2011**, *353*, 1005-1017).

R_f value: 0.68 (PE:EA = 10:1).

1H NMR (400 MHz, Chloroform-*d*) δ 2.40 (t, J = 7.5 Hz, 2H), 2.12 (s, 3H), 1.61 – 1.50 (m, 2H), 1.34 – 1.19 (m, 16H), 0.87 (t, J = 6.7 Hz, 3H).

13C NMR (101 MHz, Chloroform-*d*) δ 209.4, 43.8, 31.9, 29.8, 29.6, 29.5, 29.4, 29.3, 29.2, 23.9, 22.7, 14.1.

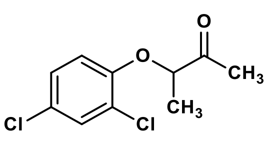

3-(2,4-dichlorophenoxy)butan-2-one (53). Prepared as described above. Purified by silica column chromatography using petroleum ether/EtOAc to give 80% yield (185.6 mg) of the product as a white solid.

R_f value: 0.53 (PE:EA = 10:1).

^{1}H NMR (400 MHz, Chloroform-*d*) δ 7.41 (d, *J* = 2.5 Hz, 1H), 7.16 (dd, *J* = 8.8, 2.5 Hz, 1H), 6.74 (d, *J* = 8.8 Hz, 1H), 4.62 (q, *J* = 6.8 Hz, 1H), 2.25 (s, 3H), 1.55 (d, *J* = 6.8 Hz, 3H).

^{13}C NMR (101 MHz, Chloroform-*d*) δ 208.9, 151.8, 130.5, 127.7, 126.9, 124.5, 115.2, 80.6, 24.8, 17.2.

HRMS: Calculated for $C_{10}H_{10}Cl_2O_2$, $[M+H]^+$ 233.0131; found 233.0121.

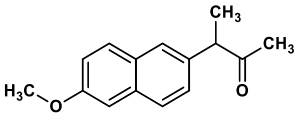

3-(6-methoxynaphthalen-2-yl)butan-2-one (54). Prepared as described above. Purified by silica column chromatography using petroleum ether/EtOAc to give 63% yield (143.6 mg) of the product as a yellow oil. ^{1}H NMR and ^{13}C NMR data match previously reported data. (Known compound: *Org. Lett.*, **2022**, *24*, 4804-4809).

R_f value: 0.31 (PE:EA = 10:1).

^{1}H NMR (400 MHz, Chloroform-*d*) δ 7.74 (dd, *J* = 8.6, 4.4 Hz, 2H), 7.64 (d, *J* = 1.8 Hz, 1H), 7.31 (dd, *J* = 8.5, 1.9 Hz, 1H), 7.22 – 7.13 (m, 2H), 3.94 (s, 3H), 3.90 (q, *J* = 7.0 Hz, 1H), 2.09 (s, 3H), 1.49 (d, *J* = 6.9 Hz, 3H).

^{13}C NMR (101 MHz, Chloroform-*d*) δ 209.0, 157.8, 135.8, 133.7, 129.2, 129.1, 127.6, 126.5, 126.3, 119.2, 105.7, 55.3, 53.7, 28.4, 17.3.

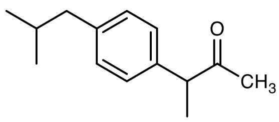

3-(4-isobutylphenyl)butan-2-one (55). Prepared as described above. Purified by silica column chromatography using petroleum ether/EtOAc to give 55% yield (112.2 mg) of the product as a yellow oil. ^{1}H NMR and ^{13}C NMR data match previously reported data. (Known compound: *Org. Lett.*, **2022**, *24*, 4804-4809).

R_f value: 0.66 (PE:EA = 10:1).

^{1}H NMR (400 MHz, Chloroform-*d*) δ 7.16 – 7.08 (m, 4H), 3.71 (q, *J* = 7.0 Hz, 1H), 2.45 (d, *J* = 7.1 Hz,

2H), 2.04 (d, *J* = 1.0 Hz, 3H), 1.91 – 1.79 (m, 1H), 1.38 (dd, *J* = 7.0, 0.9 Hz, 3H), 0.90 (dd, *J* = 6.7, 0.9 Hz, 6H).

^{13}C NMR (101 MHz, Chloroform-*d*) δ 209.2, 140.7, 137.8, 129.7, 127.5, 53.4, 45.0, 30.2, 28.3, 22.4, 17.2.

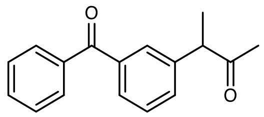

3-(3-benzoylphenyl)butan-2-one (56). Prepared as described above. Purified by silica column chromatography using petroleum ether/EtOAc to give 50% yield (126.1 mg) of the product as a yellow oil. ^{1}H NMR and ^{13}C NMR data match previously reported data. (Known compound: *J. Am. Chem. Soc.*, **2020**, *142*, 7683-7689).

R_f value: 0.26 (PE:EA = 10:1).

^{1}H NMR (400 MHz, Chloroform-*d*) δ 7.83 – 7.76 (m, 2H), 7.72 – 7.64 (m, 2H), 7.59 (t, *J* = 7.4 Hz, 1H), 7.53 – 7.40 (m, 4H), 3.83 (q, *J* = 7.0 Hz, 1H), 2.08 (s, 3H), 1.43 (d, *J* = 7.0 Hz, 3H).

^{13}C NMR (101 MHz, Chloroform-*d*) δ 208.2, 196.4, 140.9, 138.3, 137.4, 132.6, 131.7, 130.0, 129.5, 129.1, 128.9, 128.4, 53.5, 28.5, 17.3.

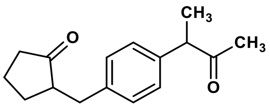

2-(4-(3-oxobutan-2-yl)benzyl)cyclopentan-1-one (57). Prepared as described above. Purified by silica column chromatography using petroleum ether/EtOAc to give 57% yield (139.1 mg) of the product as a yellow oil.

R_f value: 0.15 (PE:EA = 10:1).

^{1}H NMR (400 MHz, Chloroform-*d*) δ 7.23 – 7.06 (m, 4H), 3.73 (q, *J* = 7.0 Hz, 1H), 3.13 (dd, *J* = 13.9, 4.2 Hz, 1H), 2.52 (dd, *J* = 13.9, 9.4 Hz, 1H), 2.42 – 2.28 (m, 2H), 2.20 – 2.06 (m, 2H), 2.05 (s, 3H), 2.03 – 1.91 (m, 1H), 1.83 – 1.69 (m, 1H), 1.64 – 1.51 (m, 1H), 1.38 (d, *J* = 6.9 Hz, 3H).

^{13}C NMR (101 MHz, Chloroform-*d*) δ 208.9, 139.0, 138.4, 129.4, 127.9, 53.3, 50.9, 38.2, 35.2, 29.2, 28.3, 20.5, 17.2.

HRMS: Calculated for $C_{16}H_{20}O_2$, $[M+H]^+$ 244.1463; found 244.1461.

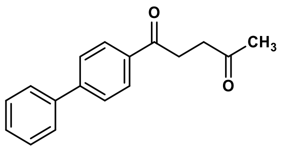

1-([1,1'-biphenyl]-4-yl)pentane-1,4-dione (58). Prepared as described above. Purified by silica column chromatography using petroleum ether/EtOAc to give 66% yield (166.3 mg) of the product as a white solid. ^{1}H NMR and ^{13}C NMR data match previously reported data. (Known compound: *ACS Catal.,* **2016***,* *6,* 2982–2987).

Rf value: 0.38 (PE:EA = 4:1).

^{1}H NMR (400 MHz, Chloroform-*d*) δ 8.11 – 7.94 (m, 2H), 7.72 – 7.67 (m, 2H), 7.65 – 7.61 (m, 2H), 7.48 – 7.42 (m, 3H), 3.31 (t, *J* = 6.3 Hz, 2H), 2.91 (t, *J* = 6.3 Hz, 2H), 2.27 (s, 3H).

^{13}C NMR (101 MHz, Chloroform-*d*) δ 207.4, 198.2, 145.9, 139.9, 135.4, 129.0, 128.7, 128.3, 127.3 (d, *J* = 3.7 Hz), 37.1, 32.5, 30.2.

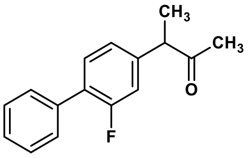

3-(2-fluoro-[1,1'-biphenyl]-4-yl)butan-2-one (59). Prepared as described above. Purified by silica column chromatography using petroleum ether/EtOAc to give 56% yield (135.5 mg) of the product as a yellow oil. ^{1}H NMR and ^{13}C NMR data match previously reported data. (Known compound: *Nat. Commun.*, **2022**, *13*, 2846).

Rf value: 0.37(PE:EA = 10:1).

^{1}H NMR (400 MHz, Chloroform-*d*) δ 7.60 – 7.54 (m, 2H), 7.50 – 7.37 (m, 4H), 7.13 – 7.04 (m, 2H), 3.82 (q, *J* = 7.0 Hz, 1H), 2.15 (s, 3H), 1.46 (d, *J* = 7.0 Hz, 3H).

^{13}C NMR (101 MHz, Chloroform-*d*) δ 208.1, 159.9 (d, *J* = 248.9 Hz), 141.9 (d, *J* = 7.6 Hz), 135.4 (d, *J* = 1.4 Hz), 131.2 (d, *J* = 4.0 Hz), 129.0 (d, *J* = 2.9 Hz), 128.5, 127.9 (d, *J* = 13.4 Hz), 127.8, 123.9 (d, *J* = 3.3 Hz), 115.5 (d, *J* = 23.3 Hz), 53.1 (d, *J* = 1.4 Hz), 28.5, 17.2.

^{19}F NMR (376 MHz, Chloroform-d) δ -117.18.

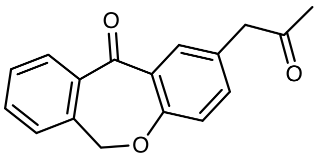

2-(2-oxopropyl)dibenzo[b,e]oxepin-11(6H)-one (60). Prepared as described above. Purified by silica column chromatography using petroleum ether/EtOAc to give 49% yield (130.3 mg) of the product as a yellow oil. ^{1}H NMR and ^{13}C NMR data match previously reported data. (Known compound: *Angew. Chem. Int. Ed.*, **2023**, *62*, e202214633).

R$_f$ value: 0.19 (PE:EA = 10:1).

^{1}H NMR (400 MHz, Chloroform-*d*) δ 8.04 (d, *J* = 2.4 Hz, 1H), 7.89 (dd, *J* = 7.7, 1.4 Hz, 1H), 7.55 (td, *J* = 7.5, 1.4 Hz, 1H), 7.47 (td, *J* = 7.6, 1.3 Hz, 1H), 7.39 – 7.29 (m, 2H), 7.03 (d, *J* = 8.4 Hz, 1H), 5.18 (s, 2H), 3.73 (s, 2H), 2.19 (s, 3H).

^{13}C NMR (101 MHz, Chloroform-*d*) δ 206.0, 190.9, 160.5, 140.4, 136.5, 135.6, 132.8, 132.5, 129.5, 129.3, 128.0, 127.8, 125.3, 121.2, 73.6, 49.7, 29.5.

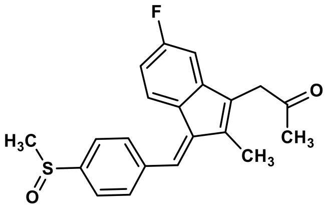

(Z)-1-(5-fluoro-2-methyl-1-(4-(methylsulfinyl)benzylidene)-1H-inden-3-yl)propan-2-one (61). Prepared as described above. Purified by silica column chromatography using petroleum ether/EtOAc to give 58% yield (205.3 mg) of the product as a orange oil.

R$_f$ value: 0.18 (PE:EA = 1:1).

^{1}H NMR (400 MHz, Chloroform-*d*) δ 7.74 – 7.61 (m, 4H), 7.18 – 7.09 (m, 2H), 6.77 (dd, *J* = 8.8, 2.4 Hz, 1H), 6.55 (td, *J* = 8.8, 2.5 Hz, 1H), 3.61 (s, 2H), 2.80 (s, 3H), 2.18 (d, *J* = 2.9 Hz, 6H).

^{13}C NMR (101 MHz, Chloroform-*d*) δ 205.0, 163.4 (d, *J* = 246.9 Hz), 146.7 (d, *J* = 8.7 Hz), 145.4, 141.6, 139.64, 138.1, 132.2 (d, *J* = 2.5 Hz), 130.3, 129.6 (d, *J* = 2.9 Hz), 128.2 (d, *J* = 1.9 Hz), 123.9, 123.8 (d, *J* = 3.1 Hz), 110.9 (d, *J* = 22.7 Hz), 106.0 (d, *J* = 23.9 Hz), 43.8, 41.4, 29.4, 10.6.

^{19}F NMR (376 MHz, Chloroform-*d*) δ -112.71.

HRMS: Calculated for $C_{21}H_{19}FO_2S$, $[M+H]^+$ 355.1163; found 355.1158.

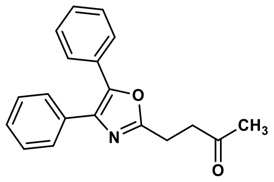

4-(4,5-diphenyloxazol-2-yl)butan-2-one (62). Prepared as described above. Purified by silica column chromatography using petroleum ether/EtOAc to give 53% yield (154.2 mg) of the product as a colorless oil. ^{1}H NMR and ^{13}C NMR data match previously reported data. (Known compound: *Nat. Catal.*, **2020**, *3*, 621-627).

R_f value: 0.25 (PE:EA = 8:1).

^{1}H NMR (400 MHz, Chloroform-*d*) δ 7.65 – 7.59 (m, 2H), 7.59 – 7.52 (m, 2H), 7.40 – 7.28 (m, 6H), 3.18 – 3.00 (m, 4H), 2.25 (s, 3H).

^{13}C NMR (101 MHz, Chloroform-*d*) δ 206.5, 162.3, 145.4, 135.0, 132.5, 129.0, 128.7, 128.6, 128.5, 128.1, 127.9, 126.5, 39.9, 30.0, 22.2.

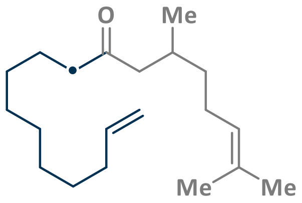

2,6-dimethyloctadeca-2,17-dien-8-one (63). Prepared as described above. Purified by silica column chromatography using petroleum ether/EtOAc to give 53% yield (154.9 mg) of the product as a yellow oil. ^{1}H NMR and ^{13}C NMR data match previously reported data. (Known compound: *J. Am. Chem. Soc.* **2019**, *141*, 6726-6739).

R_f value: 0.5(PE:EA = 10:1).

^{1}H NMR (400 MHz, Chloroform-*d*) δ 5.95 – 5.74 (m, 1H), 5.15 – 5.06 (m, 1H), 5.00 (dq, *J* = 17.1, 1.8 Hz, 1H), 4.97 – 4.91 (m, 1H), 2.45 – 2.35 (m, 3H), 2.21 (dd, *J* = 15.7, 8.1 Hz, 1H), 2.10 – 1.94 (m, 5H), 1.70 (s, 3H), 1.61 (s, 3H), 1.59 – 1.50 (m, 2H), 1.43 – 1.34 (m, 2H), 1.33 – 1.25 (m, 9H), 1.25 – 1.15 (m, 1H), 0.90 (d, *J* = 6.6 Hz, 3H).

^{13}C NMR (101 MHz, Chloroform-*d*) δ 211.4, 139.2 131.5, 124.4, 114.1, 50.3, 43.4, 37.0, 33.8, 29.4, 29.3, 29.3, 29.1, 28.9, 28.9, 25.7, 25.5, 23.8, 19.8, 17.7.

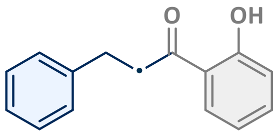

1-(2-hydroxyphenyl)-3-phenylpropan-1-one (64). Prepared as described above. Purified by silica column chromatography using petroleum ether/EtOAc to give 50% yield (113.0 mg) of the product as a yellow oil. ^{1}H NMR and ^{13}C NMR data match previously reported data.

R$_f$ value: 0.21 (PE:EA = 10:1).

^{1}H NMR (400 MHz, Chloroform-*d*) δ 12.36 (s, 1H), 7.78 (dd, *J* = 8.1, 1.7 Hz, 1H), 7.50 (ddd, *J* = 8.7, 7.1, 1.7 Hz, 1H), 7.40 – 7.32 (m, 2H), 7.32 – 7.21 (m, 3H), 7.03 (dd, *J* = 8.4, 1.2 Hz, 1H), 6.92 (ddd, *J* = 8.2, 7.2, 1.2 Hz, 1H), 3.37 (dd, *J* = 8.5, 6.9 Hz, 2H), 3.11 (dd, *J* = 8.5, 6.9 Hz, 2H).

^{13}C NMR (101 MHz, Chloroform-*d*) δ 205.4, 162.5, 140.8, 136.4, 129.9, 128.7, 128. 4, 126.4, 119.3, 119.0, 118.6, 40.1, 30.0.

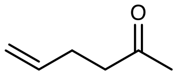

hex-5-en-2-one (65). Prepared as described above. Purified by silica column chromatography using petroleum ether/EtOAc to give 44% yield (43.1 mg) of the product as a colorless oil. ^{1}H NMR and ^{13}C NMR data match previously reported data. (Known compound: *Org. Lett.*, **2008**, *10*, 1927-1930).

R$_f$ value: 0.44 (PE:EA = 10:1).

^{1}H NMR (400 MHz, Chloroform-*d*) δ 5.79 (ddt, *J* = 16.8, 10.2, 6.5 Hz, 1H), 5.06 – 4.93 (m, 2H), 2.52 (t, *J* = 7.4 Hz, 2H), 2.35 – 2.28 (m, 2H), 2.14 (s, 3H).

^{13}C NMR (101 MHz, Chloroform-*d*) δ 208.2, 137.0, 115.2, 42.7, 30.0, 27.8.

8. Supplementary References

1. Yamase, T.; Takabayashi, N.; Kaji, M. SoIution Photochemistry of Tetrakis(tetrabutyIammonium) Decatungstate(VI) and Catalytic Hydrogen Evolution from Alcohols. *J. Chem. Soc., Dalton Trans,* **1984**, 793–799.
2. Y. Gao, B. Jiang, N. C. Friede, A. C. Hunter, D. G. Boucher, S. D. Minteer, M. S. Sigman, S. E. Reisman, P. S. Baran, *J. Am. Chem. Soc.* **2024**, *146*, 4872-4882.
3. T. M. Masson, S. D. A. Zondag, J. H. A. Schuurmans, T. Noël, *React. Chem. Eng.* **2024**, *9*, 2218-2225.
4. Y. Yang, X. Zhang, L.-P. Zhong, J. Lan, X. Li, C.-C. Li, L. W. Chung, *Nat. Commun.*, **2020,** *11*, 1850.
5. Frisch, M. J.; Trucks, G. W.; Schlegel, H. B.; Scuseria, G. E.; Robb, M. A.; Cheeseman, J. R.; Scalmani, G.; Barone, V.; Petersson, G. A.; Nakatsuji, H.; Li, X.; Caricato, M.; Marenich, A. V.; Bloino, J.; Janesko, B. G.; Gomperts, R.; Mennucci, B.; Hratchian, H. P.; Ortiz, J. V.; Izmaylov, A. F.; Sonnenberg, J. L.; Williams; Ding, F.; Lipparini, F.; Egidi, F.; Goings, J.; Peng, B.; Petrone, A.; Henderson, T.; Ranasinghe, D.; Zakrzewski, V. G.; Gao, J.; Rega, N.; Zheng, G.; Liang, W.; Hada, M.; Ehara, M.; Toyota, K.; Fukuda, R.; Hasegawa, J.; Ishida, M.; Nakajima, T.; Honda, Y.; Kitao, O.; Nakai, H.; Vreven, T.; Throssell, K.; Montgomery Jr., J. A.; Peralta, J. E.; Ogliaro, F.; Bearpark, M. J.; Heyd, J. J.; Brothers, E. N.; Kudin, K. N.; Staroverov, V. N.; Keith, T. A.; Kobayashi, R.; Normand, J.; Raghavachari, K.; Rendell, A. P.; Burant, J. C.; Iyengar, S. S.; Tomasi, J.; Cossi, M.; Millam, J. M.; Klene, M.; Adamo, C.; Cammi, R.; Ochterski, J. W.; Martin, R. L.; Morokuma, K.; Farkas, O.; Foresman, J. B.; Fox, D. J. Gaussian 16 Rev. A.01, Wallingford, CT, 2016.
6. Adamo, C. & Barone, V. Toward reliable density functional methods without adjustable parameters: The PBE0 model. *J. Chem. Phys.* **110**, 6158-6170, (1999).
7. Grimme, S., Ehrlich, S. & Goerigk, L. Effect of the damping function in dispersion corrected density functional theory. J. *Comput. Chem.* **32**, 1456-1465, (2011).

9. NMR Spectra

^{1}H NMR (400 MHz, $CDCl_3$) **1-(1-phenylcyclopropyl)ethan-1-one (1)**

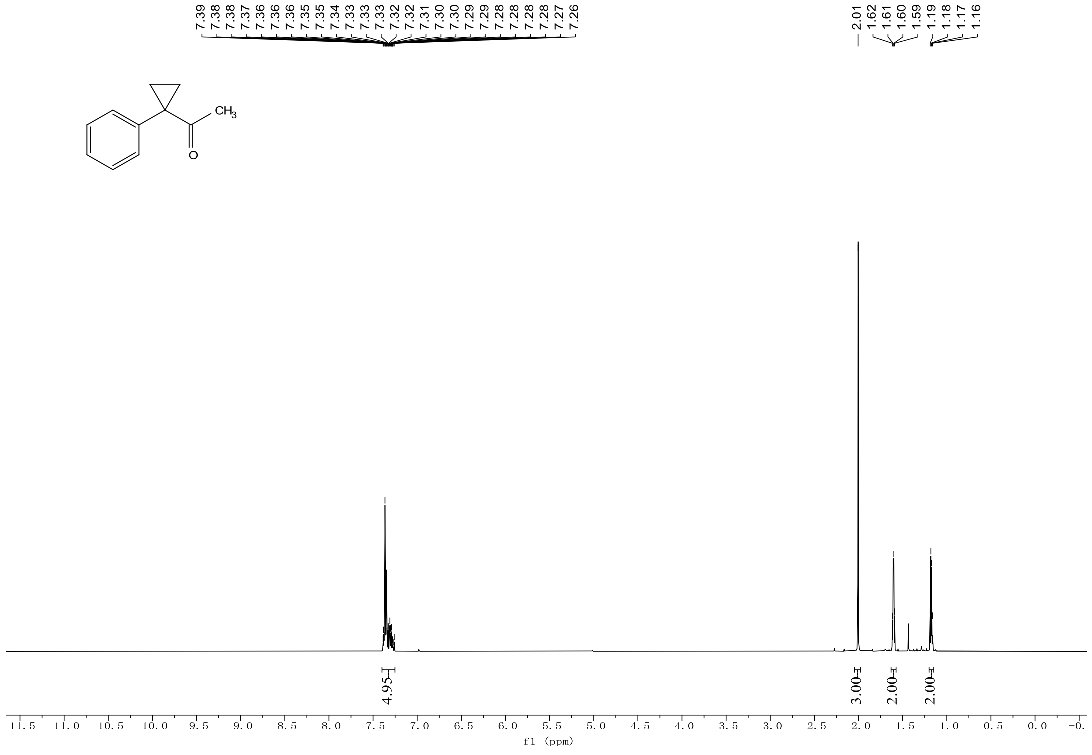

^{13}C NMR (101 MHz, $CDCl_3$) **1-(1-phenylcyclopropyl)ethan-1-one (1)**

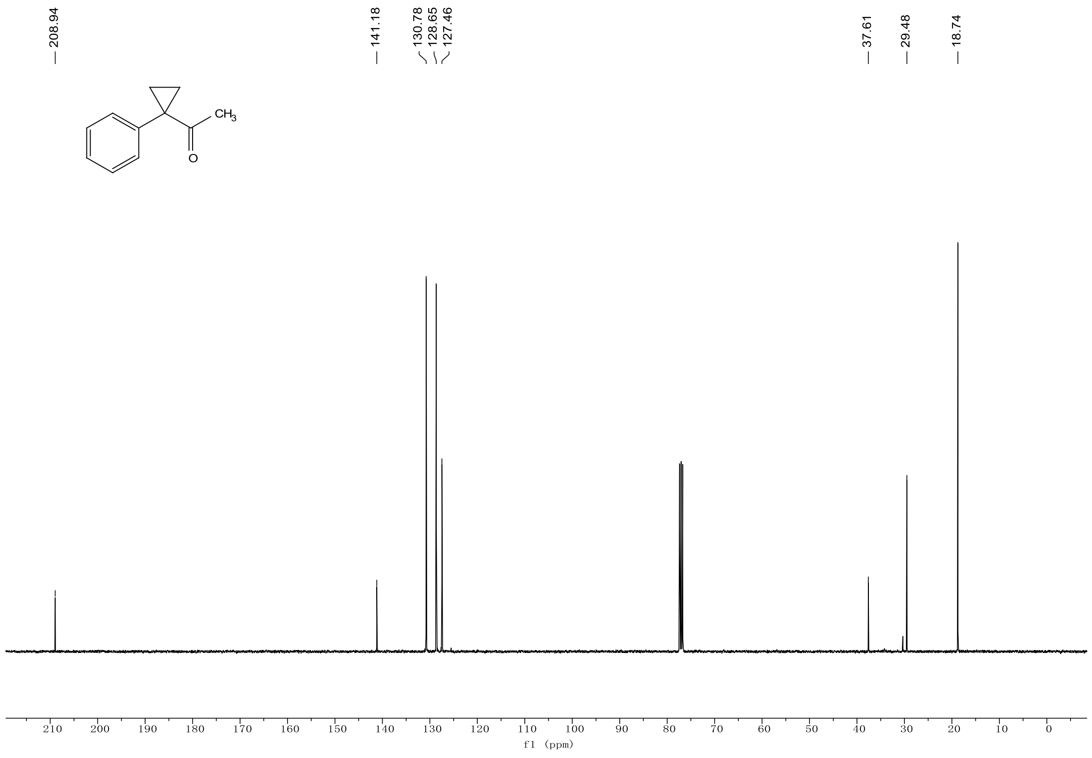

^{1}H NMR (400 MHz, $CDCl_3$) **1-(1-phenylcyclopropyl)propan-1-one (2)**

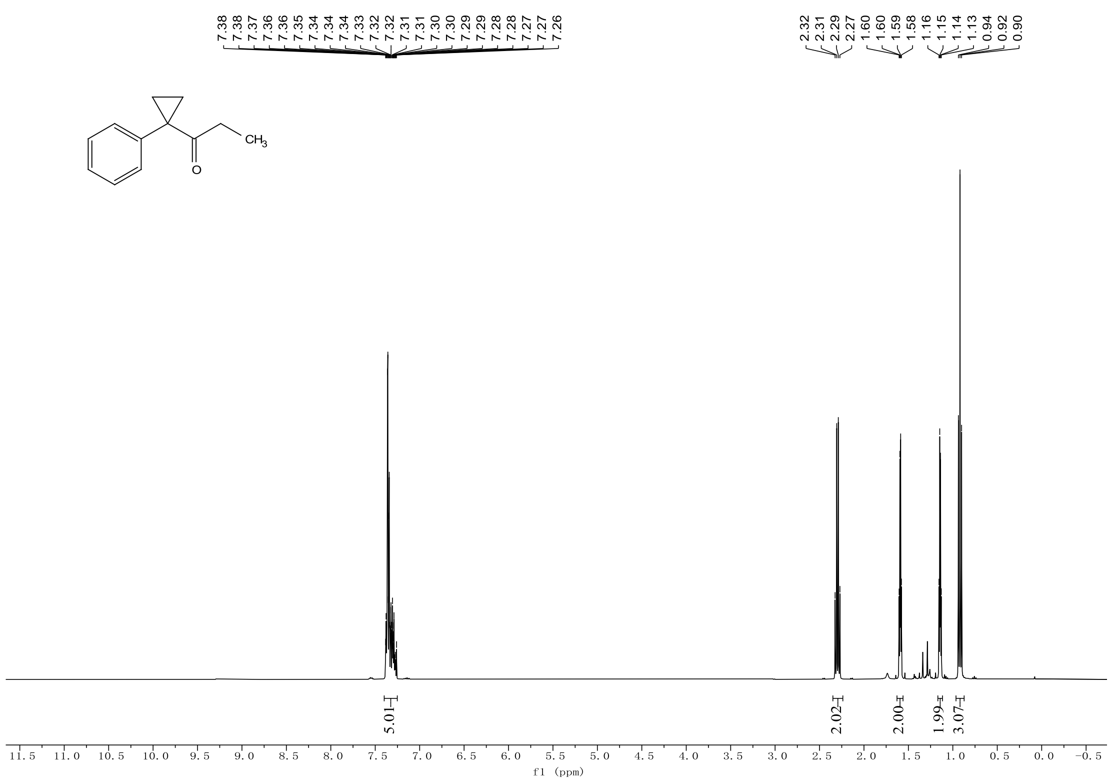

^{13}C NMR (101 MHz, $CDCl_3$) **1-(1-phenylcyclopropyl)propan-1-one (2)**

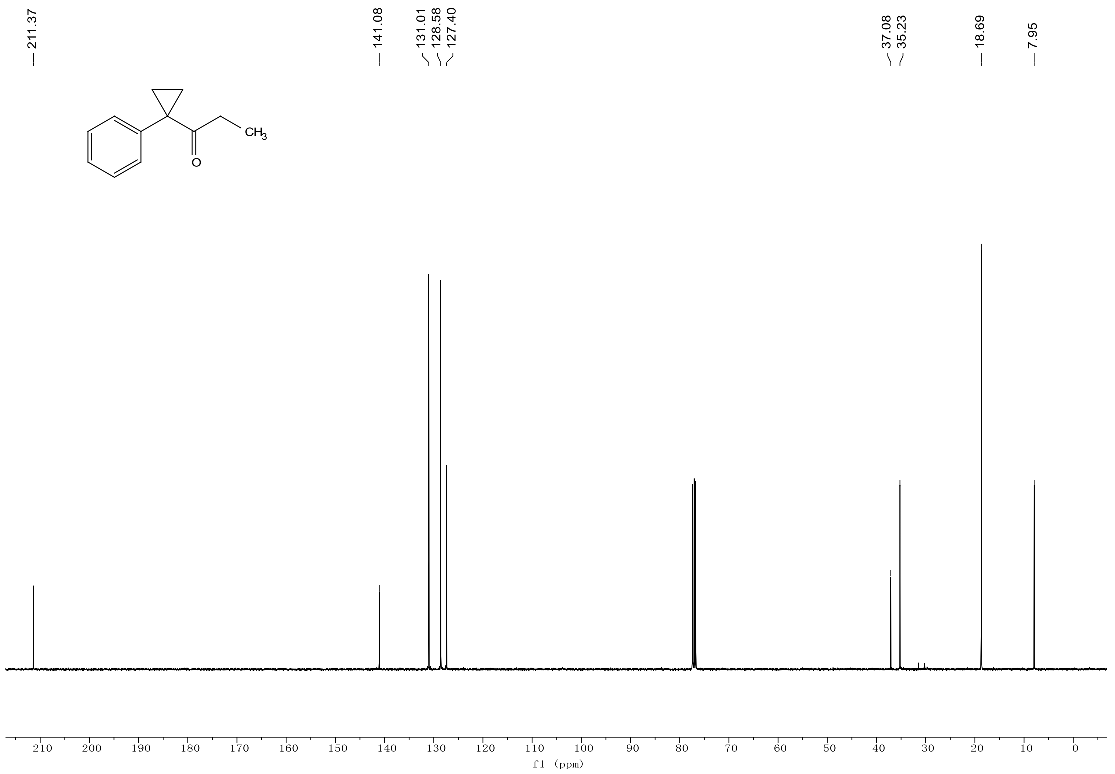

^{1}H NMR (400 MHz, $CDCl_3$) **2-phenyl-1-(1-phenylcyclopropyl)ethan-1-one (3)**

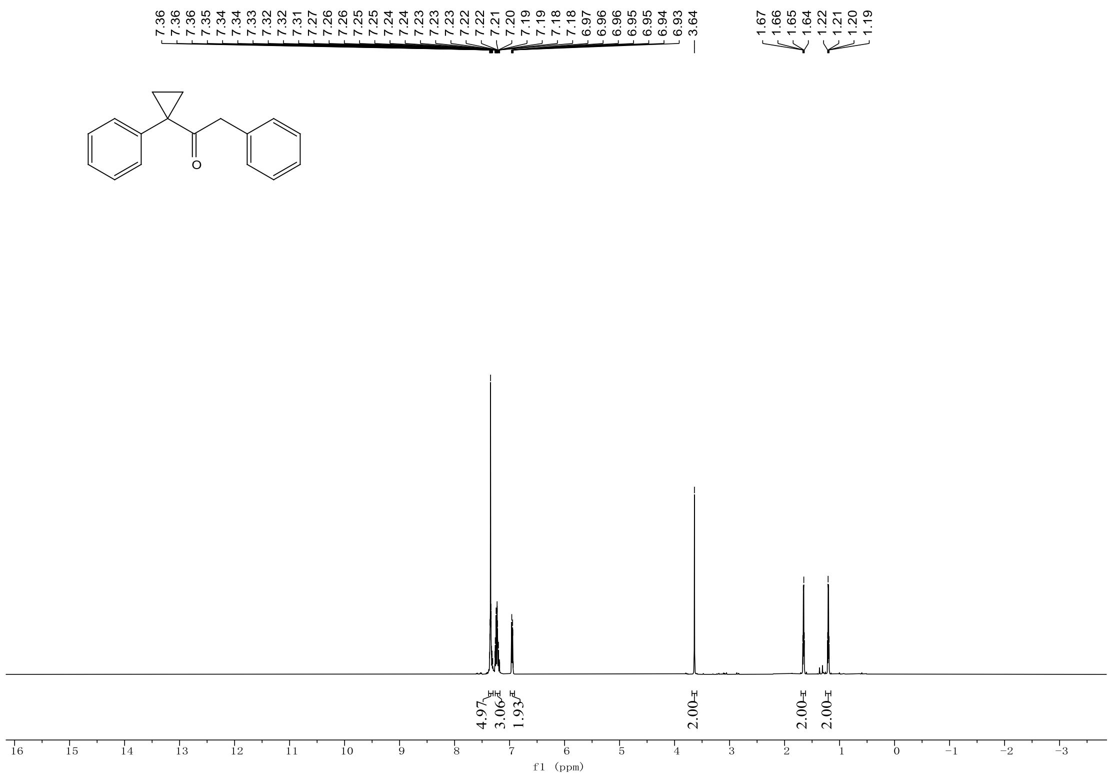

^{13}C NMR (101 MHz, $CDCl_3$) **2-phenyl-1-(1-phenylcyclopropyl)ethan-1-one (3)**

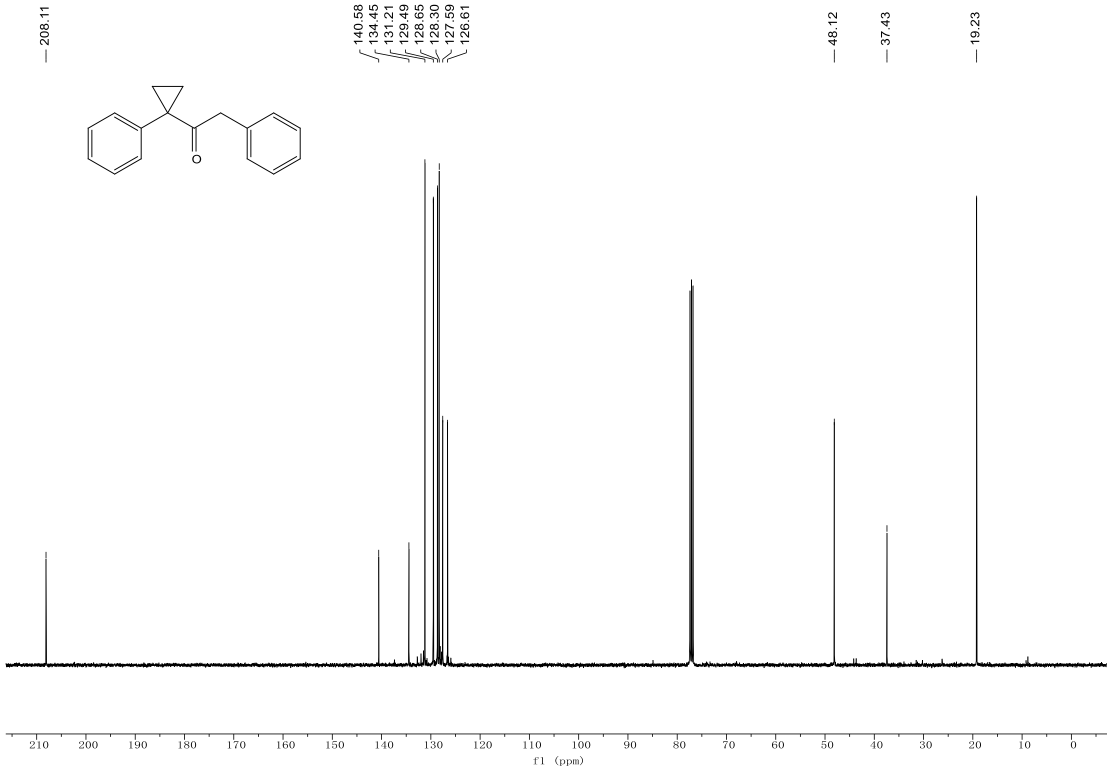

^{1}H NMR (400 MHz, $CDCl_3$) **cyclopropyl(1-phenylcyclopropyl)methanone (4)**

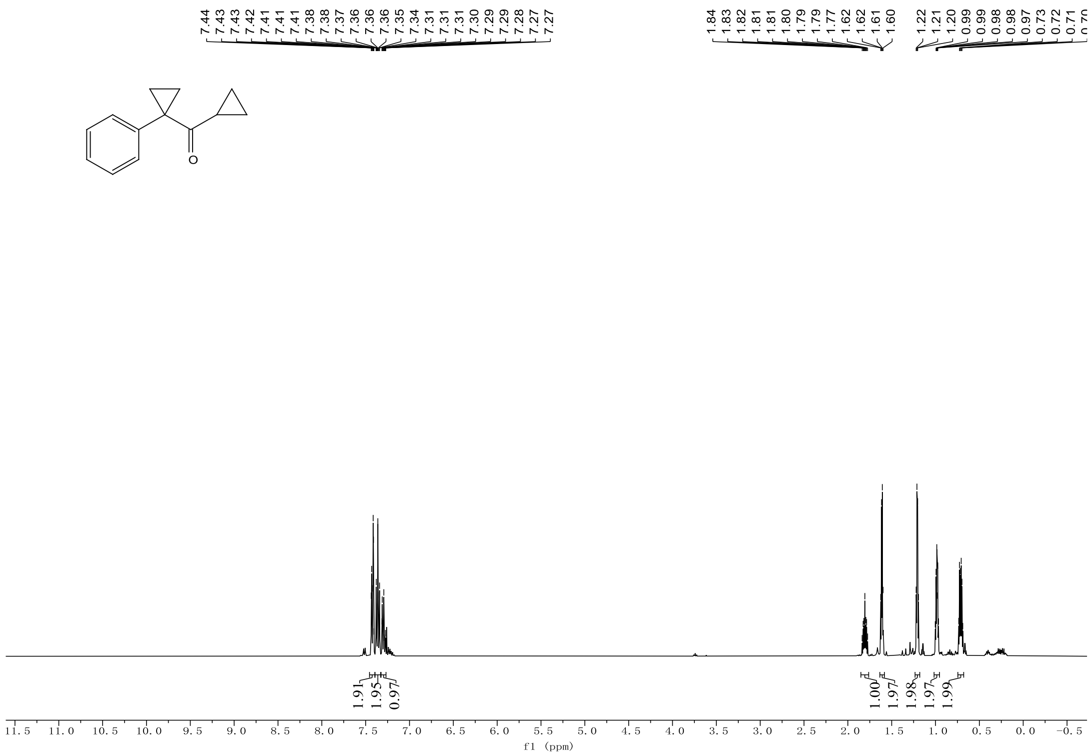

^{13}C NMR (101 MHz, $CDCl_3$) **cyclopropyl(1-phenylcyclopropyl)methanone (4)**

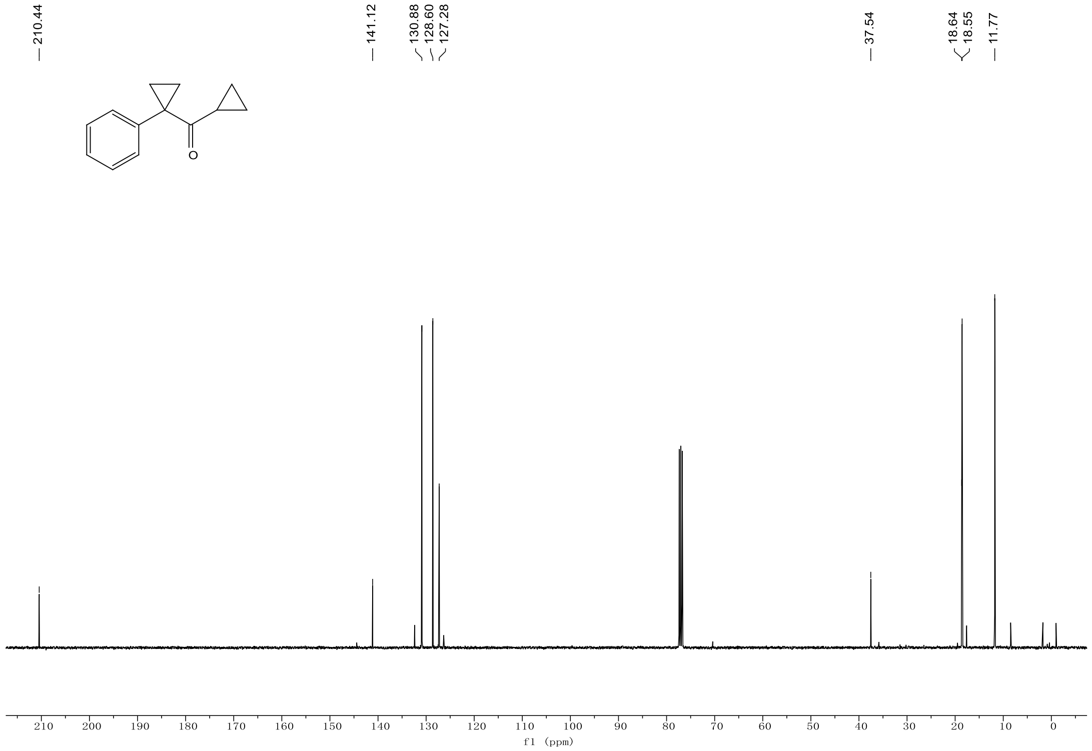

^{1}H NMR (400 MHz, $CDCl_3$) **3,3-dimethyl-1-phenoxybutan-2-one (5).**

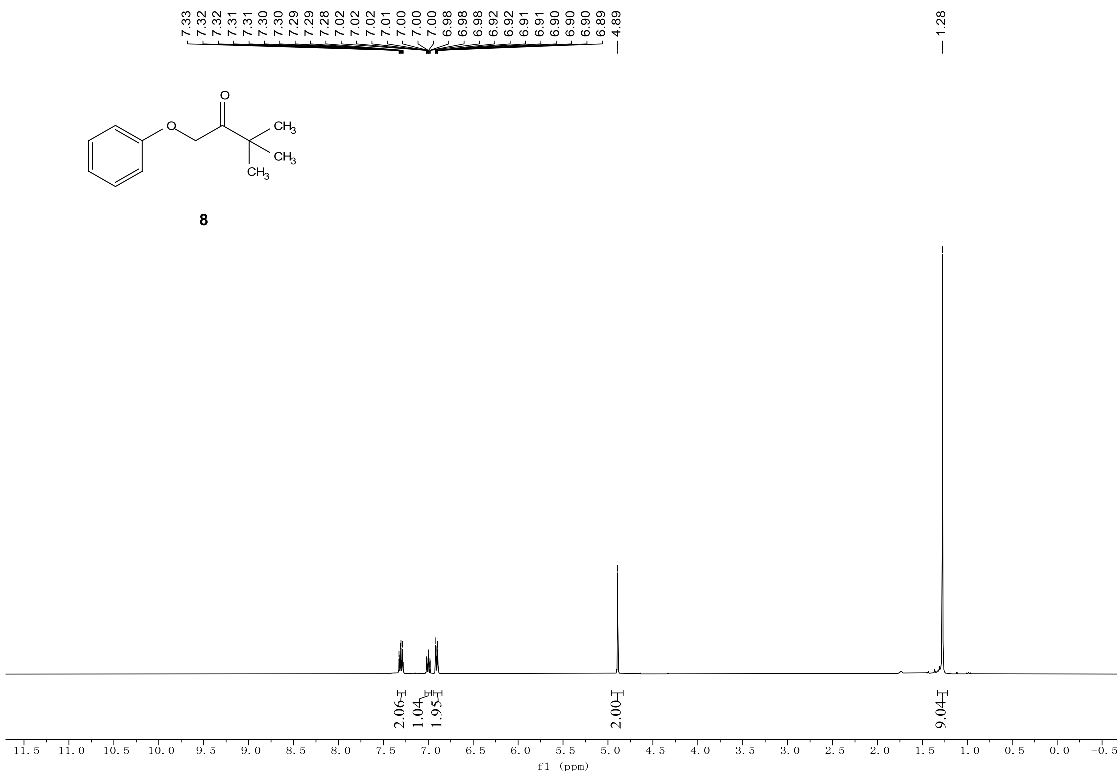

^{13}C NMR (101 MHz, $CDCl_3$) **3,3-dimethyl-1-phenoxybutan-2-one (5).**

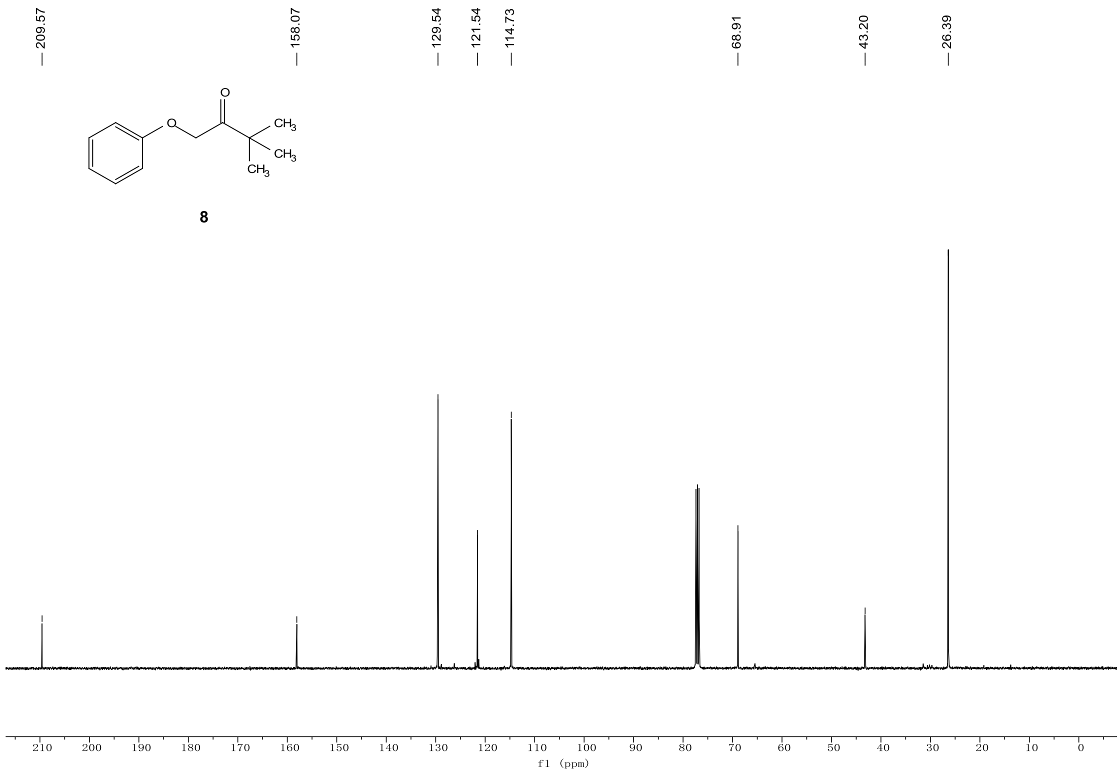

^{1}H NMR (400 MHz, $CDCl_3$) **1-(3-methyloxetan-3-yl)-2-phenoxyethan-1-one (6)**

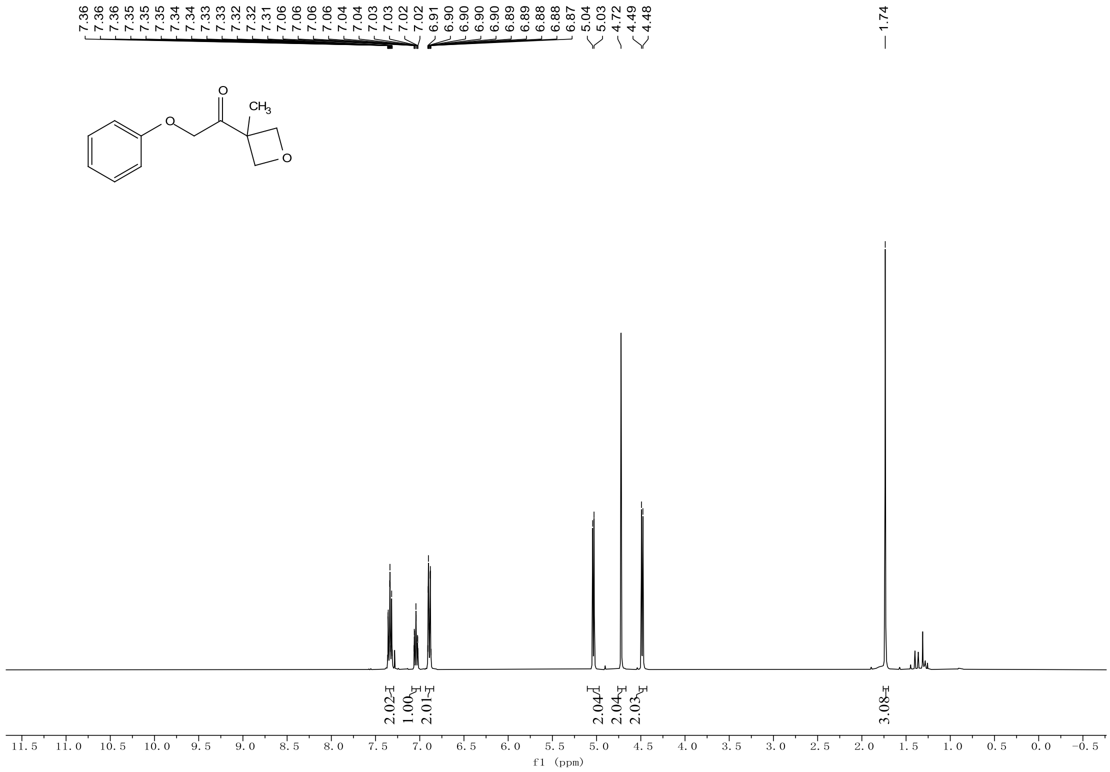

^{13}C NMR (101 MHz, $CDCl_3$) **1-(3-methyloxetan-3-yl)-2-phenoxyethan-1-one (6)**

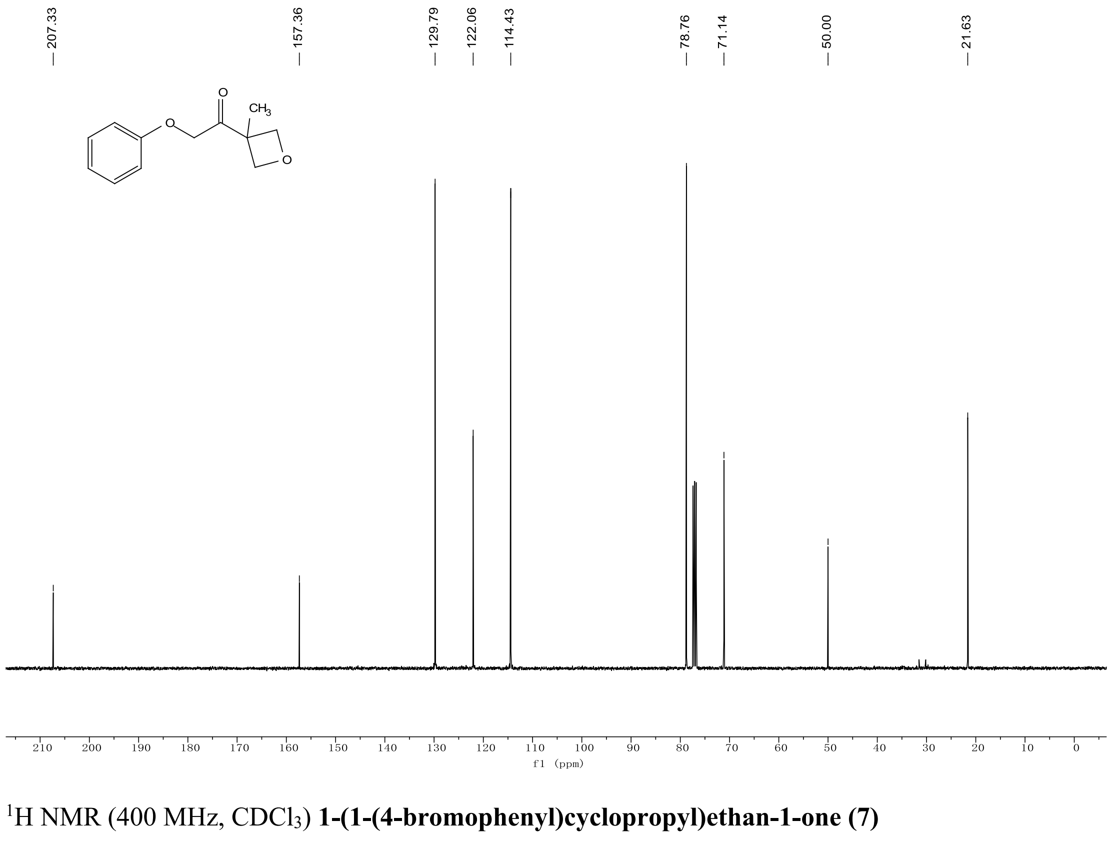

^{1}H NMR (400 MHz, $CDCl_3$) **1-(1-(4-bromophenyl)cyclopropyl)ethan-1-one (7)**

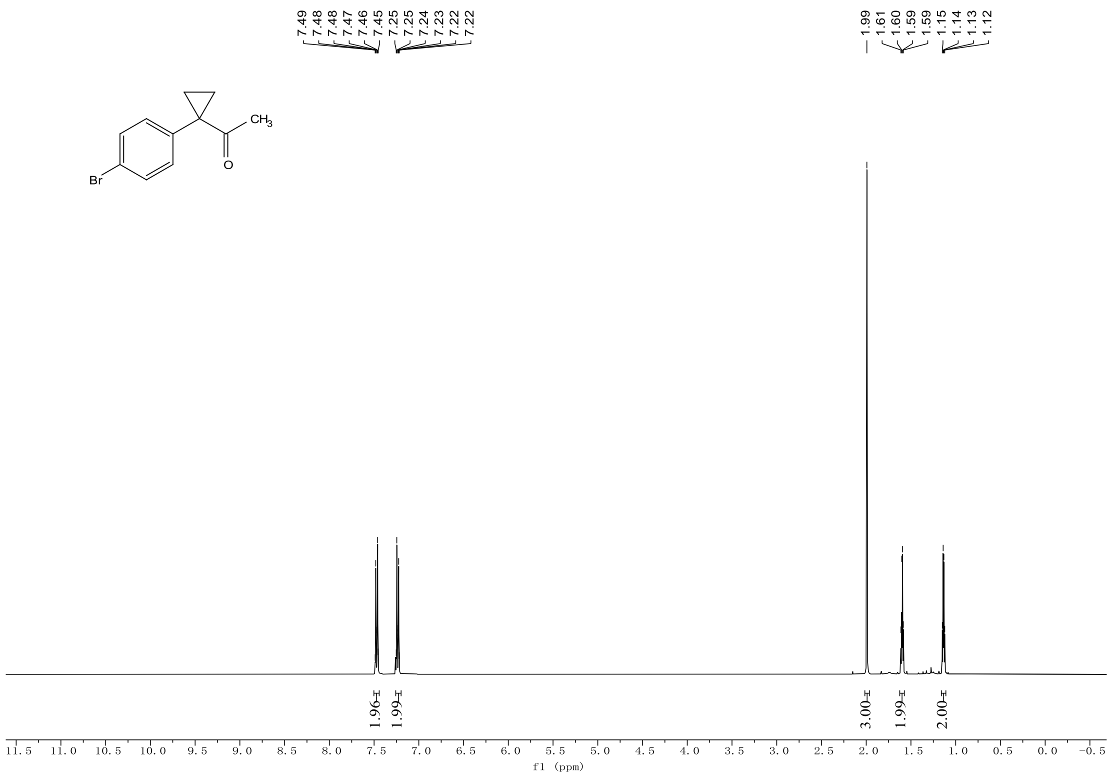

^{13}C NMR (101 MHz, $CDCl_3$) **1-(1-(4-bromophenyl)cyclopropyl)ethan-1-one (7)**

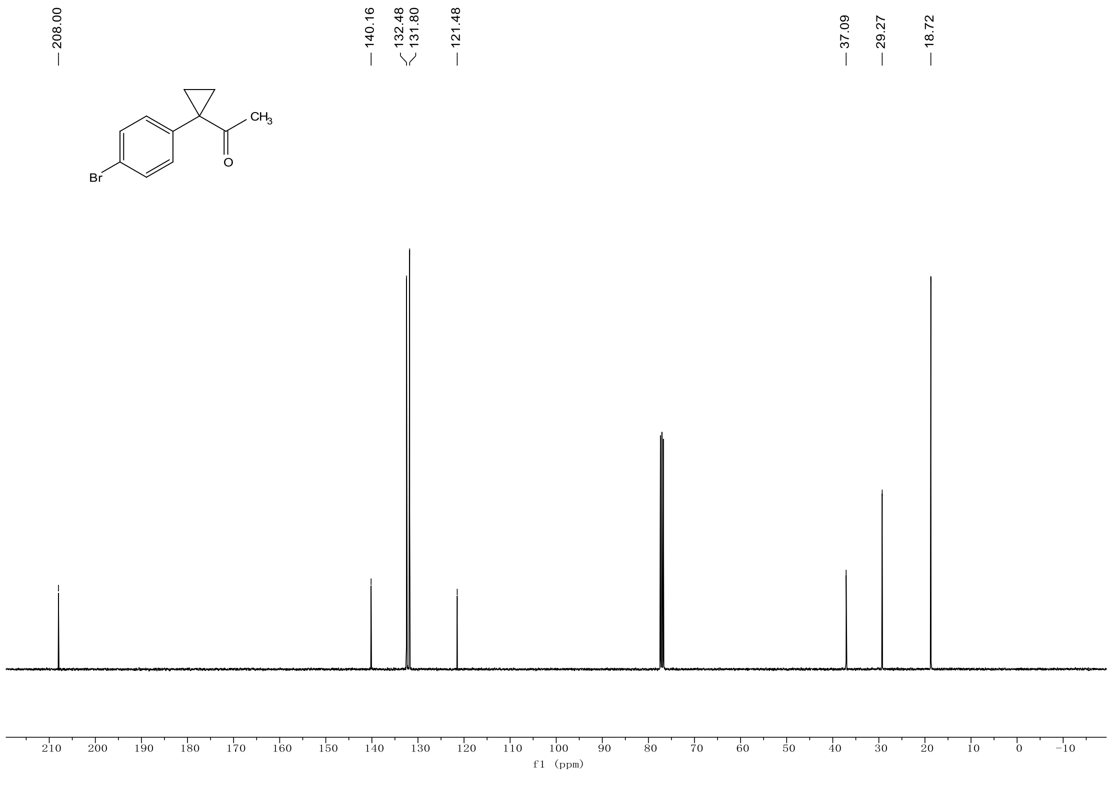

^{1}H NMR (400 MHz, $CDCl_3$) **1-(1-(4-fluorophenyl)cyclopropyl)ethan-1-one (8)**

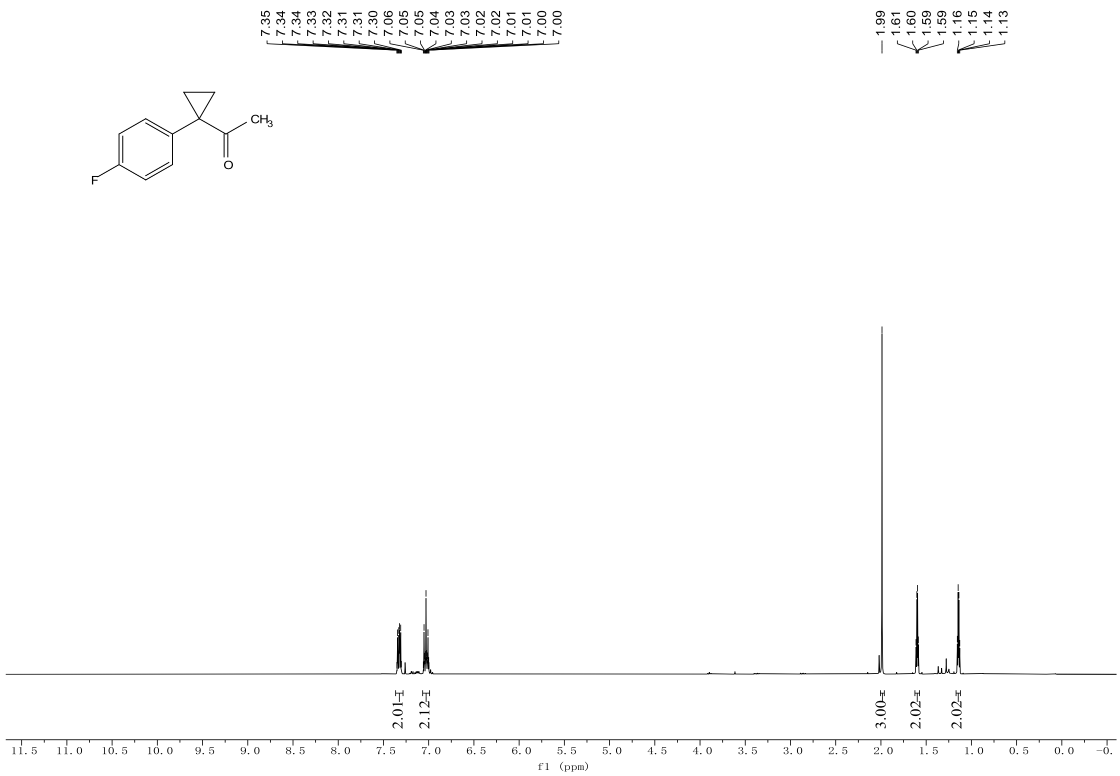

^{13}C NMR (101 MHz, $CDCl_3$) **1-(1-(4-fluorophenyl)cyclopropyl)ethan-1-one (8)**

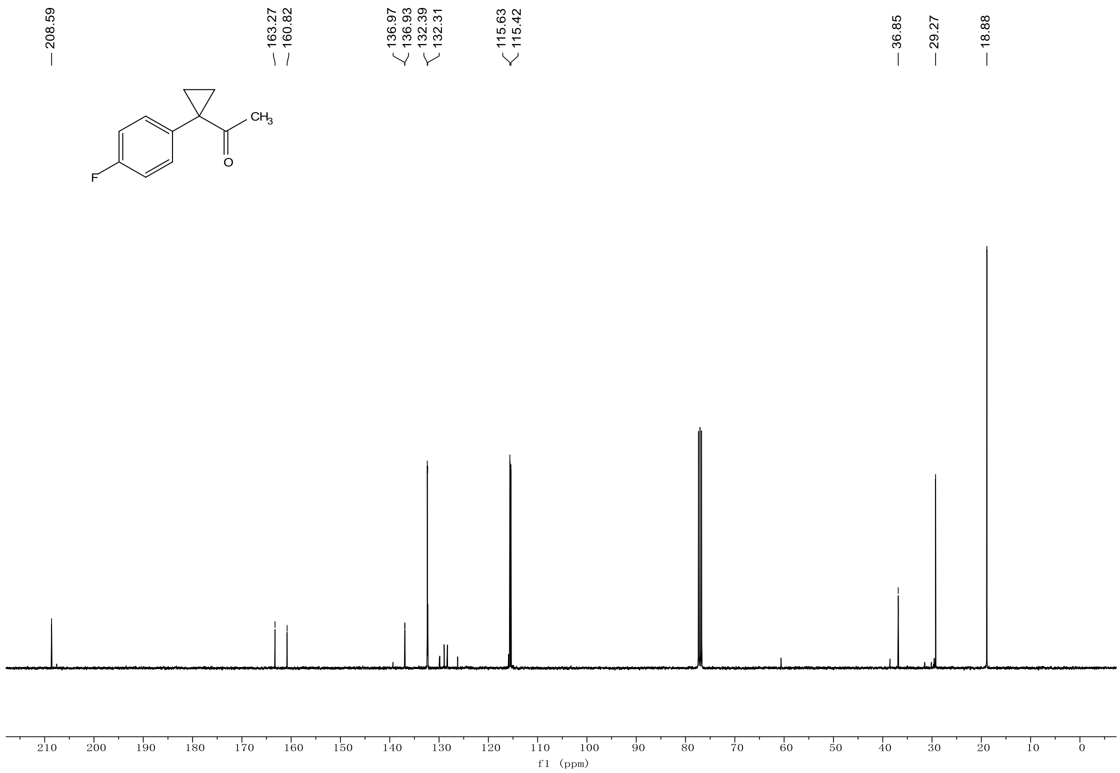

^{19}F NMR (376 MHz, $CDCl_3$) **1-(1-(4-fluorophenyl)cyclopropyl)ethan-1-one (8)**

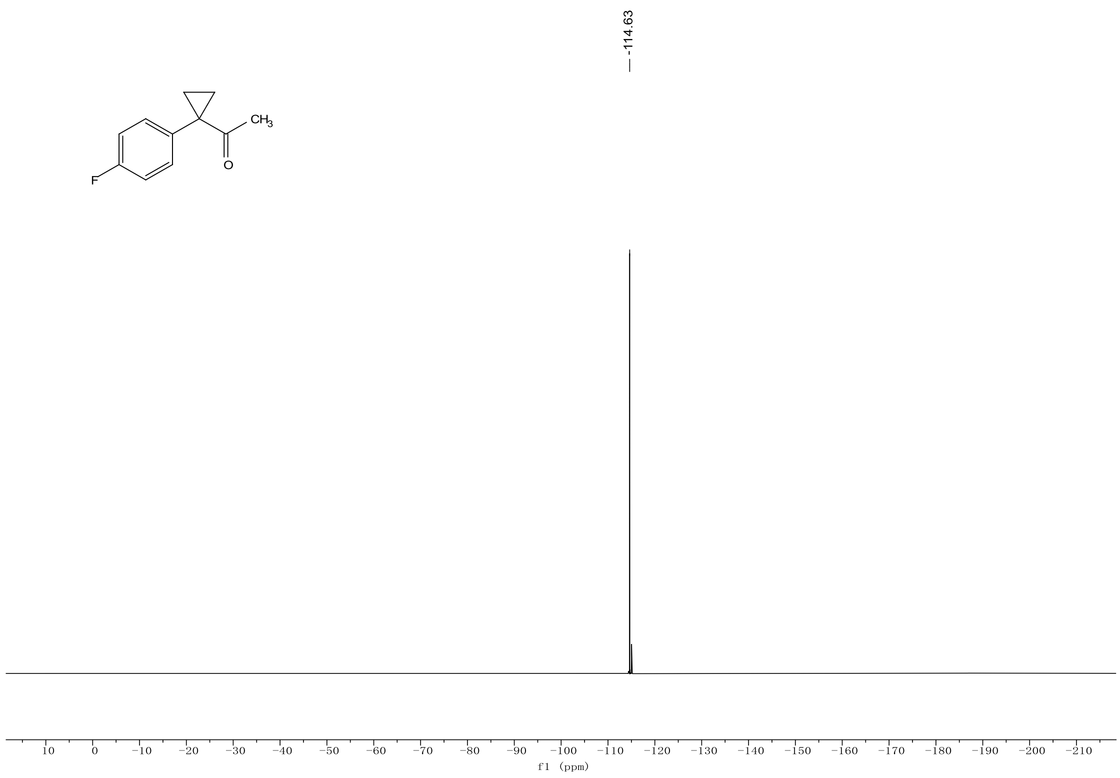

^1H NMR (400 MHz, $CDCl_3$) **1-(1-(4-methoxyphenyl)cyclopropyl)ethan-1-one (9)**

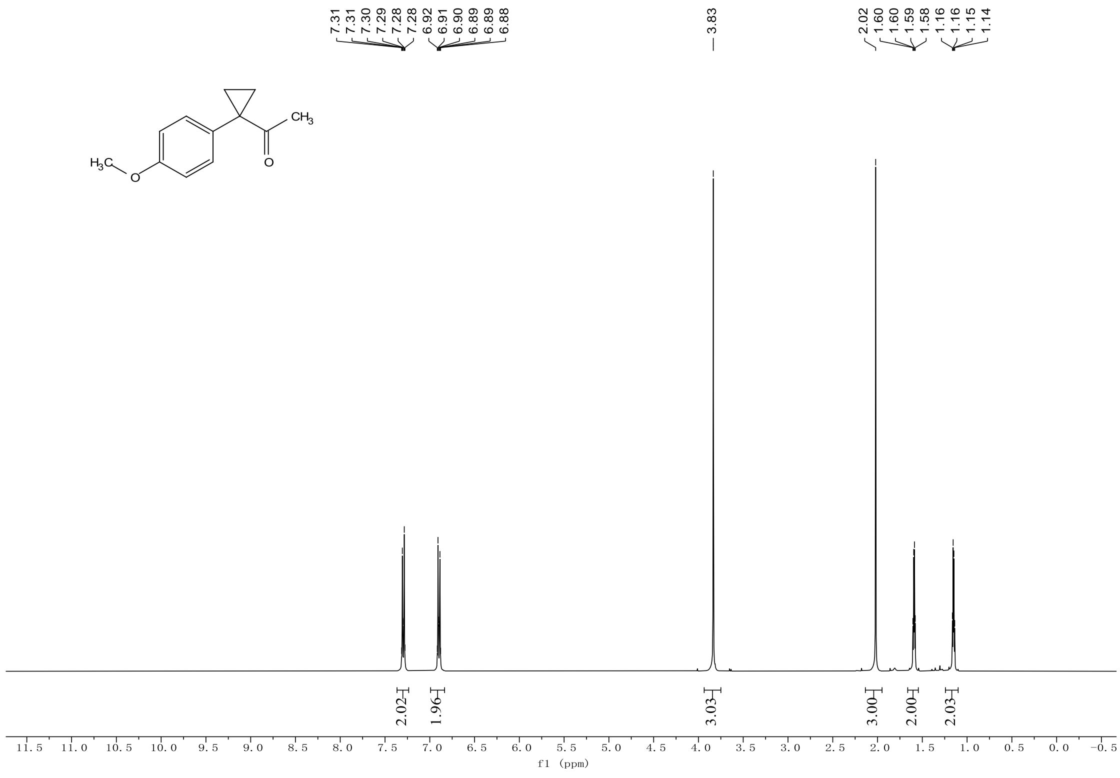

^{13}C NMR (101 MHz, $CDCl_3$) **1-(1-(4-methoxyphenyl)cyclopropyl)ethan-1-one (9)**

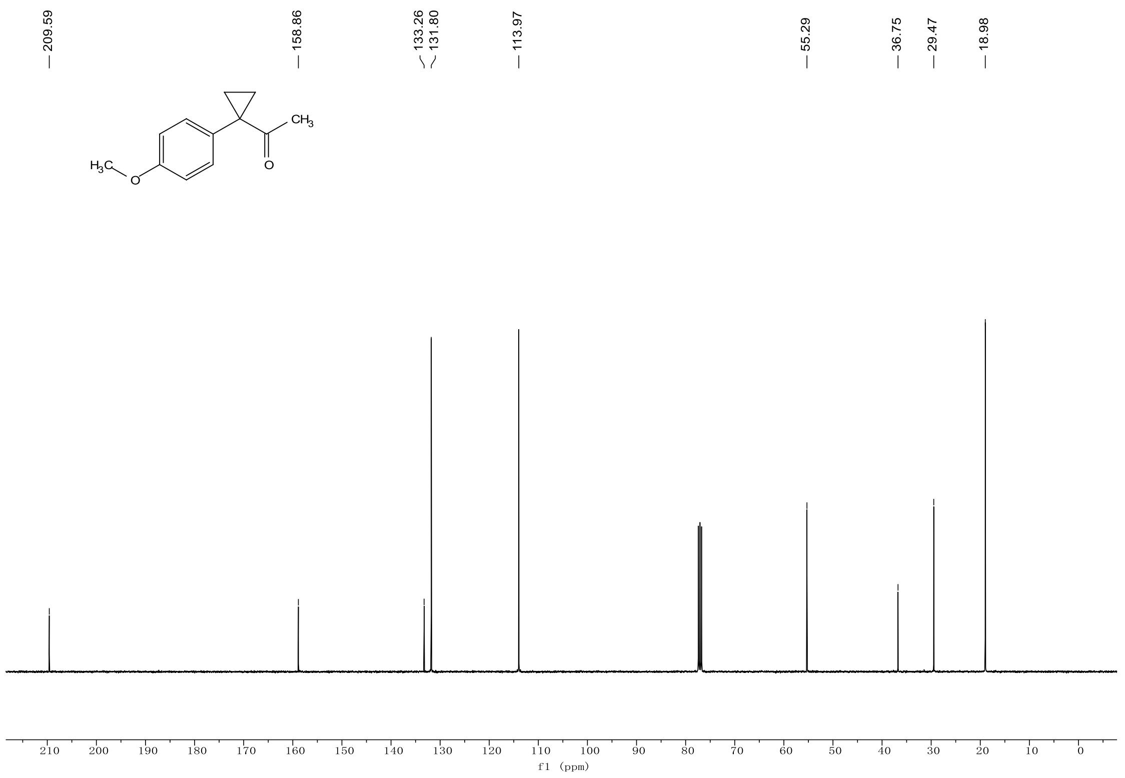

^{1}H NMR (400 MHz, $CDCl_3$) **1-(1-(2,2-difluorobenzo[*d*][1,3]dioxol-5-yl)cyclopropyl)ethan-1-one (10)**

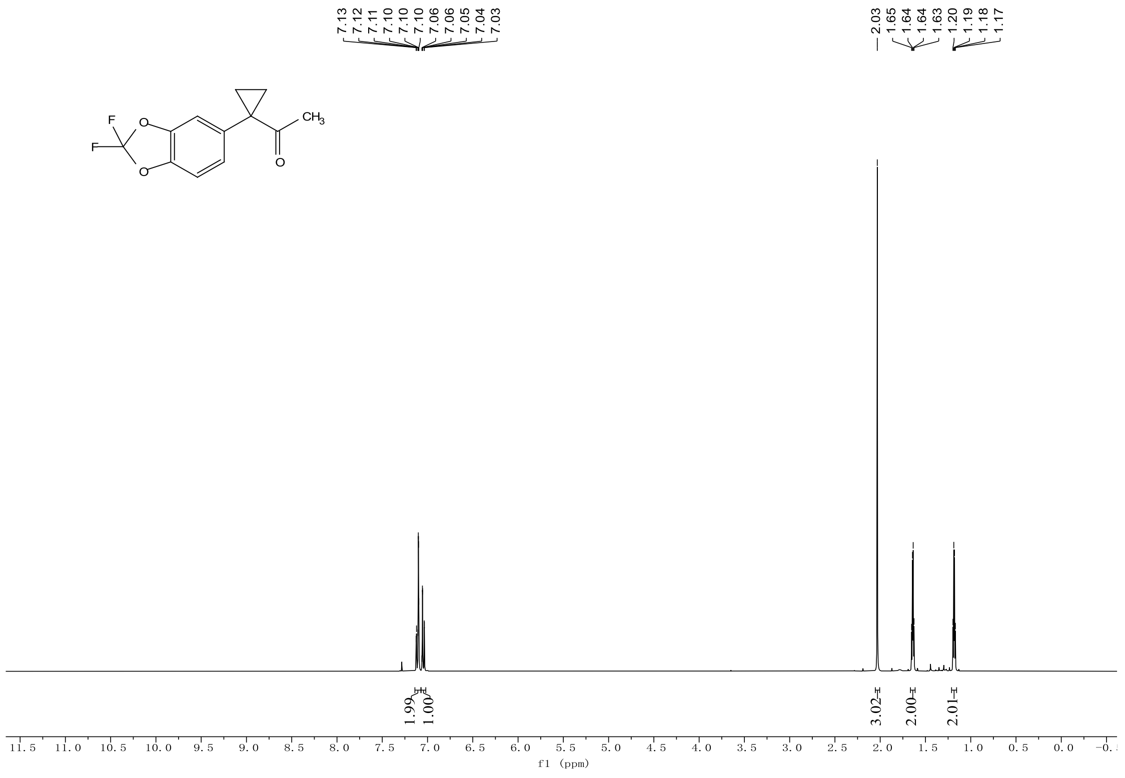

^{13}C NMR (101 MHz, $CDCl_3$) **1-(1-(2,2-difluorobenzo[*d*][1,3]dioxol-5-yl)cyclopropyl)ethan-1-one (10)**

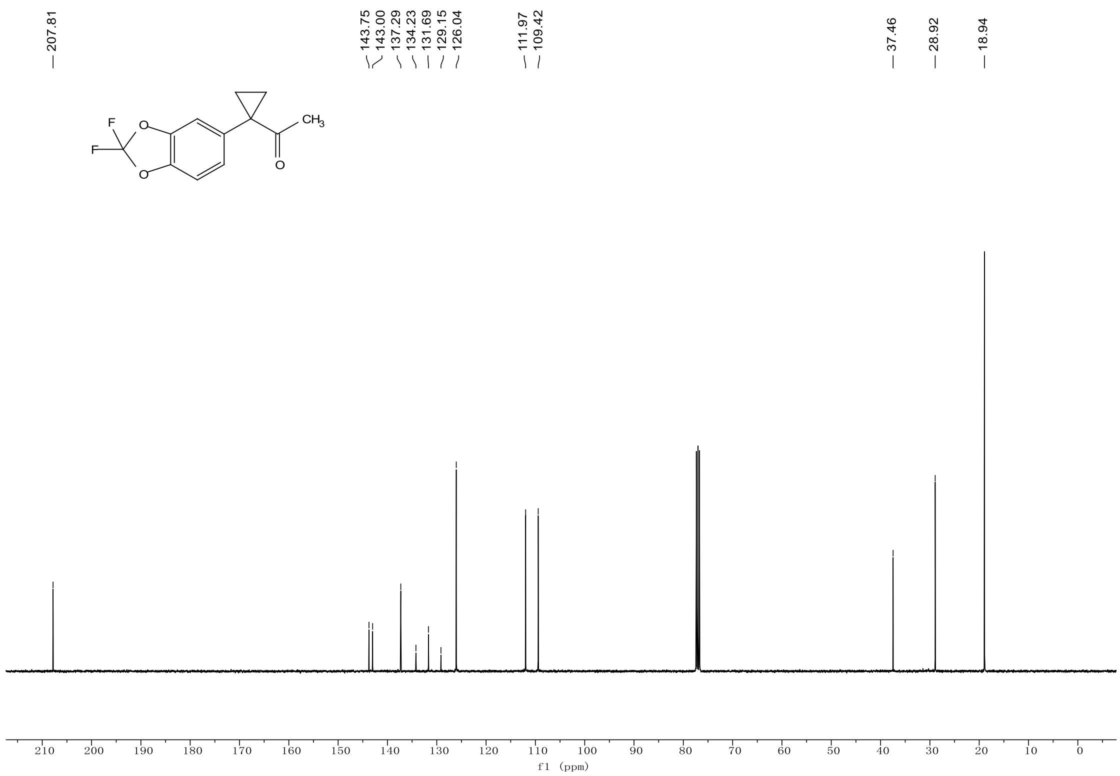

^{19}F NMR (376 MHz, $CDCl_3$) **1-(1-(2,2-difluorobenzo[*d*][1,3]dioxol-5-yl)cyclopropyl)ethan-1-one (10)**

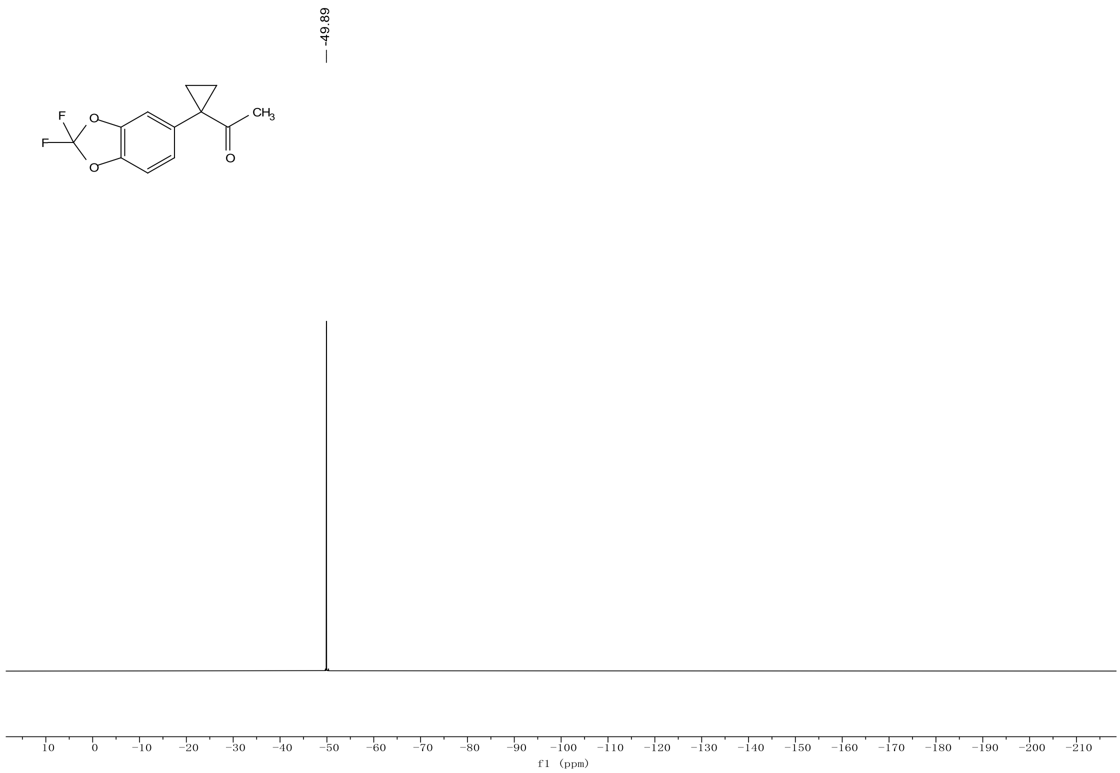

^{1}H NMR (400 MHz, $CDCl_3$) **3-methyl-3-phenoxybutan-2-one (11)**

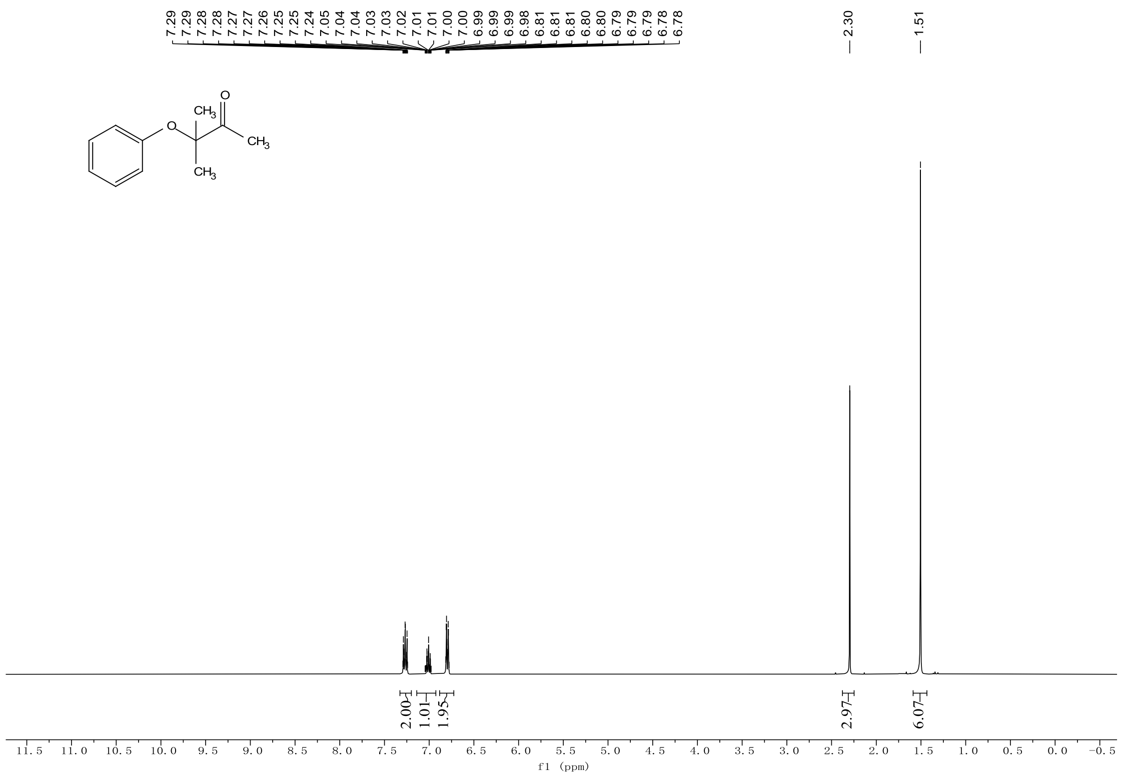

^{13}C NMR (101 MHz, $CDCl_3$) **3-methyl-3-phenoxybutan-2-one (11)**

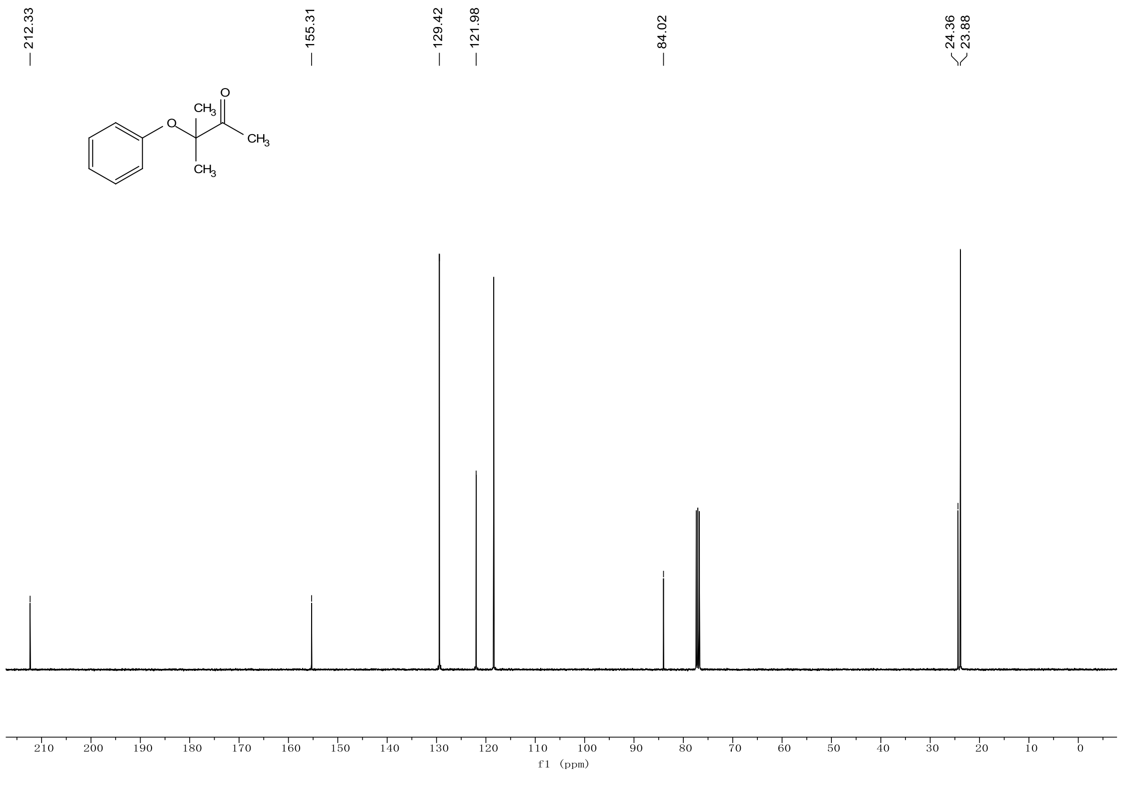

^{1}H NMR (400 MHz, $CDCl_3$) **3-(4-chlorophenoxy)-3-methylbutan-2-one (12)**

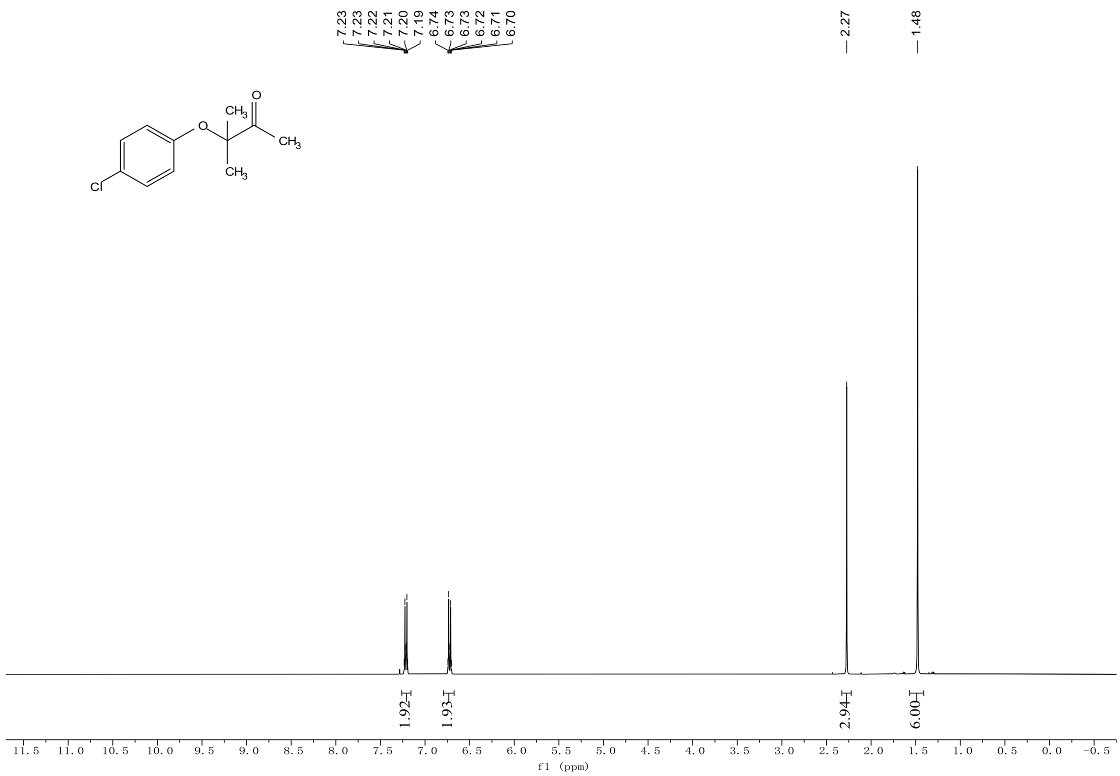

^{13}C NMR (101 MHz, $CDCl_3$) **3-(4-chlorophenoxy)-3-methylbutan-2-one (12)**

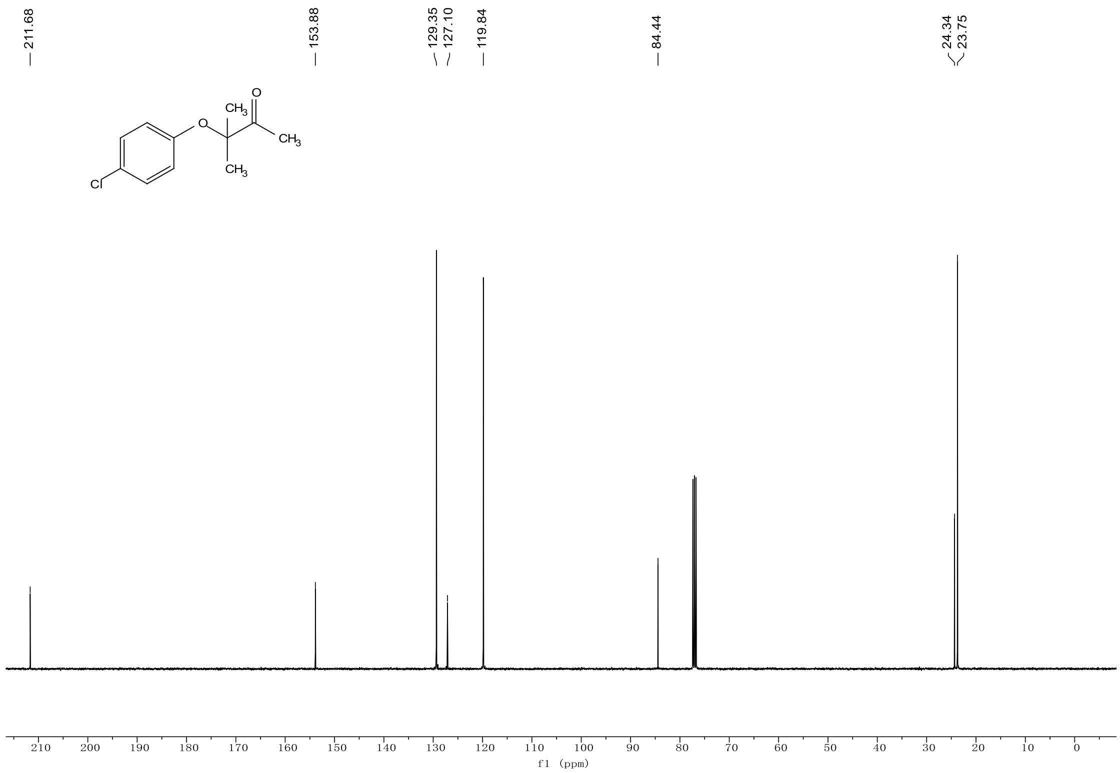

^{1}H NMR (400 MHz, $CDCl_3$) **1-(1-phenylcyclobutyl)ethan-1-one (13)**

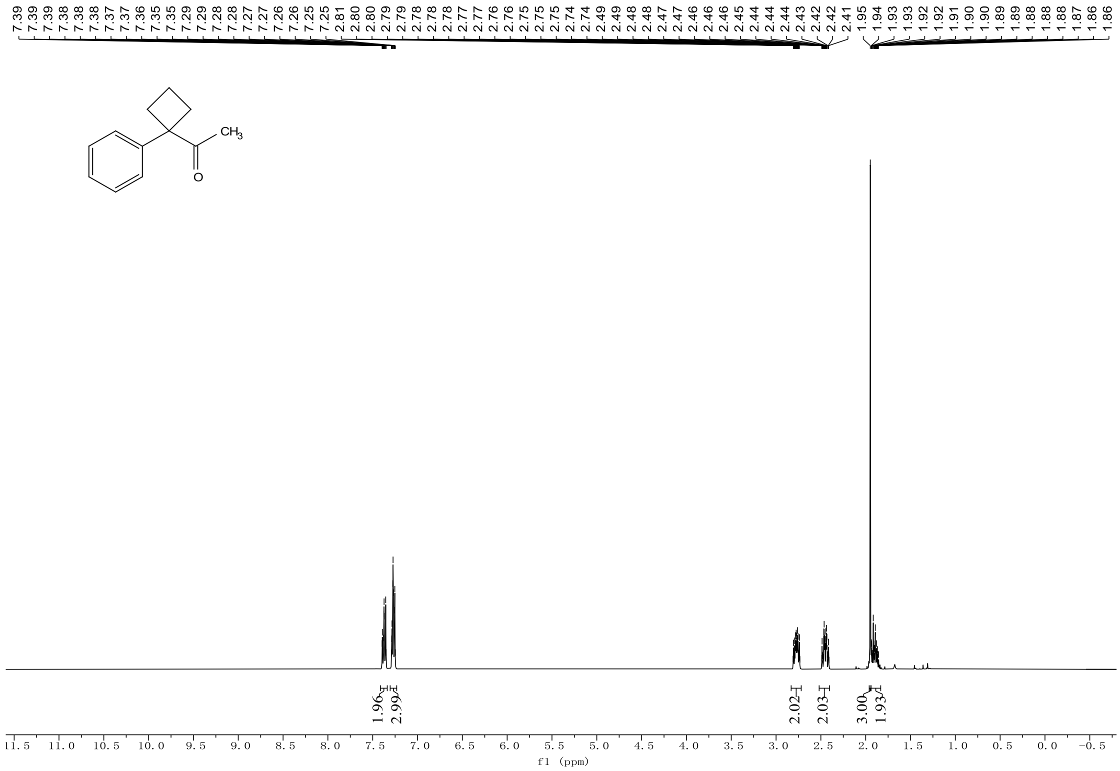

^{13}C NMR (101 MHz, $CDCl_3$) **1-(1-phenylcyclobutyl)ethan-1-one (13)**

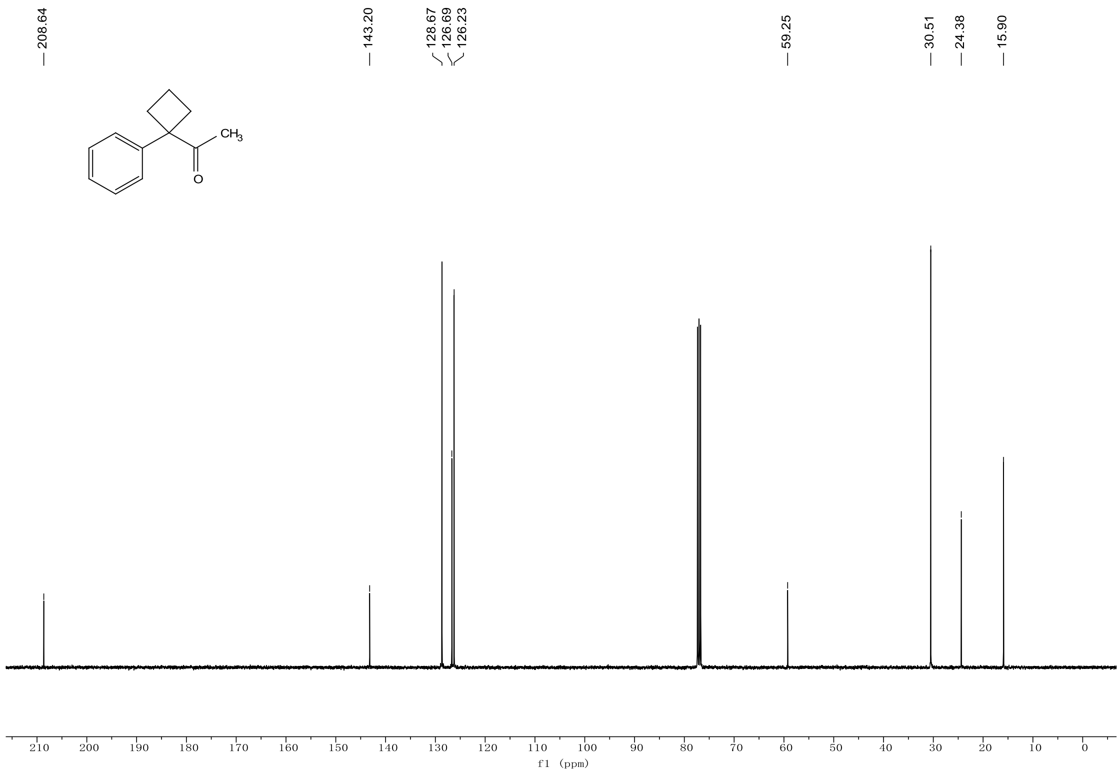

^{1}H NMR (400 MHz, $CDCl_3$) **1-(adamantan-1-yl)ethan-1-one (14)**

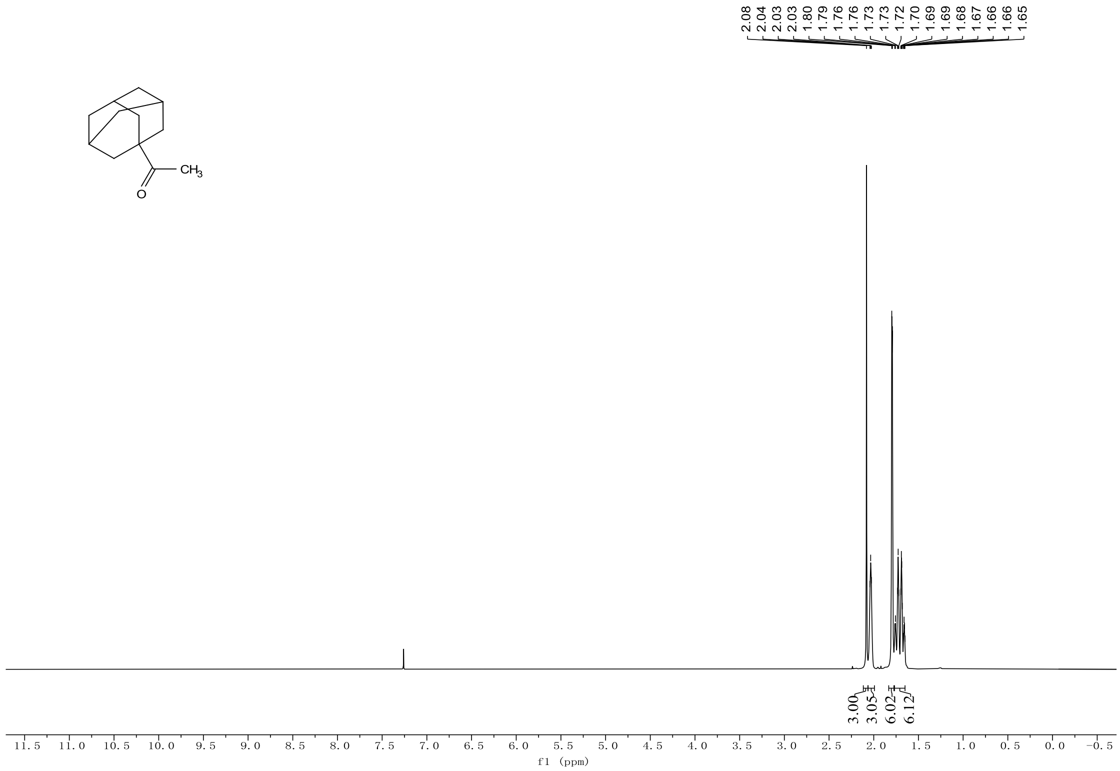

^{13}C NMR (101 MHz, $CDCl_3$) **1-(adamantan-1-yl)ethan-1-one (14)**

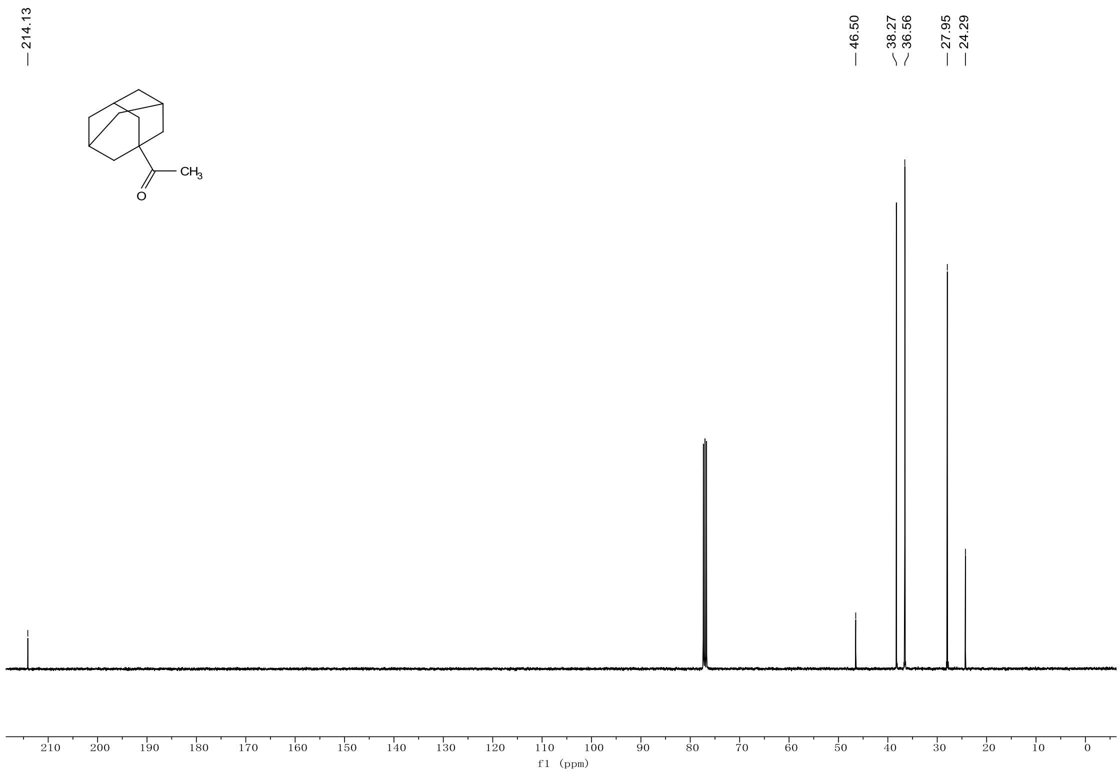

^1H NMR (400 MHz, $CDCl_3$) **methyl 4-acetylbicyclo[2.2.2]octane-1-carboxylate (15)**

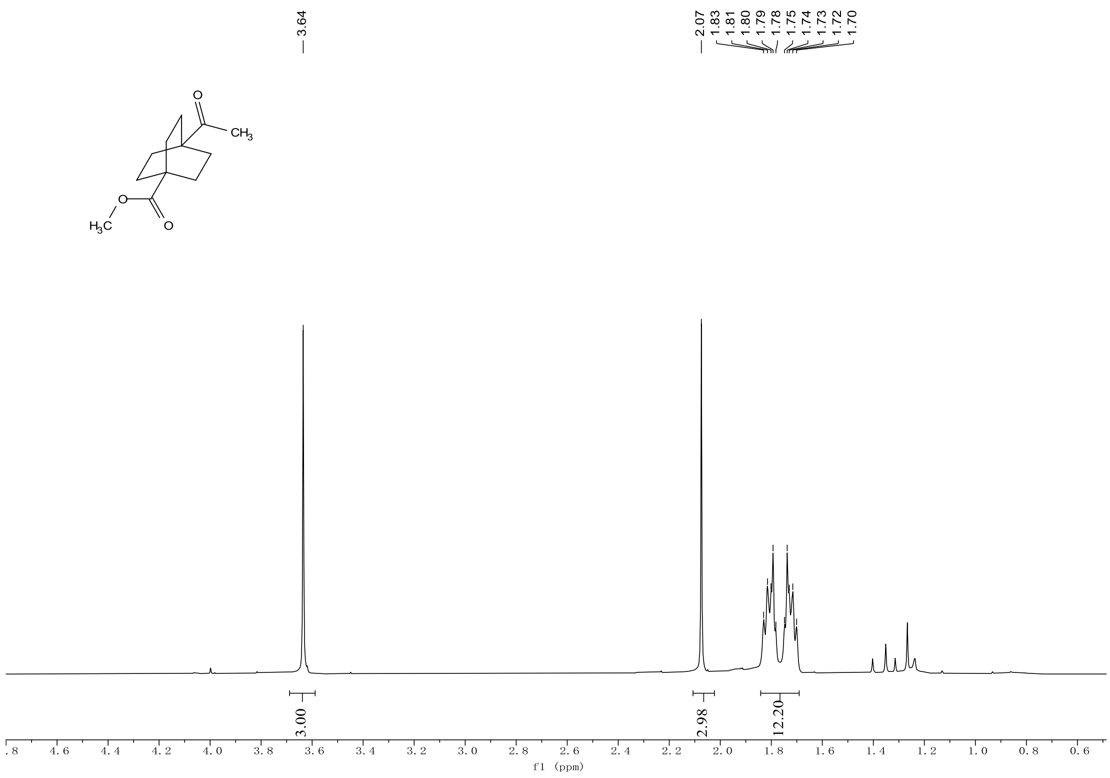

^{13}C NMR (101 MHz, $CDCl_3$) **methyl 4-acetylbicyclo[2.2.2]octane-1-carboxylate (15)**

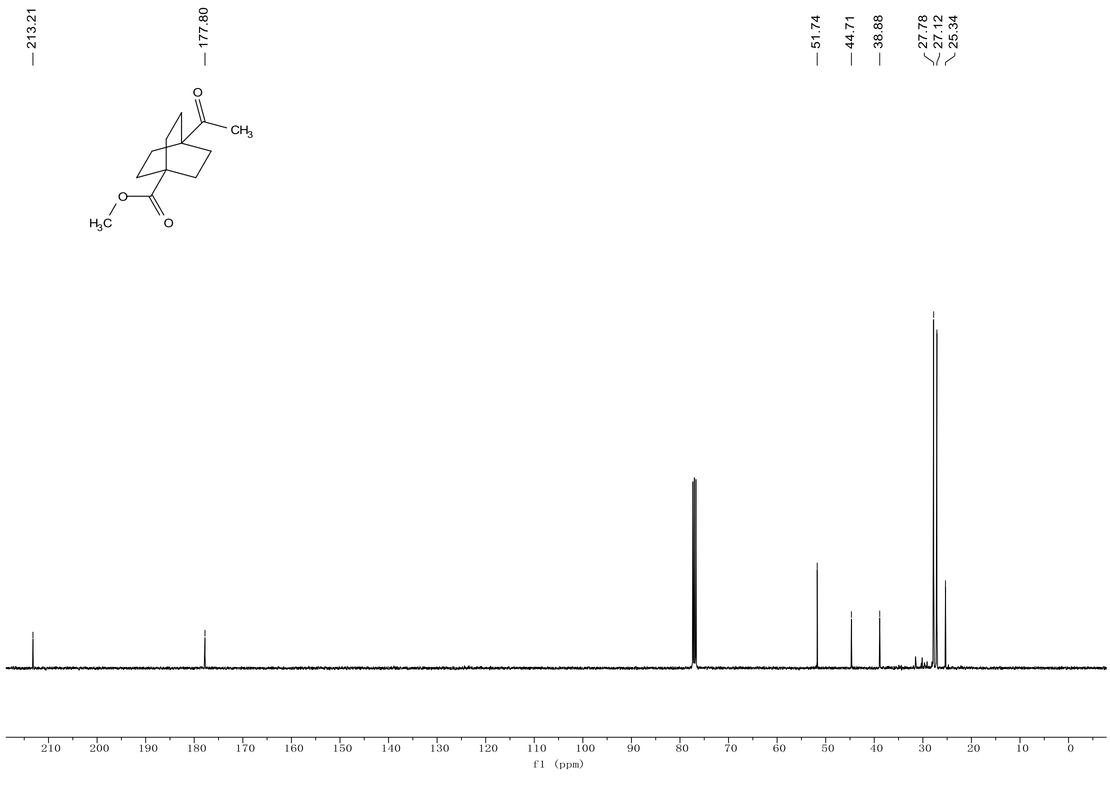

^{1}H NMR (400 MHz, $CDCl_3$) **methyl 3-acetylbicyclo[1.1.1]pentane-1-carboxylate (16)**

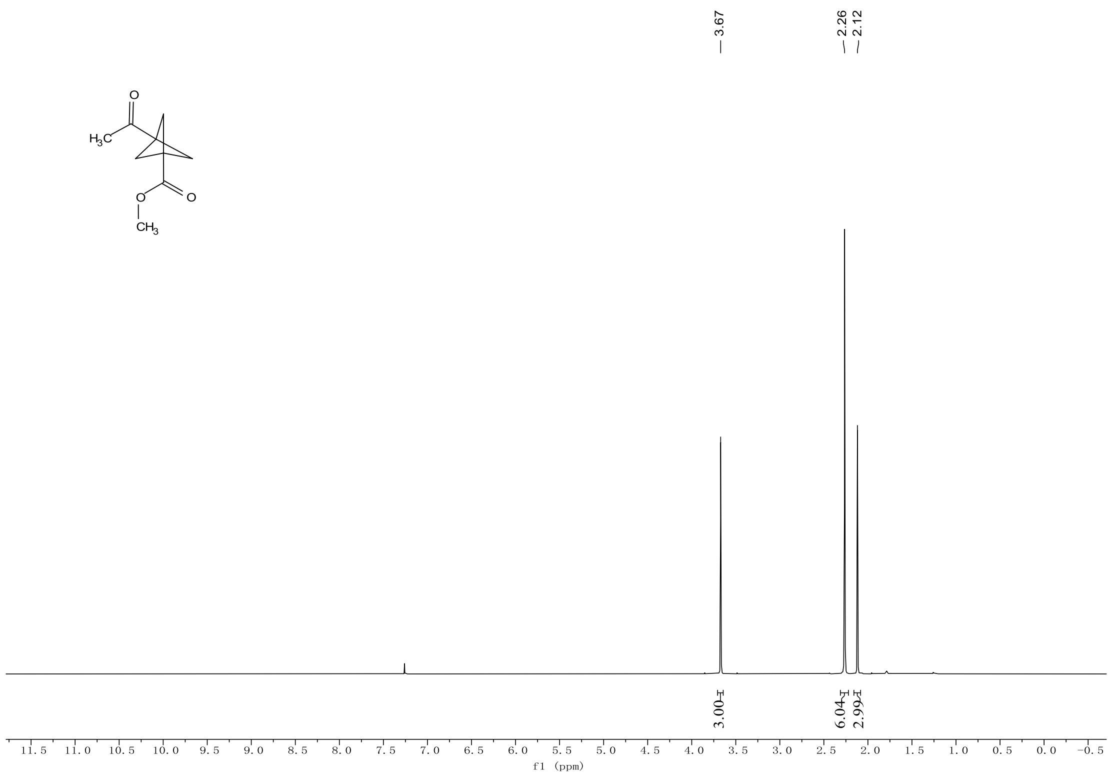

^{13}C NMR (101 MHz, $CDCl_3$) **methyl 3-acetylbicyclo[1.1.1]pentane-1-carboxylate (16)**

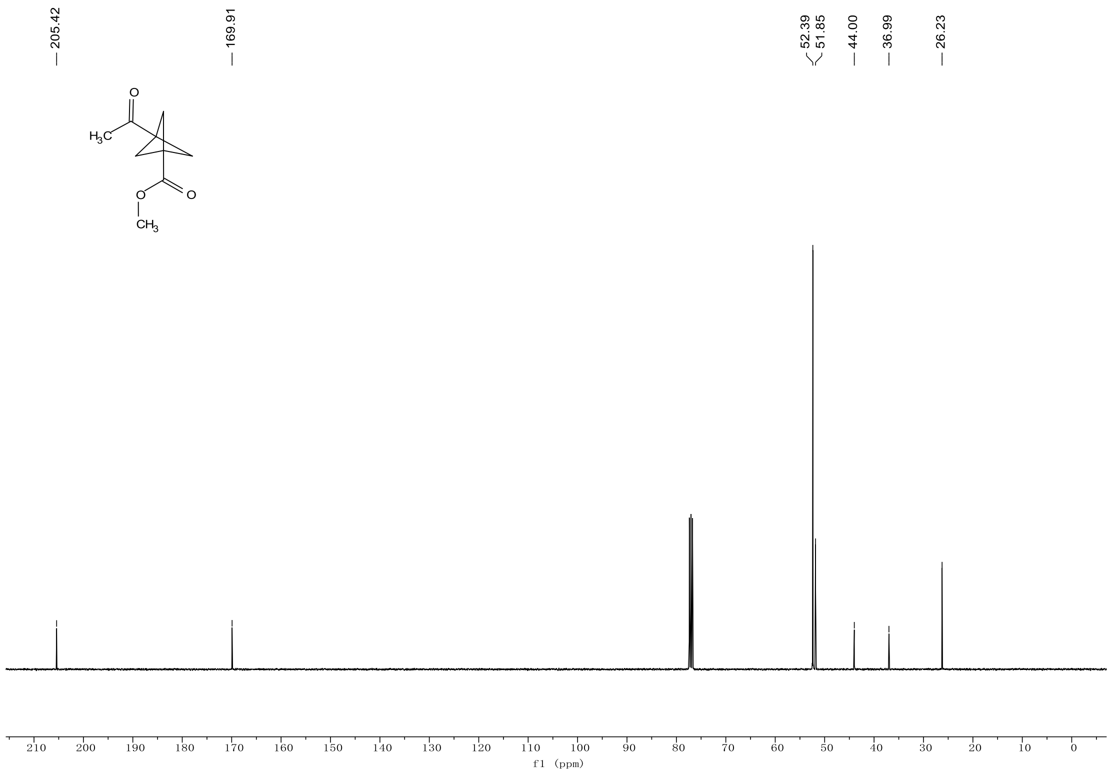

^{1}H NMR (400 MHz, $CDCl_3$) **1-phenoxypropan-2-one (19)**

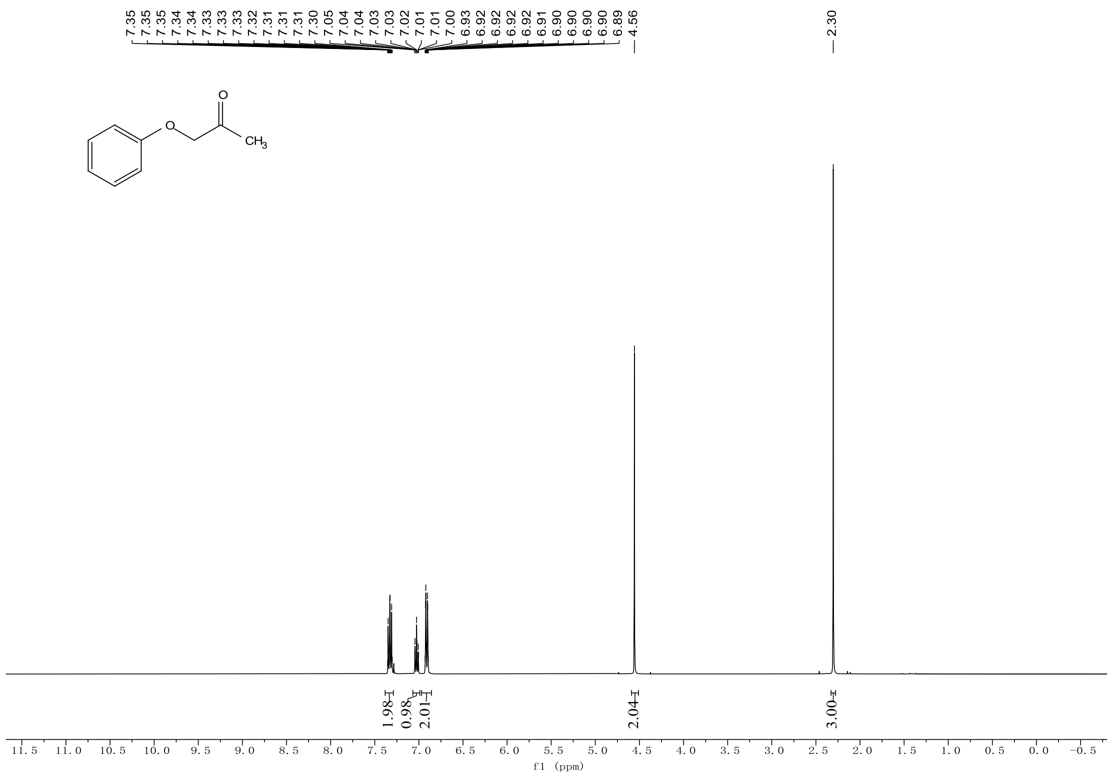

^{13}C NMR (101 MHz, $CDCl_3$) **1-phenoxypropan-2-one (19)**

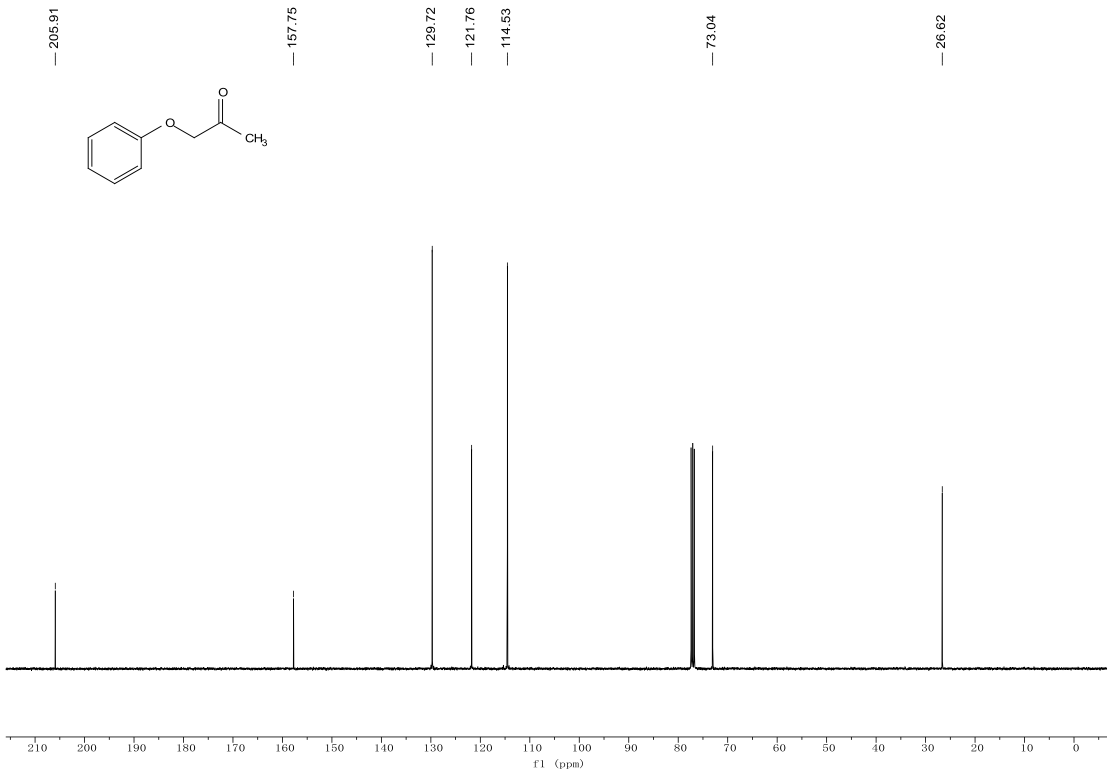

^{1}H NMR (400 MHz, $CDCl_3$) **1-phenoxybutan-2-one (20)**

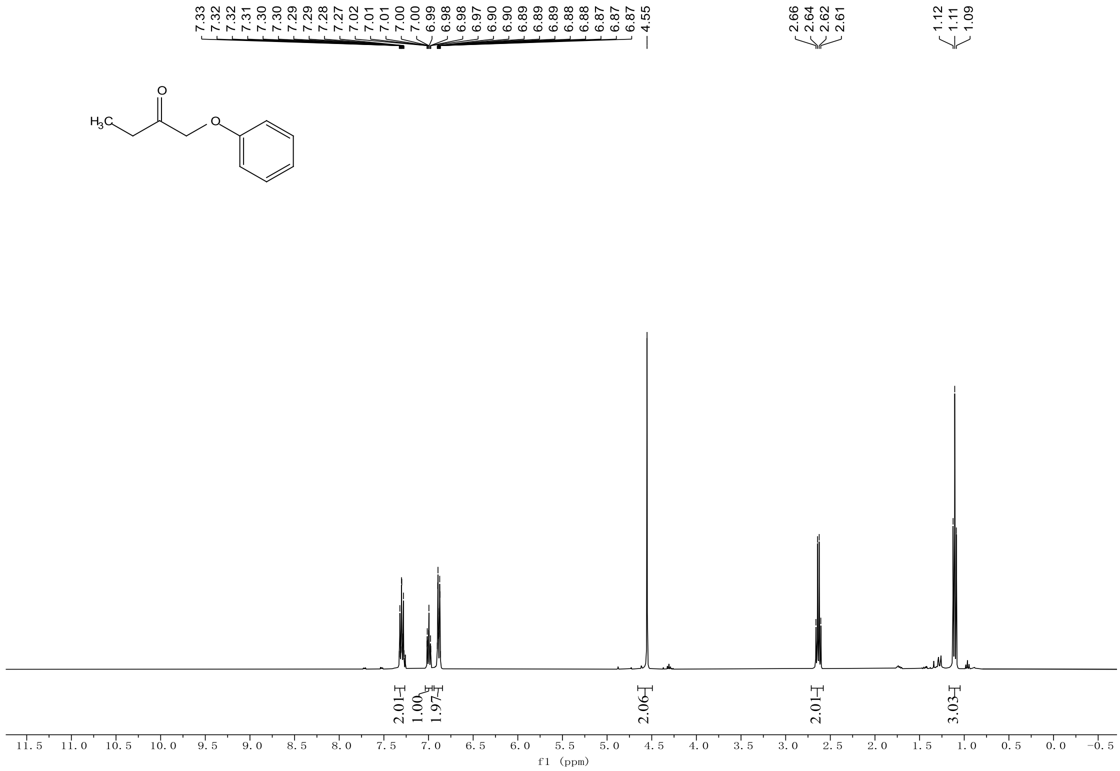

^{13}C NMR (101 MHz, $CDCl_3$) **1-phenoxybutan-2-one (20)**

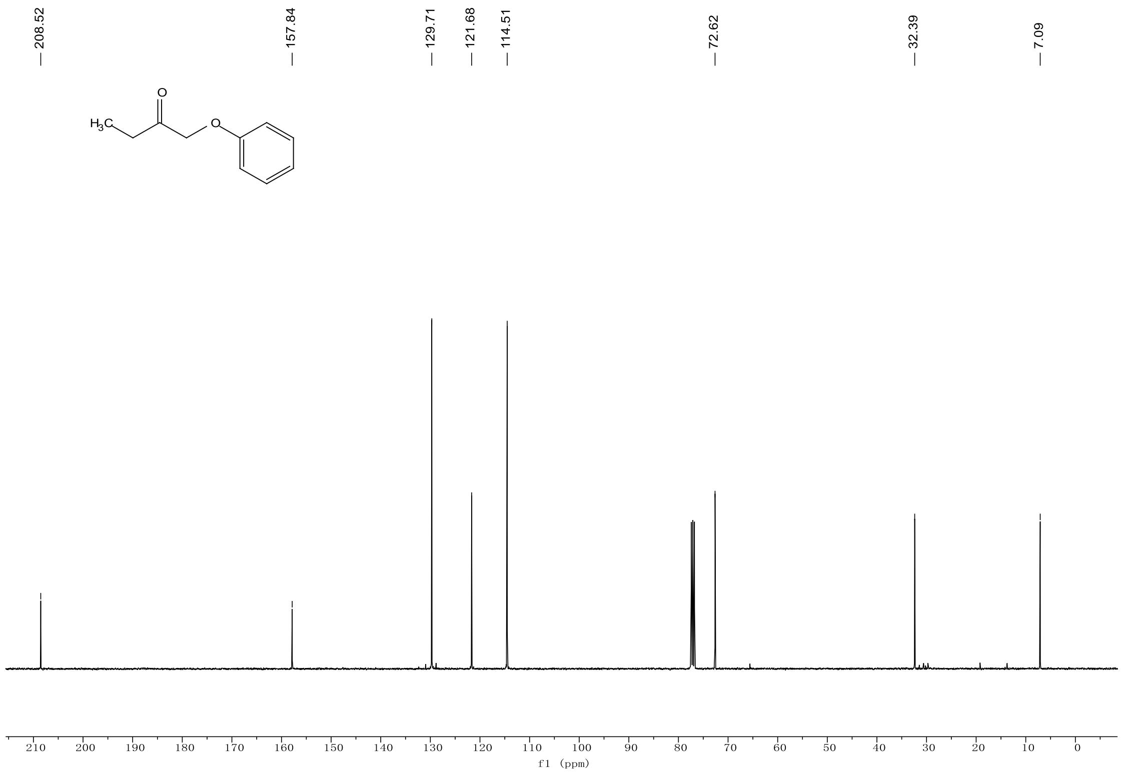

^{1}H NMR (400 MHz, $CDCl_3$) **1-phenoxypentan-2-one (21)**

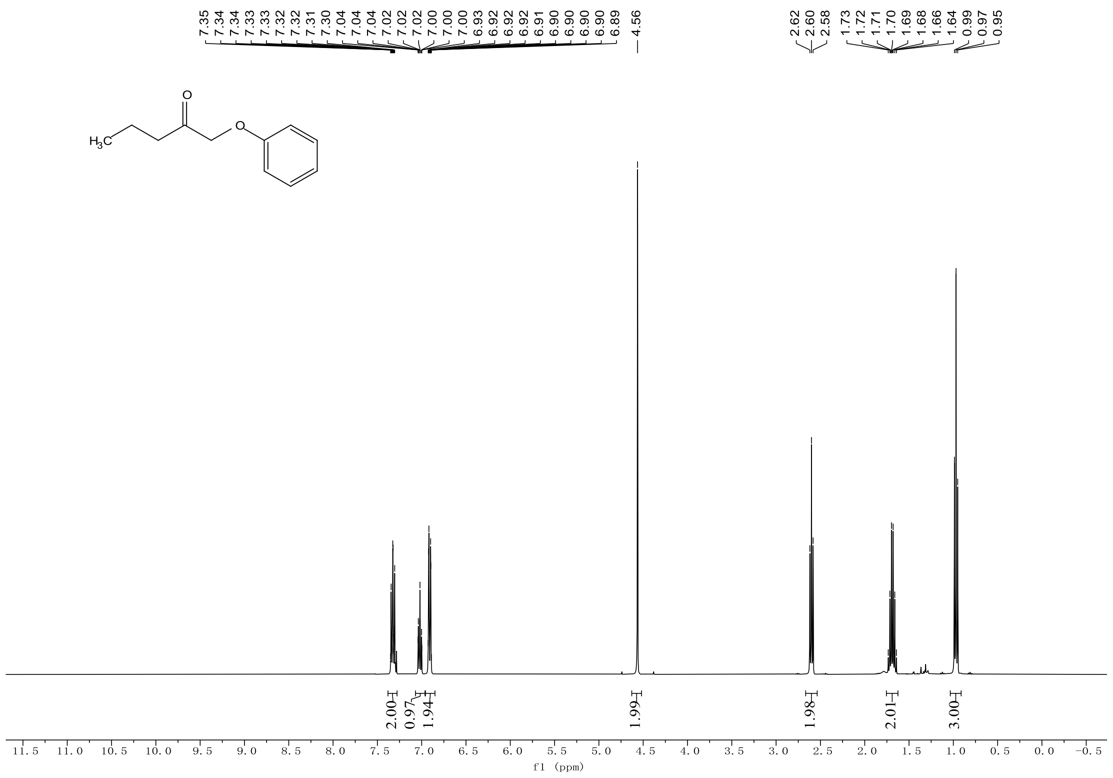

^{13}C NMR (101 MHz, $CDCl_3$) **1-phenoxypentan-2-one (21)**

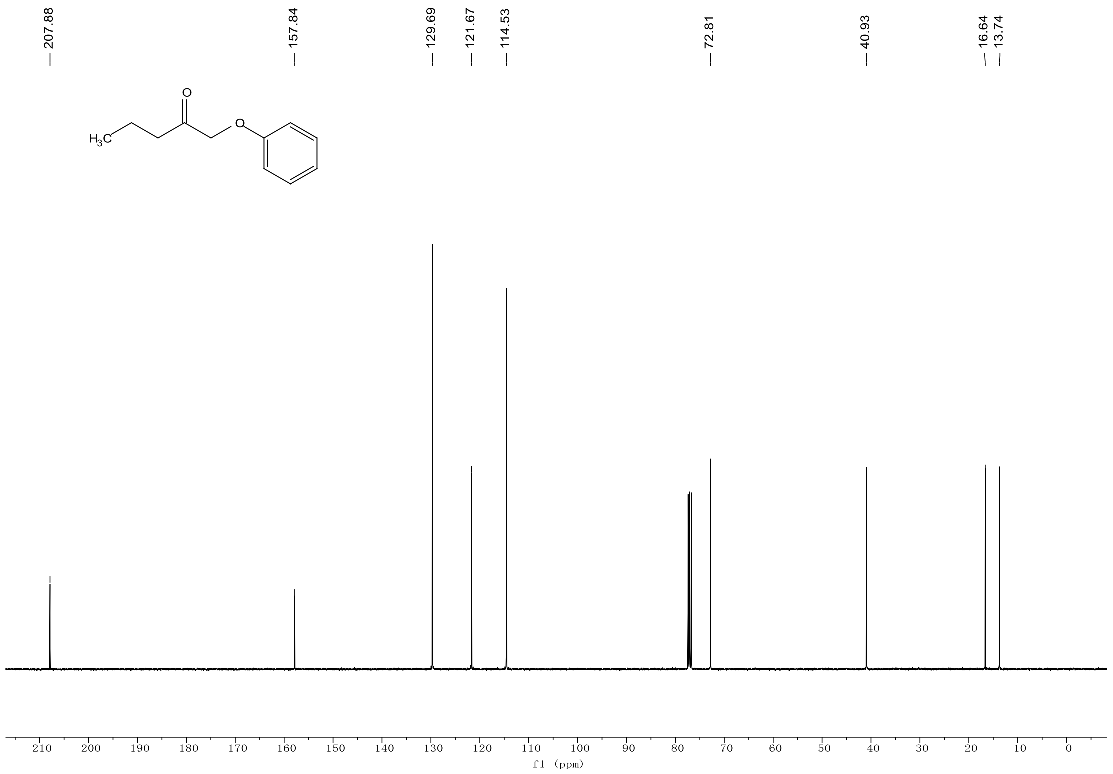

^{1}H NMR (400 MHz, $CDCl_3$) **1-phenoxyhexan-2-one (22)**

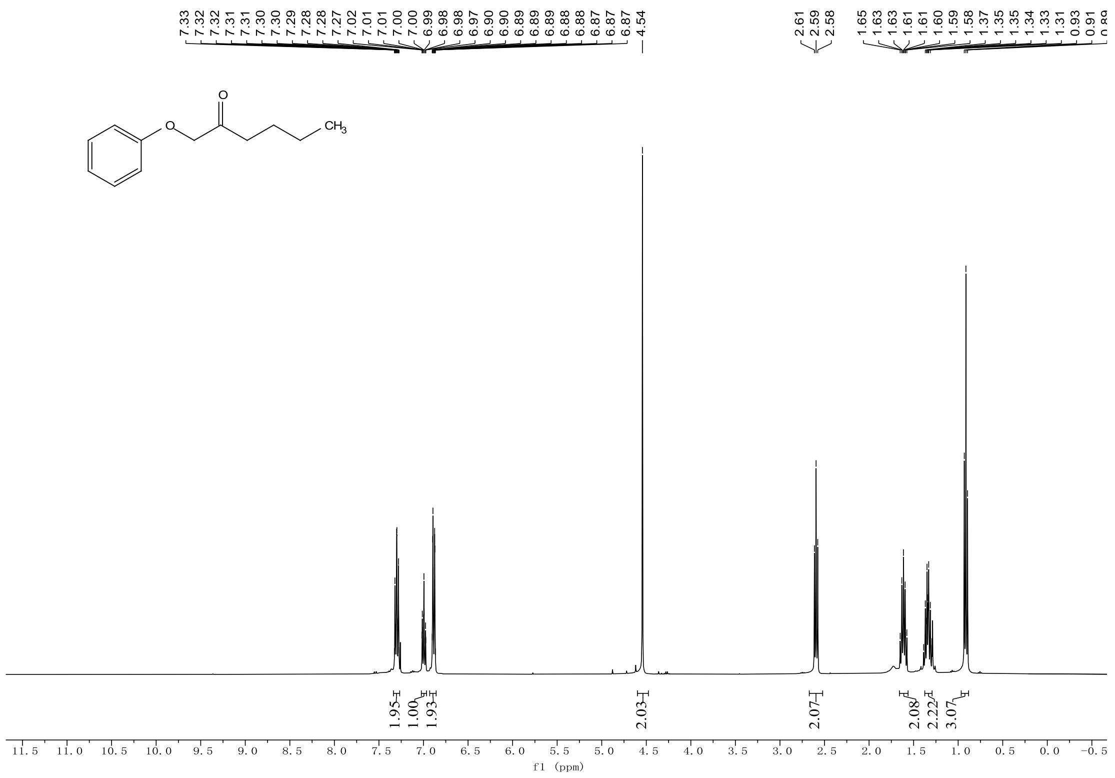

^{13}C NMR (101 MHz, $CDCl_3$) **1-phenoxyhexan-2-one (22)**

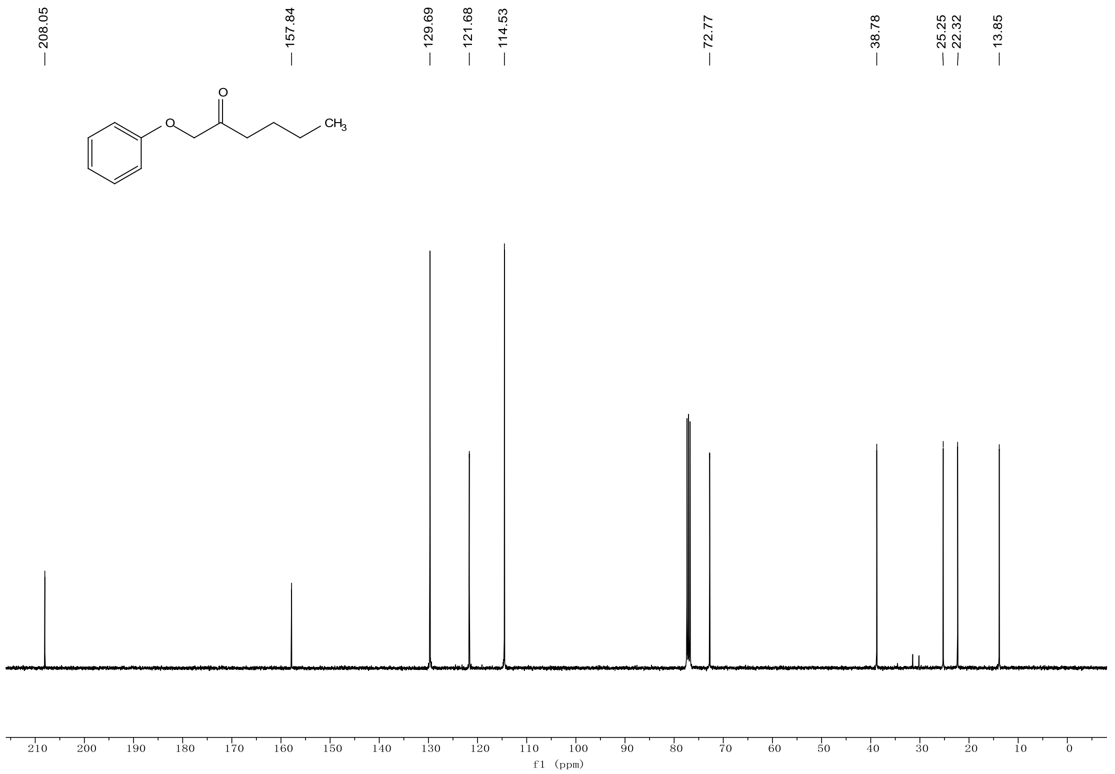

^{1}H NMR (400 MHz, $CDCl_3$) **4-methyl-1-phenoxypentan-2-one (23)**

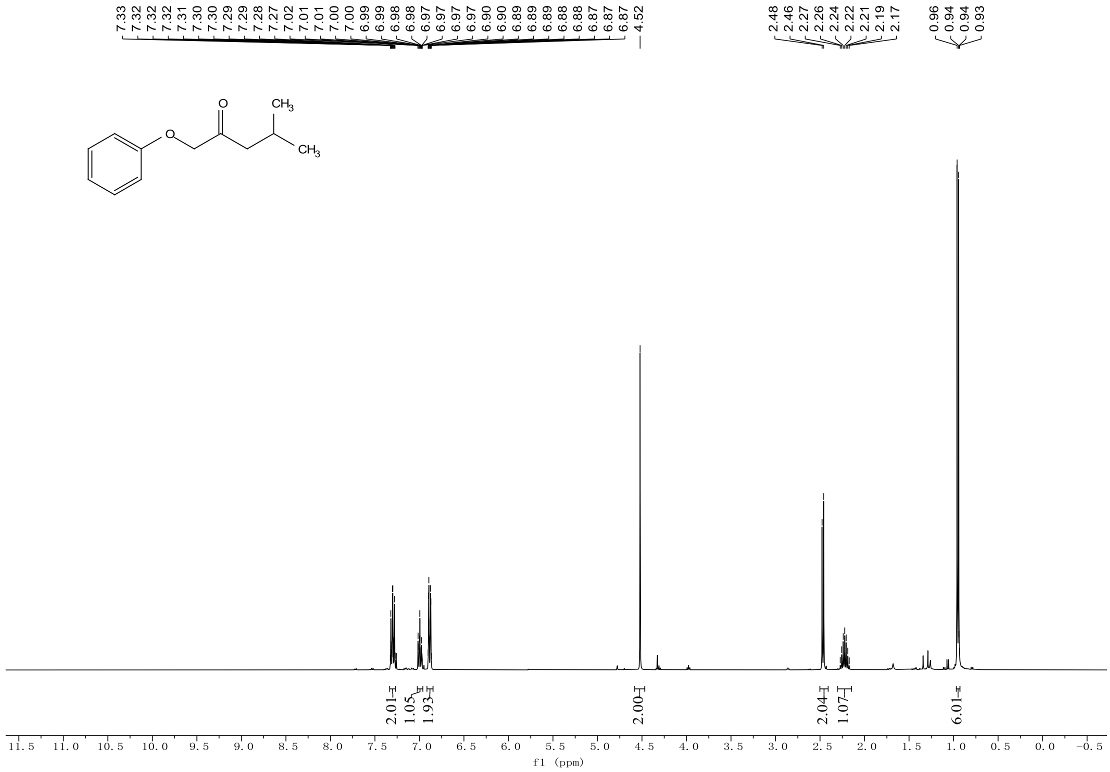

^{13}C NMR (101 MHz, $CDCl_3$) **4-methyl-1-phenoxypentan-2-one (23)**

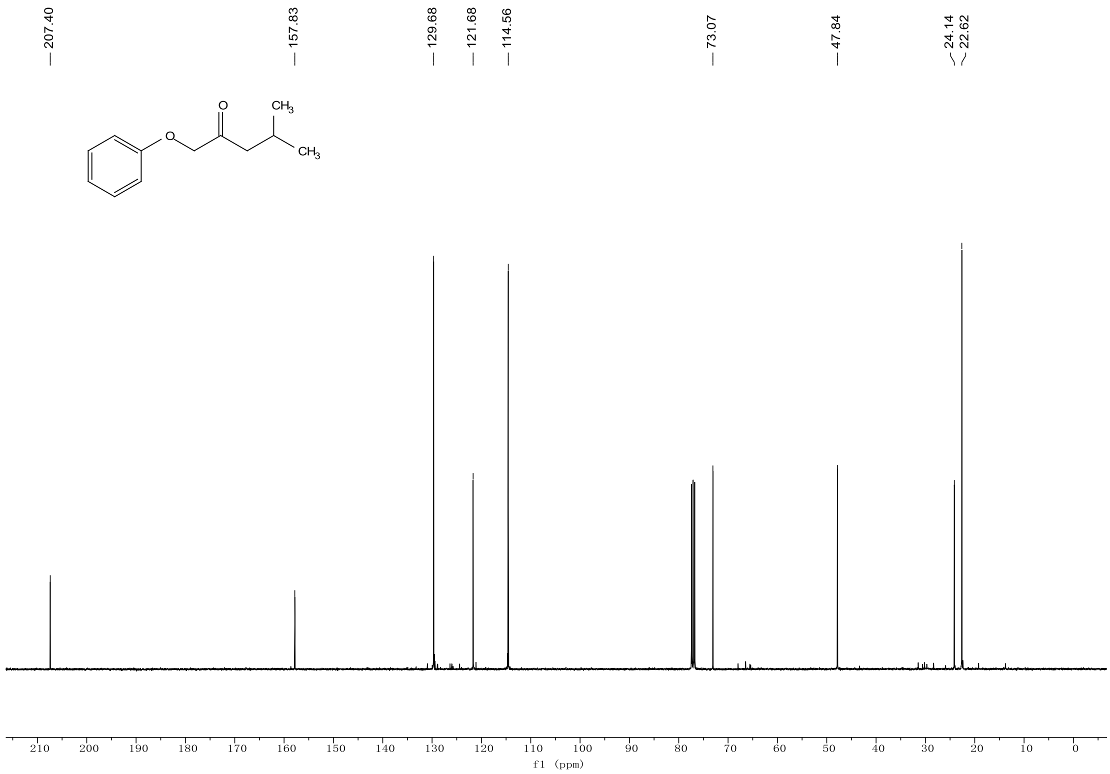

^{1}H NMR (400 MHz, $CDCl_3$) **3-methyl-1-phenoxybutan-2-one (P24)**

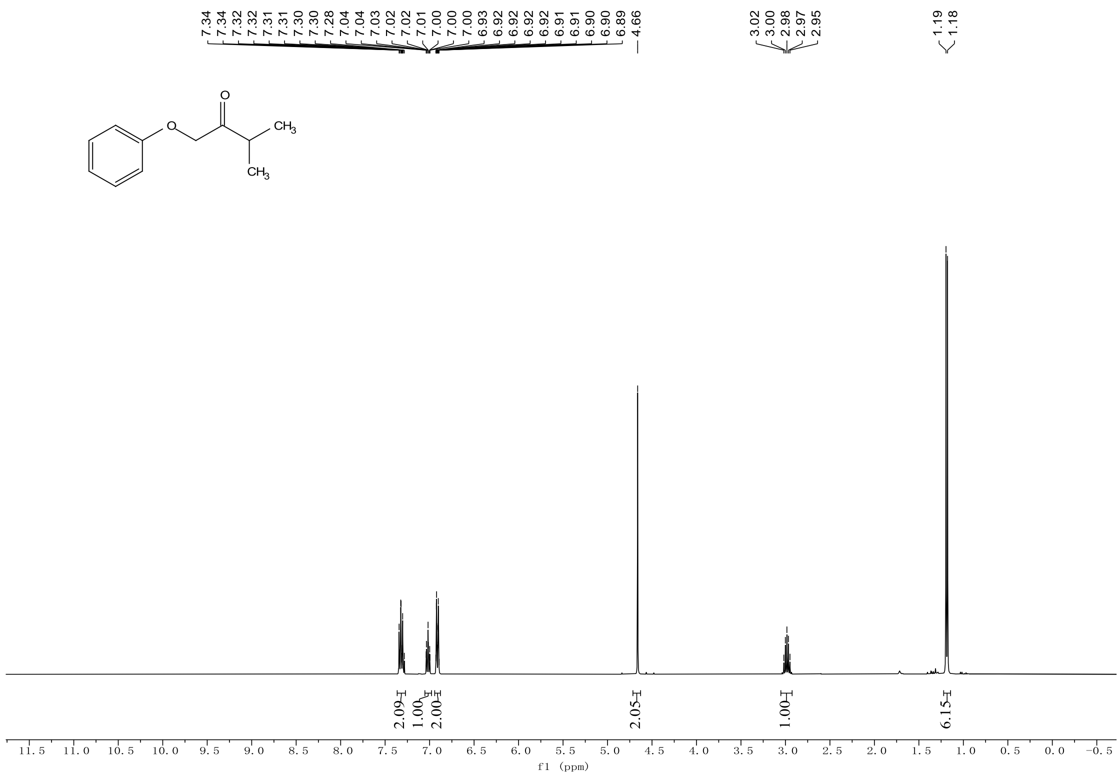

^{13}C NMR (101 MHz, $CDCl_3$) **3-methyl-1-phenoxybutan-2-one (P24)**

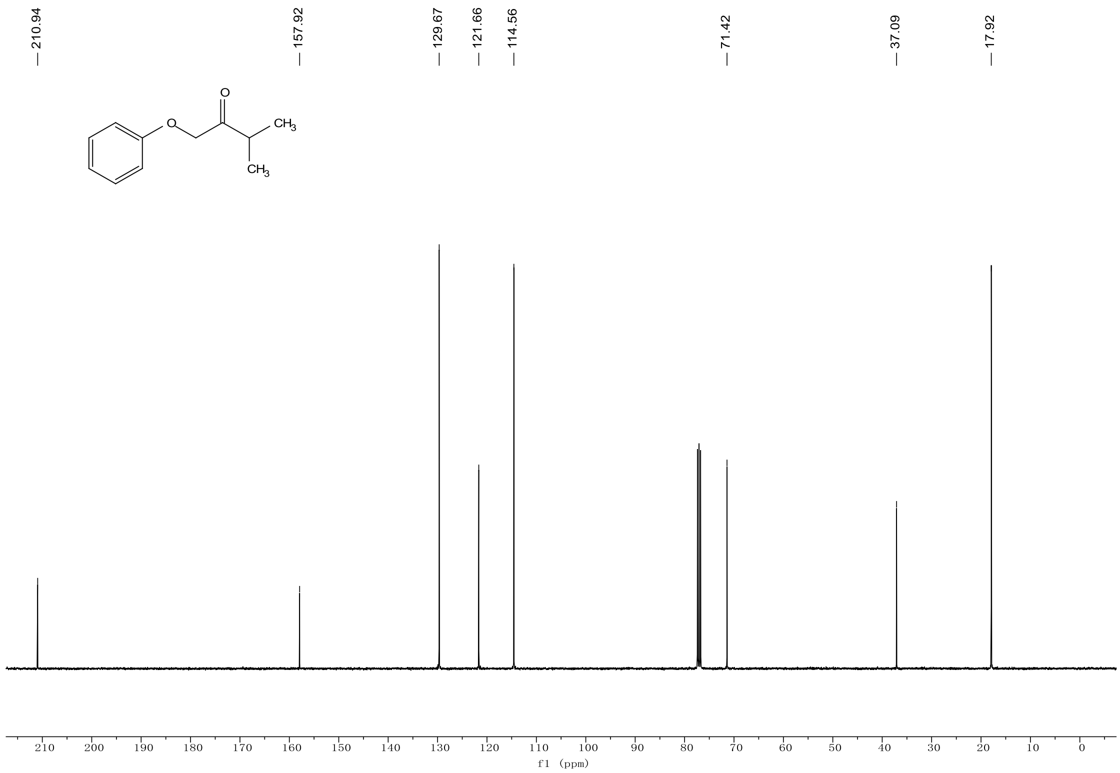

^{1}H NMR (400 MHz, $CDCl_3$) **3-methyl-1-phenoxypentan-2-one (P25)**

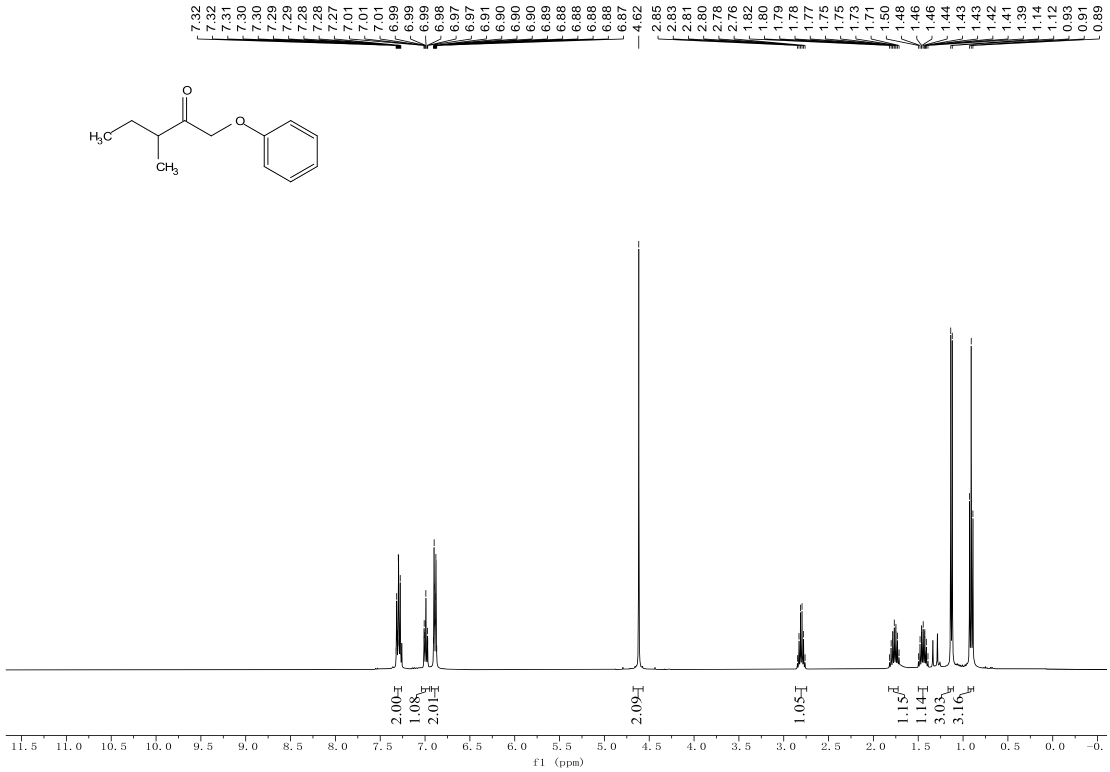

^{13}C NMR (101 MHz, $CDCl_3$) **3-methyl-1-phenoxypentan-2-one (P25)**

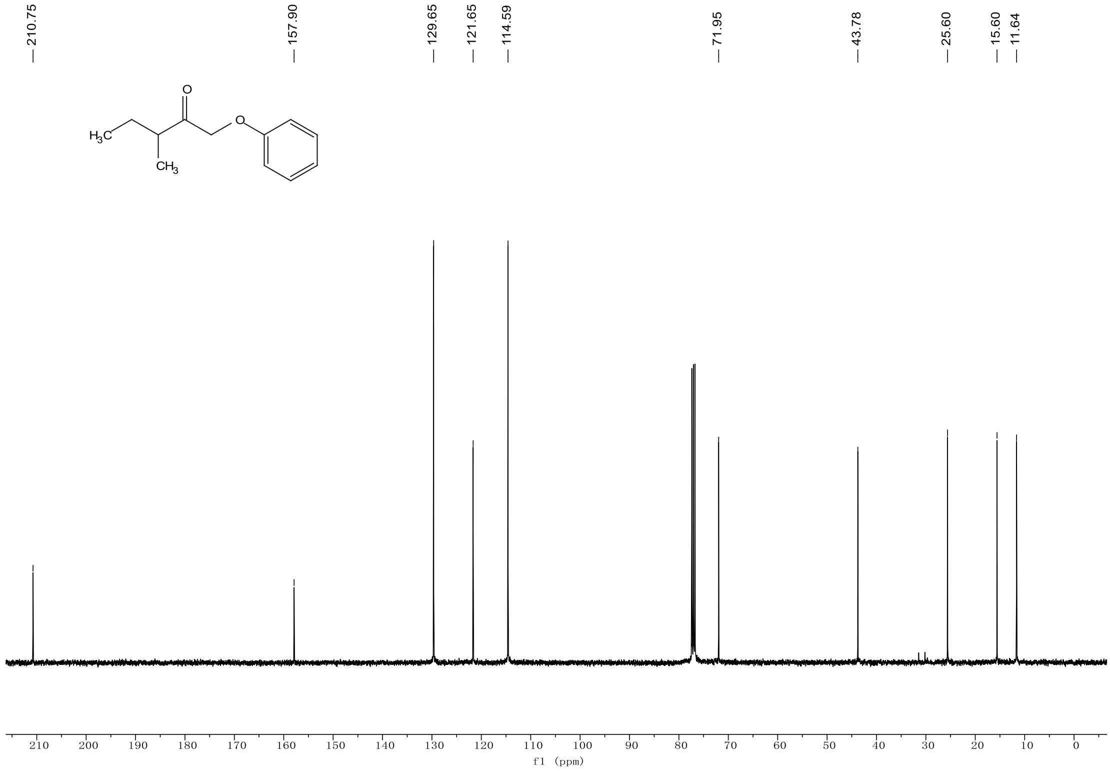

^{1}H NMR (400 MHz, $CDCl_3$) **1-cyclopropyl-2-phenoxyethan-1-one (P26)**

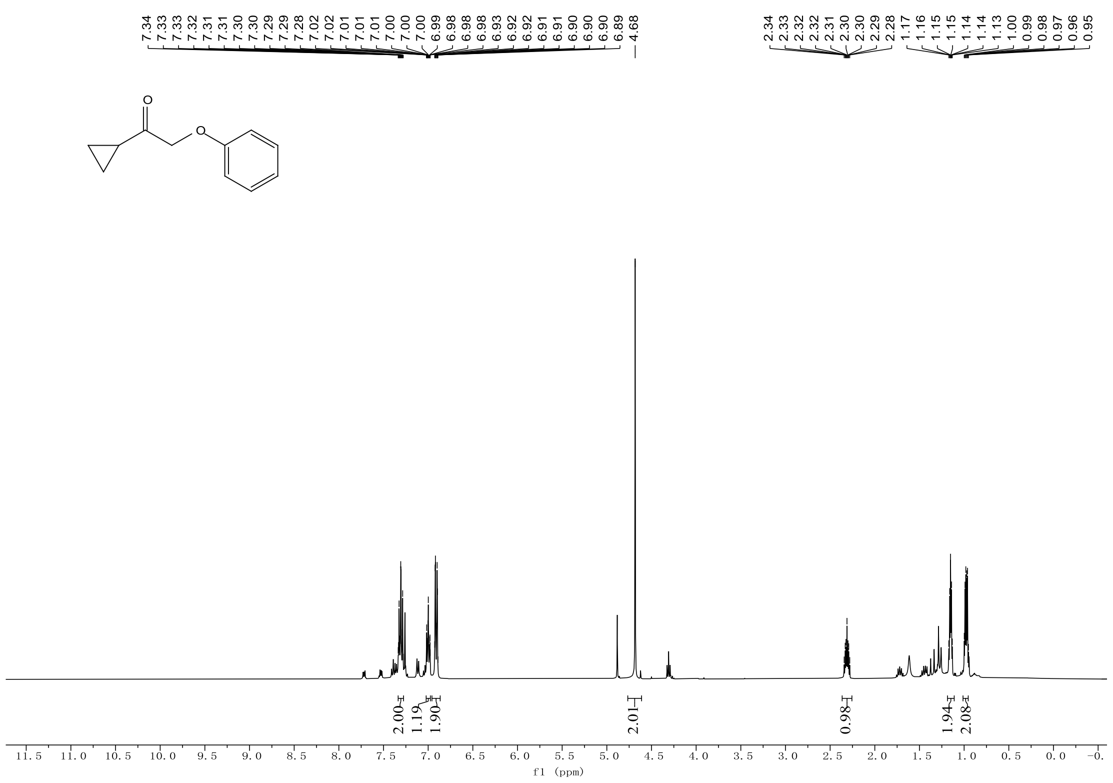

^{13}C NMR (101 MHz, $CDCl_3$) **1-cyclopropyl-2-phenoxyethan-1-one (P26)**

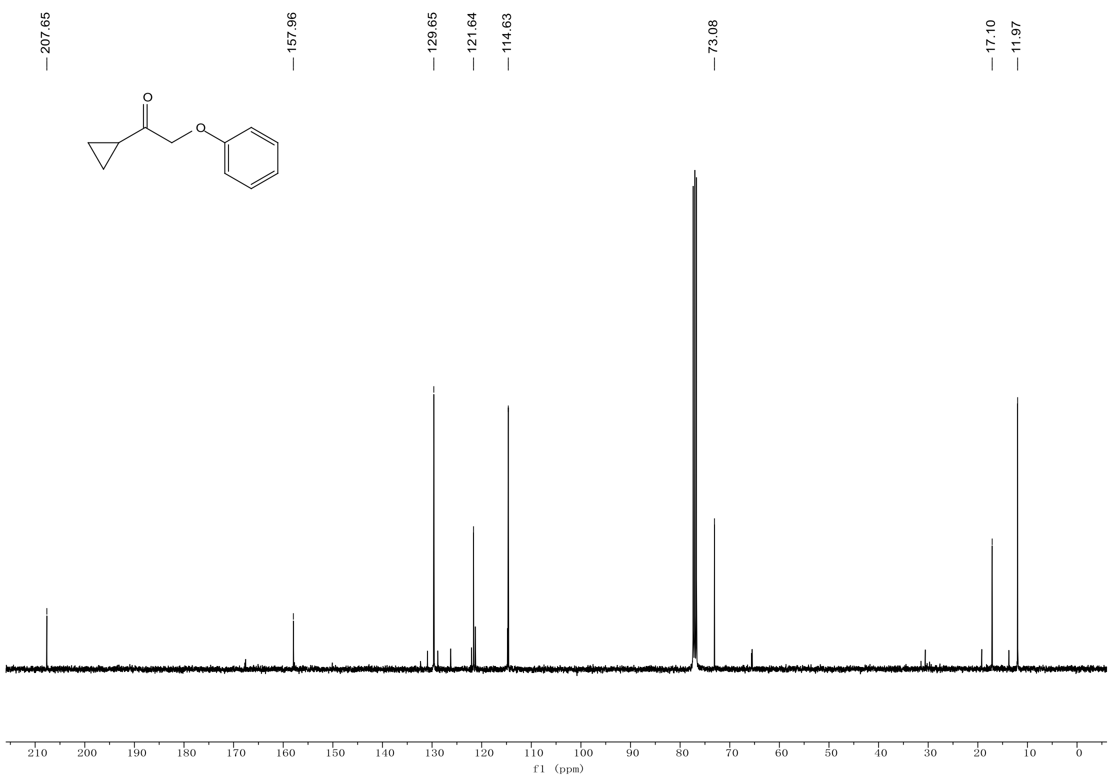

^{1}H NMR (400 MHz, $CDCl_3$) **1-cyclobutyl-2-phenoxyethan-1-one (P27)**

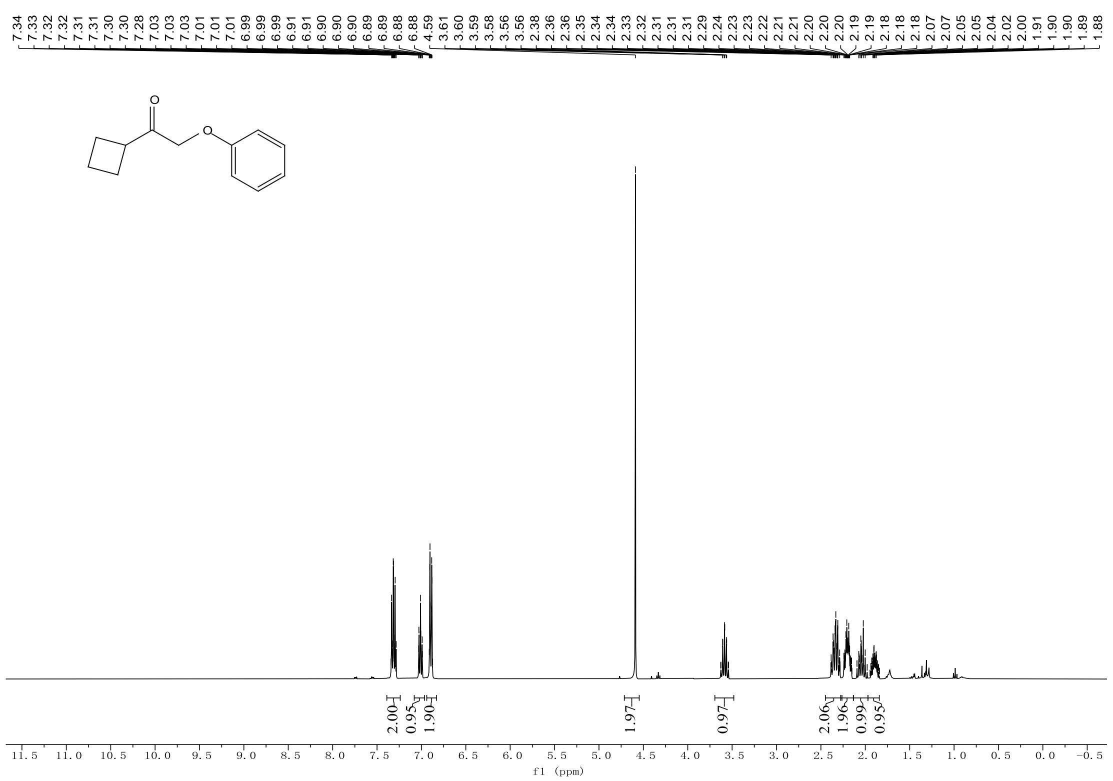

^{13}C NMR (101 MHz, $CDCl_3$) **1-cyclobutyl-2-phenoxyethan-1-one (P27)**

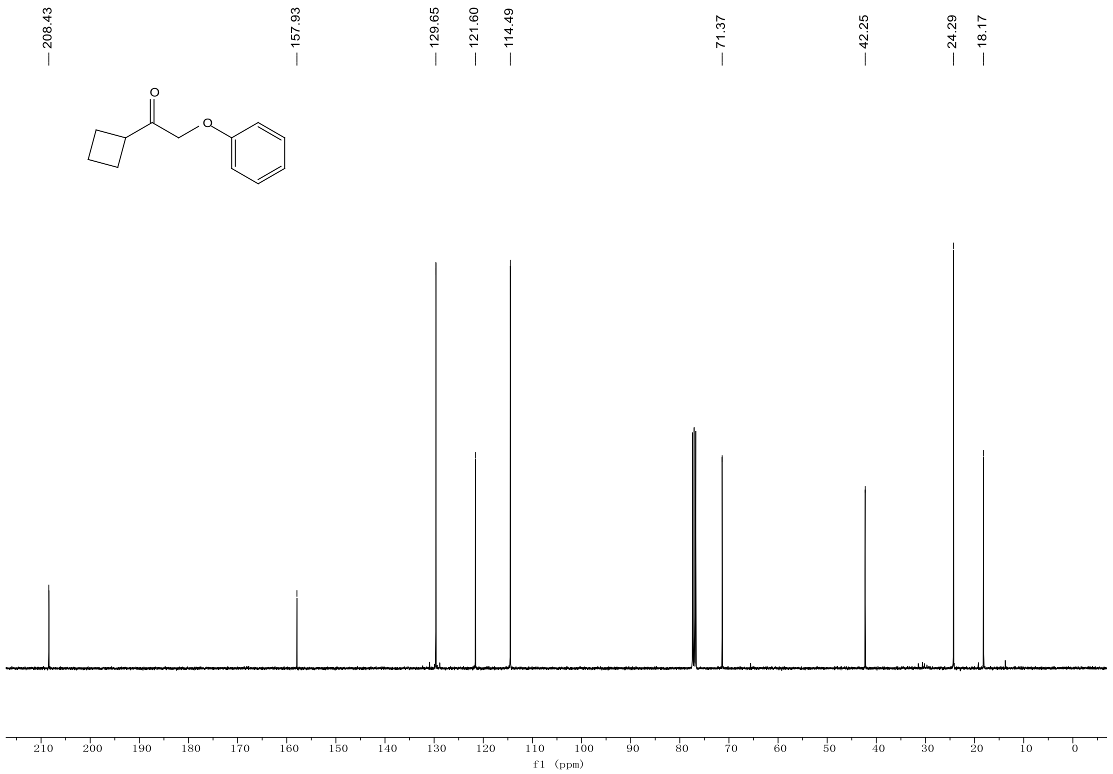

^{1}H NMR (400 MHz, $CDCl_3$) **1-cyclopentyl-2-phenoxyethan-1-one (P28)**

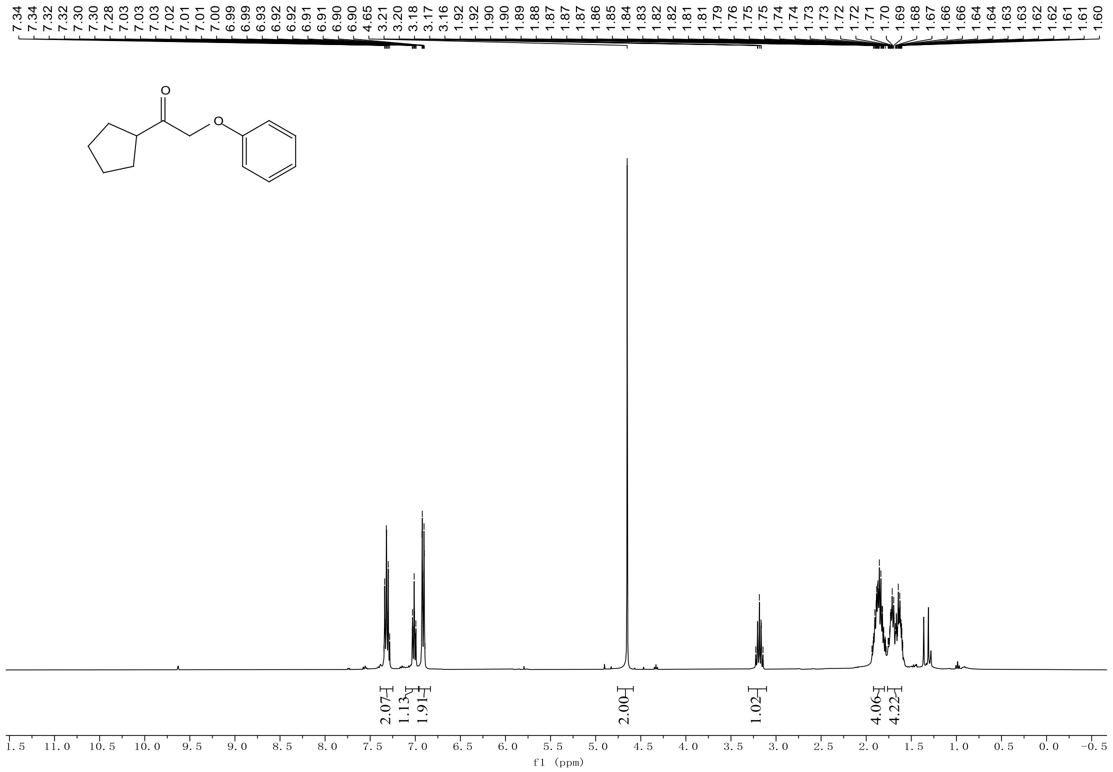

^{13}C NMR (101 MHz, $CDCl_3$) **1-cyclopentyl-2-phenoxyethan-1-one (P28)**

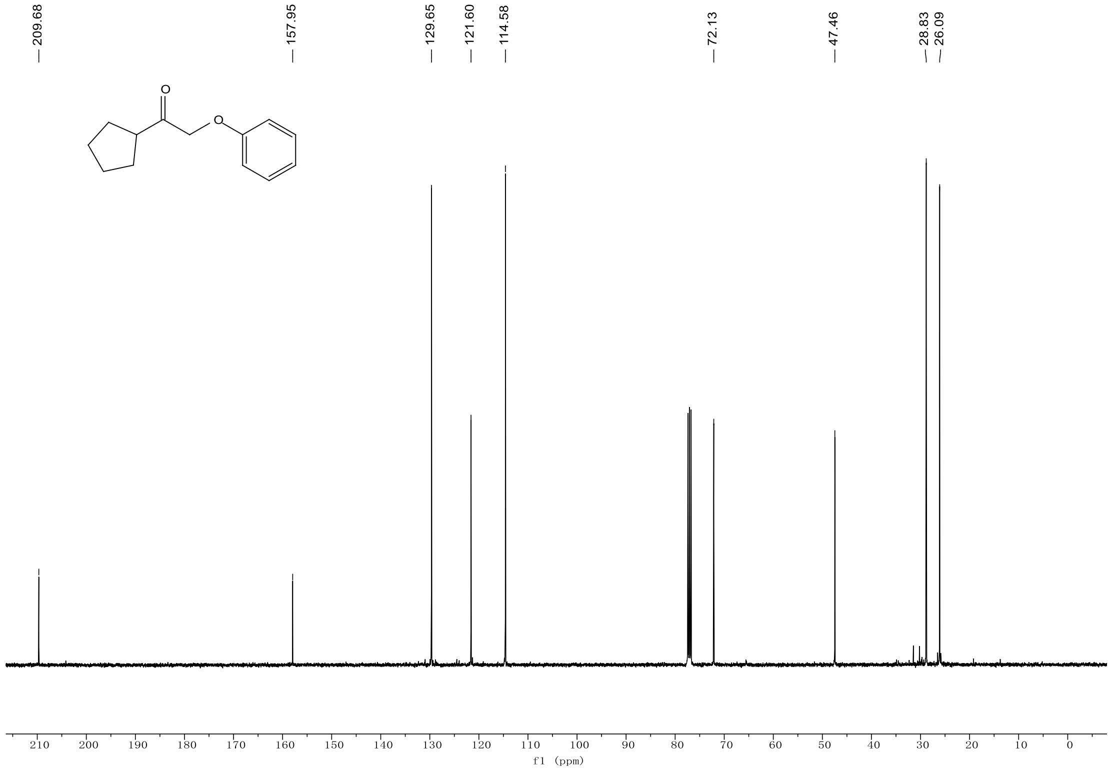

^{1}H NMR (400 MHz, $CDCl_3$) **1-cyclohexyl-2-phenoxyethan-1-one (P29)**

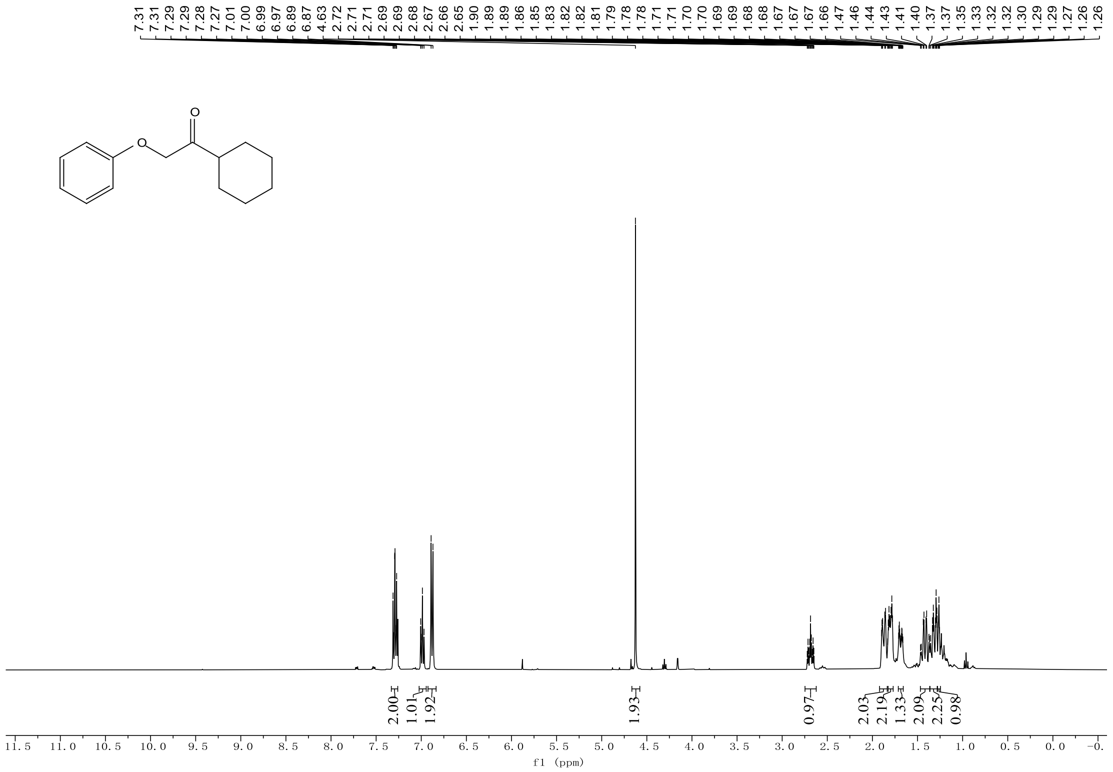

^{13}C NMR (101 MHz, $CDCl_3$) **1-cyclohexyl-2-phenoxyethan-1-one (P29)**

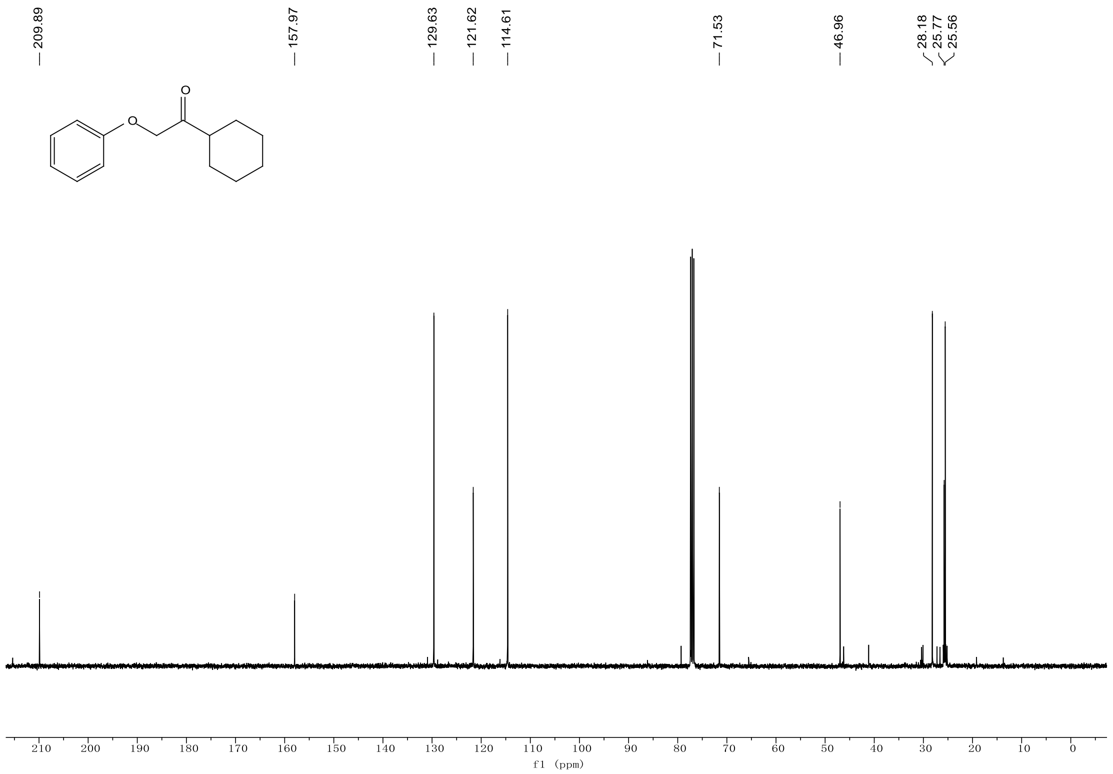

^{1}H NMR (400 MHz, $CDCl_3$) **6-chloro-1-phenoxyhexan-2-one (P30)**

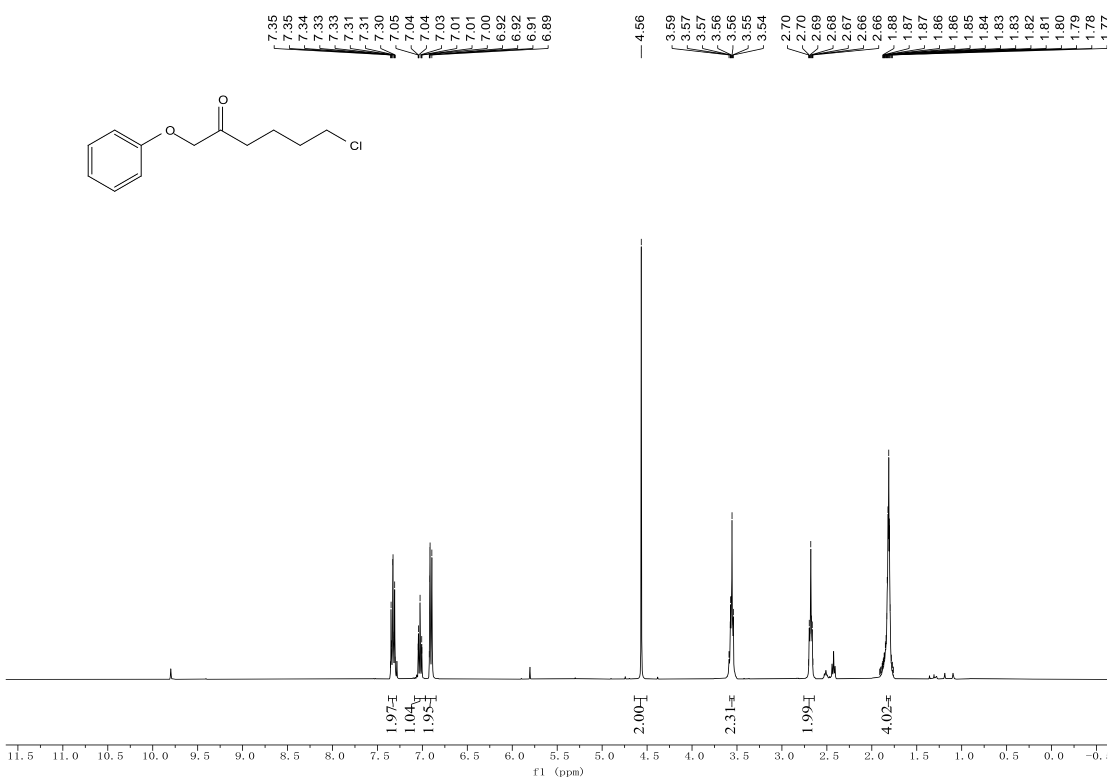

^{13}C NMR (101 MHz, $CDCl_3$) **6-chloro-1-phenoxyhexan-2-one (P30)**

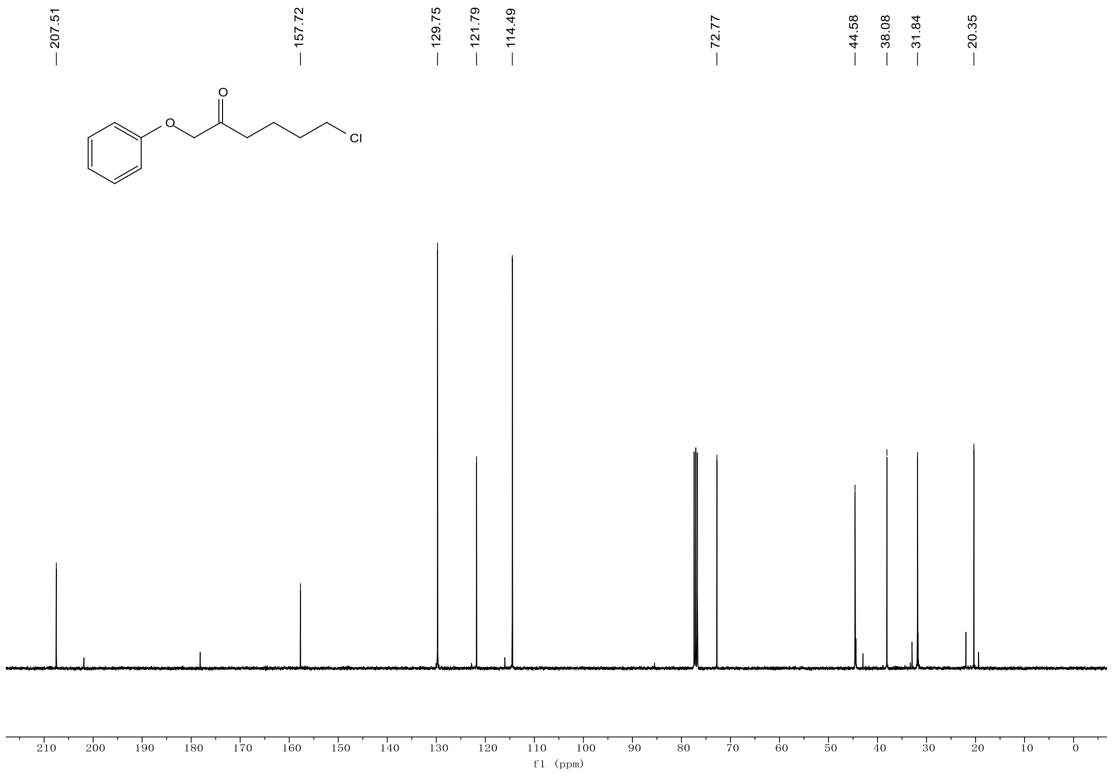

^{1}H NMR (400 MHz, $CDCl_3$) **4-phenylbutan-2-one (P31)**

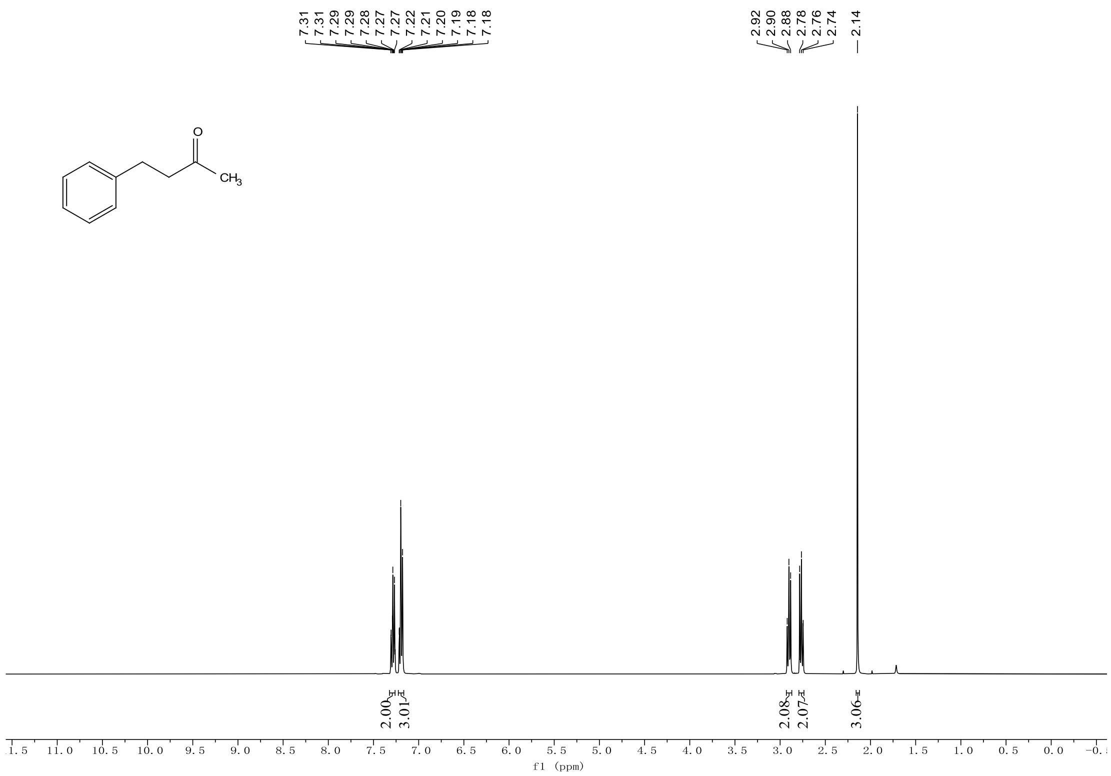

^{13}C NMR (101 MHz, $CDCl_3$) **4-phenylbutan-2-one (P31)**

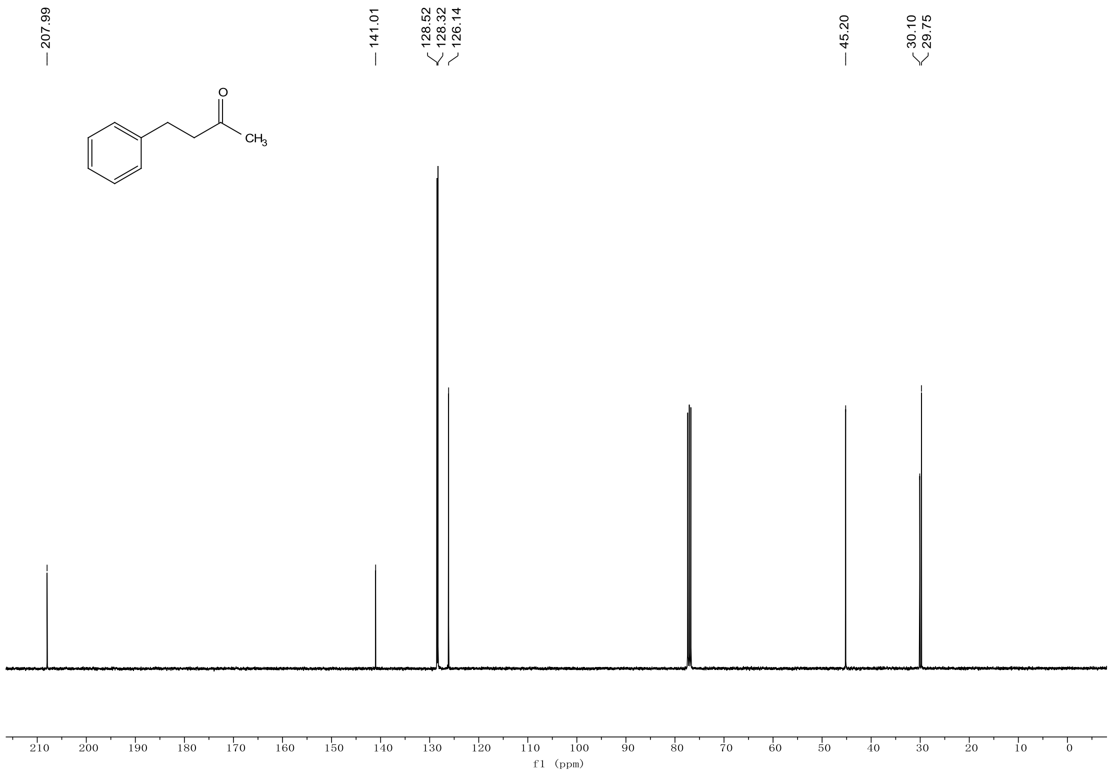

^{1}H NMR (400 MHz, $CDCl_3$) **1-phenylpentan-3-one (P32)**

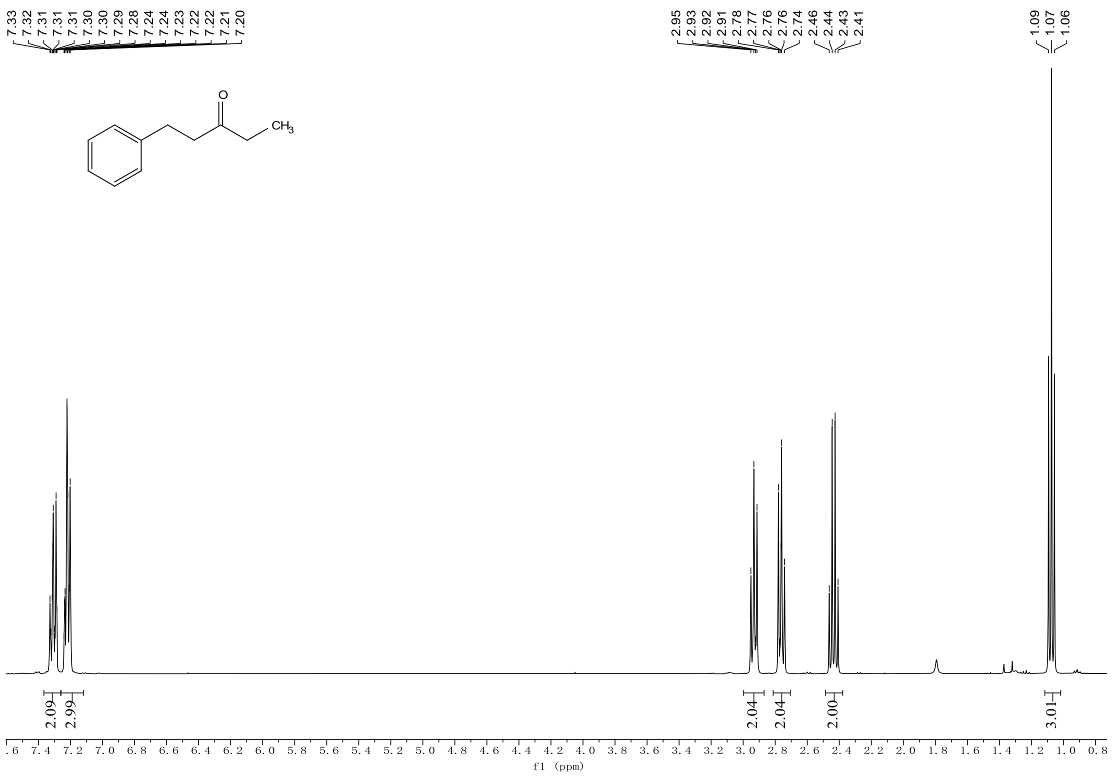

^{13}C NMR (101 MHz, $CDCl_3$) **1-phenylpentan-3-one (P32)**

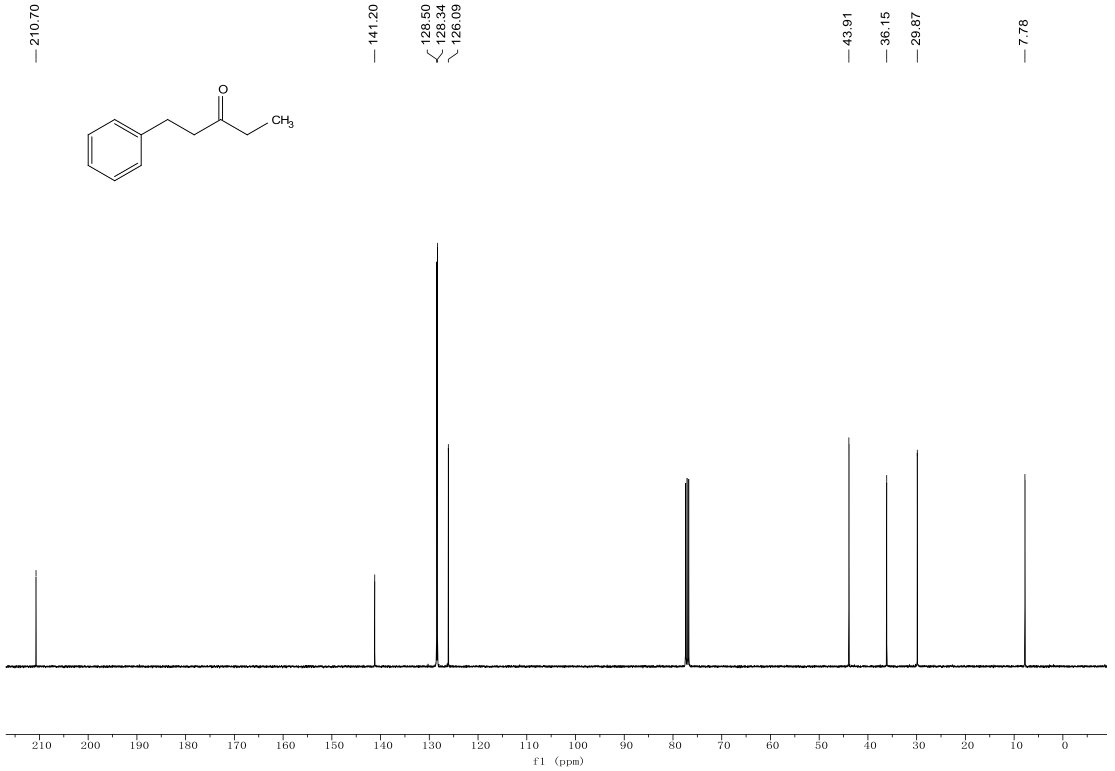

^{1}H NMR (400 MHz, $CDCl_3$) **4-methyl-1-phenylpentan-3-one (P33)**

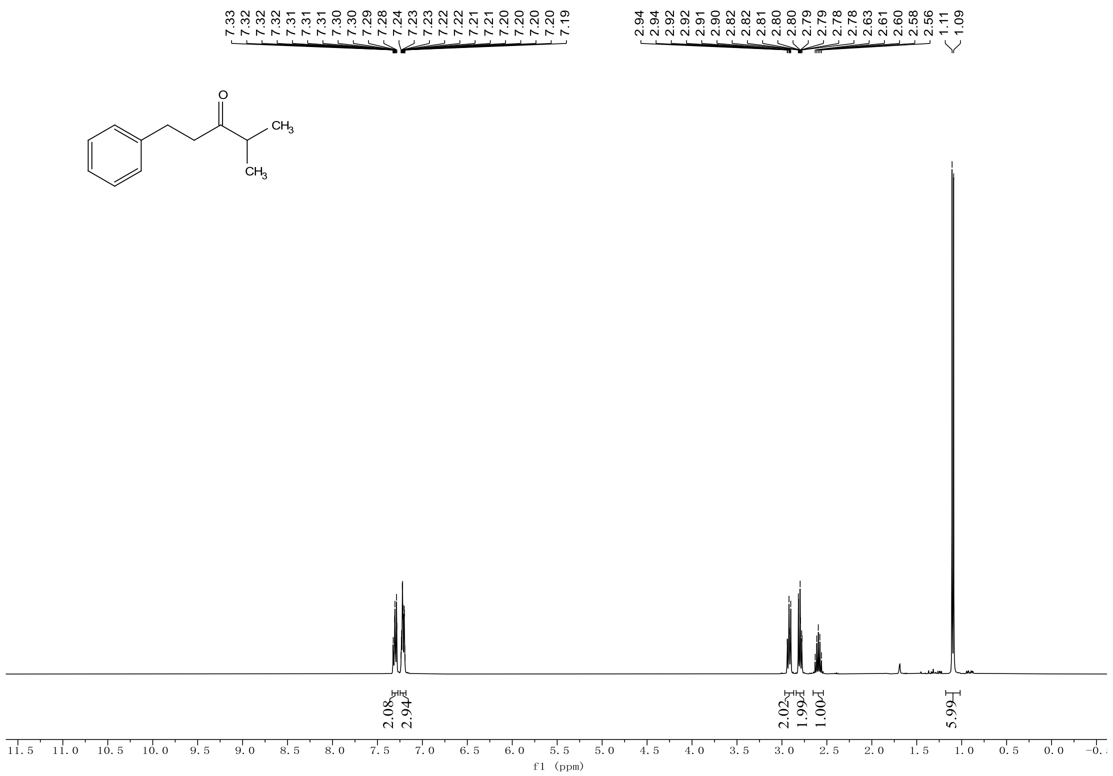

^{13}C NMR (101 MHz, CDCl$_3$) **4-methyl-1-phenylpentan-3-one (P33)**

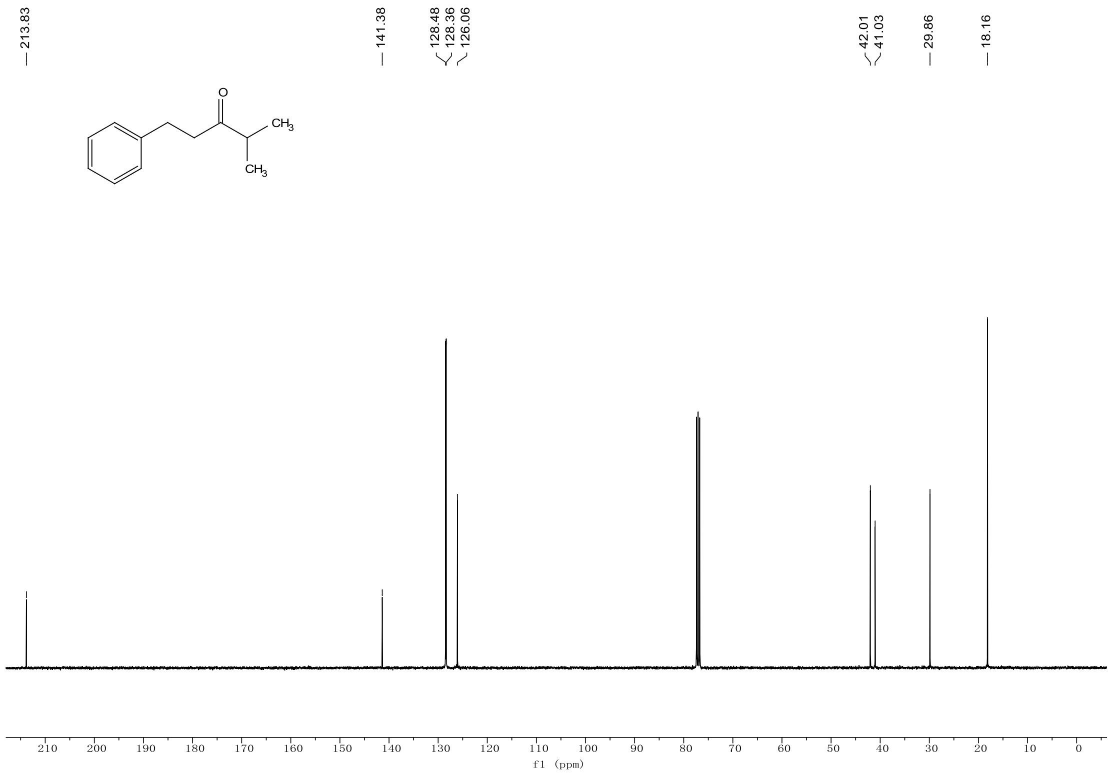

^{1}H NMR (400 MHz, CDCl$_3$) **1-cyclopropyl-3-phenylpropan-1-one (P34)**

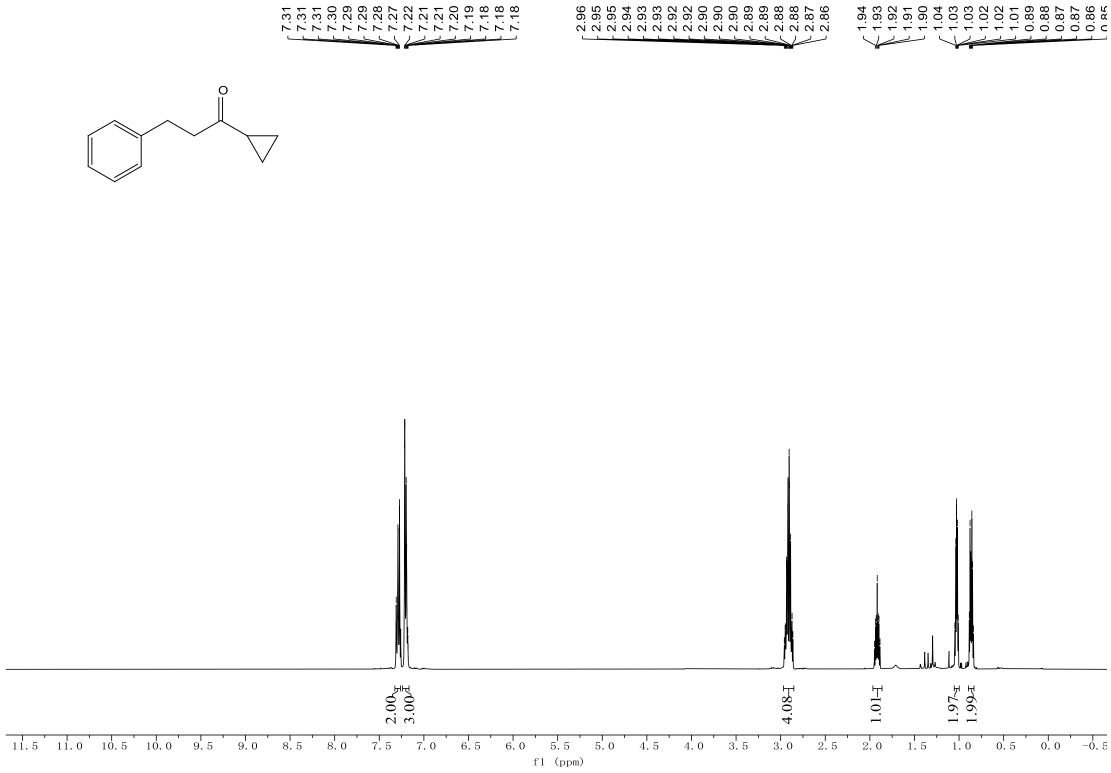

^{13}C NMR (101 MHz, $CDCl_3$) **1-cyclopropyl-3-phenylpropan-1-one (P34)**

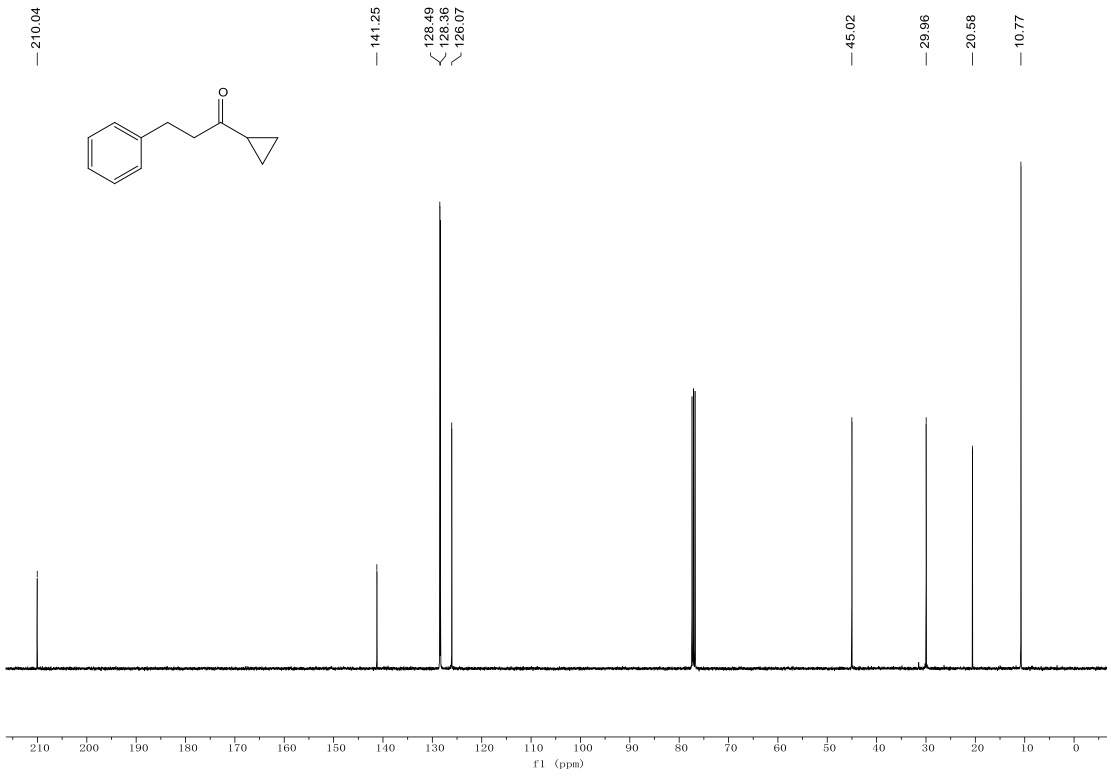

^{1}H NMR (400 MHz, $CDCl_3$) **1-cyclobutylethan-1-one (P35)**

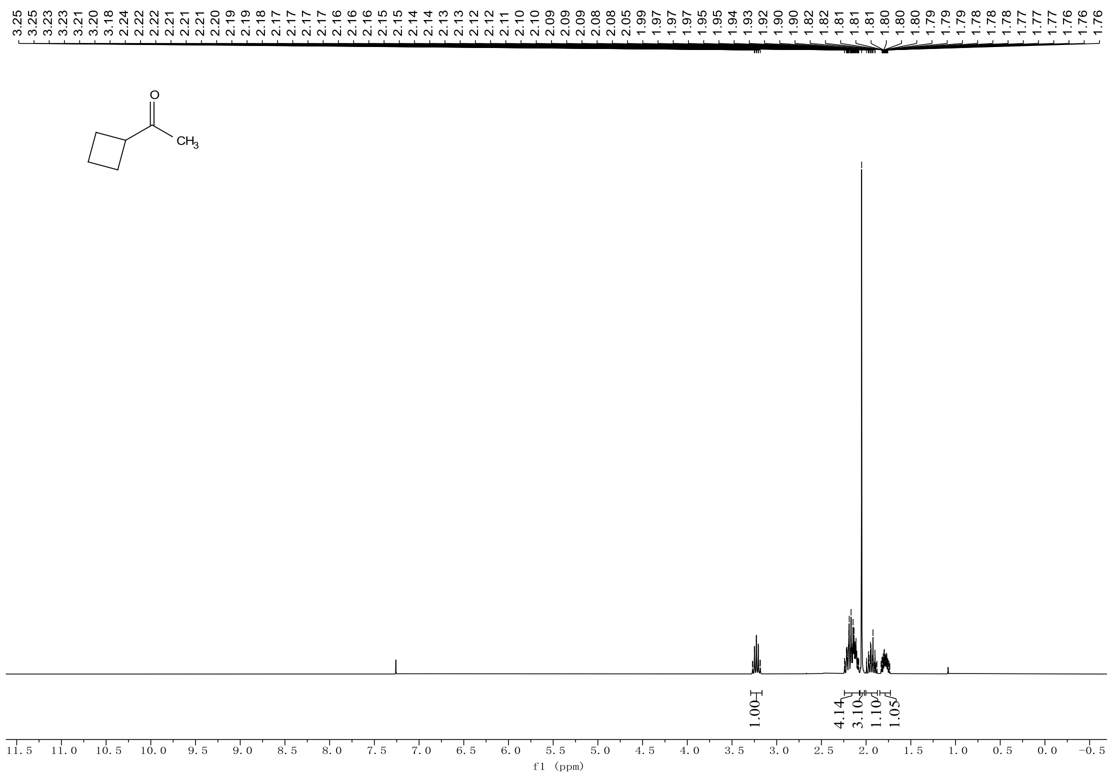

^{13}C NMR (101 MHz, $CDCl_3$) **1-cyclobutylethan-1-one (P35)**

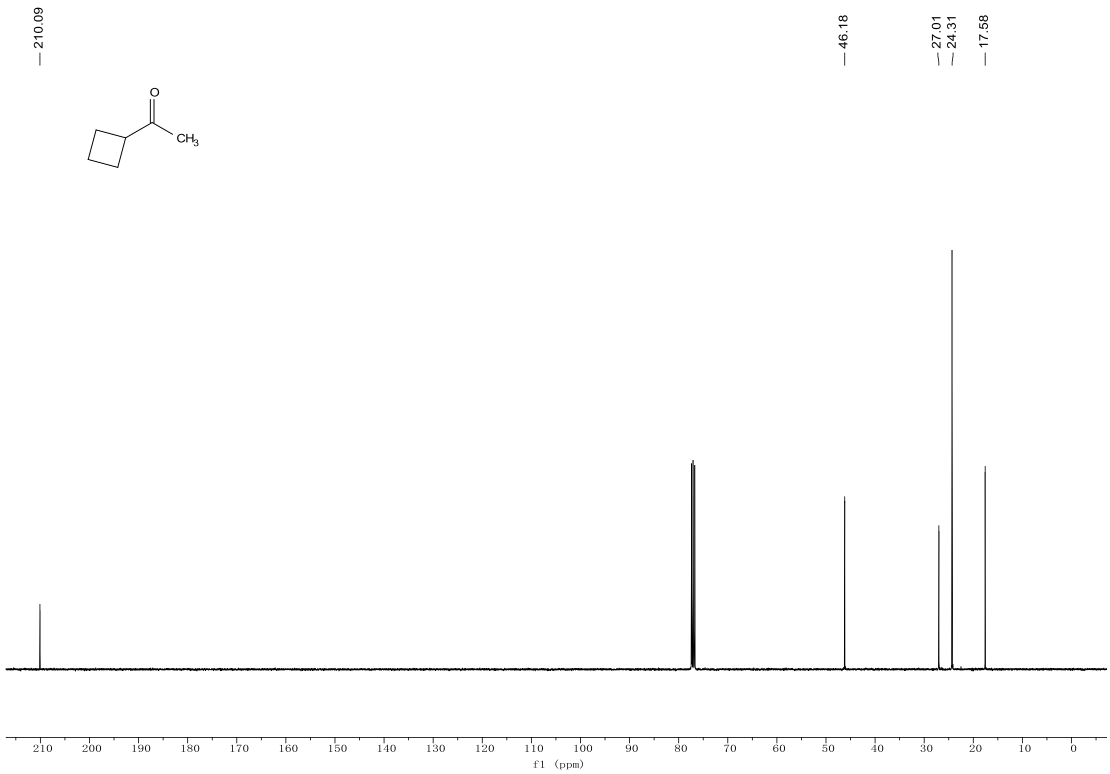

^{1}H NMR (400 MHz, $CDCl_3$) **1-cyclopentylethan-1-one (P36)**

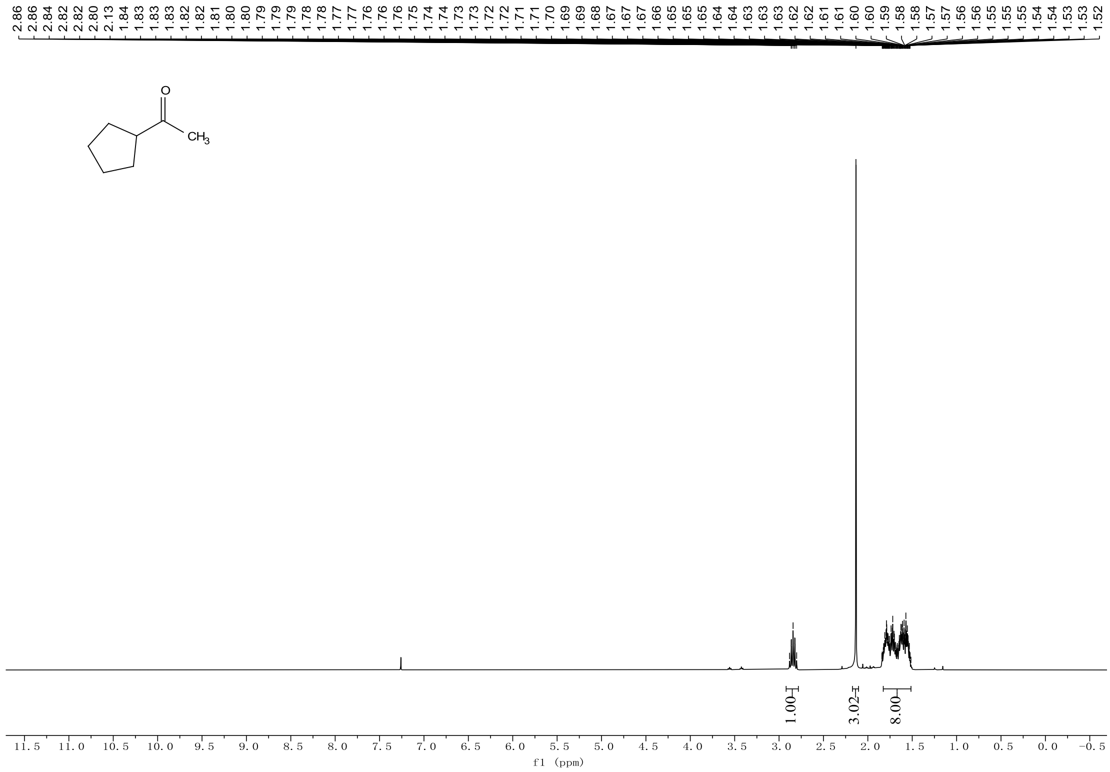

^{13}C NMR (101 MHz, $CDCl_3$) **1-cyclopentylethan-1-one (P36)**

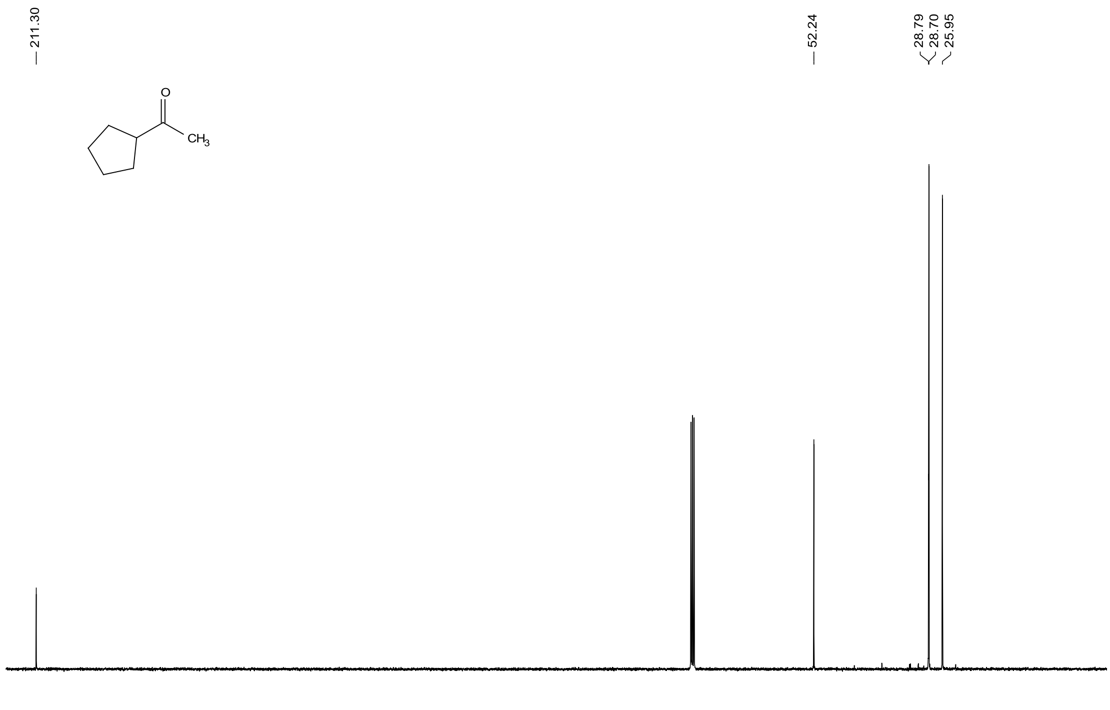

^{1}H NMR (400 MHz, $CDCl_3$) **1-cyclohexylethan-1-one (P37)**

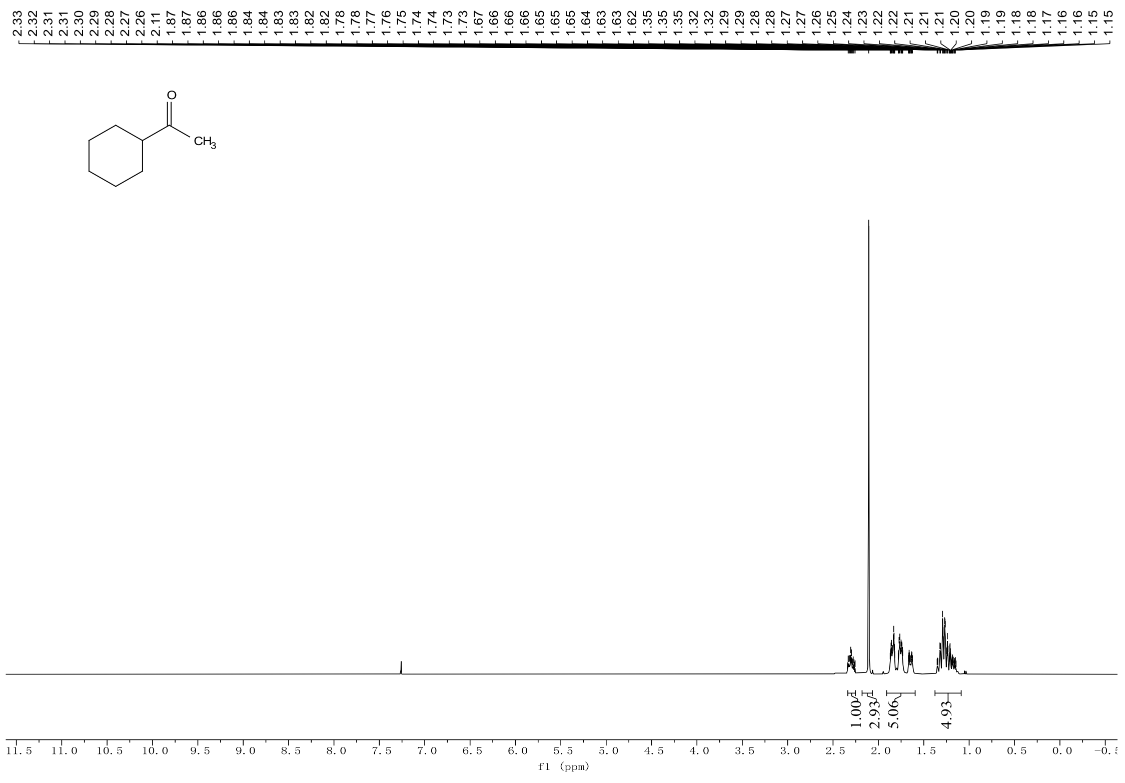

^{13}C NMR (101 MHz, $CDCl_3$) **1-cyclohexylethan-1-one (P37)**

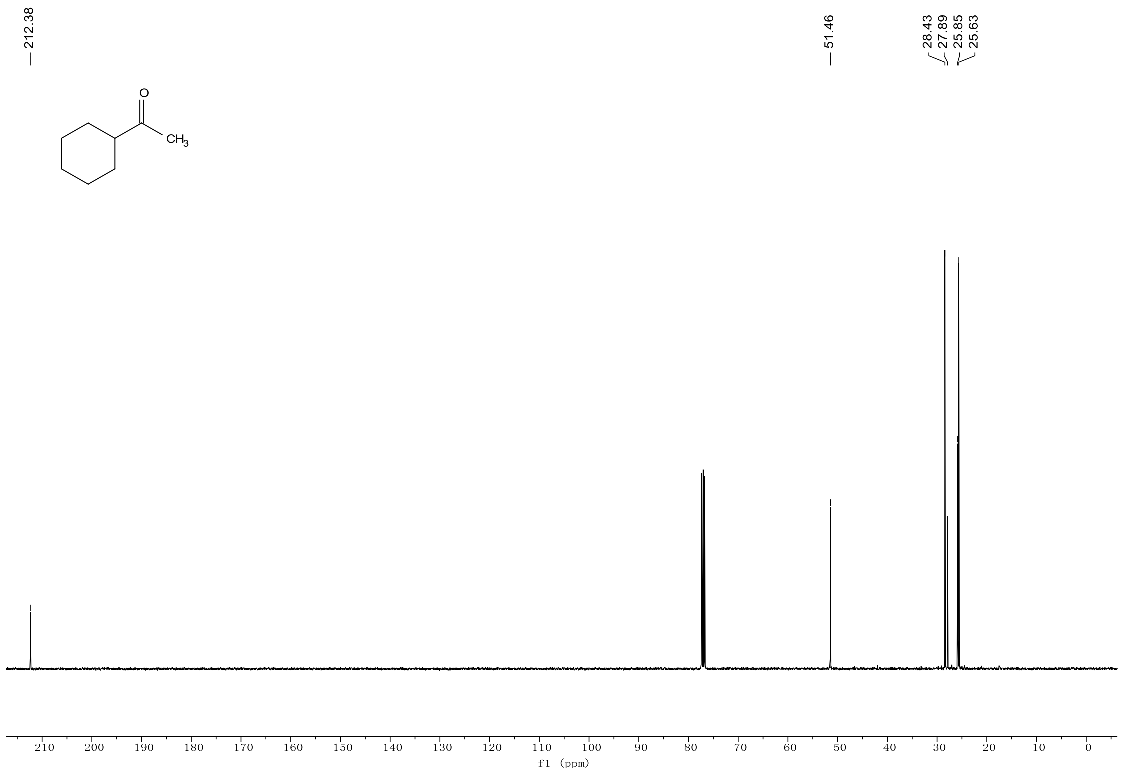

^{1}H NMR (400 MHz, $CDCl_3$) **1-(4'-pentyl-[1,1'-bi(cyclohexan)]-4-yl)ethan-1-one (P38)**

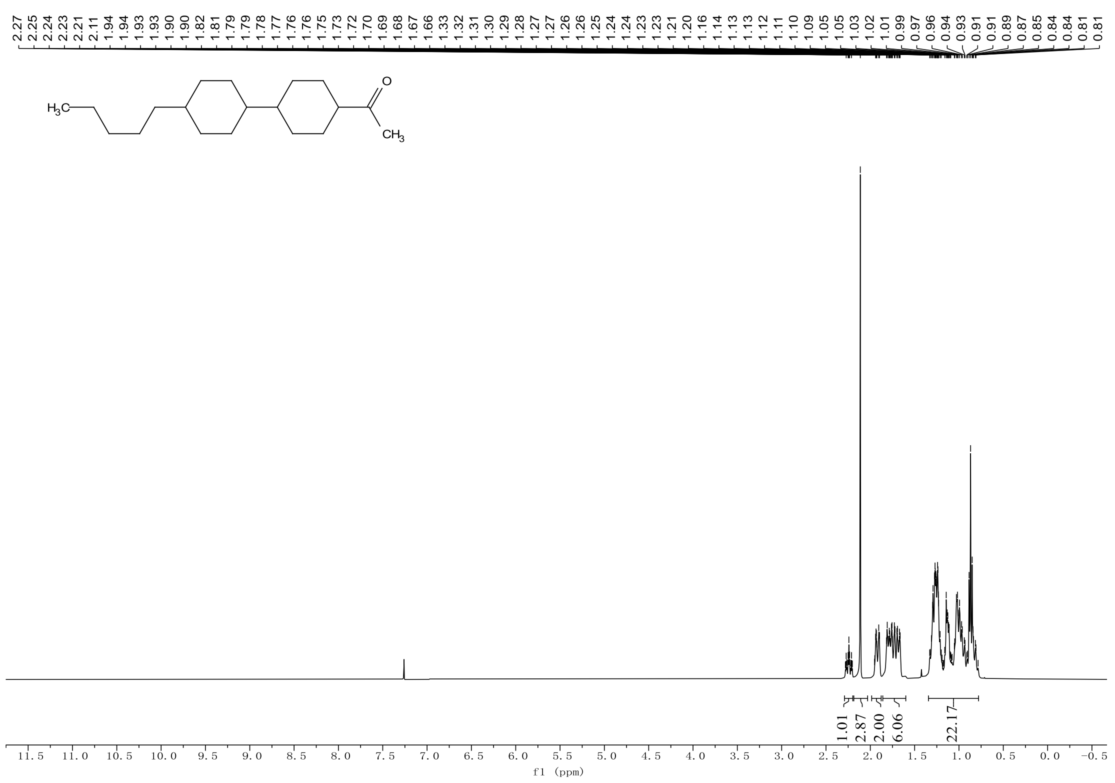

^{13}C NMR (101 MHz, $CDCl_3$) **1-(4'-pentyl-[1,1'-bi(cyclohexan)]-4-yl)ethan-1-one (P38)**

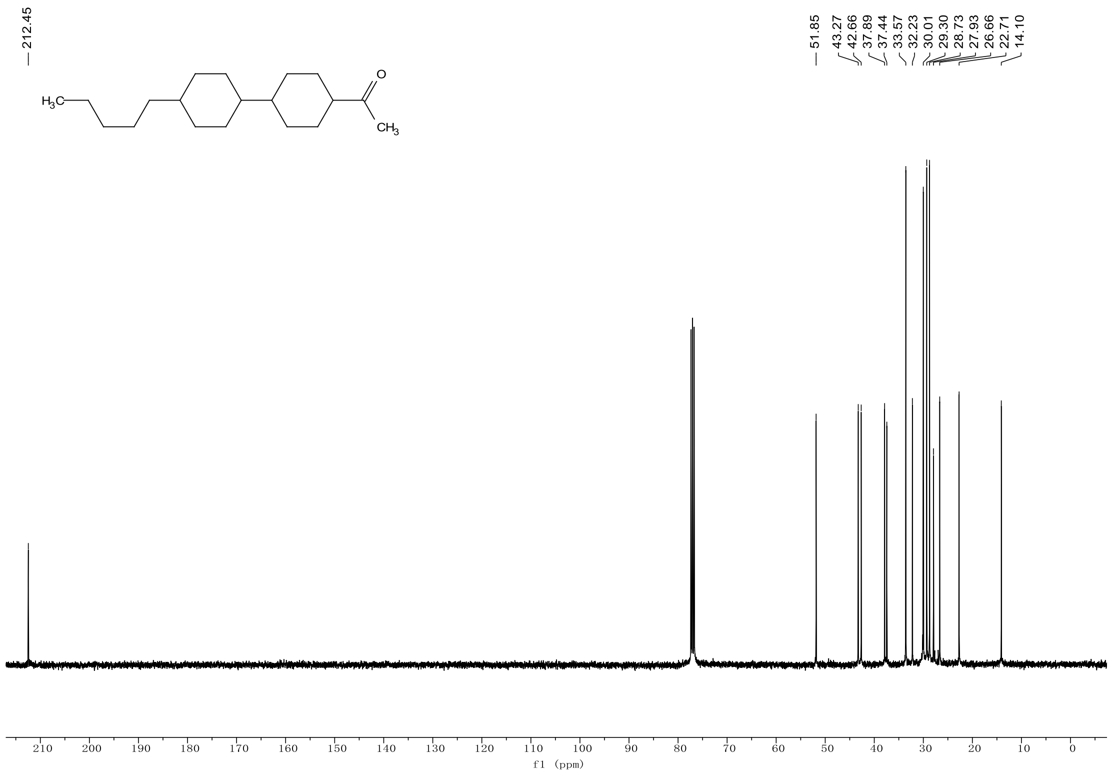

^{1}H NMR (400 MHz, $CDCl_3$) ***tert*-butyl 4-acetylpiperidine-1-carboxylate (P39).**

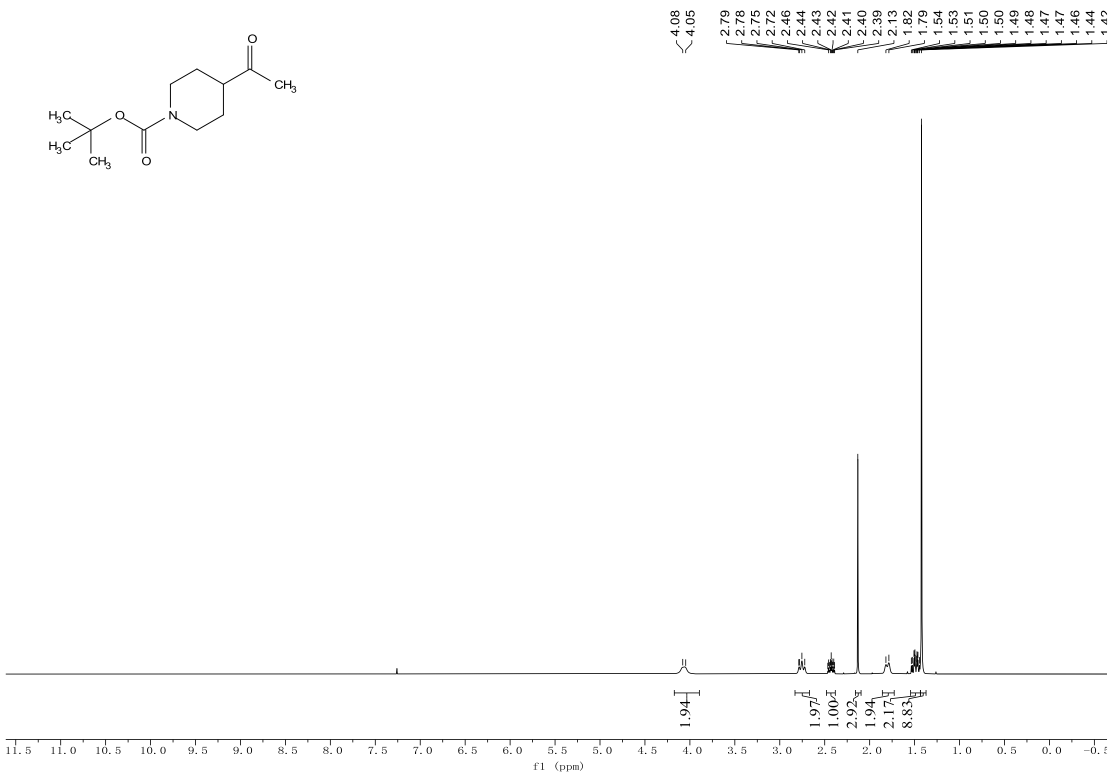

^{13}C NMR (101 MHz, $CDCl_3$) ***tert*-butyl 4-acetylpiperidine-1-carboxylate (P39).**

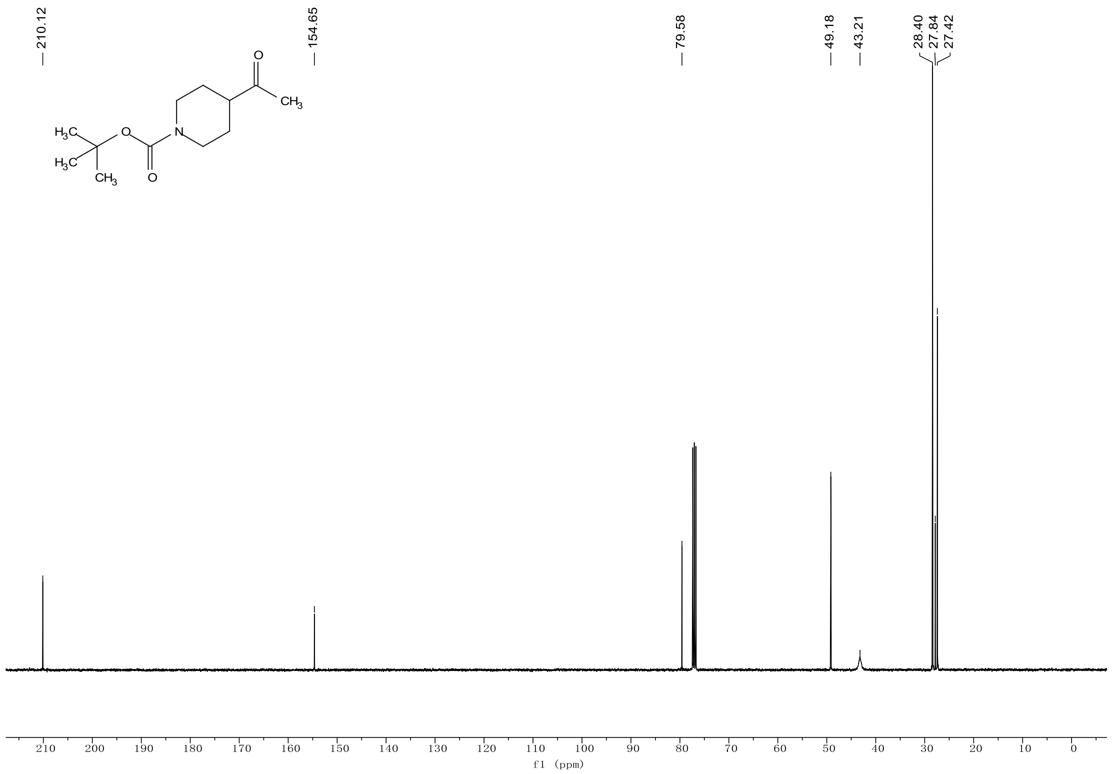

^{1}H NMR (400 MHz, $CDCl_3$) **1-(4-methoxyphenyl)propan-2-one (P40)**

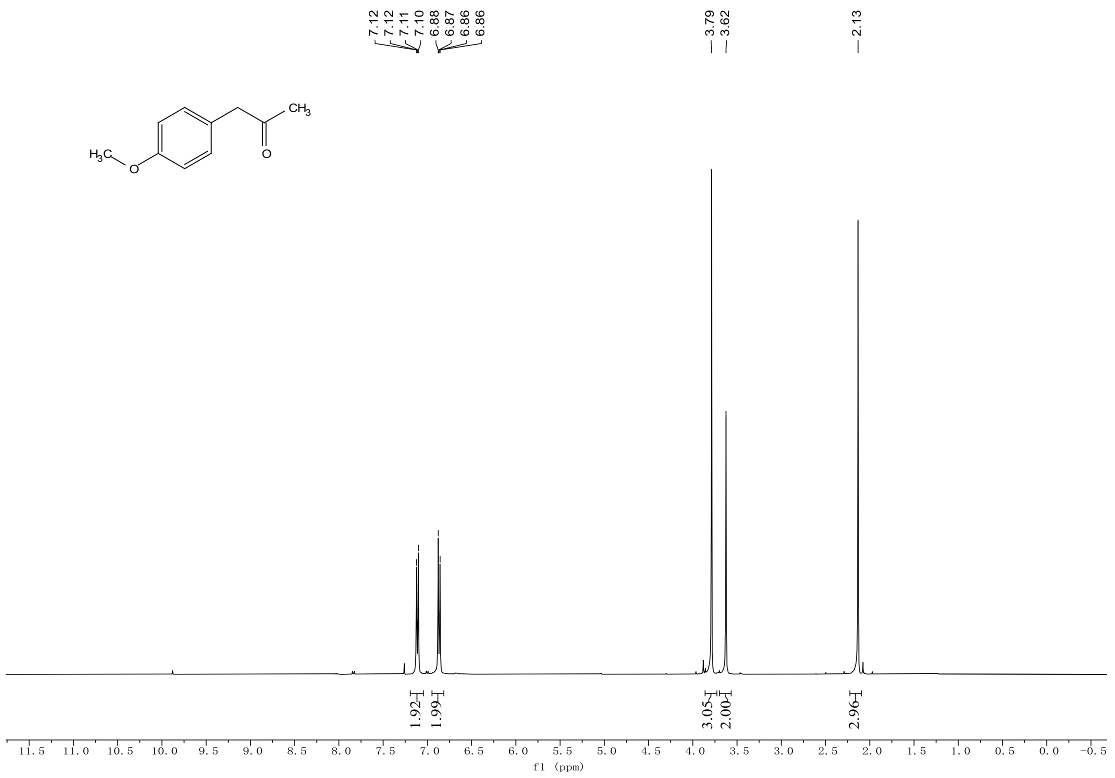

^{13}C NMR (101 MHz, $CDCl_3$) **1-(4-methoxyphenyl)propan-2-one (P40)**

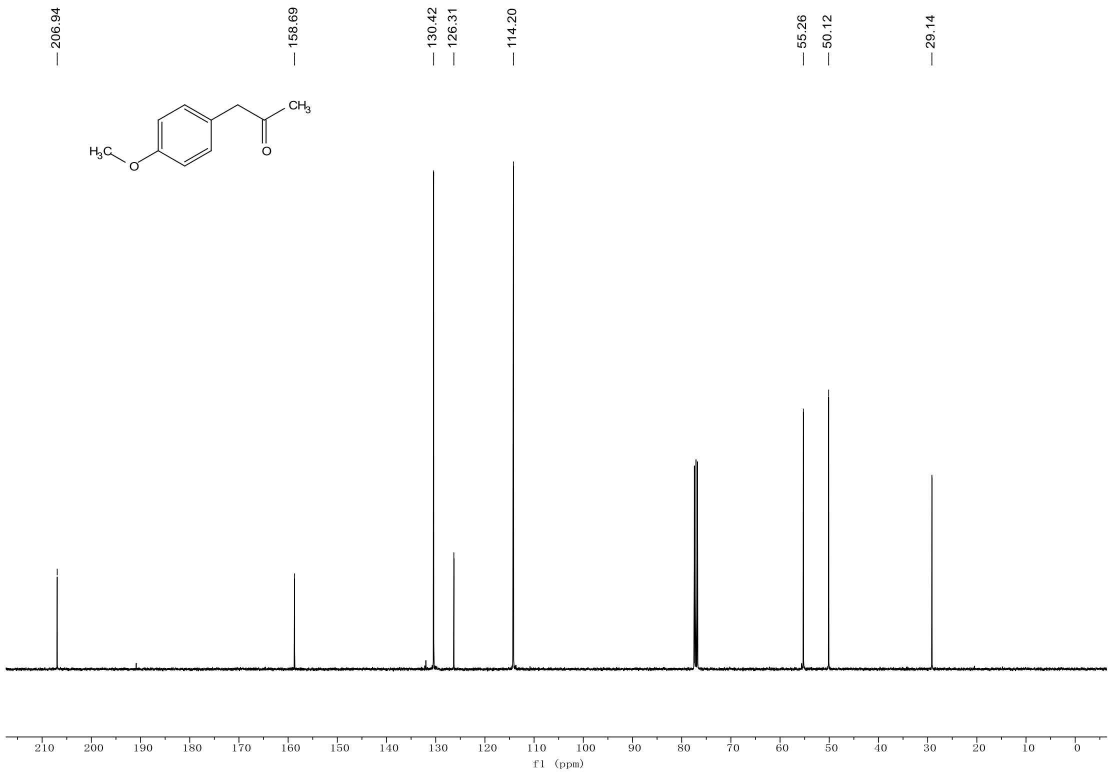

^{1}H NMR (400 MHz, $CDCl_3$) **1-(phenylthio)propan-2-one (P41)**

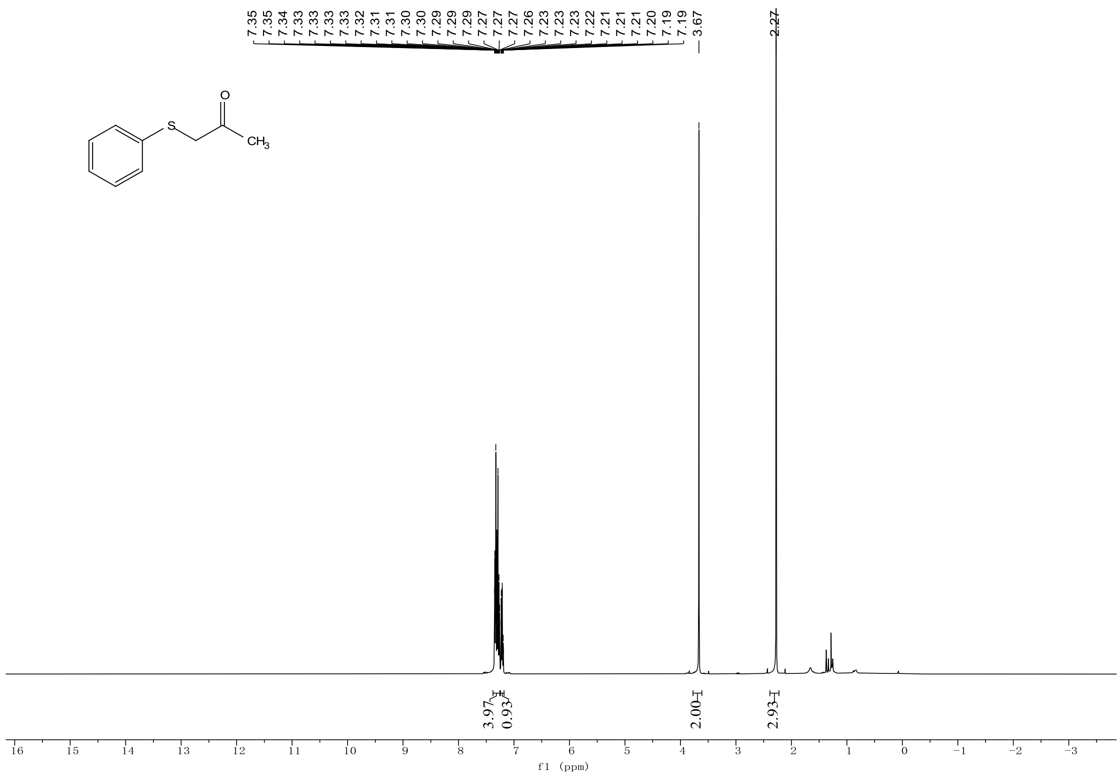

^{13}C NMR (101 MHz, CDCl$_3$) **1-(phenylthio)propan-2-one (P41)**

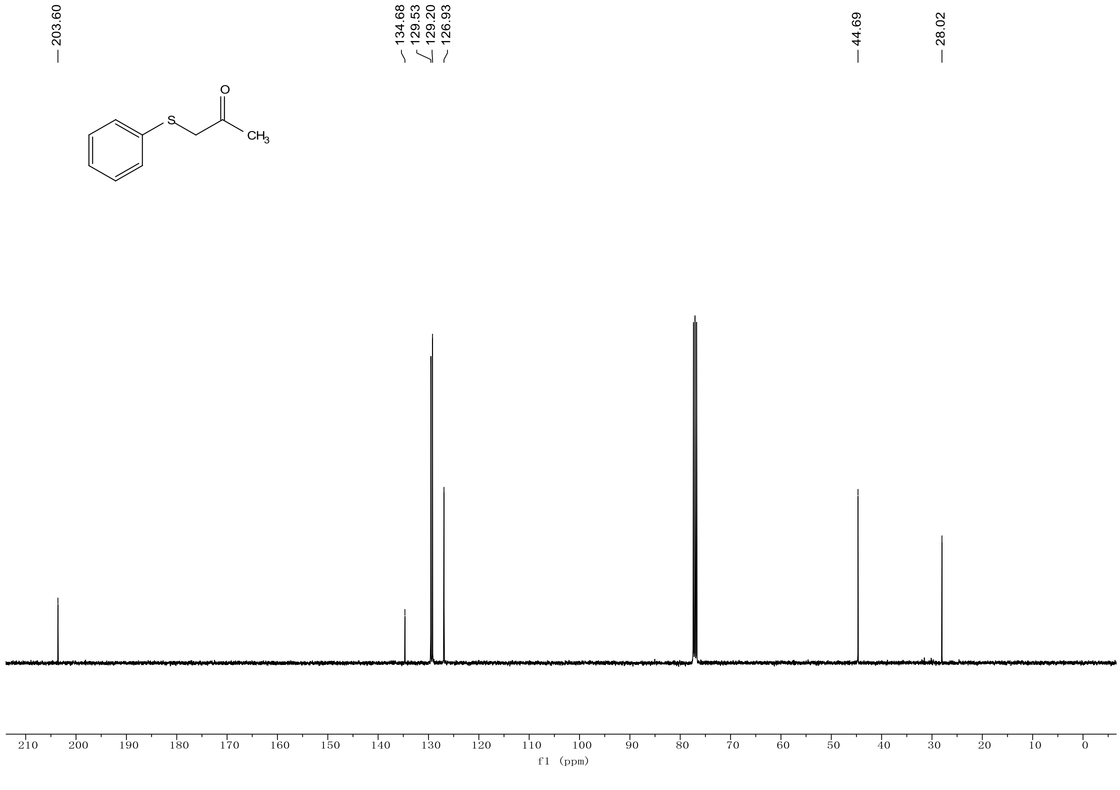

^{1}H NMR (400 MHz, CDCl$_3$) **2-(3-oxobutyl)isoindoline-1,3-dione (P42)**

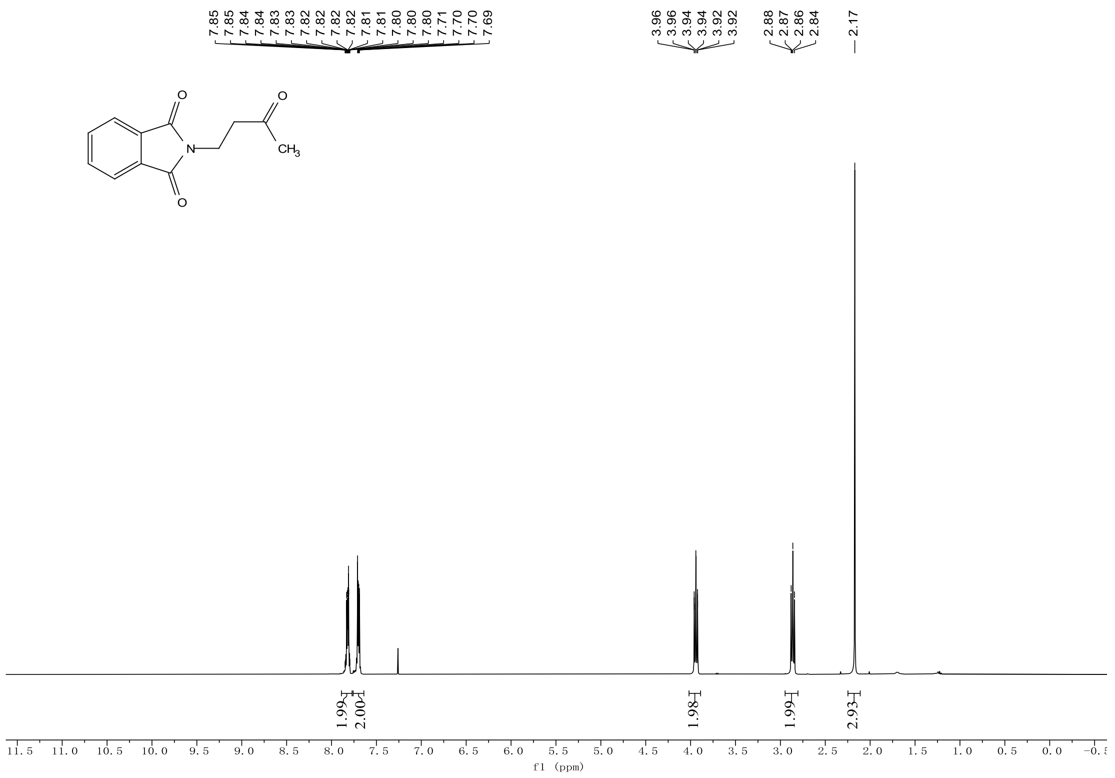

^{13}C NMR (101 MHz, $CDCl_3$) **2-(3-oxobutyl)isoindoline-1,3-dione (P42)**

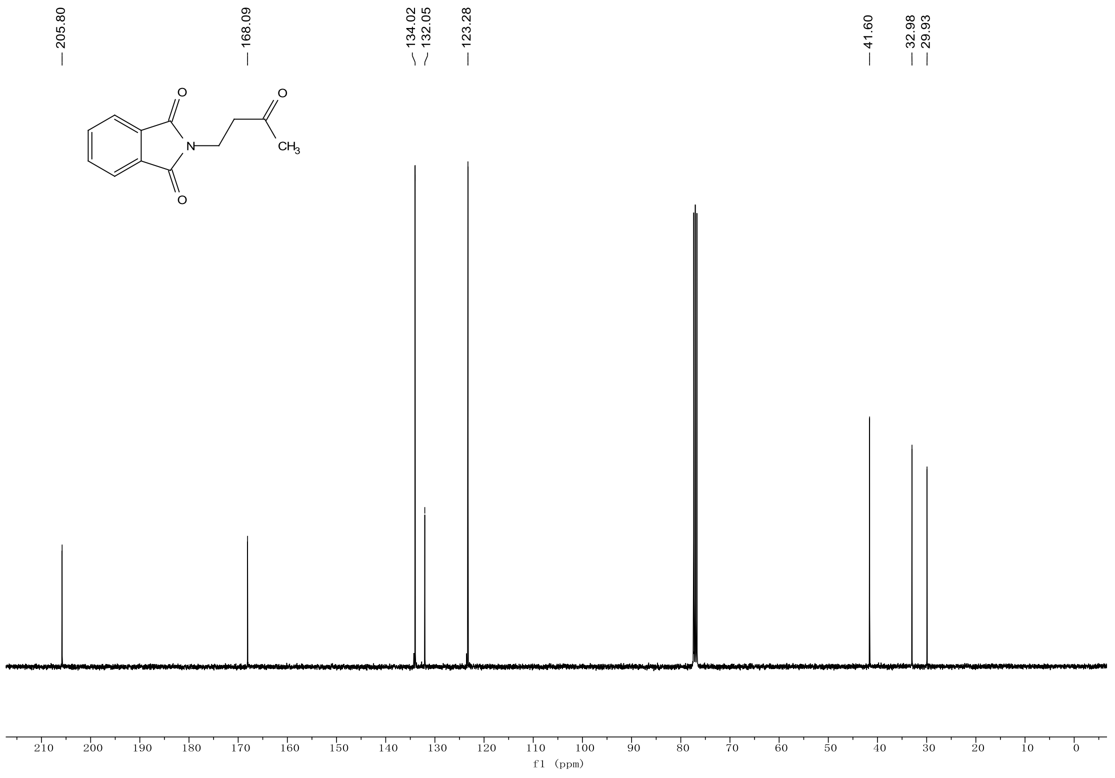

^{1}H NMR (400 MHz, $CDCl_3$) **1-(1*H*-indol-3-yl)propan-2-one (P43)**

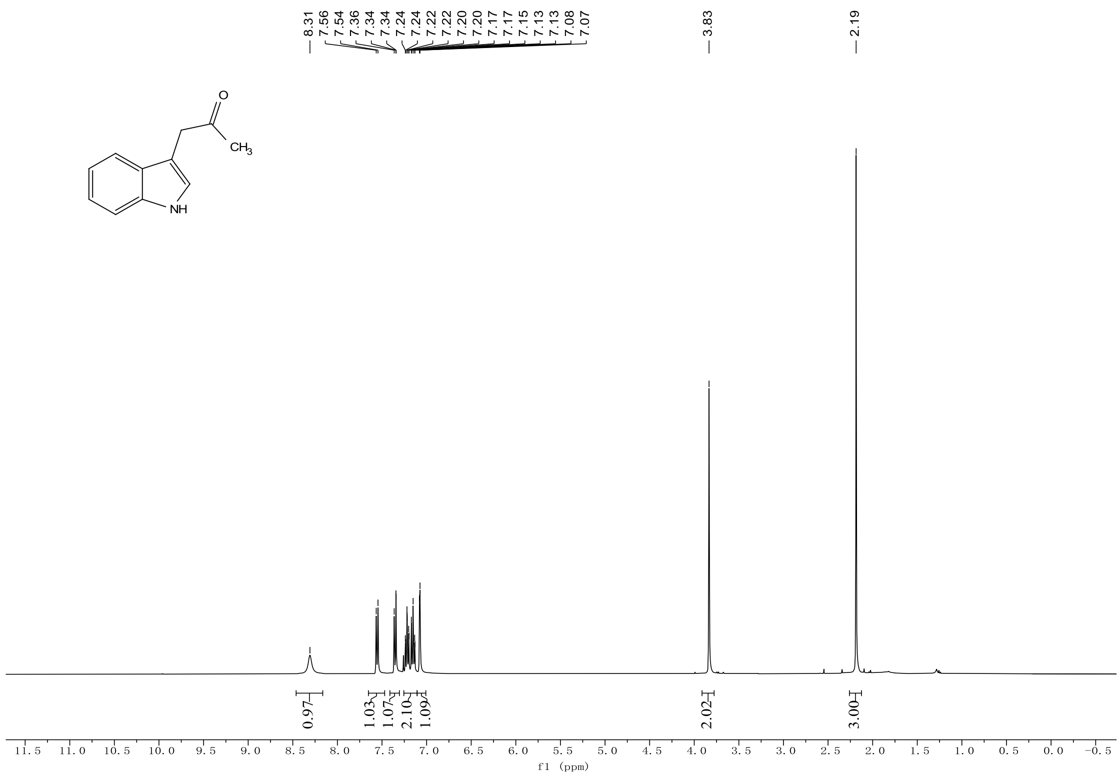

^{13}C NMR (101 MHz, $CDCl_3$) **1-(1*H*-indol-3-yl)propan-2-one (P43)**

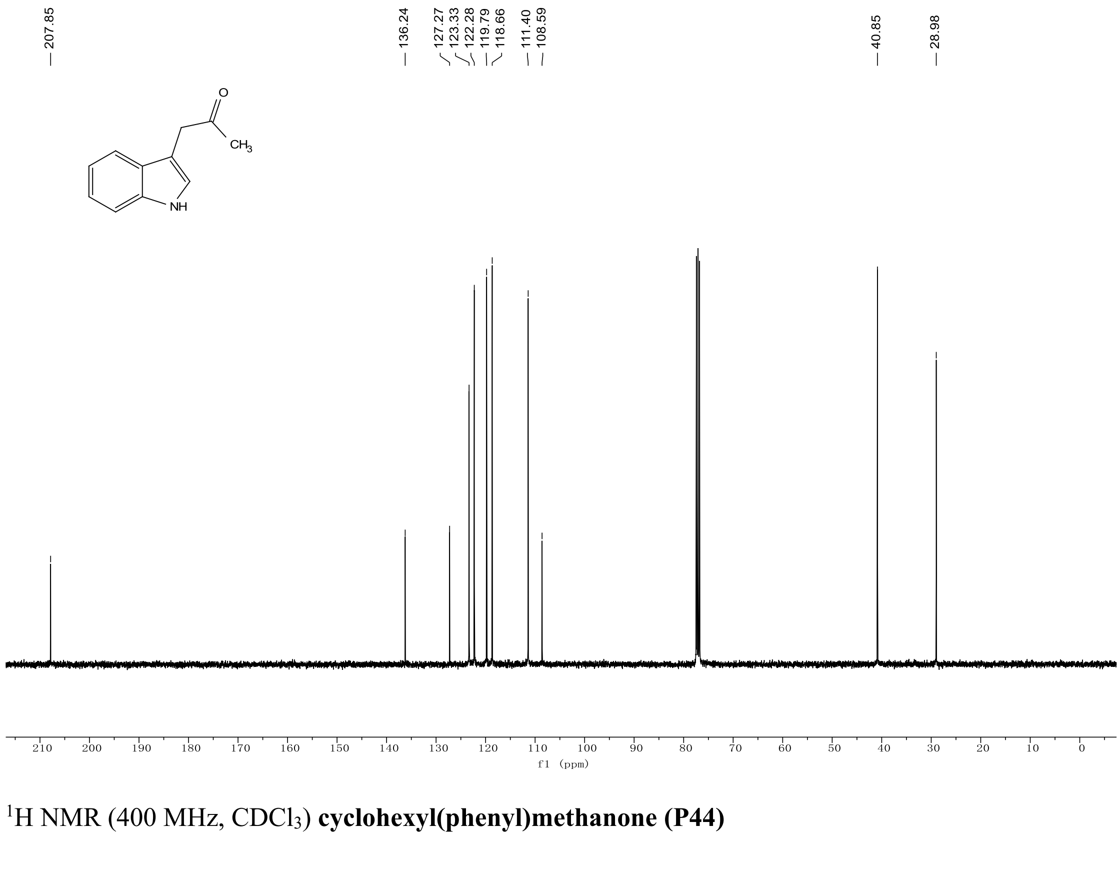

^{1}H NMR (400 MHz, $CDCl_3$) **cyclohexyl(phenyl)methanone (P44)**

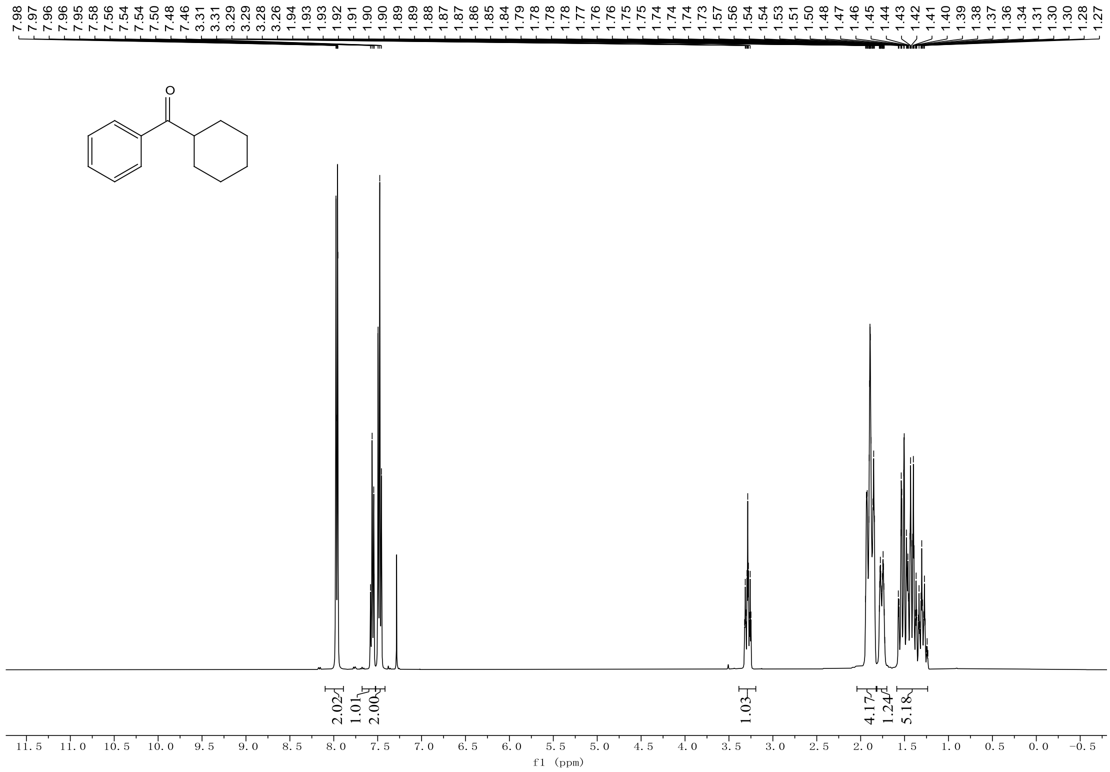

^{13}C NMR (101 MHz, $CDCl_3$) **cyclohexyl(phenyl)methanone (P44)**

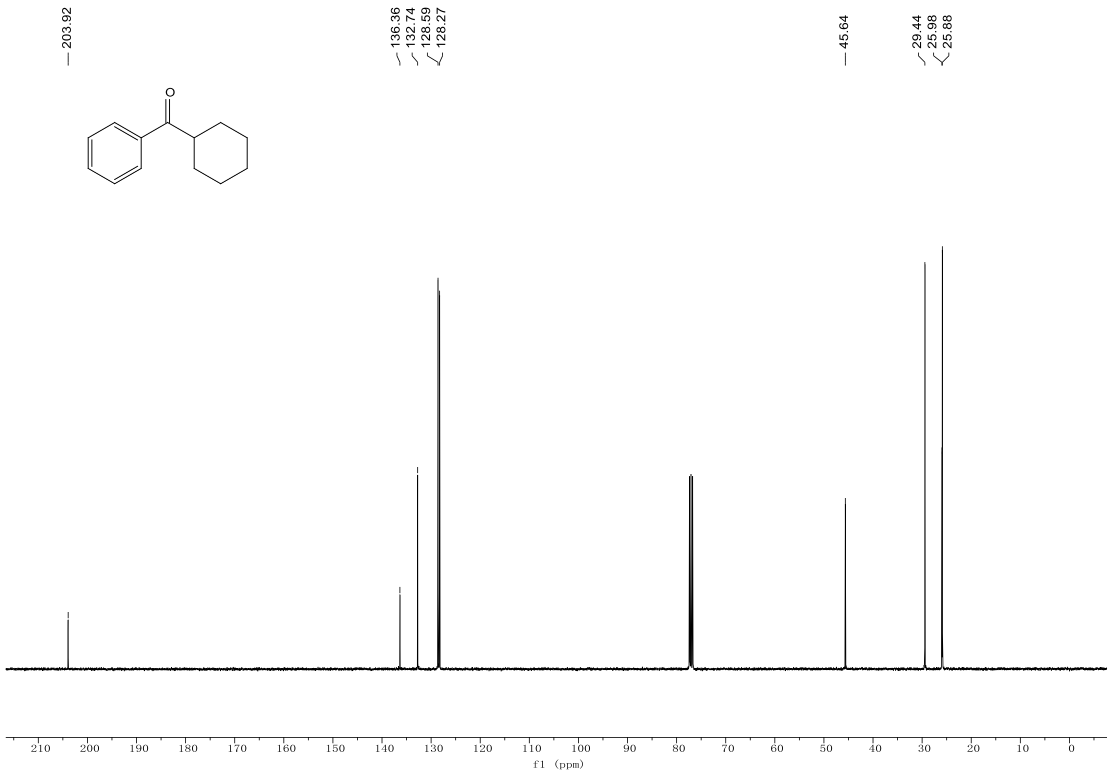

^{1}H NMR (400 MHz, $CDCl_3$) **cyclohexyl(naphthalen-2-yl)methanone (P45)**

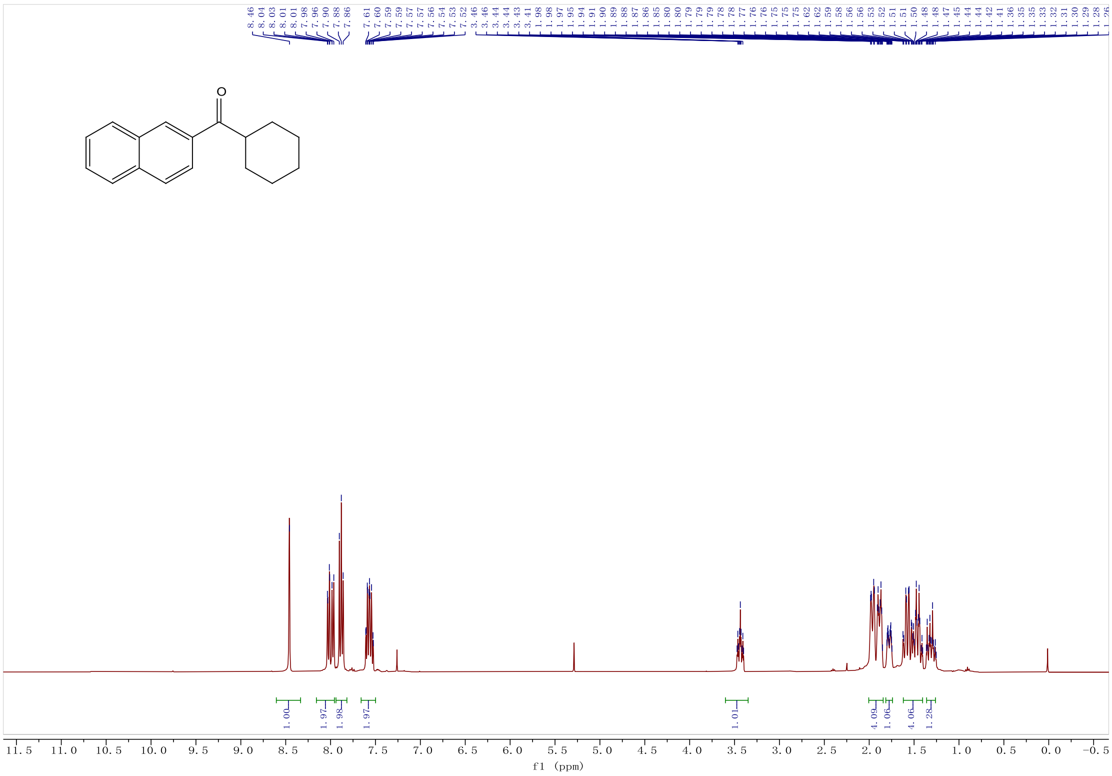

^{13}C NMR (101 MHz, $CDCl_3$) **cyclohexyl(naphthalen-2-yl)methanone (P45)**

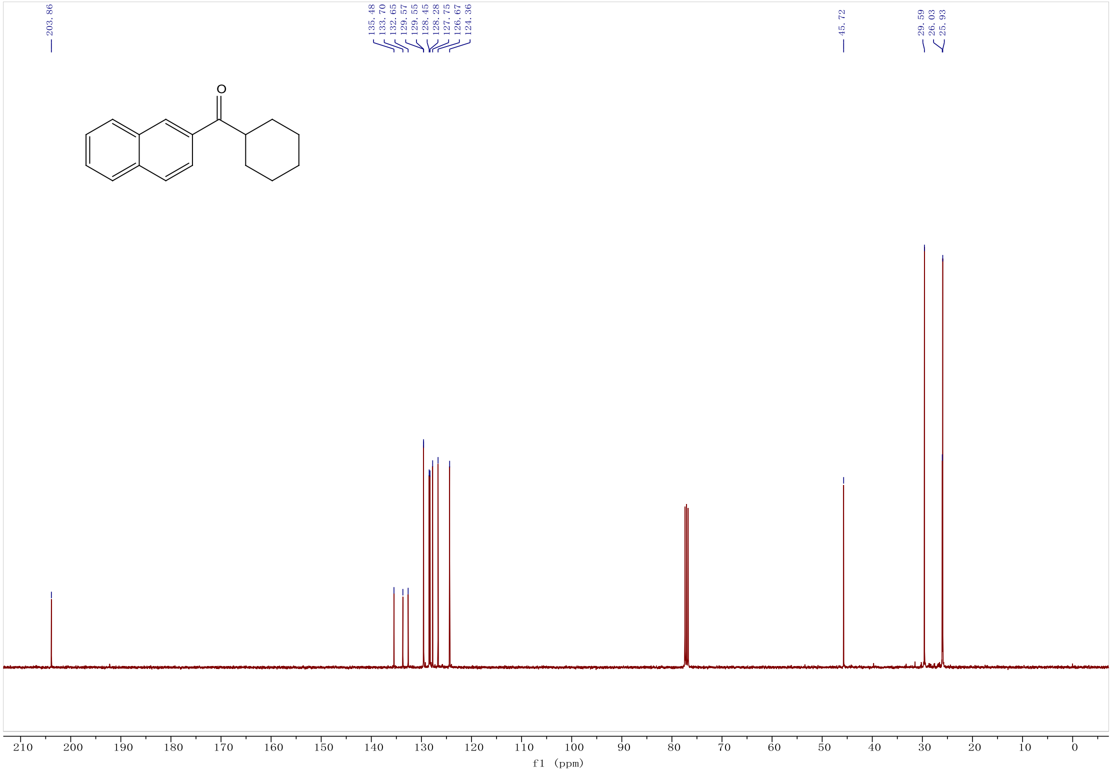

^{1}H NMR (400 MHz, $CDCl_3$) **cyclohexyl(4-(methylsulfonyl)phenyl)methanone (P46)**

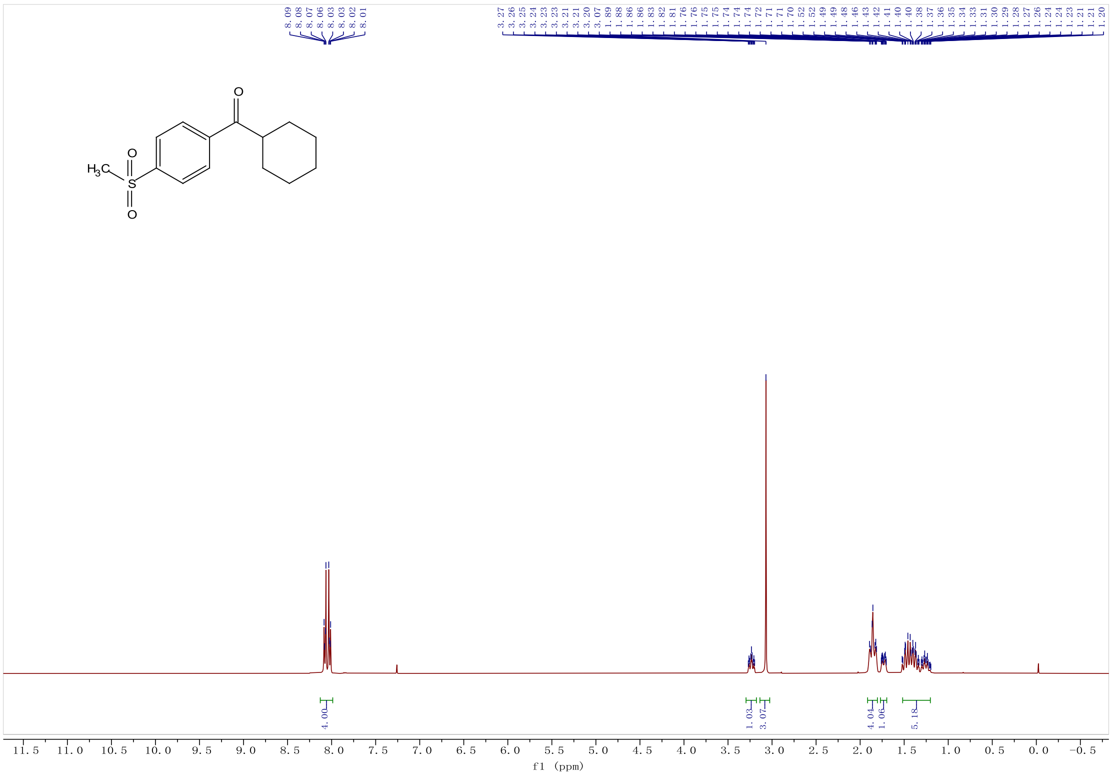

^{13}C NMR (101 MHz, $CDCl_3$) **cyclohexyl(4-(methylsulfonyl)phenyl)methanone (P46)**

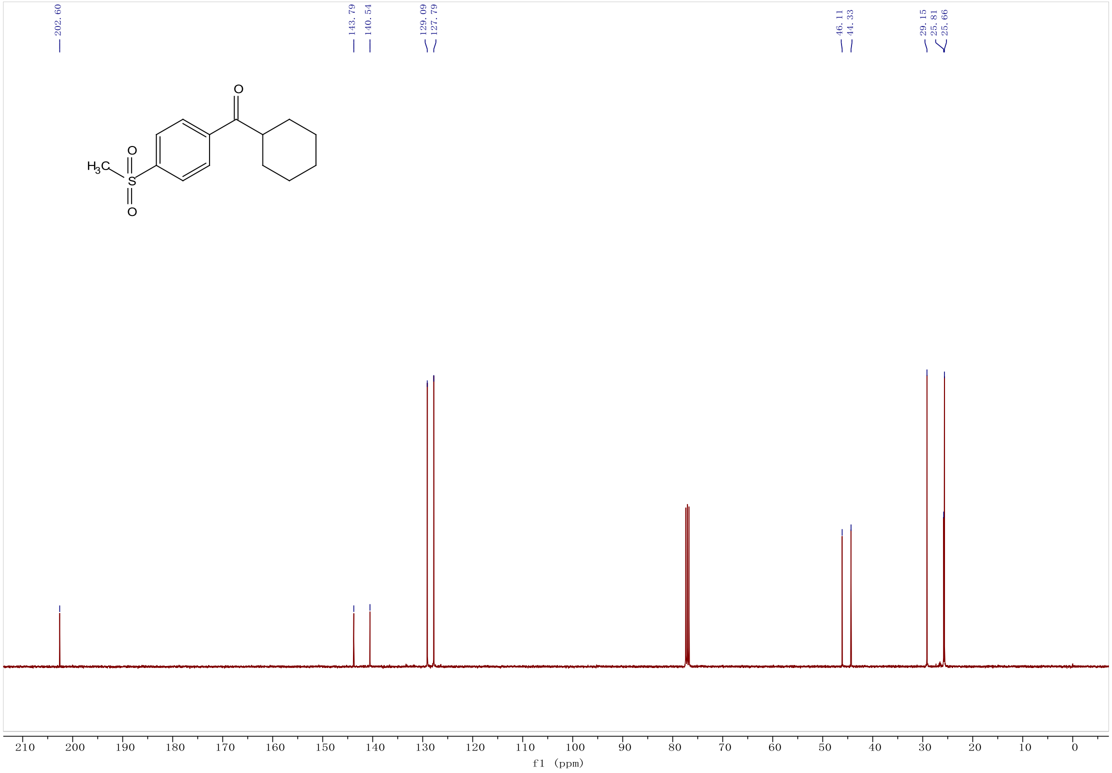

^{1}H NMR (400 MHz, $CDCl_3$) **1-phenoxydecan-2-one (P47)**

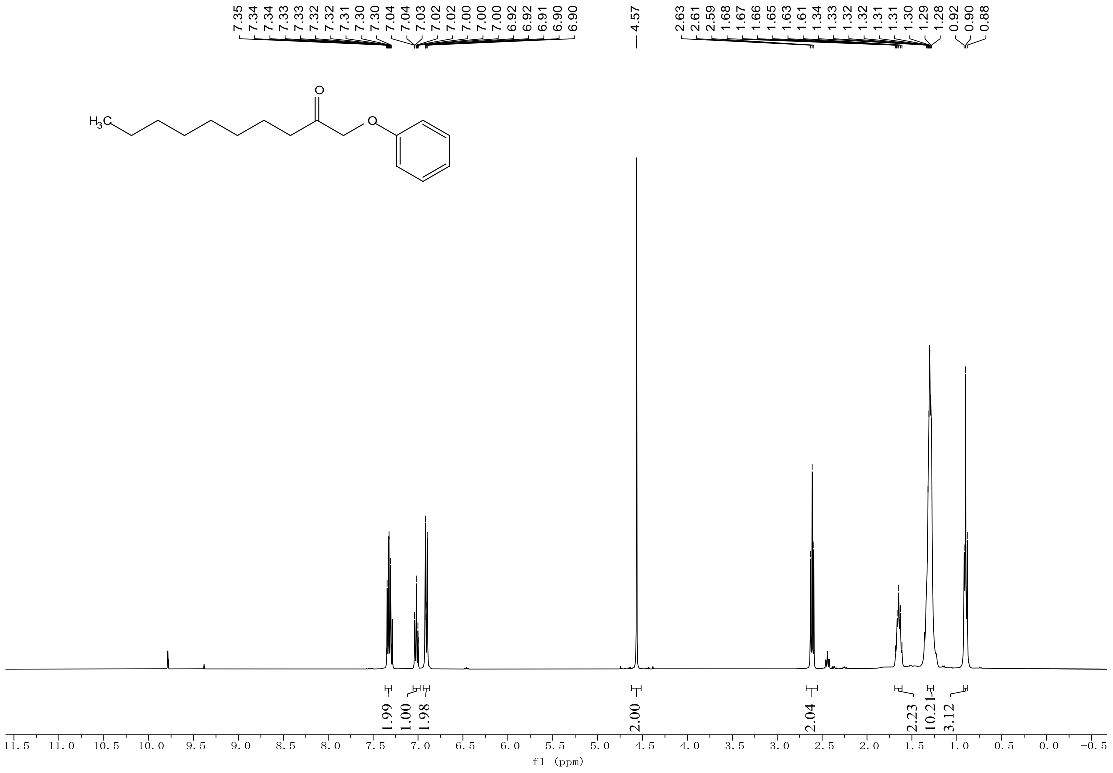

^{13}C NMR (101 MHz, $CDCl_3$) **1-phenoxydecan-2-one (P47)**

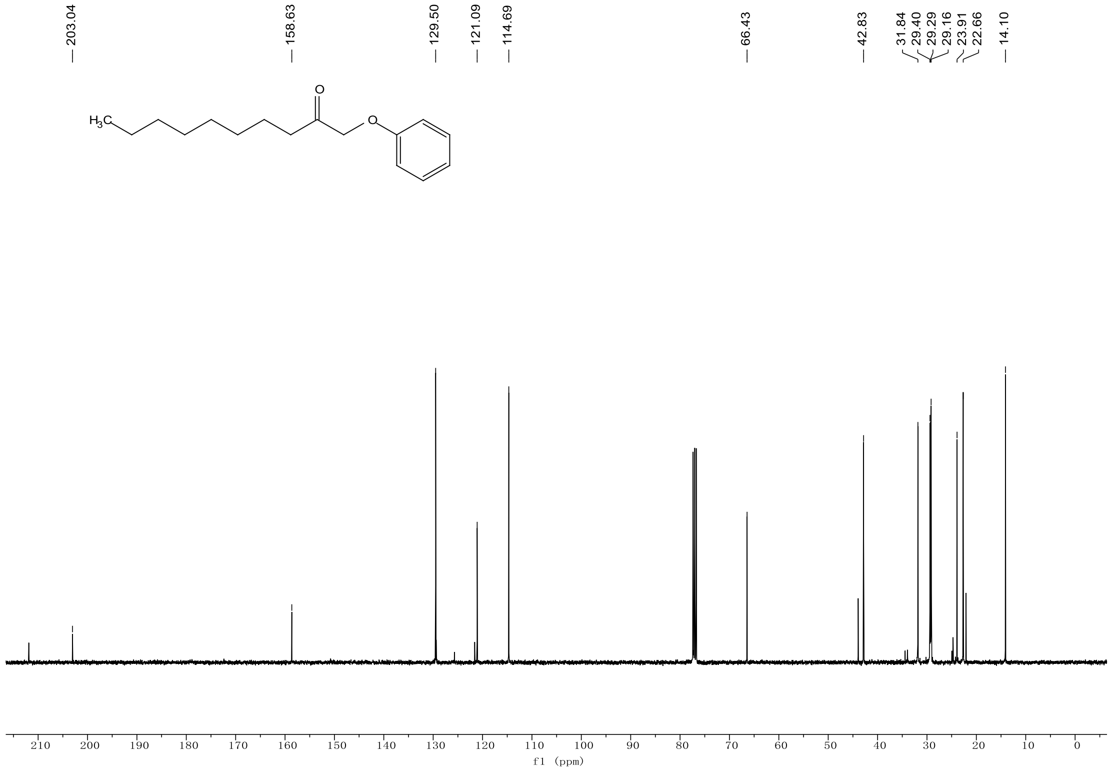

^{1}H NMR (400 MHz, $CDCl_3$) **1-phenoxydodecan-2-one (P48)**

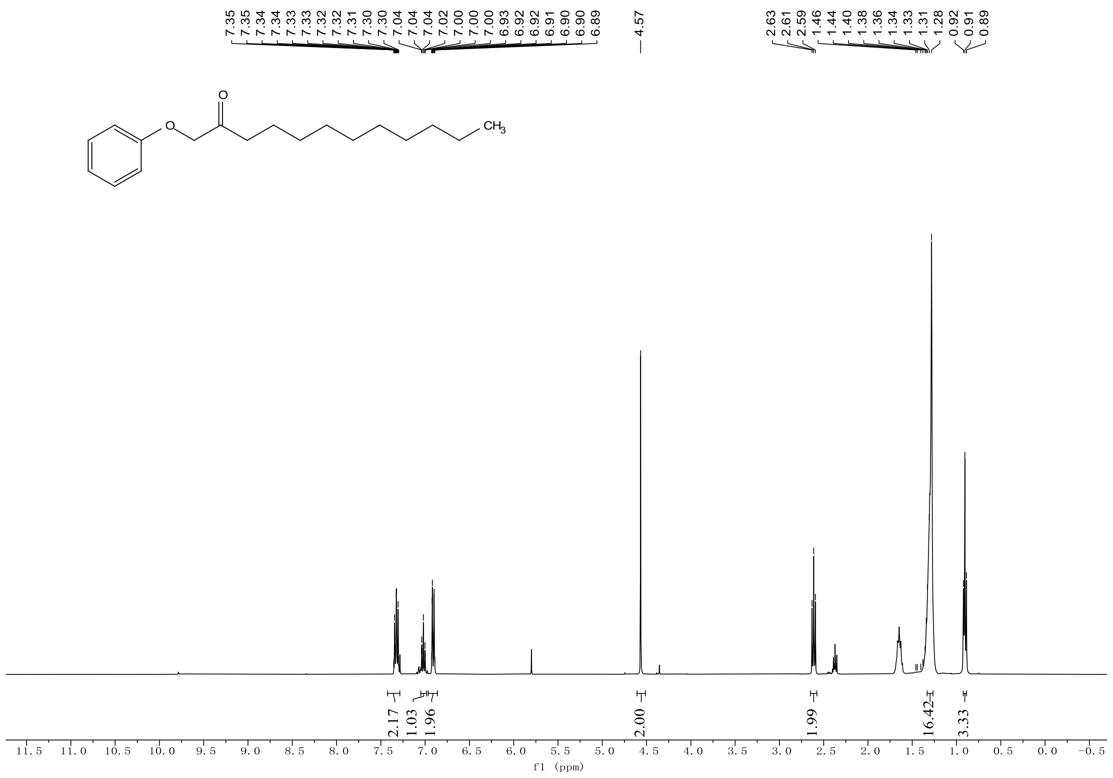

^{13}C NMR (101 MHz, $CDCl_3$) **1-phenoxydodecan-2-one (P48)**

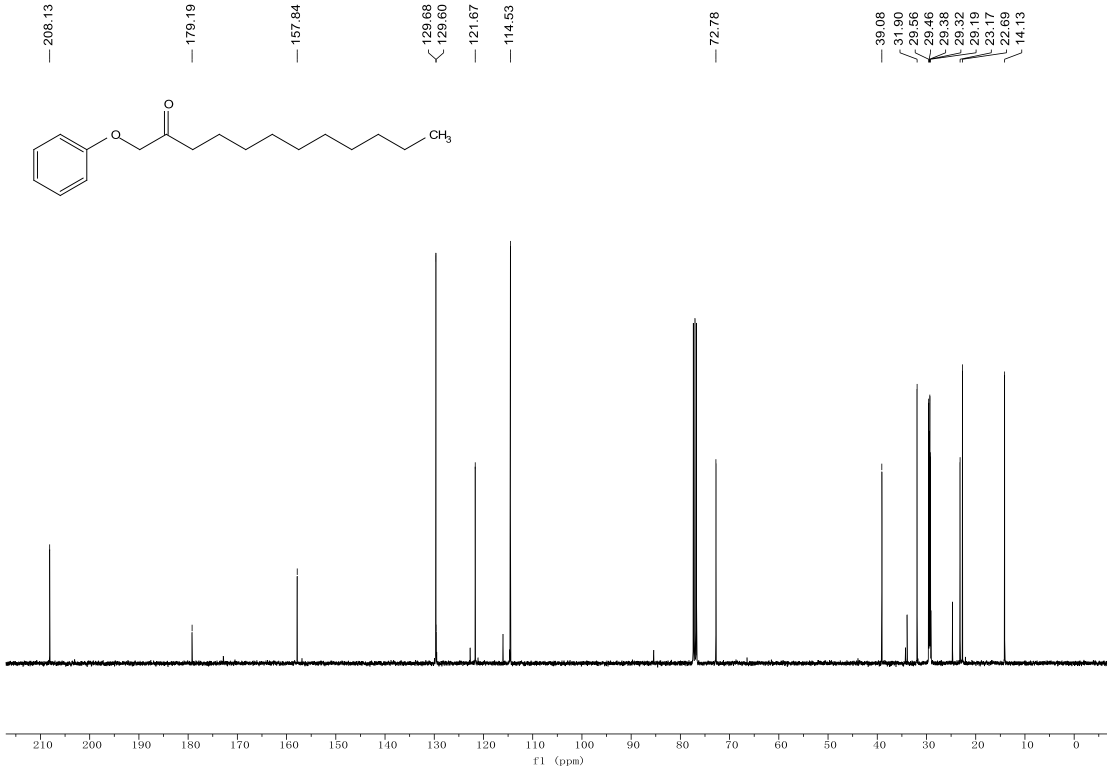

^{1}H NMR (400 MHz, $CDCl_3$) **4-(benzo[d][1,3]dioxol-5-yl)-3-methyl-1-phenoxybutan-2-one (P49)**

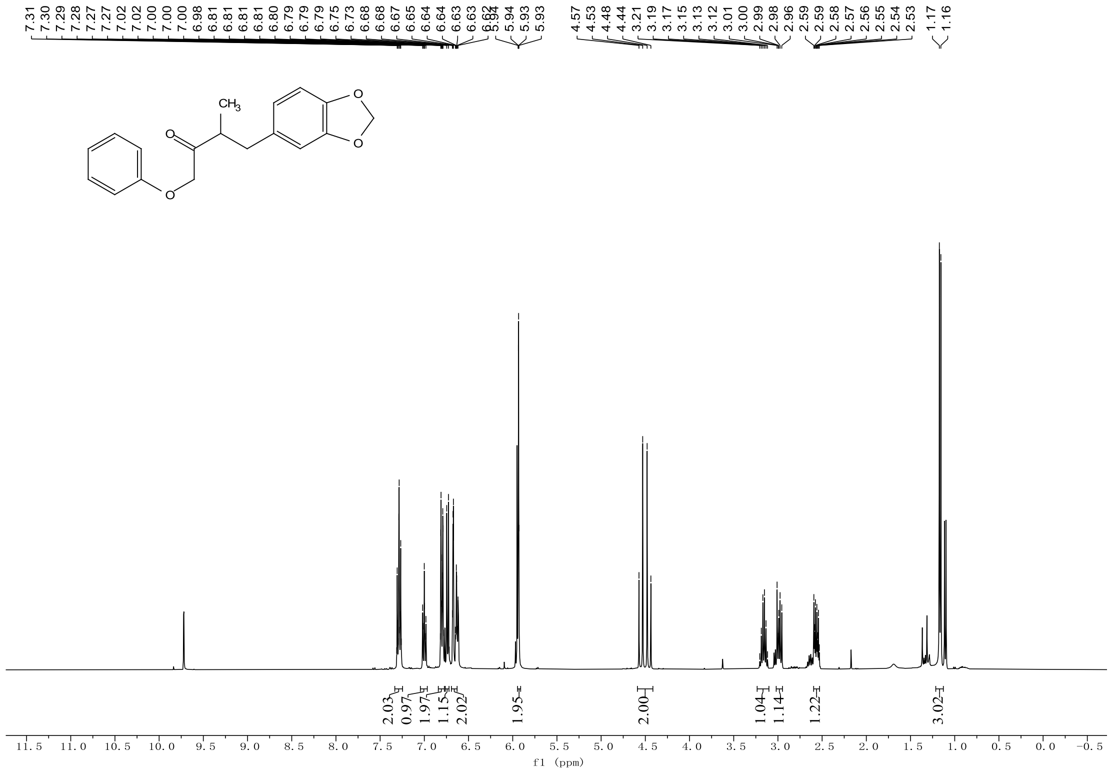

^{13}C NMR (101 MHz, $CDCl_3$) **4-(benzo[d][1,3]dioxol-5-yl)-3-methyl-1-phenoxybutan-2-one (P49)**

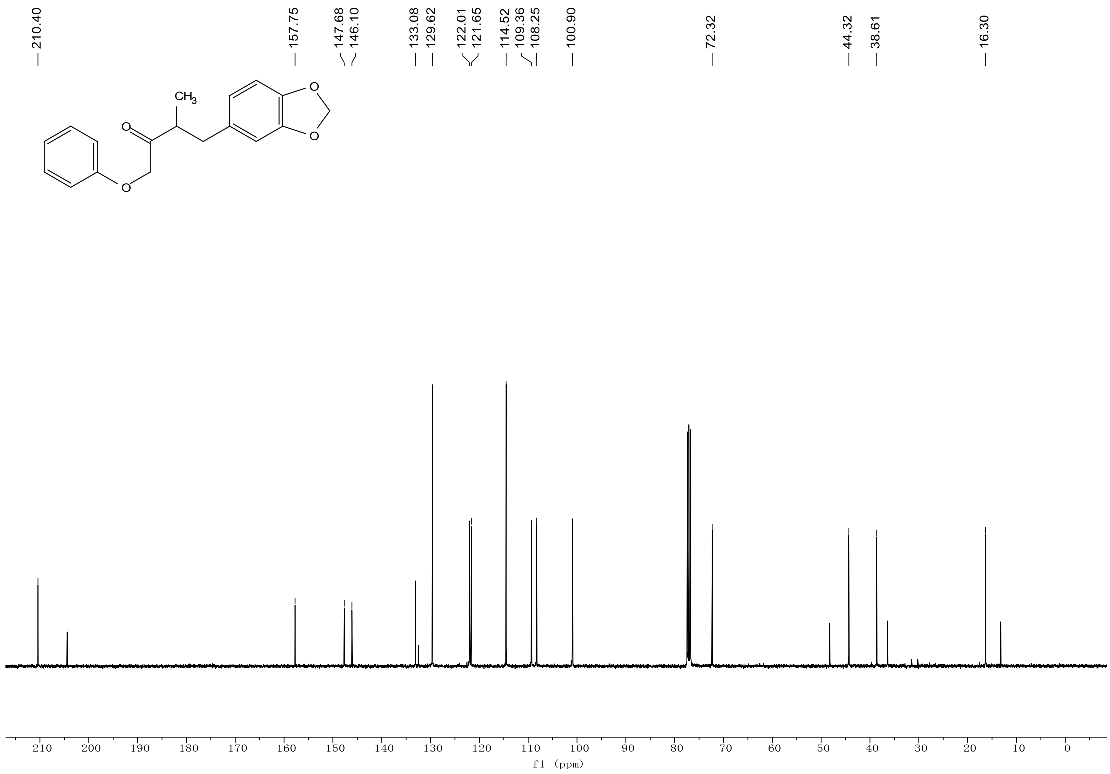

^{1}H NMR (400 MHz, $CDCl_3$) **3-(4-(2,2-dichlorocyclopropyl)phenoxy)-3-methylbutan-2-one (P50)**

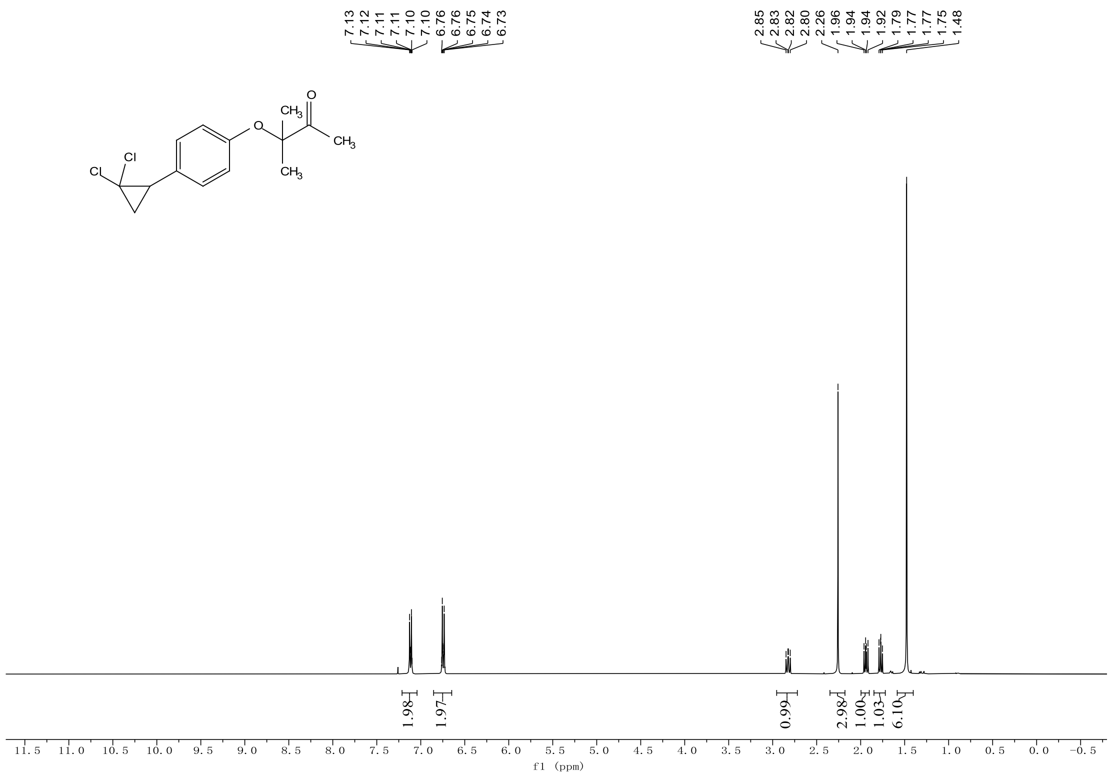

^{13}C NMR (101 MHz, $CDCl_3$) **3-(4-(2,2-dichlorocyclopropyl)phenoxy)-3-methylbutan-2-one (P50)**

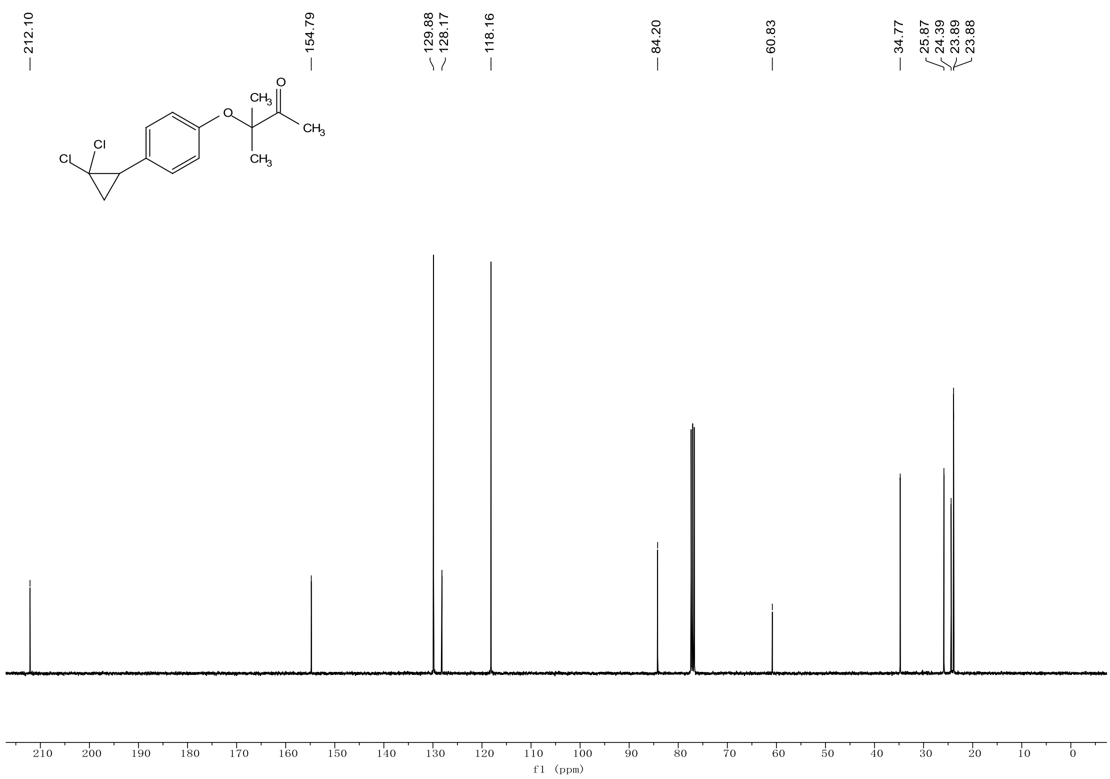

^{1}H NMR (400 MHz, $CDCl_3$) **(1S)-1-acetyl-4,7,7-trimethyl-2-oxabicyclo[2.2.1]heptan-3-one (P51)**

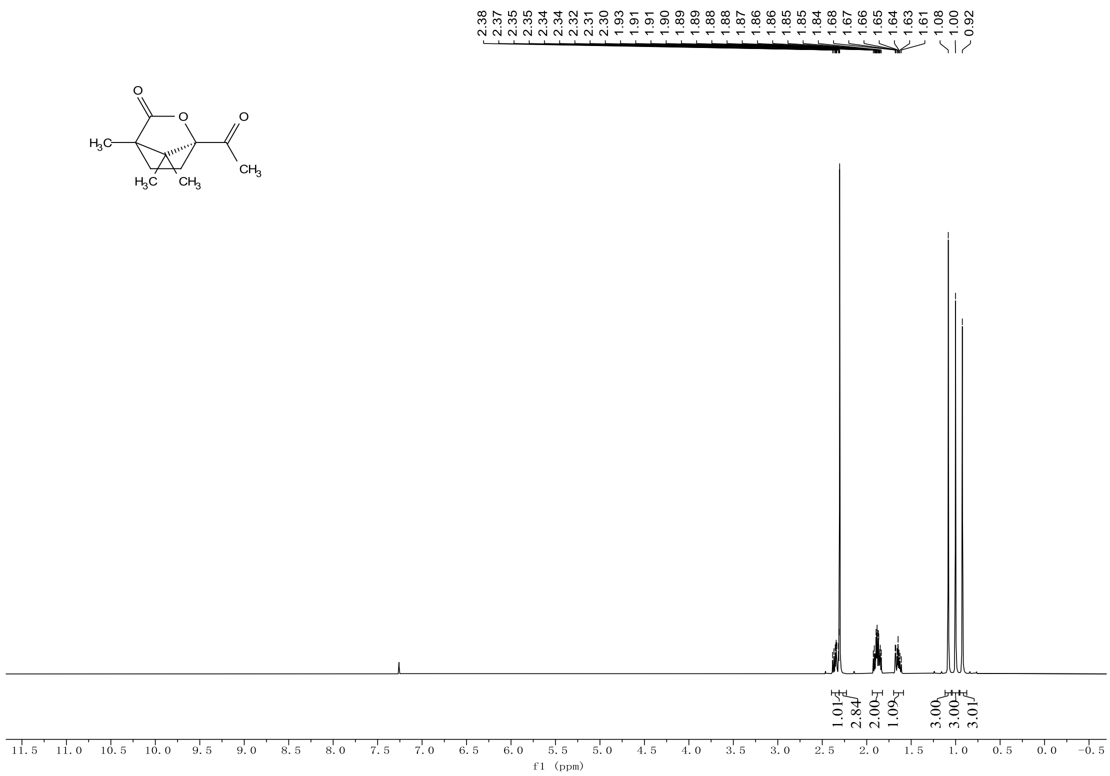

^{13}C NMR (101 MHz, $CDCl_3$) **(1S)-1-acetyl-4,7,7-trimethyl-2-oxabicyclo[2.2.1]heptan-3-one (P51)**

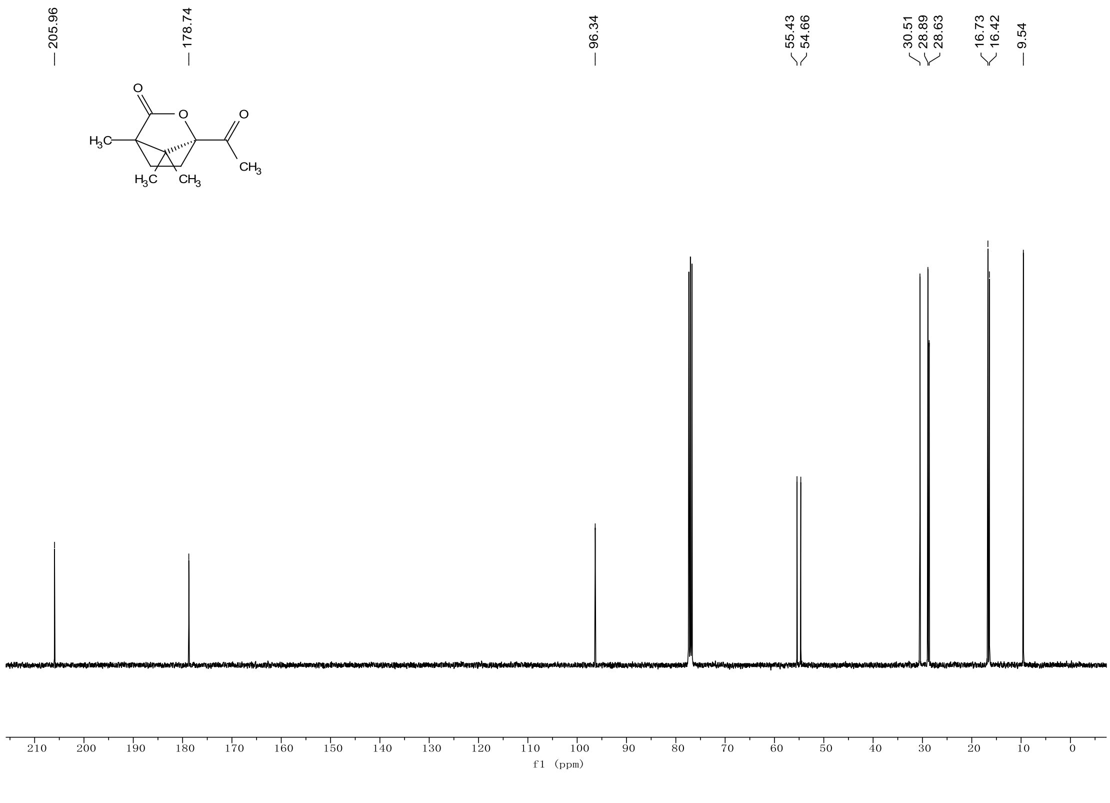

^{1}H NMR (400 MHz, $CDCl_3$) **tridecan-2-one (P52)**

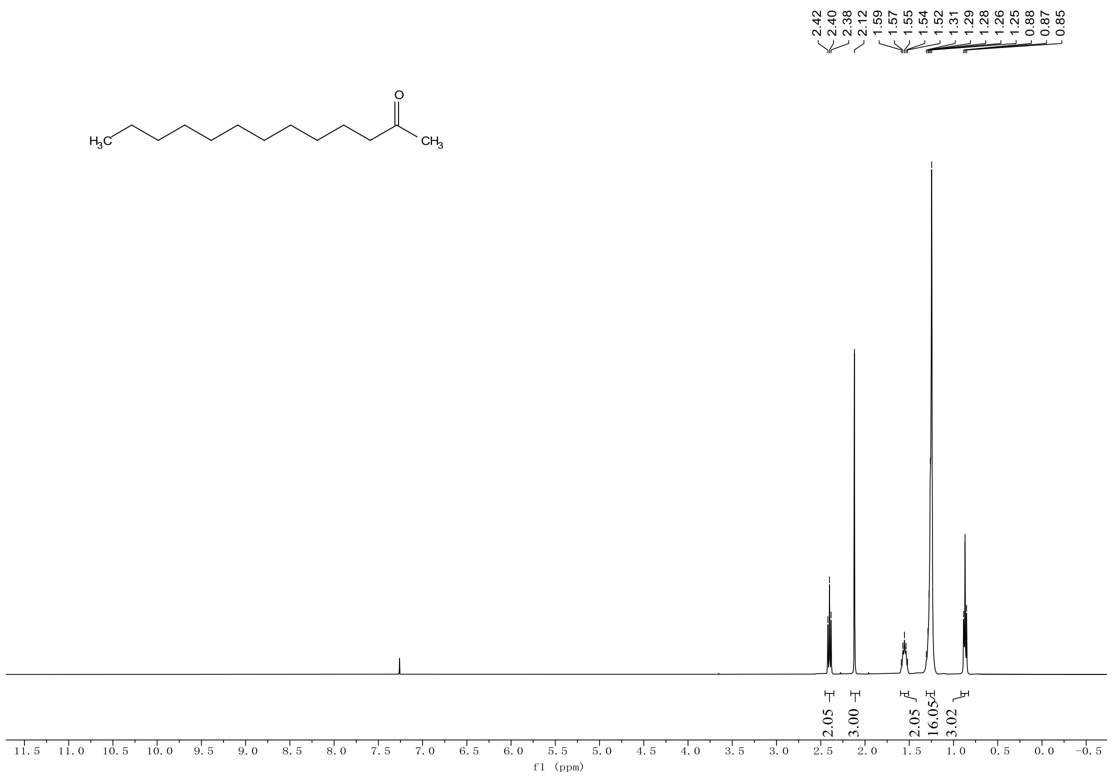

^{13}C NMR (101 MHz, $CDCl_3$) **tridecan-2-one (P52)**

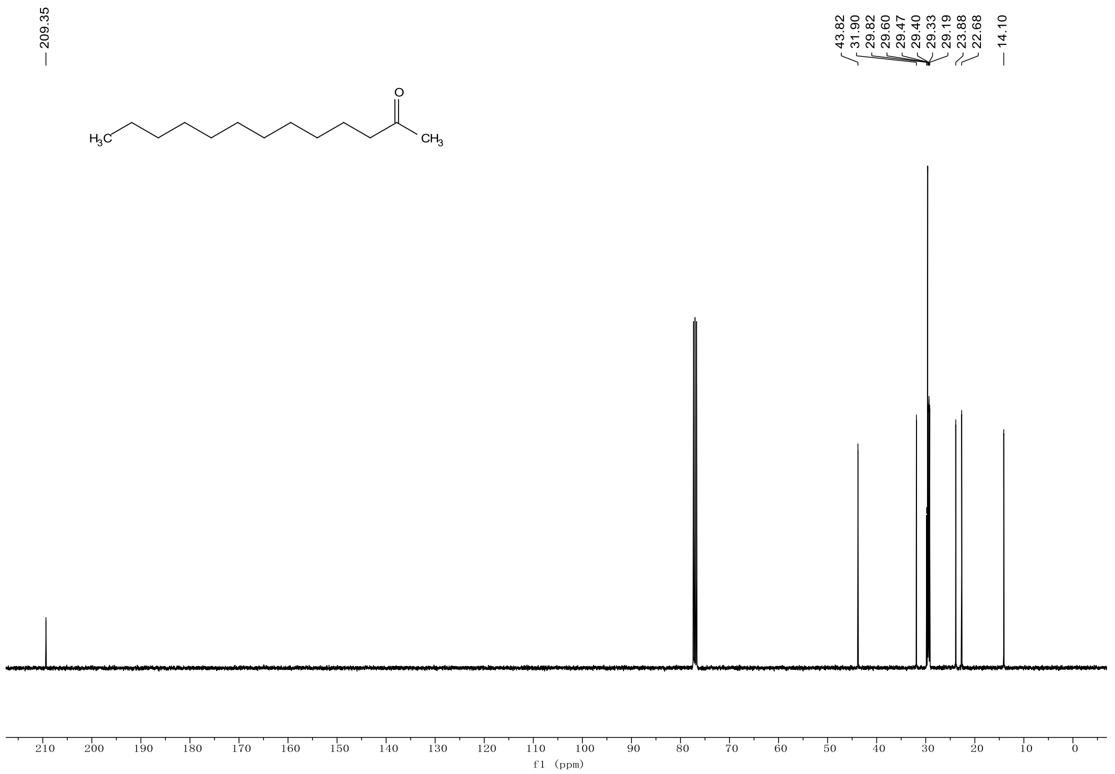

^{1}H NMR (400 MHz, $CDCl_3$) **3-(2,4-dichlorophenoxy)butan-2-one (P53)**

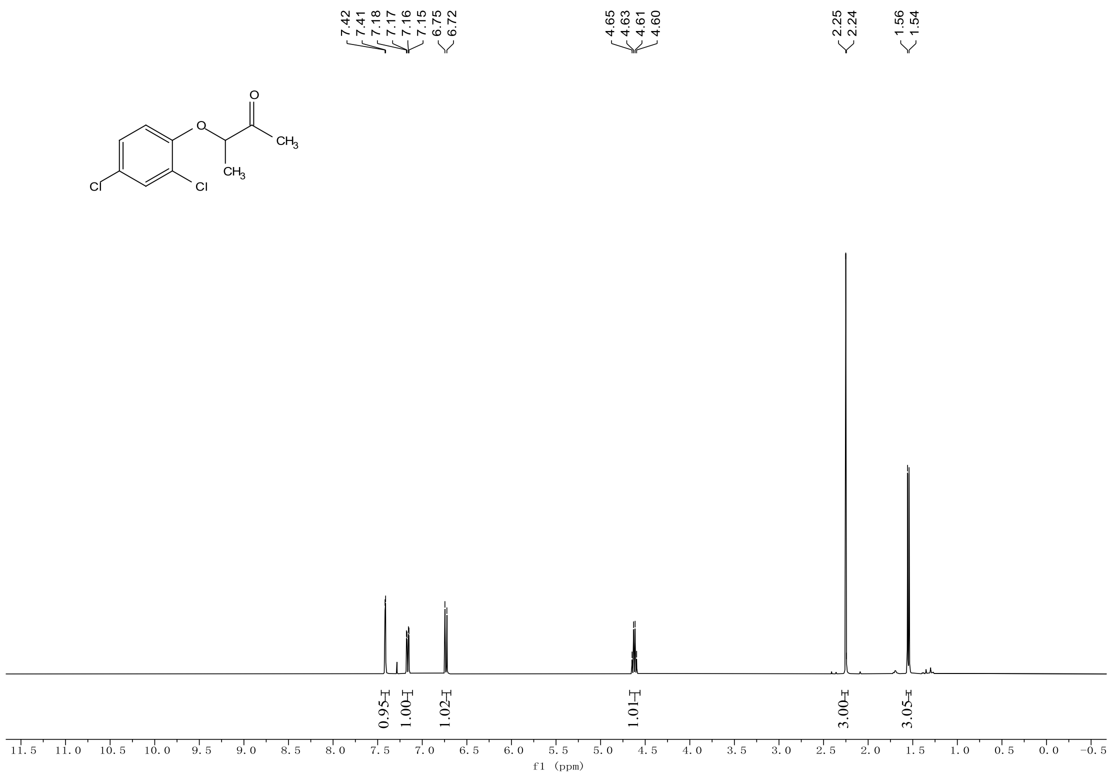

^{13}C NMR (101 MHz, $CDCl_3$) **3-(2,4-dichlorophenoxy)butan-2-one (P53)**

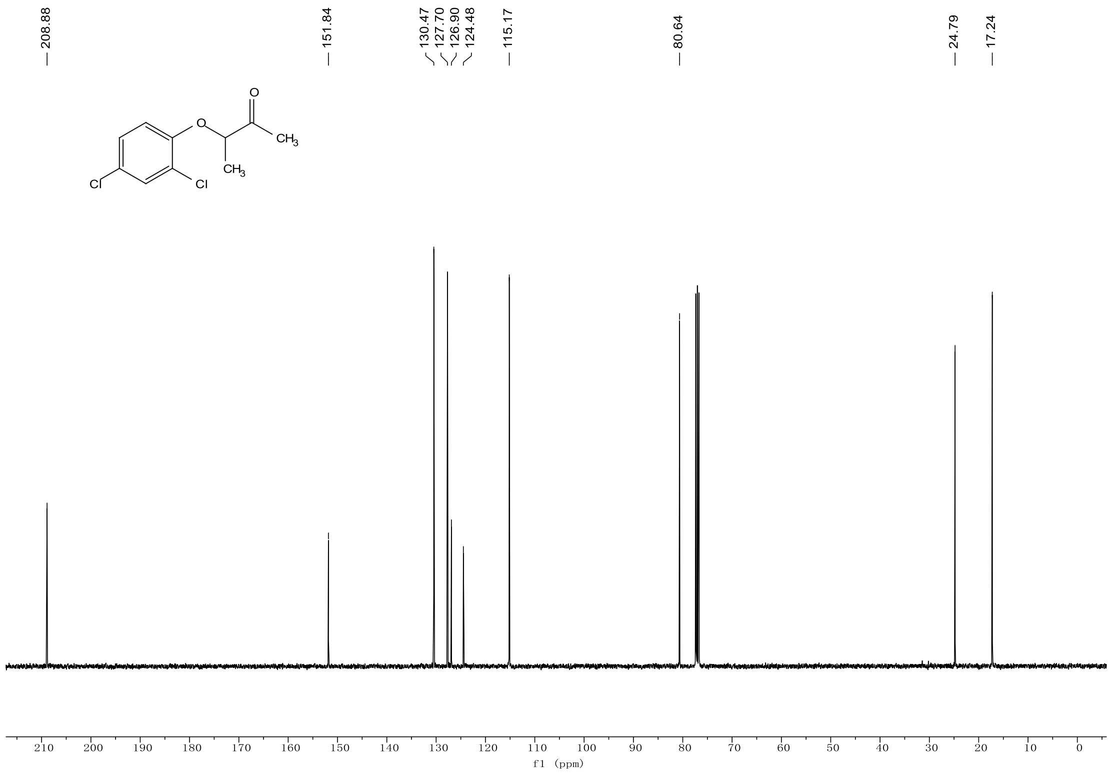

^{1}H NMR (400 MHz, $CDCl_3$) **3-(6-methoxynaphthalen-2-yl)butan-2-one (P54)**

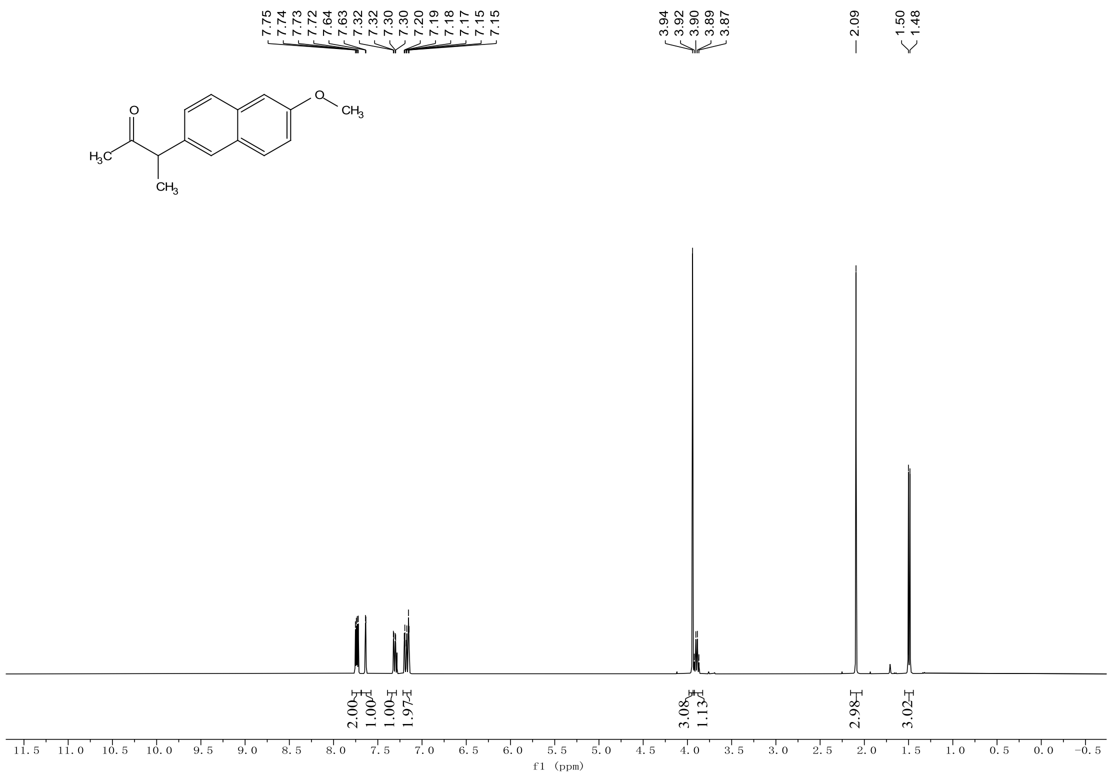

^{13}C NMR (101 MHz, $CDCl_3$) **3-(6-methoxynaphthalen-2-yl)butan-2-one (P54)**

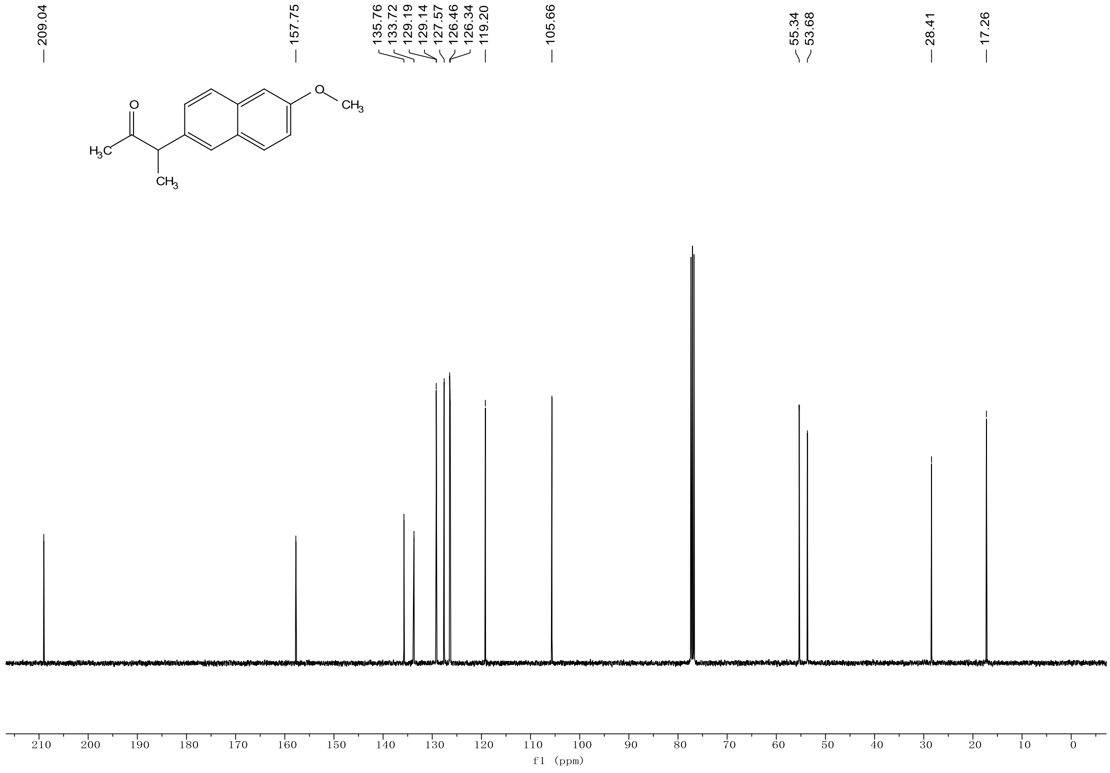

^{1}H NMR (400 MHz, $CDCl_3$) **3-(4-isobutylphenyl)butan-2-one (P55)**

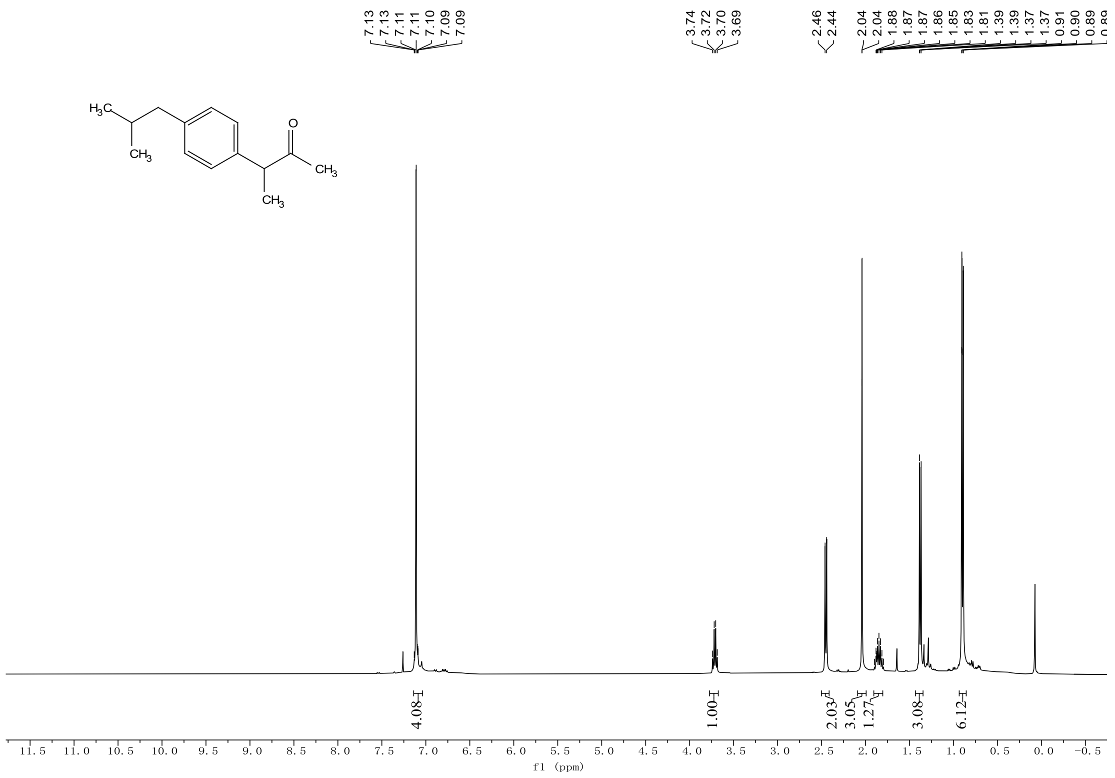

^{13}C NMR (101 MHz, $CDCl_3$) **3-(4-isobutylphenyl)butan-2-one (P55)**

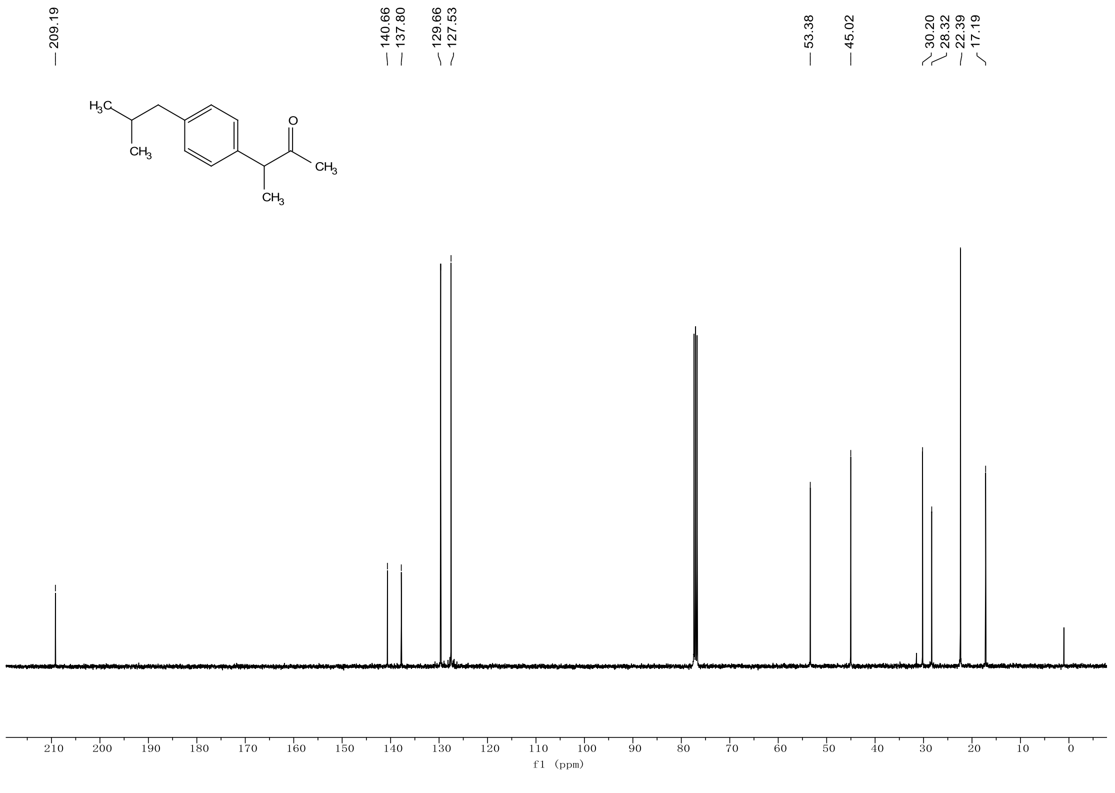

^{1}H NMR (400 MHz, $CDCl_3$) **3-(3-benzoylphenyl)butan-2-one (P56)**

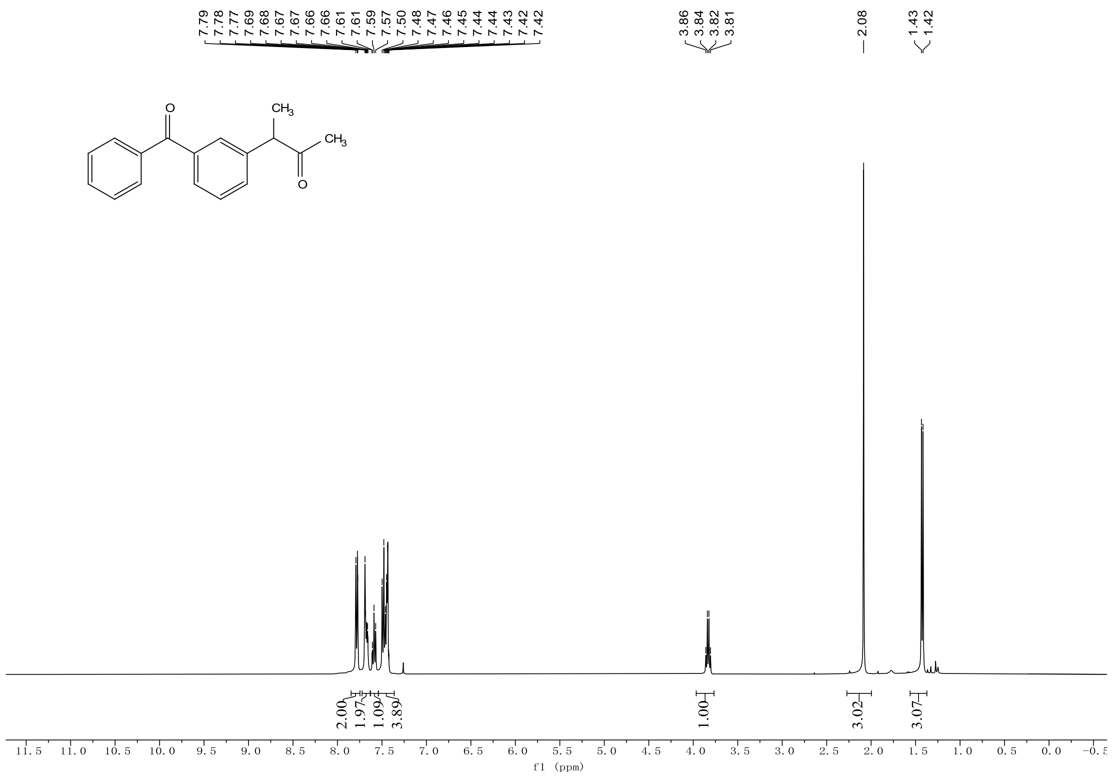

^{13}C NMR (101 MHz, $CDCl_3$) **3-(3-benzoylphenyl)butan-2-one (P56)**

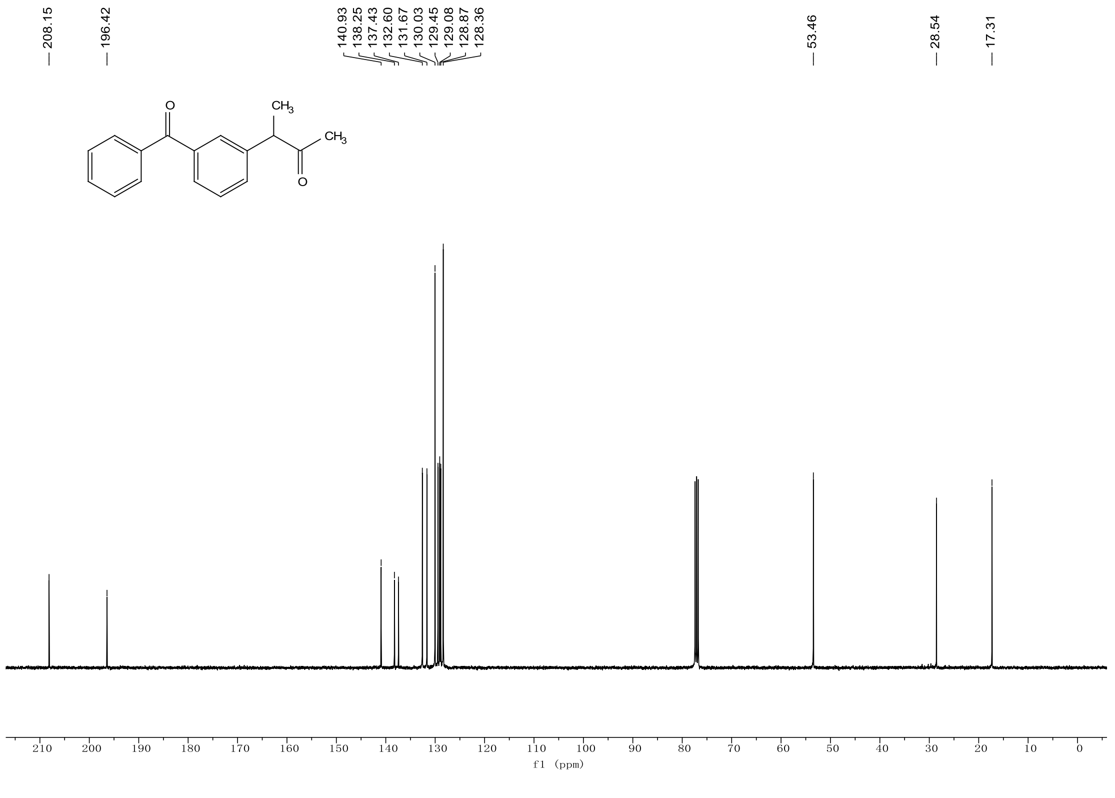

^{1}H NMR (400 MHz, $CDCl_3$) **22-(4-(3-oxobutan-2-yl)benzyl)cyclopentan-1-one (P57)**

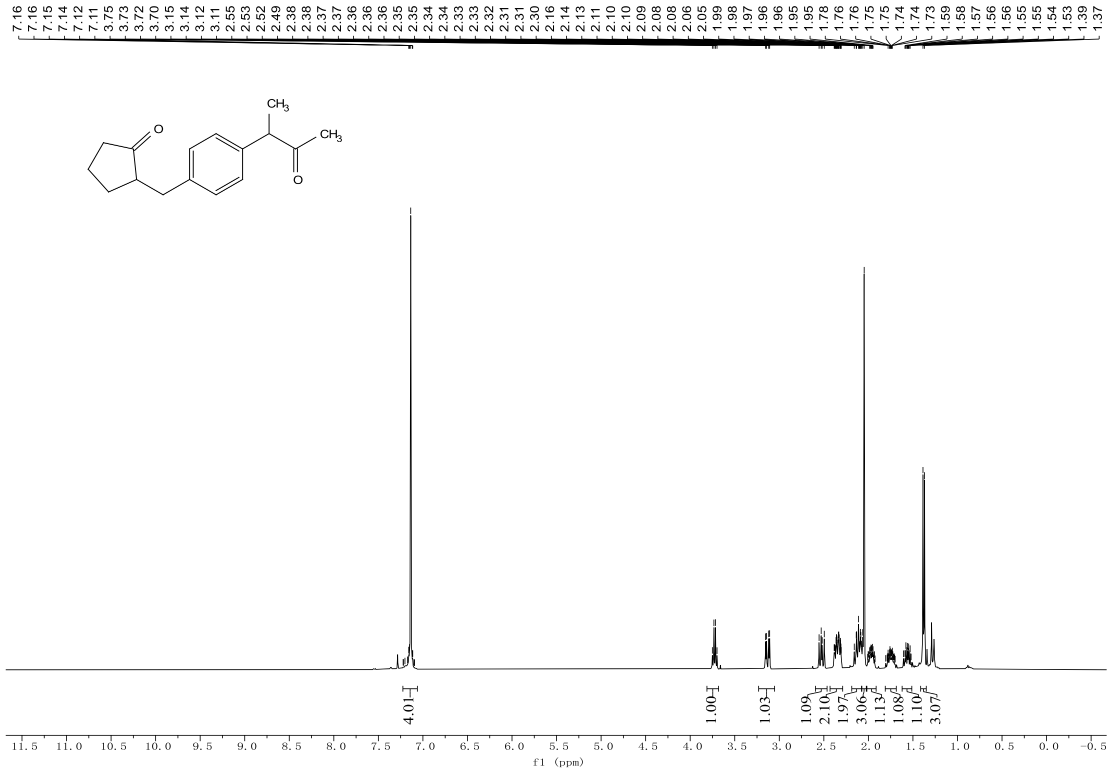

^{13}C NMR (101 MHz, $CDCl_3$) **2-(4-(3-oxobutan-2-yl)benzyl)cyclopentan-1-one (P57)**

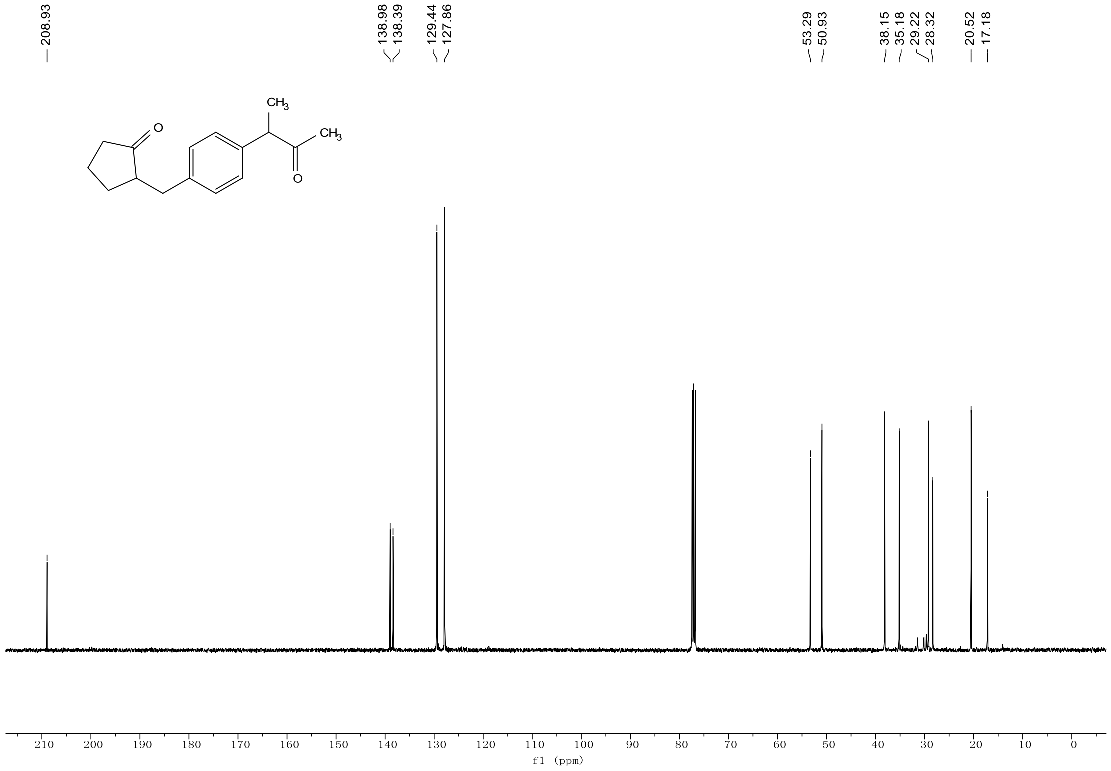

^{1}H NMR (400 MHz, $CDCl_3$) **1-([1,1'-biphenyl]-4-yl)pentane-1,4-dione (P58)**

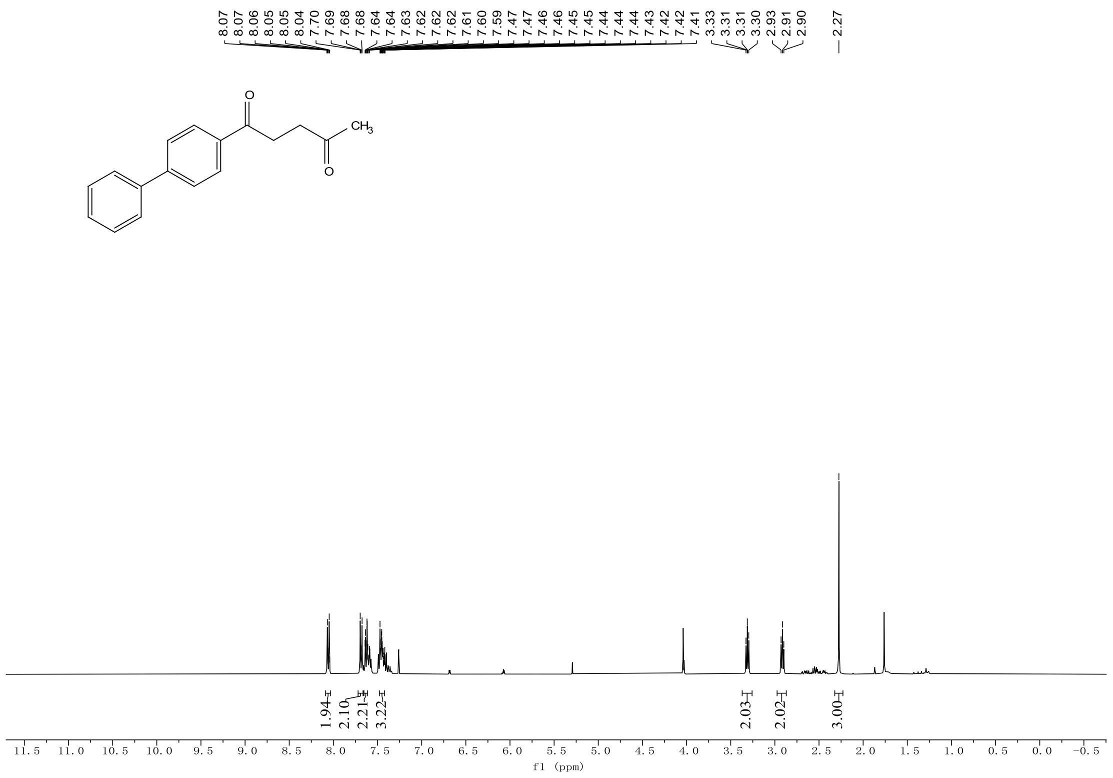

^{13}C NMR (101 MHz, $CDCl_3$) **1-([1,1'-biphenyl]-4-yl)pentane-1,4-dione (P58)**

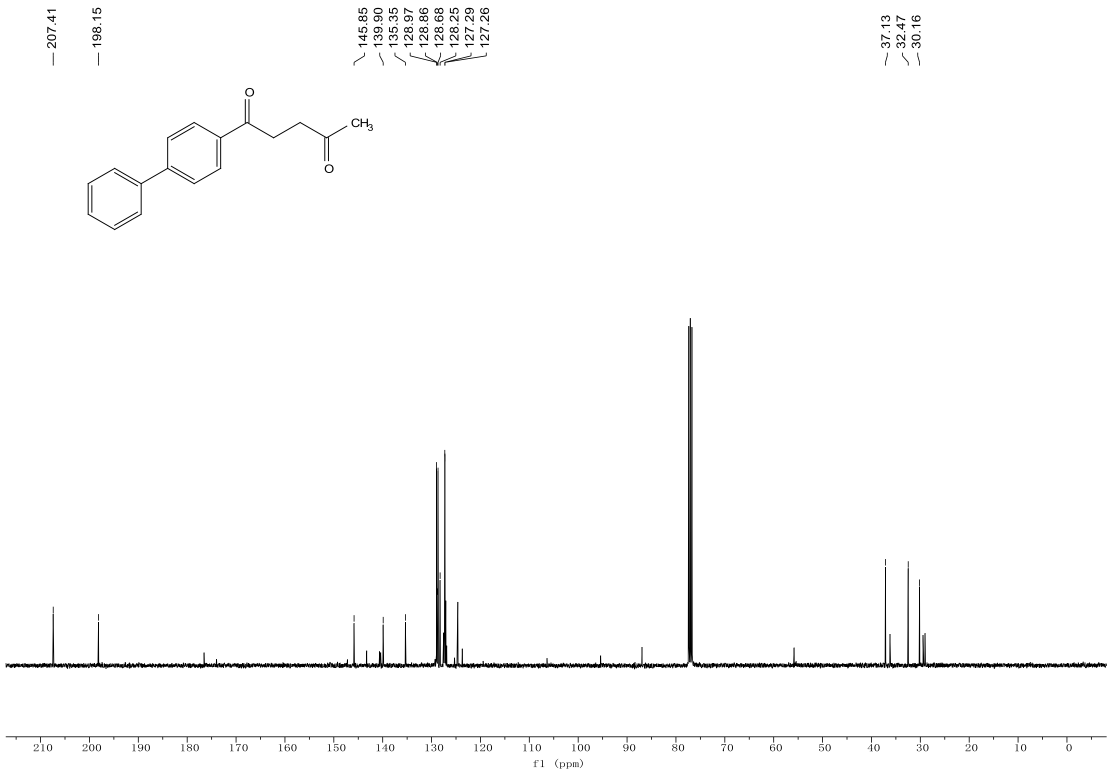

^{1}H NMR (400 MHz, $CDCl_3$) **3-(2-fluoro-[1,1'-biphenyl]-4-yl)butan-2-one (P59)**

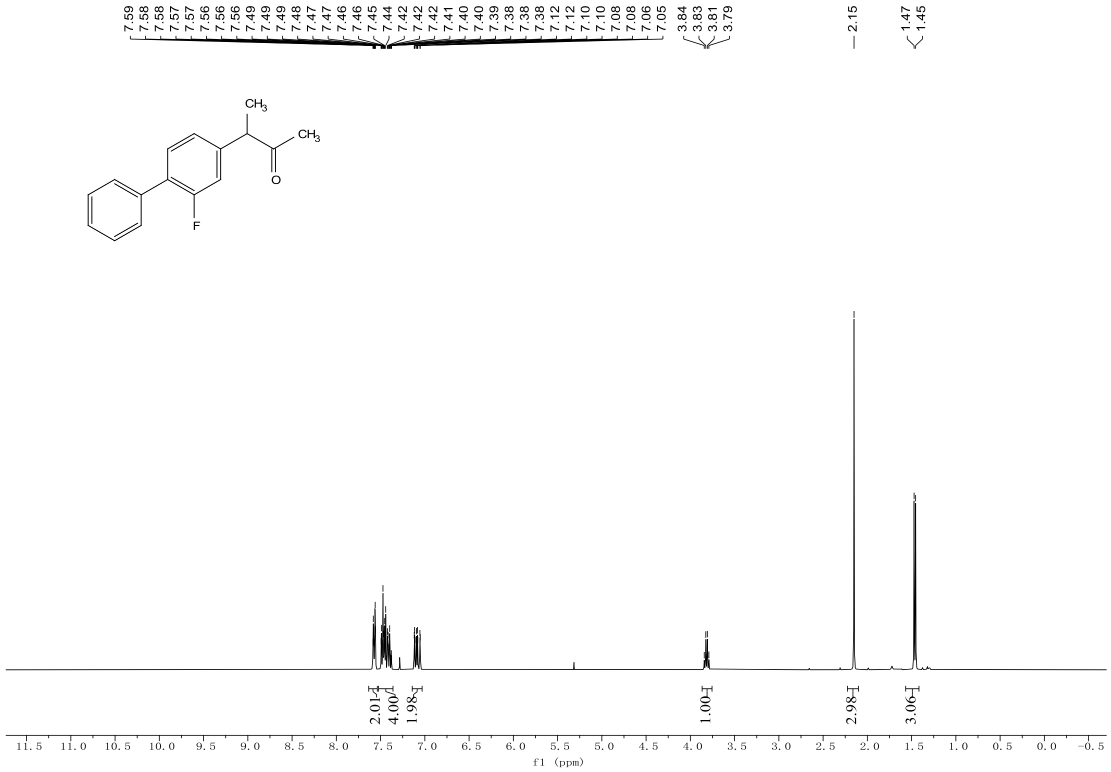

^{13}C NMR (101 MHz, $CDCl_3$) **3-(2-fluoro-[1,1'-biphenyl]-4-yl)butan-2-one (P59)**

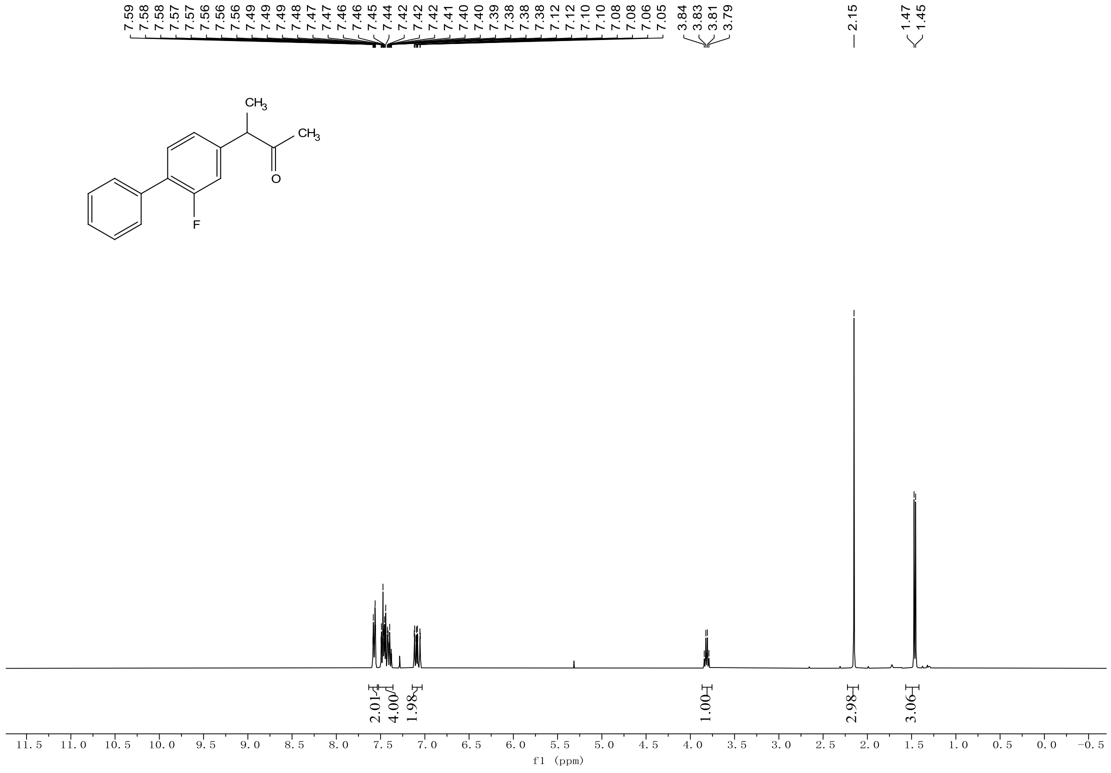

^{1}H NMR (400 MHz, $CDCl_3$) **2-(2-oxopropyl)dibenzo[b,e]oxepin-11(6H)-one (P60)**

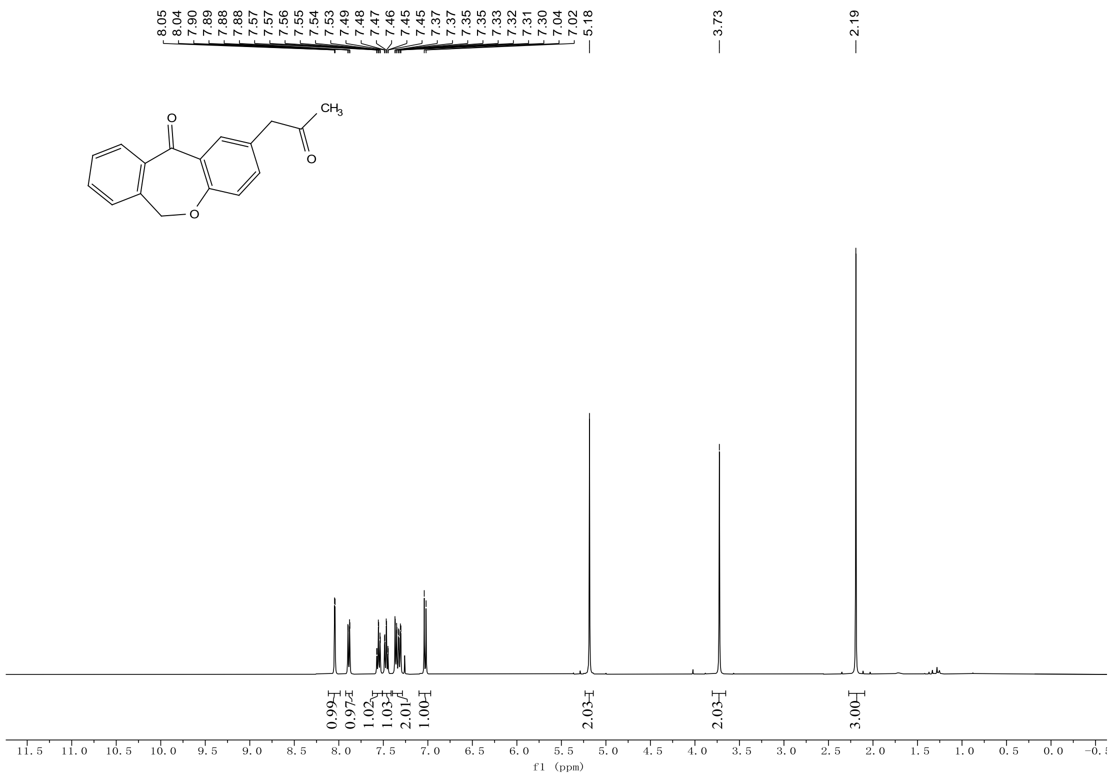

^{13}C NMR (101 MHz, $CDCl_3$) **2-(2-oxopropyl)dibenzo[b,e]oxepin-11(6H)-one (P60)**

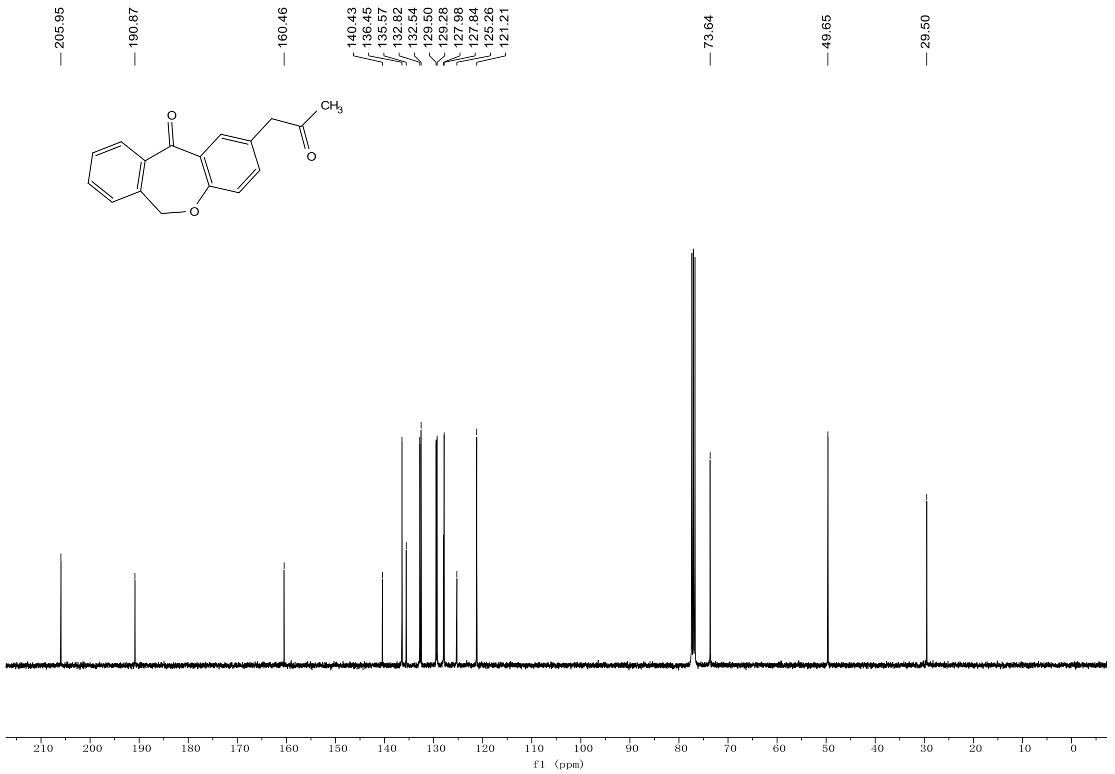

^{1}H NMR (400 MHz, $CDCl_3$) **(Z)-1-(5-fluoro-2-methyl-1-(4-(methylsulfinyl)benzylidene)-1H-inden-3-yl)propan-2-one (P61)**

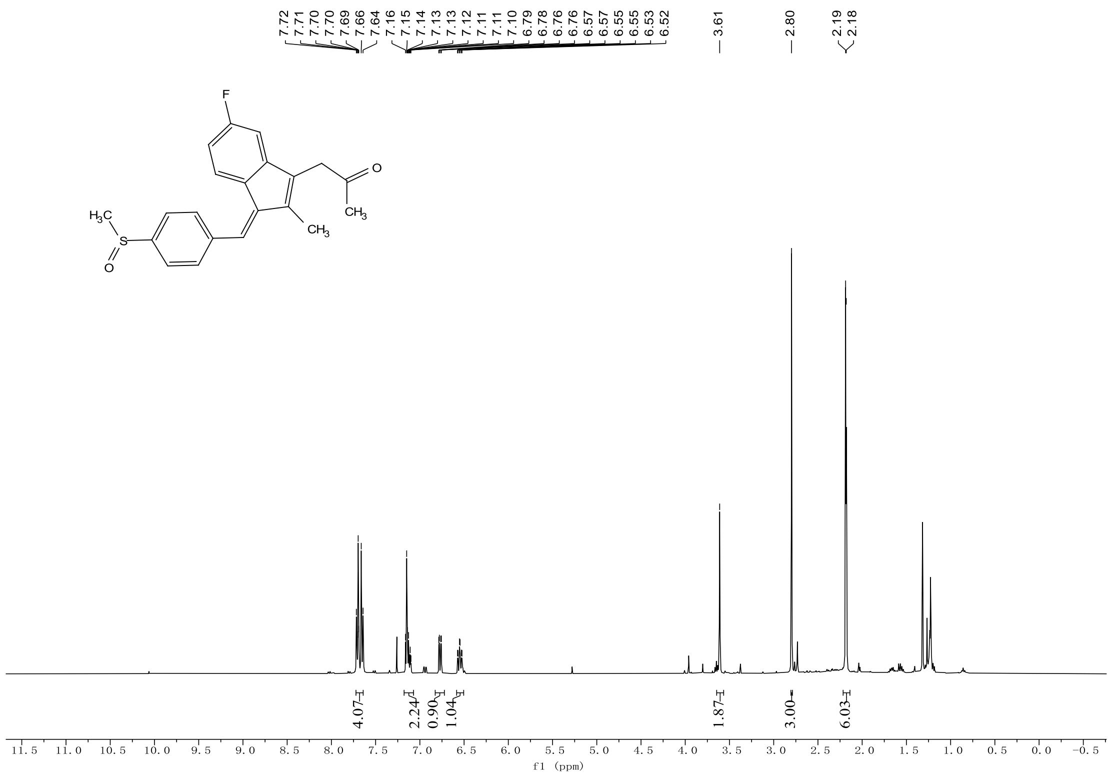

^{13}C NMR (101 MHz, $CDCl_3$) **(Z)-1-(5-fluoro-2-methyl-1-(4-(methylsulfinyl)benzylidene)-1H-inden-3-yl)propan-2-one (P61)**

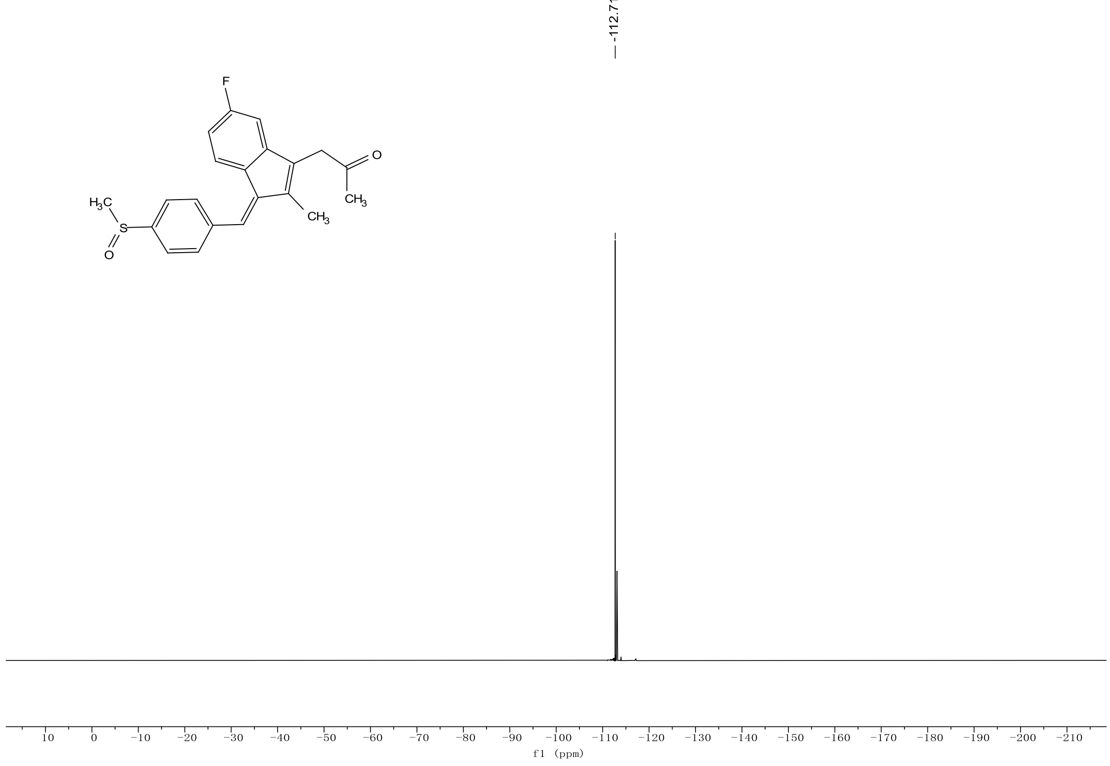

^{1}H NMR (400 MHz, $CDCl_3$) **2-methylnaphthalen-1-ol (P62)**

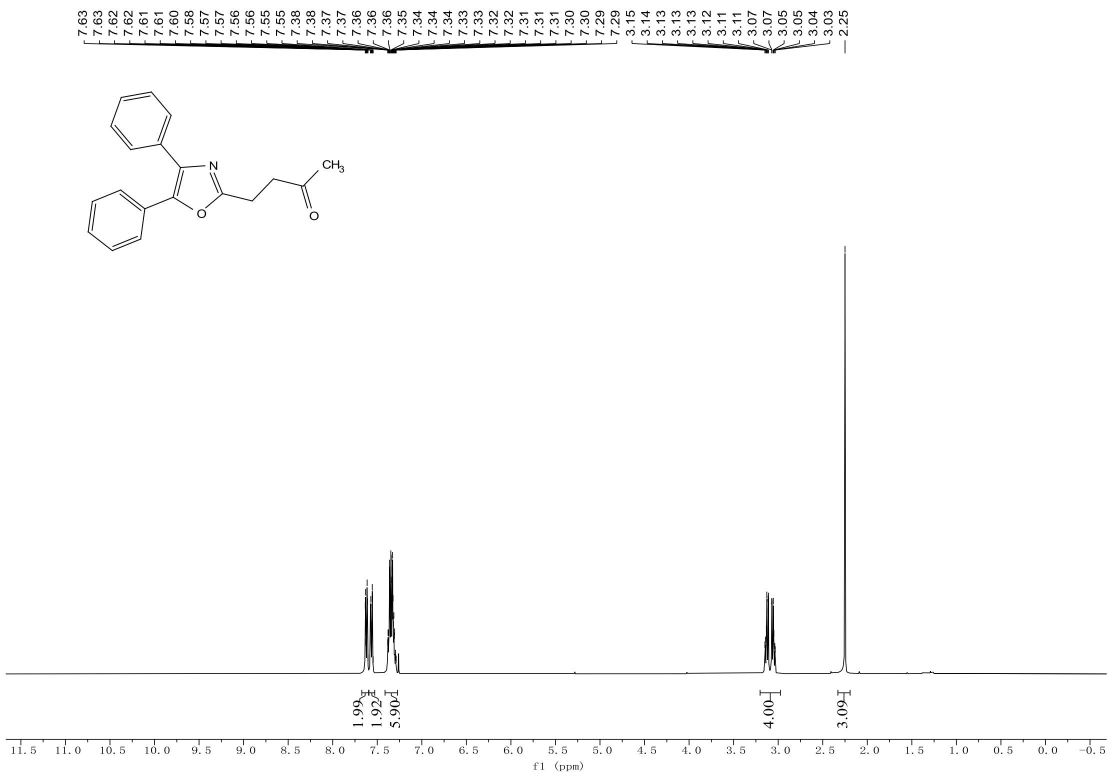

^{13}C NMR (101 MHz, $CDCl_3$) **2-methylnaphthalen-1-ol (P62)**

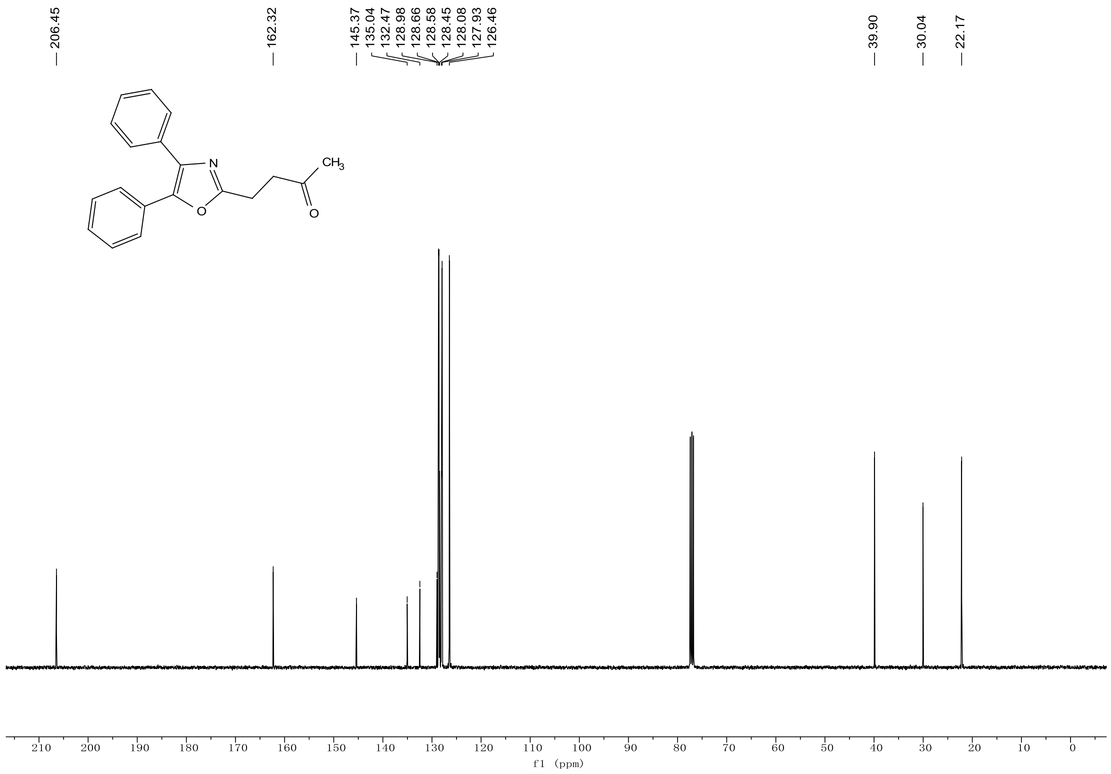

^{1}H NMR (400 MHz, $CDCl_3$) **2,6-dimethyloctadeca-2,17-dien-8-one (P63)**

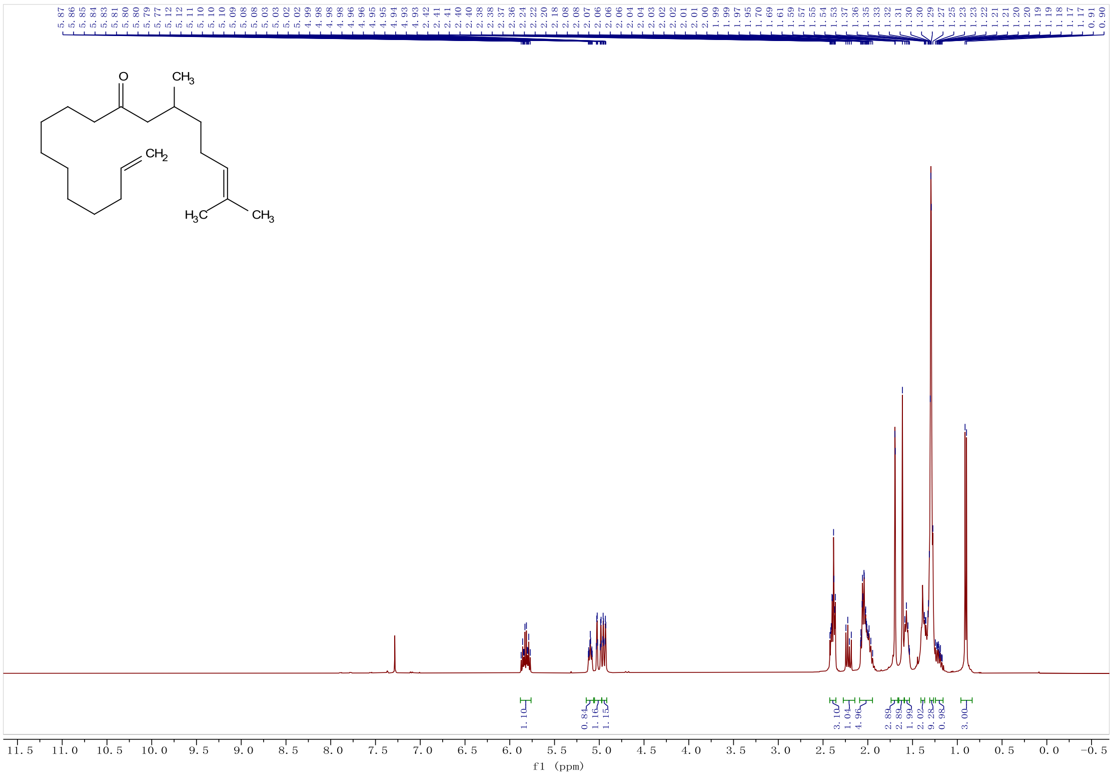

^{13}C NMR (101 MHz, $CDCl_3$) **2,6-dimethyloctadeca-2,17-dien-8-one (P63)**

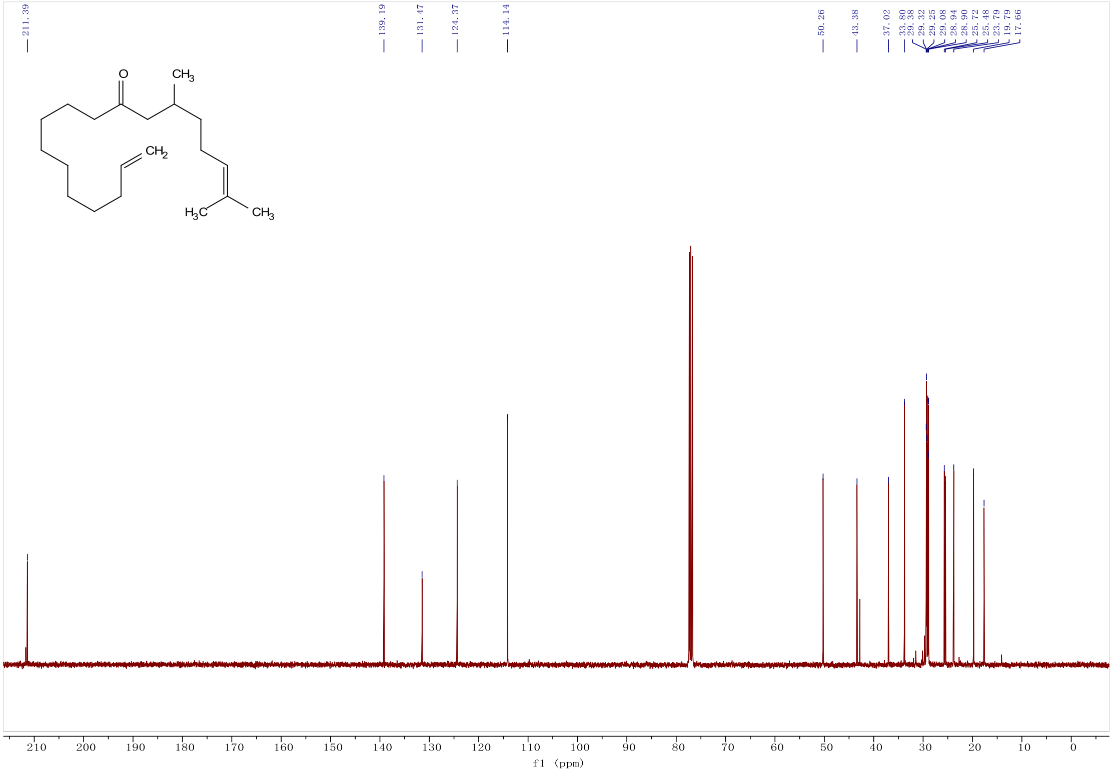

^{1}H NMR (400 MHz, $CDCl_3$) **1-(2-hydroxyphenyl)-3-phenylpropan-1-one (P64)**

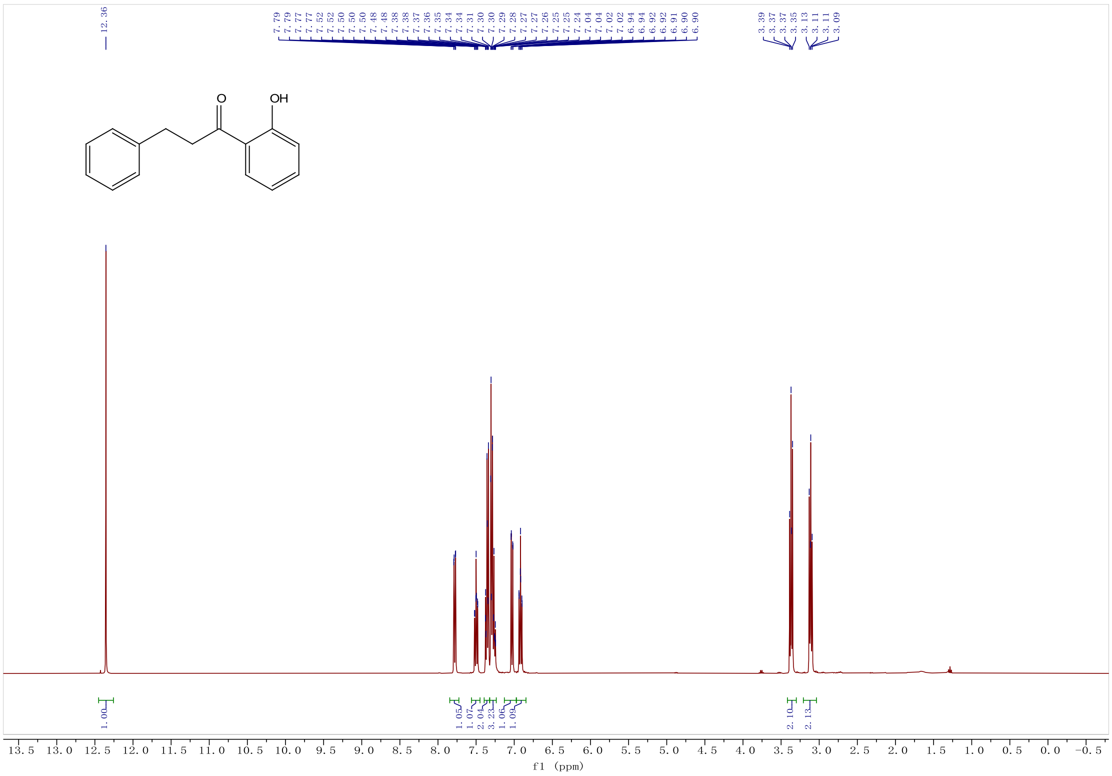

^{13}C NMR (101 MHz, $CDCl_3$) **1-(2-hydroxyphenyl)-3-phenylpropan-1-one (P64)**

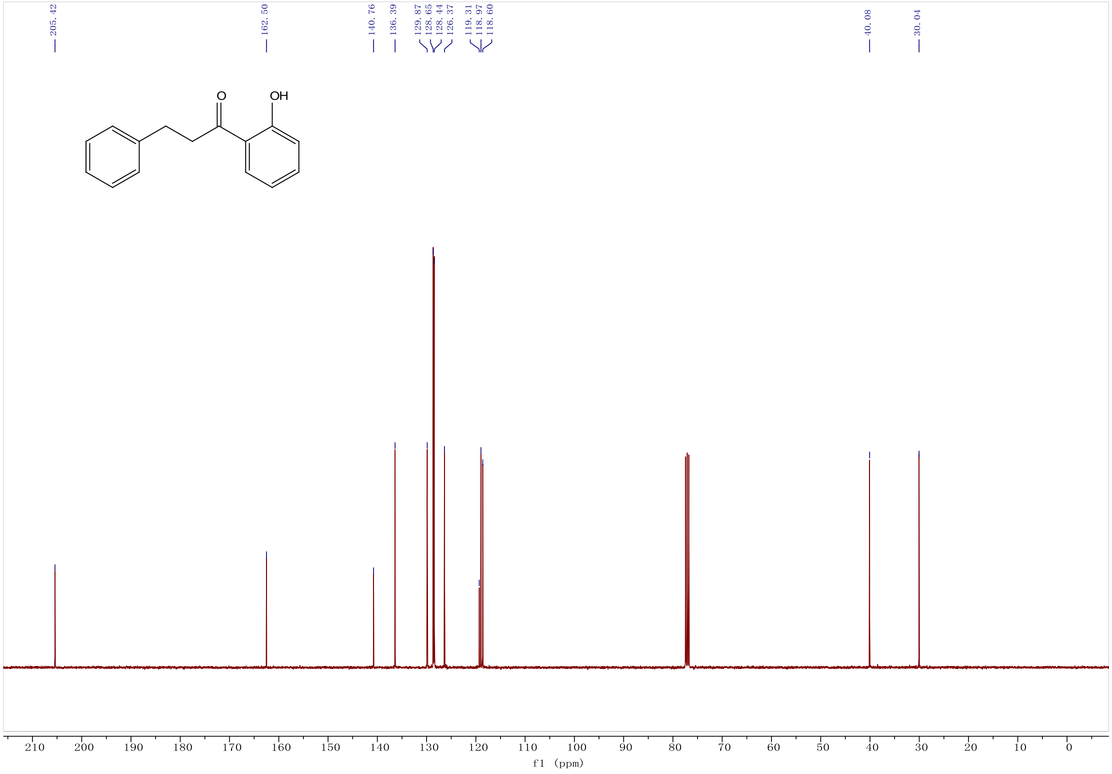

^{1}H NMR (400 MHz, $CDCl_3$) **2-methylnaphthalen-1-ol (P65)**

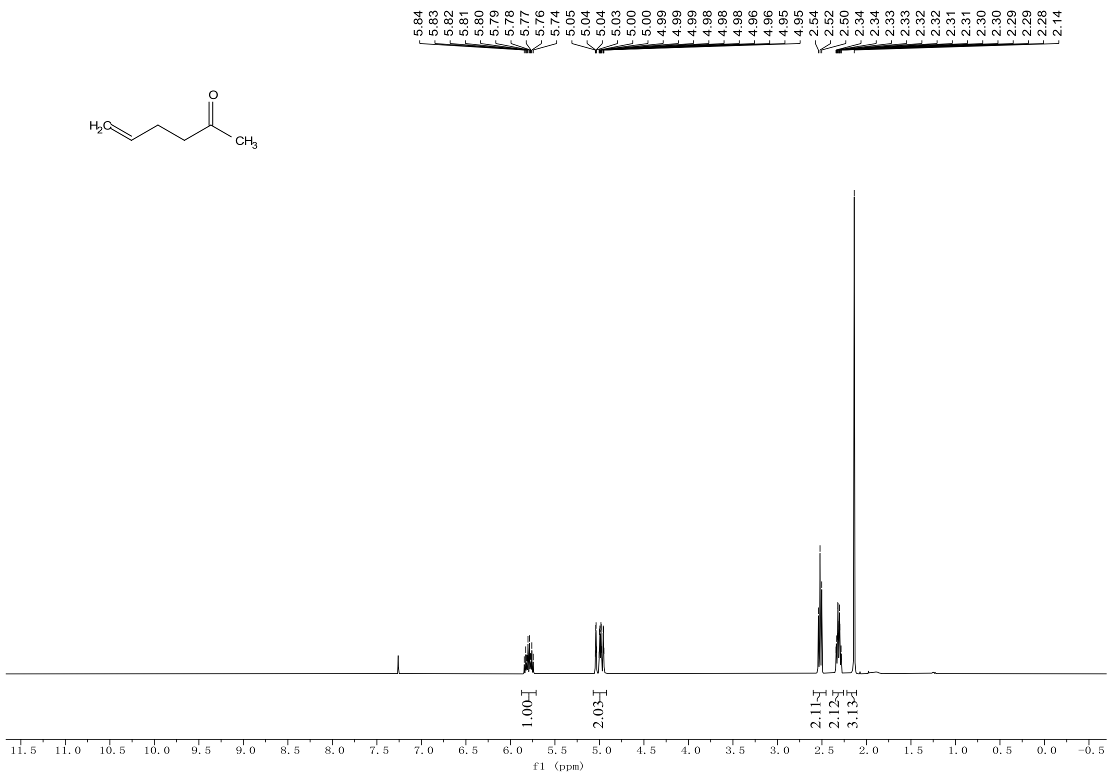

^{13}C NMR (101 MHz, $CDCl_3$) **2-methylnaphthalen-1-ol (P65)**

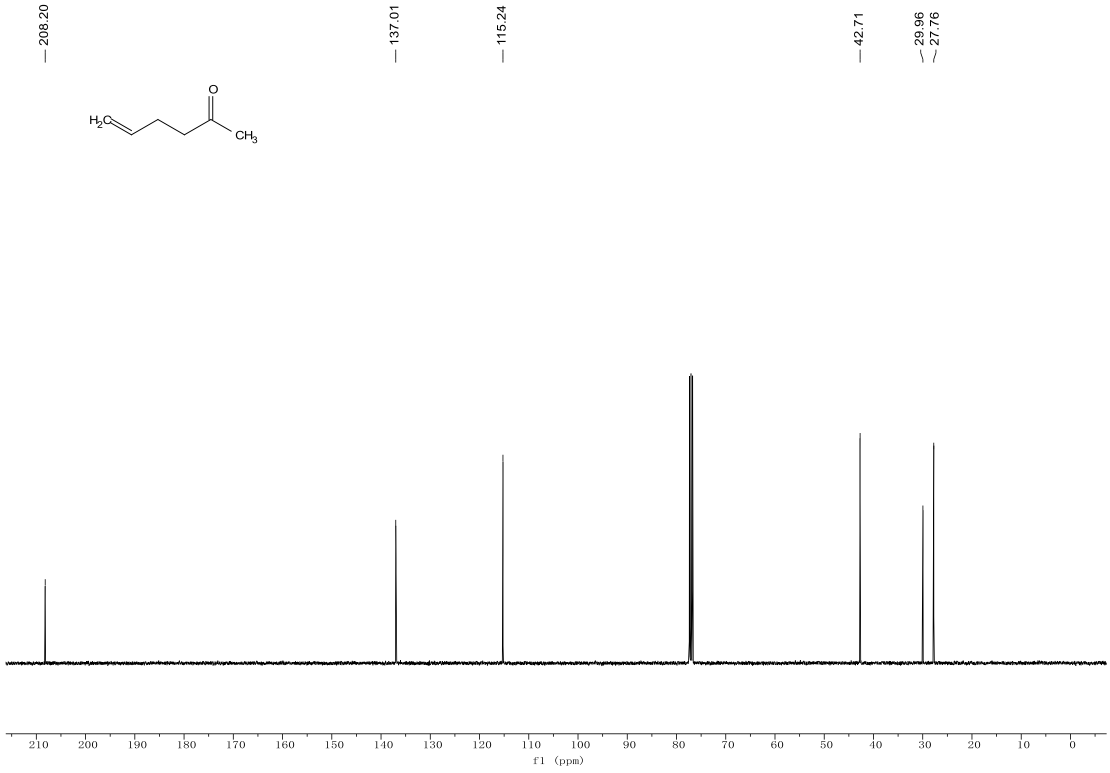

Supplement: SC-017-D5SC09414C-s001 [file SC-017-D5SC09414C-s001.pdf]
